# Supplementary material for: Modeling glioblastoma heterogeneity as a dynamic network of cell states
Source: Mol Syst Biol. 2021 Sep 16;17(9):e10105. doi: 10.15252/msb.202010105 (PMC8444284; doi:10.15252/msb.202010105)
Supplement: Supplementary file 1 — Appendix [file MSB-17-e10105-s003.pdf]

Modeling glioblastoma heterogeneity as a dynamic network of cell states

Larsson, Dalmo et al. 2021

Appendix

Considerations behind the experimental setup 3

Growth rates and seeding densities 3

Sampling interval and fraction of reseeding 4

Barcode library diversity 4

Comparison between barcoded and non-barcoded cells 5

Relation to in vivo assays 5

Supplementary Figures and Tables 7

Appendix Table S1 8

Appendix Table S2 9

Appendix Figure S1 10

Appendix Figure S2 11

Appendix Figure S3 12

Appendix Figure S4 13

Appendix Figure S5 14

Appendix Figure S6 15

Appendix Figure S7 16

Considerations behind the experimental setup

The goal of the STAG procedure is to estimate the rates of cell state transitions in a growing culture of cells (Figure 1A, main text). To estimate the rates of cell transitions with good accuracy, it is important to maximize the number of clones that can be traced in more than one experimental time point. Moreover, to get a representative sample of cell states (and thereby a relatively unbiased view of the transitions), it is also important to collect a random sample from all cells. The alternative, to use single-cell seeding, would result in a biased sample focusing on the fraction of cells that give rise to expandable cultures [1]. In the pooled barcoding setting, the progeny of cells which require the presence of other cells can also be traced. Further, our method allows us to determine not only state transitions back and forth, but also estimation of the individual growth rates of individual cell states. Implementing STAG, the single cell RNA sequencing capacity acts as the prime limiting factor and was here set to 10000 cells for each time point. Based on this limitation, we developed the experimental design as follows.

Growth rates and seeding densities

The cell growth rate, the fraction of cells re-seeded, and sampling interval (c.f. Figure 1) jointly define the final number of cells to be sequenced at each time-point. For this study, we used the Chromium Single Cell 3' Library Gel Bead Kit v3 protocol, with a loading of 16000 cells was used to achieve 10000 sequenced cells. Since some cells were lost due to washing and cell counting, we aimed at a cell population of 16000 – 20000 cells in the well as a maximum at the final time point. The cells grew exponentially (Methods) when kept at densities between 50-620 cells/mm^2 and the growth rate constant r was determined to 0.246 (95% confidence interval: 0.218 to 0.275) during the exponential growth phase. In the final experiment, cells ended up being seeded at a density of 78 cells/mm^2 and harvested at maximum 548 cells/mm^2 (Table A1) and the growth rate was estimated to 0.248. Cells were cultured in a 96 well format for the density to remain within the exponential growth boundaries while keeping the maximum total cell number within the right range. In the verification experiments, U3017MG and U3071MG growth rates were 0.228 (95% confidence interval: 0.215 - 0.240) and 0.129 (95% confidence interval: 0.102 - 0.156) and cells reached a maximum density of 384 and 193 cells/mm^2 respectively.

Sampling interval and fraction of reseeding

The sampling interval depends on the growth rate and fraction of cells reseeded, but also needs to be chosen to reflect the expected state transition rates of the cells. For instance, sampling times need to be sufficiently long so that transitions have time to occur and sufficiently frequent to capture intermediate states. We preferred a time scales of days, as this represent a typical time-scale for differentiation processes [2, 3], and also because we wanted to stay within the same order that of the cell division rate. Thus, we concluded that a sampling interval between 3 - 10 days would be desirable in our setting. The fraction of sampled cells (η, below) also needs to strike an appropriate trade-off. A very high η (close to 1) would likely cause depletion of the barcode pool, and a low η would generate data that are too sparse. We decided that 80% sampled cells would be a good cutoff. With the determined growth rate, the sampling interval became 7 days to not have a decreasing number of cells at each sampling time point. With r = 0.246 , 3 sampling time points at a t = 7 day interval and a 80% fraction of sampled cells, seeding of $N_0 = 2500$ barcoded cells would result in approximately 17540 cells at the final sampling point (Table A1), which fits into the interval 16000 – 20000.

Barcode library diversity

In the proposed protocol, cells are labelled with a pooled lentiviral barcode library, where random lentiviral particles will infect the cells. Thus, by chance alone, there will be a small fraction of cells labeled with more than one barcode, and also a small fraction of barcodes that get introduced to more than one cell. Both fractions should be kept low, which motivates the use of (i) a low multiplicity of infection (MOI) at the infection step and (ii) a high library barcode diversity in relation to the initial cell population seeded. For a thorough review of the statistical properties of pooled lentiviral barcoding, we refer to the work of Lan et al [4] which we used as a basis for our calculations. For the conducted experiments, cells were infected at a MOI of 0.1 and the Perturbseq library diversity was 10^5 [5]. Thus, when sorting out and seeding 2500 barcoded cells, 97.28% of these are expected to be uniquely barcoded, which corresponds to 2432 out of 2500 cells.

Comparison between barcoded and non-barcoded cells

The scRNA-seq data from day 0 for the cell line U3065MG contained a mix of barcoded and non-barcoded cells. This population of cells was used to compare the transcriptomic profile of cells with and without a barcode (**Figure S1**). The cells distributed similarly on the UMAP, showing no apparent batch effects or skewness of the transcriptome due to the barcoding. A total of 16 genes were differentially expressed between the two populations (adjusted p-value < 0.01, abs(log2FC) $>$ 0.25). This number can be compared to the average number of differentially expressed genes between states (>1000).

Relation to in vivo assays

In our implementation of STAG we rely on 2D cultures. The benefit of this is that a sufficient number of cells can be randomly sampled to support the estimation of cell state transitions (discussed above). Since the protocol includes only one sorting step of BFP-positive barcoded cells prior to the experiment, additional selection steps resulting in cell loss are avoided, facilitating the tracing of clones over multiple time points. As a comparison, Neftel et al [6] injected $2{*}10^4 - 10^5$ barcoded cells intracranially into mice and could after cell sorting and single cell RNA-sequencing retrieve data from 16 clones comprising a total of 72 barcoded cells. Cell recovery within this order of magnitude would not have sufficed to quantitatively evaluate state transitions as performed by the STAG model. While our detected cell states do overlap with gene signatures defined in clinical materials, embryonal brain, and other systems (Figure 3,5; main paper), we acknowledge that when performing the experiment in vitro in monolayer cultures, we generate a simplified 2D system lacking the impact of microenvironment and features related to the 3D structure of tumor tissue. Although our reductionistic approach does not recapitulate the complex in vivo conditions it allows us to study the cells intrinsic ability to transition between states. Likewise, when deciding to culture the cells adherently (2D) rather than as spheres (3D), we aimed at keeping the environment as similar as possible for all cells, to more accurately isolate the intrinsic cell state transition potential. In a 3D culture, the cells position within the sphere (peripheral or central) would potentially have acted as a hidden variable, since the position in the 3D structure could have an impact on cell state choice which eludes detection. We reserve an application of STAG to the 3D culture setting, with appropriate computational extensions, for future work.

References

[1] A. Segerman et al. "Clonal Variation in Drug and Radiation Response among Glioma-Initiating Cells Is Linked to Proneural-Mesenchymal Transition". In: *Cell Rep* 17.11 (Dec. 2016), pp. 2994–3009.

[2] K. P. L. Bhat et al. "Cancer CellMesenchymal differentiation mediated by NF-B promotes radiation resistance in glioblastoma". In: *Cancer Cell* 24.3 (Sept. 2013), pp. 331–346.

[3] V. Balasubramaniyan et al. "OncotargetAberrant mesenchymal differentiation of glioma stem-like cells: implications for therapeutic targeting". In: *Oncotarget* 6.31 (Oct. 2015), pp. 31007–31017.

[4] X. Lan et al. "Fate mapping of human glioblastoma reveals an invariant stem cell hierarchy". In: *Nature* 549.7671 (Sept. 2017), pp. 227–232.

[5] B. Adamson et al. "A Multiplexed Single-Cell CRISPR Screening Platform Enables Systematic Dissection of the Unfolded Protein Response". In: *Cell* 167.7 (Dec. 2016), pp. 1867–1882.

[6] C. Neftel et al. "An Integrative Model of Cellular States, Plasticity, and Genetics for Glioblastoma". In: *Cell* 178.4 (Aug. 2019), pp. 835–849.

Supplementary Figures and Tables

	Equation	Number of cells	Cells/mm^2
Cells seeded (N_0)	-	2500	78
Cells at day 7 (N_7)	N_0*e^(*r*t)	13995	437
Cells sampled at day 7	0.8*N_7	11196	-
Cells reseeded at day 7 (N_{7seed})	0.2*N_7	2799	87
Cells at day 14 (N_{14})	N_{7seed}*e^(*r*t)	15669	490
Cells sampled at day 14	0.8*N_{14}	12535	-
Cells reseeded at day 14 (N_{14seed})	0.2*N_{14}	3134	98
Cells sampled at day 21	N_{14seed}*e^(*r*t)	17543	548

Table S1. Summary of equations to calculate the number of cells at each time point. Cell numbers and cell densities are shown for U3065MG with growth rate constant r = 0.246 and time t = 7 days.

BD FACS AriaIII	Excitation	Emission	Voltage
FSC			27
SSC			280
405_(450/40)	405	450/40	291
488_502(530/30)	488	502(530/30)	297
633_755(780/60)	633	755(780/60)	533
BD LSR Fortessa	Excitation	Emission	Voltage
FSC			511
SSC			220
CD24-BV421	405	450/50	310
CD44-FITC	488	505(530/30)	340
HLA-DR-APC-Vio770	640	750(780/60)	200

Table S2. Optical configuration for the BD FACS AriaIII cell sorter and the BD LSR Fortessa flow cytometer.

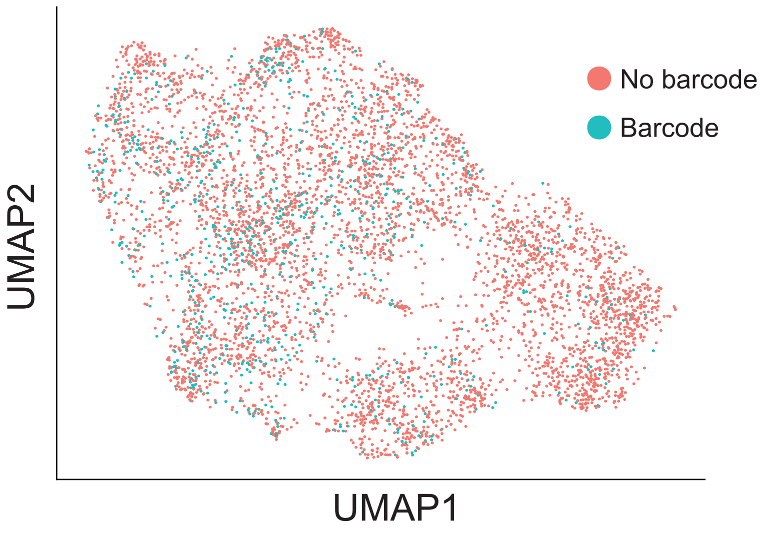

Figure S1: Comparison non-barcoded vs barcoded cells. UMAP embedding of cells with (blue) or without (red) barcode.

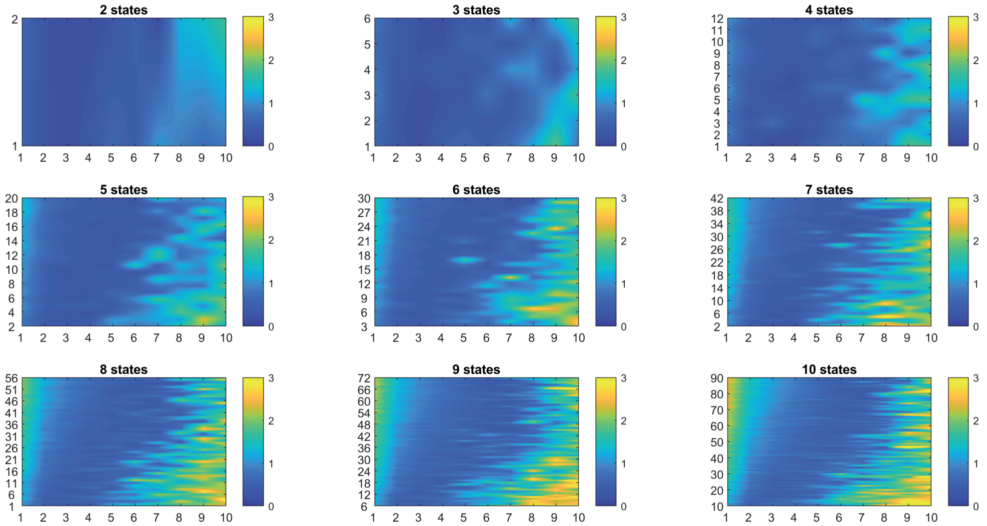

Figure S2: Benchmarking the transition and growth model. Mean distance (measured as the Frobenius norm) between simulated and estimated transition matrix under varying conditions. Number of states (k) was varied from 2-10 and for each number of state the connectivity was varied from minimum (1 transition) to a fully connected network (k*(k-1) transitions). For each connectivity condition, the sampling interval was varied from 1-10 days between samplings. For all simulations, three samplings was simulated (generating a three timepoint data set). The x-axis represent sampling interval (days) and y-axis the connectivity (number of allowed transitions).

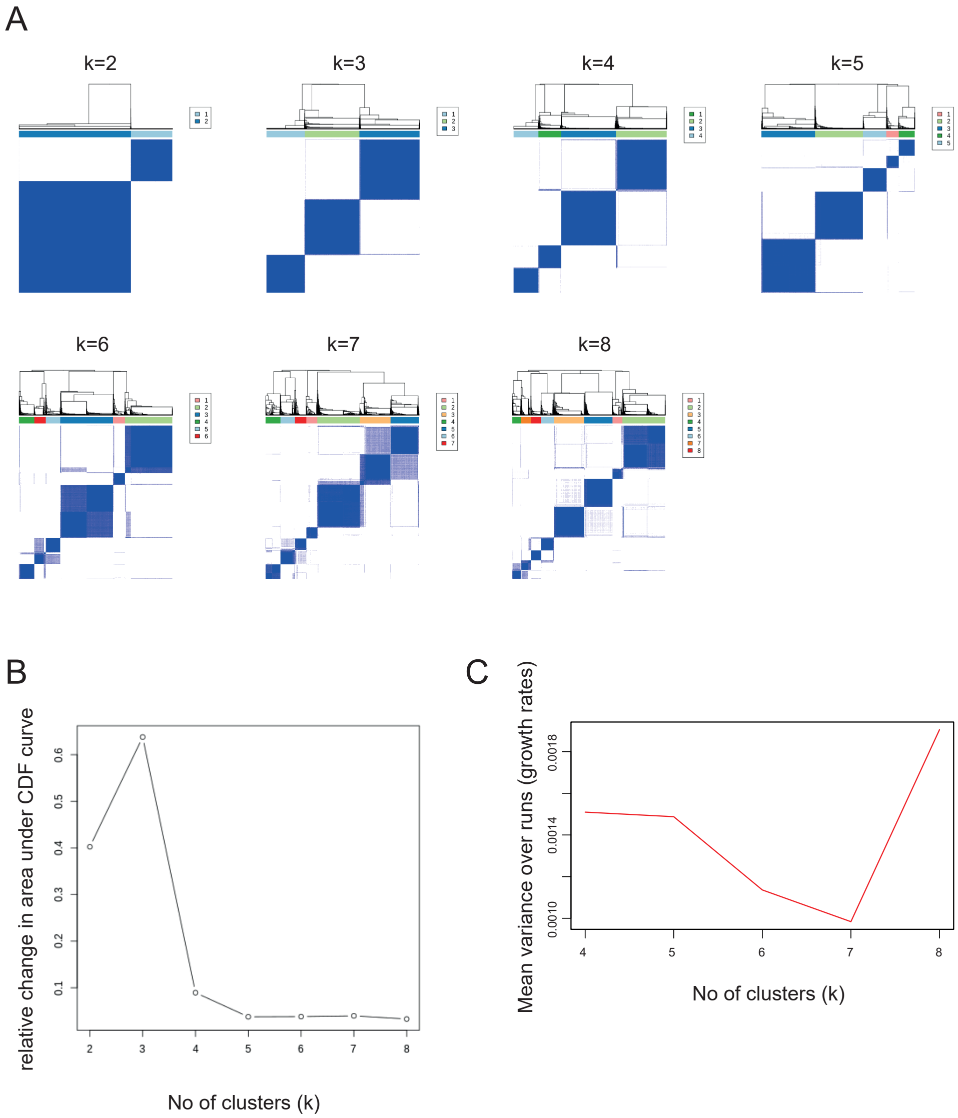

Figure S3: Choosing k (U3065MG). A) Consensus clustering maps for k=2-8 and B) delta area plot showing the relative change under the cumulative distribution function (CDF) curve as k increases from 2 to 8. C) Mean parameter variance over 100 bootstrapping runs for growth rates (column sum of A matrix) when k increases from 4 to 8. We noted that the variance was the smallest at k=6 and k=7. Preferring a simpler model over a more complex one, we focus on the case k=6.

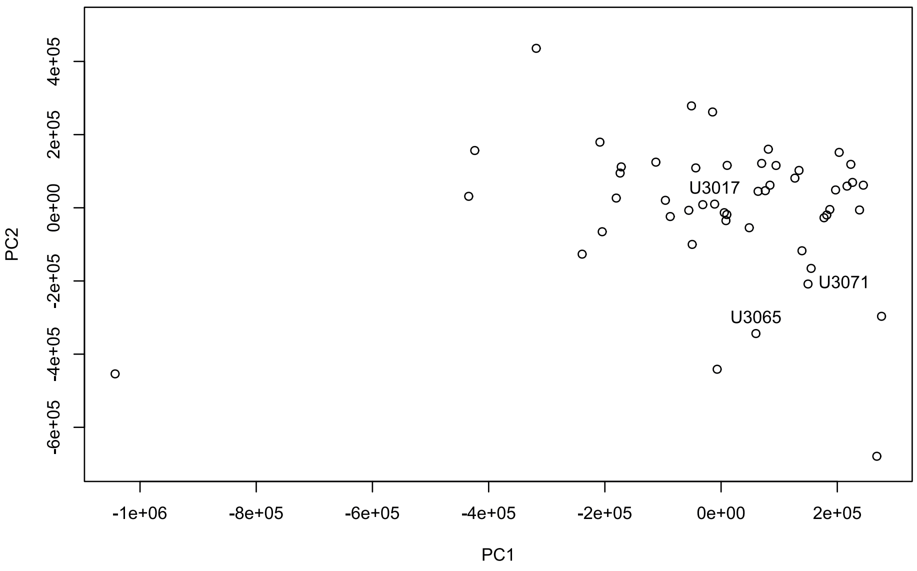

Figure S4: Choosing additional cell lines. Principal component analysis of bulk RNA sequencing data from patient derived GBM cell lines. Cell lines named (U3065MG, U3017MG, U3071MG) are those investigated in this project.

A

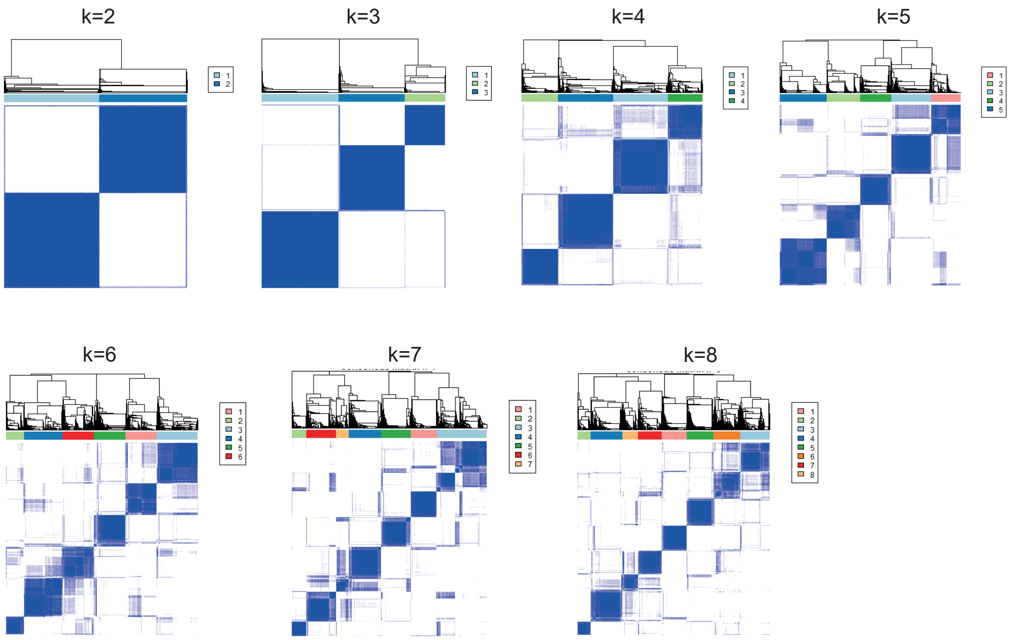

B

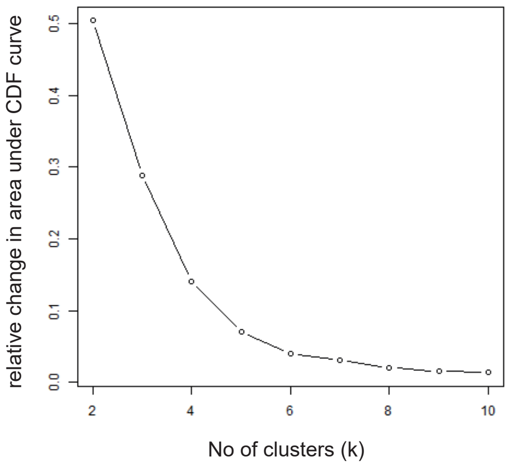

Figure S5: Choosing k (U3017MG). A) Consensus clustering maps for k=2-8 and B) delta area plot showing the relative change under the cumulative distribution function (CDF) curve as k increases from 2 to 10.

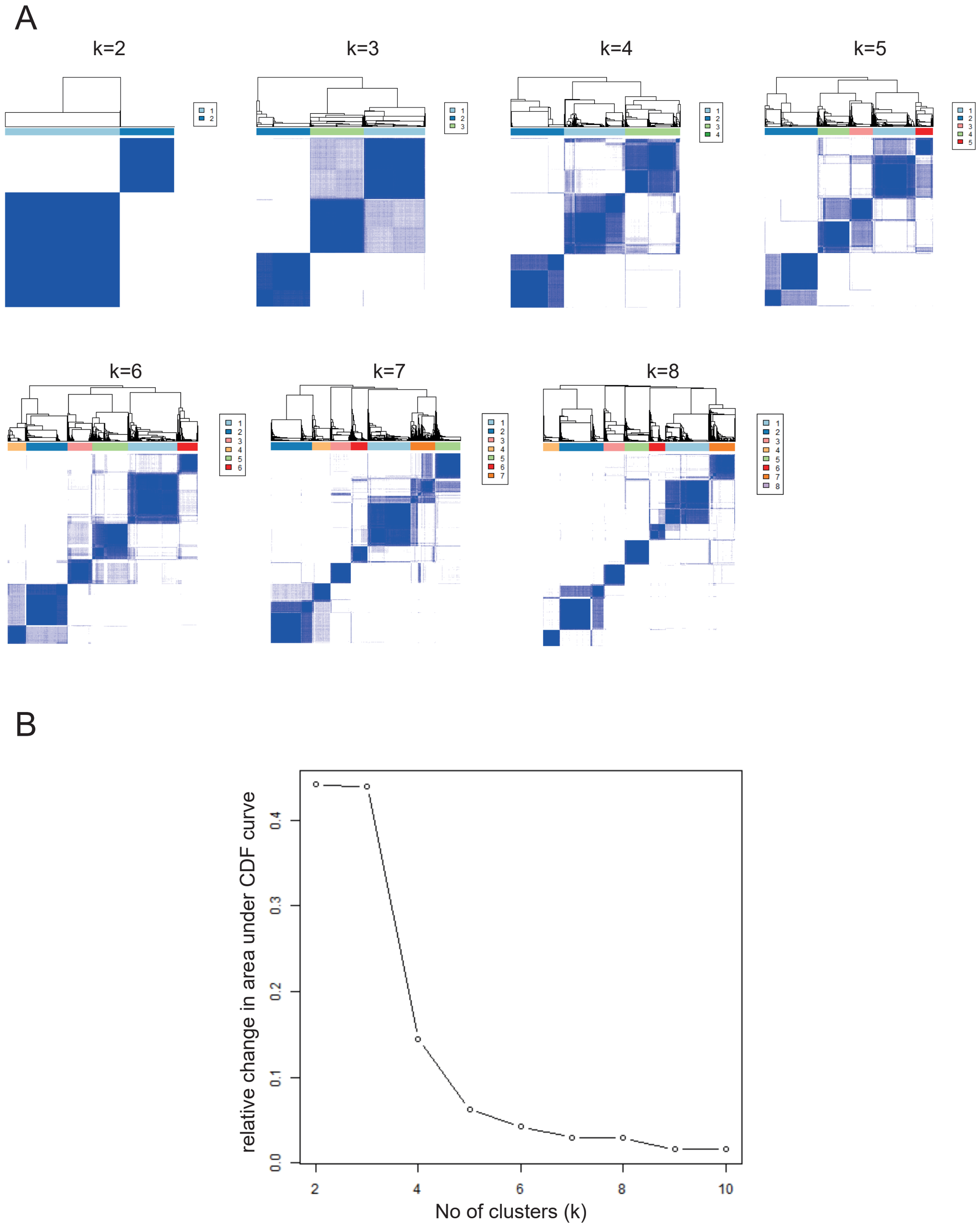

Figure S6: Choosing k (U3071MG). A) Consensus clustering maps for k=2-8 and B) delta area plot showing the relative change under the cumulative distribution function (CDF) curve as k increases from 2 to 10.

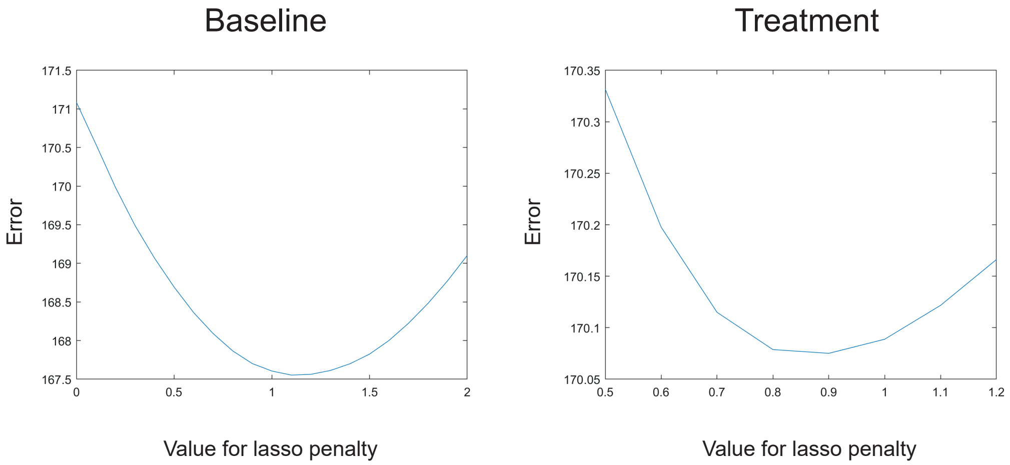

Figure S7: Choosing lasso penatly using cross validation. The value for the lasso penalty was decided using cross validation analysis, for the baseline (untreated) network and the treated networks.
