# Supplementary material for: Modeling glioblastoma heterogeneity as a dynamic network of cell states
Source: Mol Syst Biol. 2021 Sep 16;17(9):e10105. doi: 10.15252/msb.202010105 (PMC8444284; doi:10.15252/msb.202010105)
Supplement: Supplementary file 2 — Expanded View Figures PDF [file MSB-17-e10105-s005.pdf]

Expanded View Figures

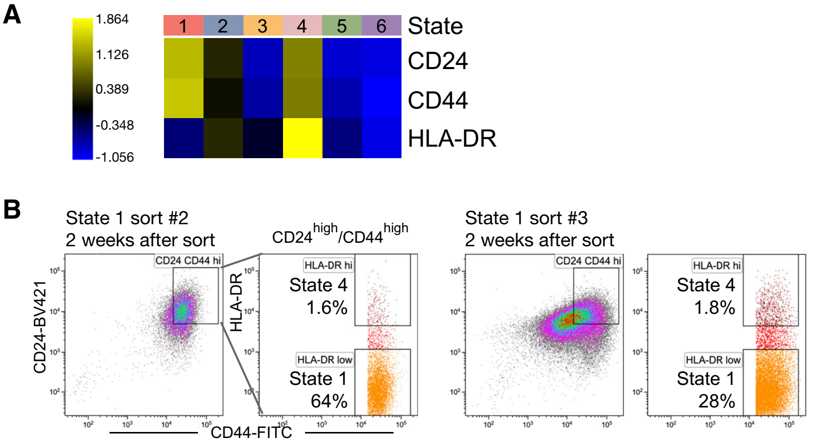

Figure EV1. Cell surface marker selection and extended FACS analyses.

A Heatmap of selected cell surface marker mRNA expression (*Z* scores) in each of the U3065MG states.

B Flow cytometry analysis of cell surface marker expression, 2 weeks after FACS-enrichment of U3065MG state 1 cells ($CD24^{high}/CD44^{high}/HLA\text{-}DR^{low}$), as in Fig 2G. Two biological replicate experiments are shown.

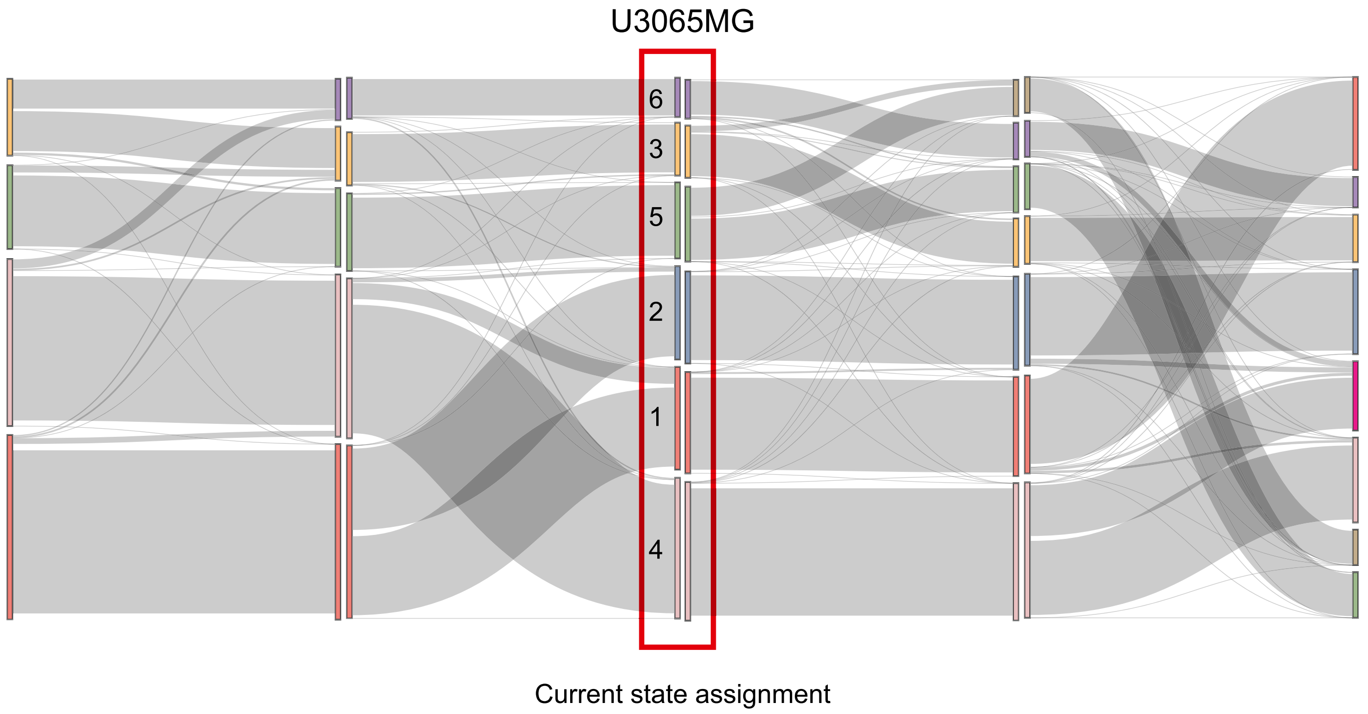

Figure EV2. Cluster robustness analysis U3065MG.

Sankey plot showing how cells change state assignment when *k* increases from 4 to 8. Inspecting the clustering solution for *k* between 4 and 8, the main trend is that increasing *k* by 1 tends to split a cluster in two, indicating that clusters are robust.

© 2021 The Authors

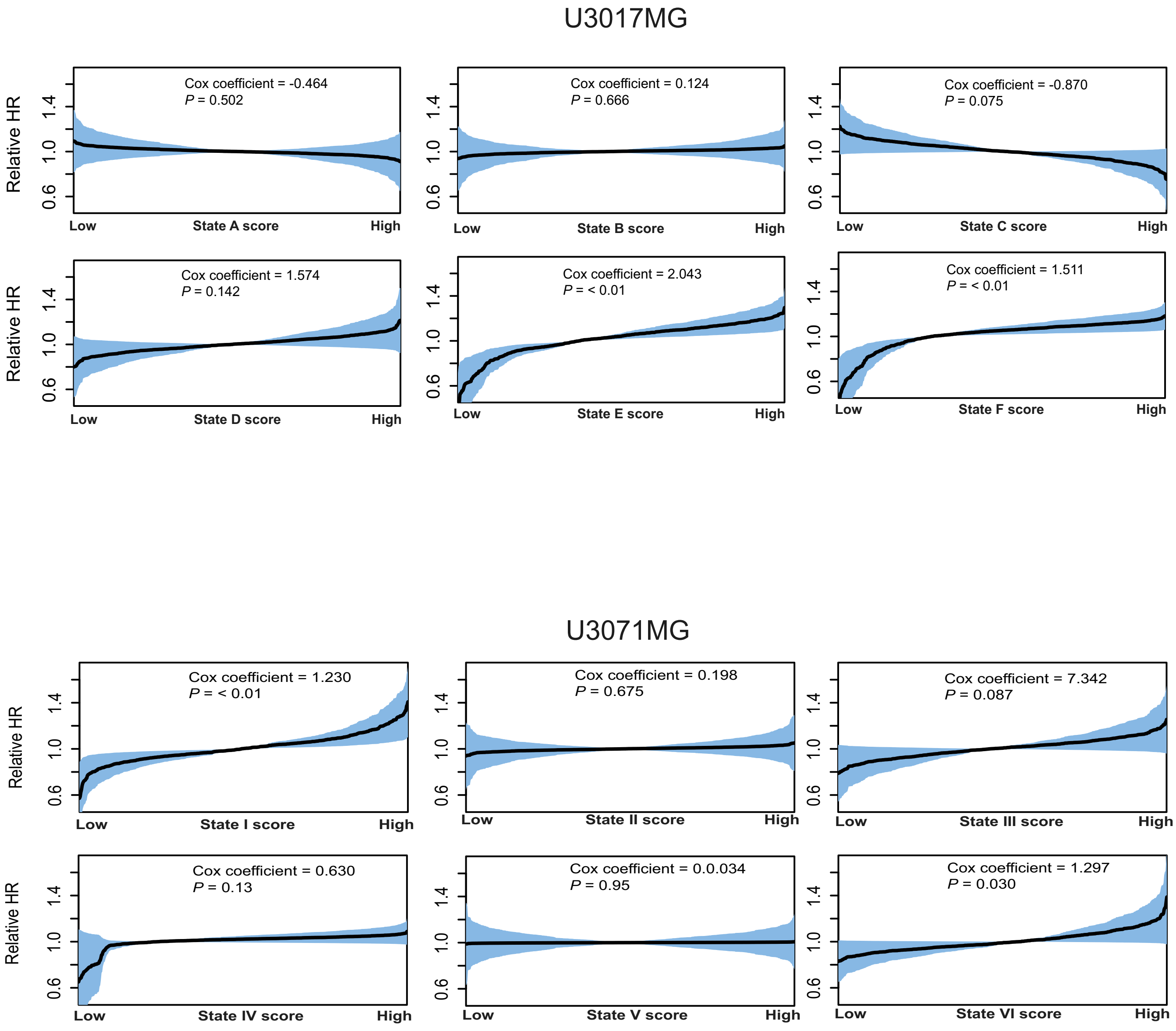

Figure EV3. Survival analysis U3017MG and U3071MG.

State signatures for states in U3017MG and U3071MG were related to the TCGA GBM cohort through ssGSEA and enrichment scores were used as independent covariate to build Cox's proportional hazards models. Shaded areas indicate 95% confidence intervals, calculated as $\pm$ 1.96 * standard error (SE).

© 2021 The Authors

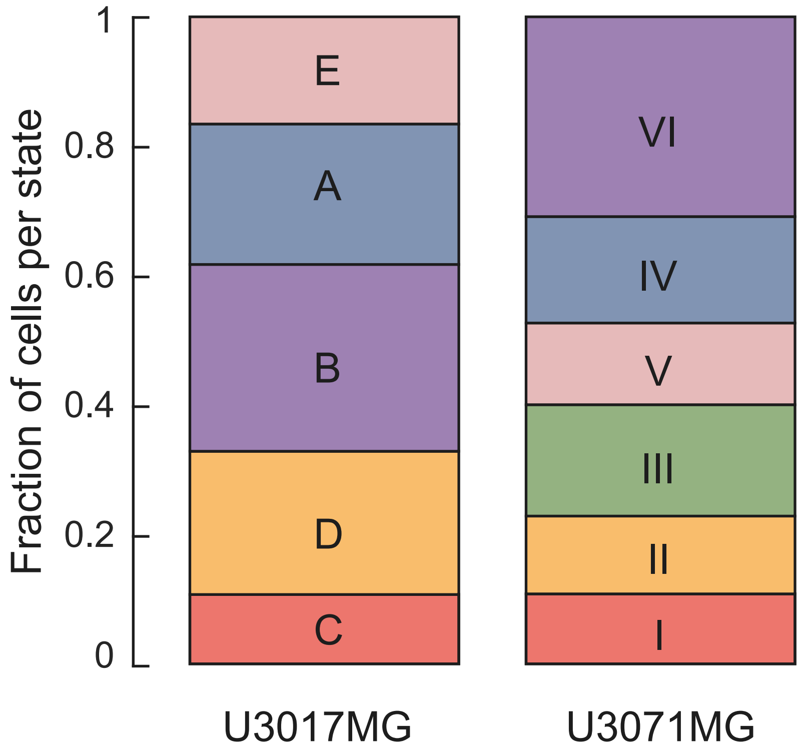

Figure EV4. State equilibria U3017MG and U3071MG.

Stacked barplots showing the predicted state equilibria for U3017MG states A–E and U3071MG states I–VI at steady state. The state F-fraction in U3017MG is < 0.001 and not shown in figure.

 © 2021 The Authors

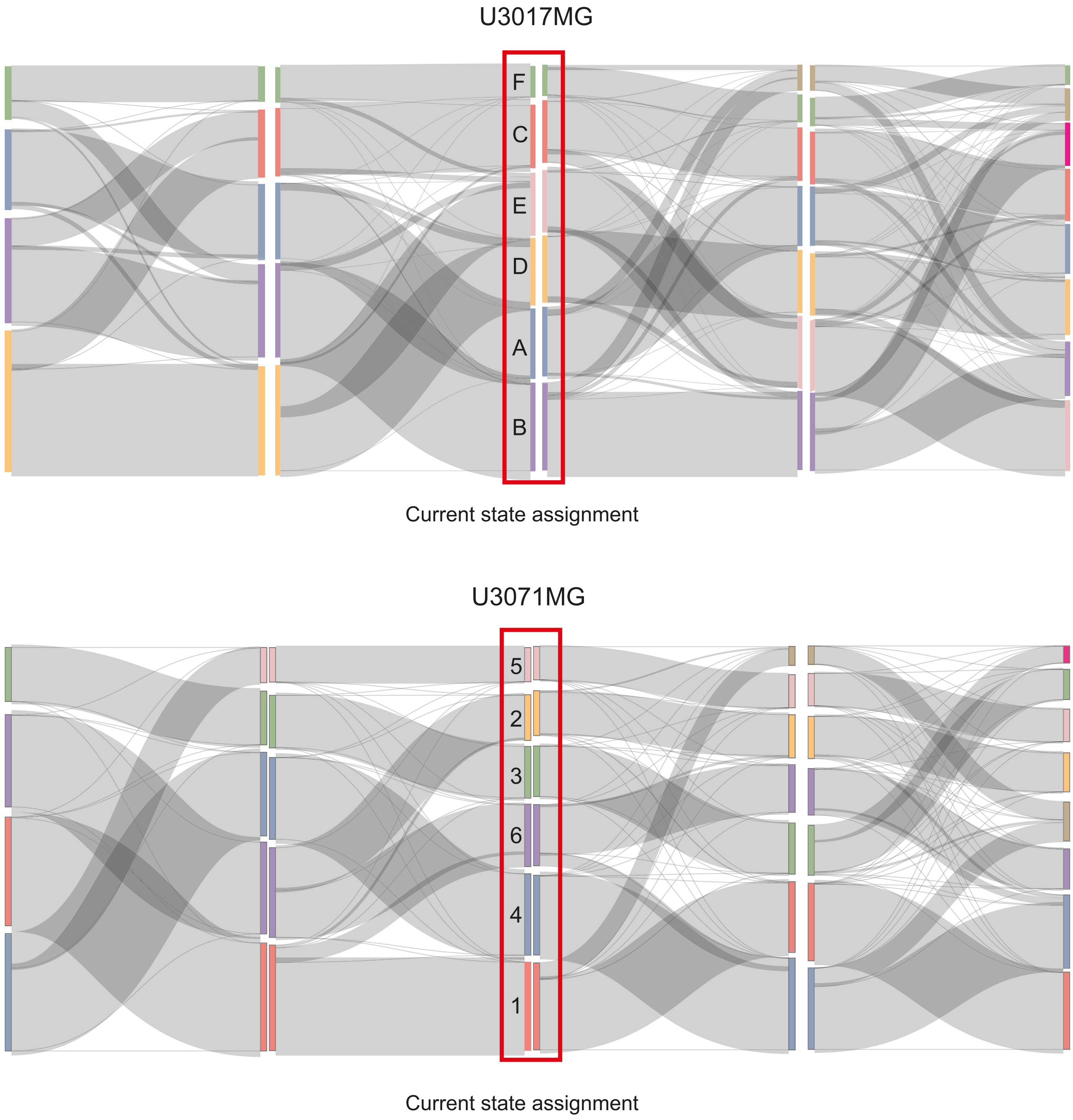

Figure EV5. Cluster robustness analysis U3017MG and U3071MG.

Sankey plot showing how cells change state assignment when *k* increases from 4 to 8.

© 2021 The Authors
