# Supplementary material for: Modeling glioblastoma heterogeneity as a dynamic network of cell states
Source: Mol Syst Biol. 2021 Sep 16;17(9):e10105. doi: 10.15252/msb.202010105 (PMC8444284; doi:10.15252/msb.202010105)
Supplement: Supplementary file 5 — Source Data for Figure 3 [file MSB-17-e10105-s001.zip › Figure3A_sourcedata/GSEA_3065/hallmarks_state1.GseaPreranked.1623416262439/gsea_report_for_na_neg_1623416262439.html]

Report for na\_neg 1623416262439 [GSEA]

| GS  follow link to MSigDB | GS DETAILS | SIZE | ES | NES | NOM p-val | FDR q-val | FWER p-val | RANK AT MAX | LEADING EDGE || 1 | HALLMARK\_ANGIOGENESIS | Details ... | 22 | -0.54 | -1.42 | 0.071 | 0.446 | 0.546 | 905 | tags=50%, list=9%, signal=55% |
| 2 | HALLMARK\_KRAS\_SIGNALING\_DN | Details ... | 37 | -0.44 | -1.32 | 0.099 | 0.472 | 0.821 | 2097 | tags=41%, list=21%, signal=51% |
| 3 | HALLMARK\_CHOLESTEROL\_HOMEOSTASIS | Details ... | 63 | -0.39 | -1.28 | 0.101 | 0.425 | 0.896 | 955 | tags=22%, list=10%, signal=24% |
| 4 | HALLMARK\_WNT\_BETA\_CATENIN\_SIGNALING | Details ... | 28 | -0.44 | -1.24 | 0.185 | 0.410 | 0.937 | 1305 | tags=29%, list=13%, signal=33% |
| 5 | HALLMARK\_MYOGENESIS | Details ... | 101 | -0.35 | -1.23 | 0.107 | 0.358 | 0.955 | 1275 | tags=26%, list=13%, signal=29% |
| 6 | HALLMARK\_NOTCH\_SIGNALING | Details ... | 24 | -0.44 | -1.21 | 0.213 | 0.358 | 0.977 | 940 | tags=29%, list=10%, signal=32% |
| 7 | HALLMARK\_COMPLEMENT | Details ... | 105 | -0.30 | -1.07 | 0.343 | 0.693 | 1.000 | 927 | tags=17%, list=9%, signal=19% |
| 8 | HALLMARK\_INFLAMMATORY\_RESPONSE | Details ... | 76 | -0.31 | -1.05 | 0.366 | 0.649 | 1.000 | 1569 | tags=26%, list=16%, signal=31% |
| 9 | HALLMARK\_BILE\_ACID\_METABOLISM | Details ... | 58 | -0.32 | -1.04 | 0.384 | 0.619 | 1.000 | 2704 | tags=38%, list=27%, signal=52% |
| 10 | HALLMARK\_TNFA\_SIGNALING\_VIA\_NFKB | Details ... | 119 | -0.28 | -1.01 | 0.412 | 0.642 | 1.000 | 732 | tags=15%, list=7%, signal=16% |
| 11 | HALLMARK\_ESTROGEN\_RESPONSE\_EARLY | Details ... | 113 | -0.28 | -0.99 | 0.469 | 0.645 | 1.000 | 643 | tags=13%, list=7%, signal=14% |
| 12 | HALLMARK\_UV\_RESPONSE\_DN | Details ... | 114 | -0.27 | -0.96 | 0.561 | 0.673 | 1.000 | 1616 | tags=25%, list=16%, signal=30% |
| 13 | HALLMARK\_PROTEIN\_SECRETION | Details ... | 89 | -0.26 | -0.91 | 0.643 | 0.757 | 1.000 | 2102 | tags=33%, list=21%, signal=41% |
| 14 | HALLMARK\_INTERFERON\_ALPHA\_RESPONSE | Details ... | 62 | -0.28 | -0.89 | 0.649 | 0.742 | 1.000 | 800 | tags=13%, list=8%, signal=14% |
| 15 | HALLMARK\_HEME\_METABOLISM | Details ... | 132 | -0.24 | -0.87 | 0.787 | 0.734 | 1.000 | 1306 | tags=18%, list=13%, signal=21% |
Table: Gene sets enriched in phenotype **na**[plain text format]****

  
