# Supplementary material for: Modeling glioblastoma heterogeneity as a dynamic network of cell states
Source: Mol Syst Biol. 2021 Sep 16;17(9):e10105. doi: 10.15252/msb.202010105 (PMC8444284; doi:10.15252/msb.202010105)
Supplement: Supplementary file 5 — Source Data for Figure 3 [file MSB-17-e10105-s001.zip › Figure3A_sourcedata/GSEA_3065/hallmarks_state1.GseaPreranked.1623416262439/gsea_report_for_na_pos_1623416262439.html]

Report for na\_pos 1623416262439 [GSEA]

| GS  follow link to MSigDB | GS DETAILS | SIZE | ES | NES | NOM p-val | FDR q-val | FWER p-val | RANK AT MAX | LEADING EDGE || 1 | HALLMARK\_MYC\_TARGETS\_V1 | Details ... | 195 | 0.68 | 2.70 | 0.000 | 0.000 | 0.000 | 1835 | tags=62%, list=19%, signal=74% |
| 2 | HALLMARK\_OXIDATIVE\_PHOSPHORYLATION | Details ... | 194 | 0.65 | 2.52 | 0.000 | 0.000 | 0.000 | 1615 | tags=53%, list=16%, signal=62% |
| 3 | HALLMARK\_REACTIVE\_OXYGEN\_SPECIES\_PATHWAY | Details ... | 38 | 0.71 | 2.16 | 0.000 | 0.000 | 0.000 | 1307 | tags=53%, list=13%, signal=60% |
| 4 | HALLMARK\_MYC\_TARGETS\_V2 | Details ... | 51 | 0.55 | 1.77 | 0.002 | 0.008 | 0.020 | 2417 | tags=47%, list=24%, signal=62% |
| 5 | HALLMARK\_PI3K\_AKT\_MTOR\_SIGNALING | Details ... | 83 | 0.48 | 1.68 | 0.000 | 0.014 | 0.047 | 1474 | tags=34%, list=15%, signal=39% |
| 6 | HALLMARK\_FATTY\_ACID\_METABOLISM | Details ... | 111 | 0.46 | 1.68 | 0.000 | 0.012 | 0.049 | 1515 | tags=26%, list=15%, signal=31% |
| 7 | HALLMARK\_DNA\_REPAIR | Details ... | 133 | 0.43 | 1.61 | 0.002 | 0.019 | 0.088 | 1760 | tags=32%, list=18%, signal=39% |
| 8 | HALLMARK\_MTORC1\_SIGNALING | Details ... | 180 | 0.41 | 1.59 | 0.000 | 0.021 | 0.107 | 1720 | tags=37%, list=17%, signal=44% |
| 9 | HALLMARK\_E2F\_TARGETS | Details ... | 192 | 0.41 | 1.59 | 0.000 | 0.020 | 0.114 | 2028 | tags=34%, list=21%, signal=42% |
| 10 | HALLMARK\_G2M\_CHECKPOINT | Details ... | 187 | 0.40 | 1.58 | 0.000 | 0.019 | 0.123 | 2044 | tags=30%, list=21%, signal=38% |
| 11 | HALLMARK\_HYPOXIA | Details ... | 138 | 0.39 | 1.51 | 0.002 | 0.033 | 0.218 | 1250 | tags=27%, list=13%, signal=30% |
| 12 | HALLMARK\_GLYCOLYSIS | Details ... | 153 | 0.39 | 1.50 | 0.002 | 0.033 | 0.236 | 1097 | tags=22%, list=11%, signal=25% |
| 13 | HALLMARK\_EPITHELIAL\_MESENCHYMAL\_TRANSITION | Details ... | 131 | 0.37 | 1.38 | 0.023 | 0.098 | 0.582 | 517 | tags=16%, list=5%, signal=17% |
| 14 | HALLMARK\_ANDROGEN\_RESPONSE | Details ... | 75 | 0.39 | 1.35 | 0.059 | 0.111 | 0.658 | 1025 | tags=28%, list=10%, signal=31% |
| 15 | HALLMARK\_APOPTOSIS | Details ... | 116 | 0.35 | 1.29 | 0.068 | 0.169 | 0.826 | 1121 | tags=24%, list=11%, signal=27% |
| 16 | HALLMARK\_UV\_RESPONSE\_UP | Details ... | 120 | 0.35 | 1.28 | 0.053 | 0.170 | 0.849 | 1389 | tags=23%, list=14%, signal=26% |
| 17 | HALLMARK\_ADIPOGENESIS | Details ... | 161 | 0.33 | 1.25 | 0.043 | 0.196 | 0.904 | 1845 | tags=32%, list=19%, signal=38% |
| 18 | HALLMARK\_P53\_PATHWAY | Details ... | 146 | 0.33 | 1.24 | 0.091 | 0.207 | 0.927 | 1482 | tags=25%, list=15%, signal=29% |
| 19 | HALLMARK\_INTERFERON\_GAMMA\_RESPONSE | Details ... | 103 | 0.33 | 1.21 | 0.132 | 0.247 | 0.966 | 1622 | tags=26%, list=16%, signal=31% |
| 20 | HALLMARK\_ESTROGEN\_RESPONSE\_LATE | Details ... | 117 | 0.33 | 1.20 | 0.134 | 0.241 | 0.967 | 1710 | tags=30%, list=17%, signal=36% |
| 21 | HALLMARK\_APICAL\_JUNCTION |  | 119 | 0.32 | 1.20 | 0.120 | 0.238 | 0.968 | 1515 | tags=25%, list=15%, signal=29% |
| 22 | HALLMARK\_KRAS\_SIGNALING\_UP |  | 90 | 0.33 | 1.17 | 0.164 | 0.263 | 0.980 | 1463 | tags=32%, list=15%, signal=37% |
| 23 | HALLMARK\_XENOBIOTIC\_METABOLISM |  | 106 | 0.31 | 1.12 | 0.238 | 0.355 | 0.995 | 1564 | tags=25%, list=16%, signal=30% |
| 24 | HALLMARK\_UNFOLDED\_PROTEIN\_RESPONSE |  | 101 | 0.31 | 1.10 | 0.253 | 0.375 | 0.998 | 1393 | tags=24%, list=14%, signal=27% |
| 25 | HALLMARK\_COAGULATION |  | 60 | 0.32 | 1.09 | 0.299 | 0.382 | 0.999 | 828 | tags=20%, list=8%, signal=22% |
| 26 | HALLMARK\_IL2\_STAT5\_SIGNALING |  | 112 | 0.30 | 1.09 | 0.295 | 0.373 | 0.999 | 1691 | tags=31%, list=17%, signal=37% |
| 27 | HALLMARK\_PEROXISOME |  | 73 | 0.31 | 1.06 | 0.354 | 0.428 | 1.000 | 1571 | tags=21%, list=16%, signal=24% |
| 28 | HALLMARK\_APICAL\_SURFACE |  | 23 | 0.38 | 1.05 | 0.376 | 0.416 | 1.000 | 1497 | tags=39%, list=15%, signal=46% |
| 29 | HALLMARK\_TGF\_BETA\_SIGNALING |  | 48 | 0.33 | 1.03 | 0.402 | 0.457 | 1.000 | 1390 | tags=29%, list=14%, signal=34% |
| 30 | HALLMARK\_ALLOGRAFT\_REJECTION |  | 68 | 0.28 | 0.95 | 0.559 | 0.642 | 1.000 | 1803 | tags=32%, list=18%, signal=39% |
| 31 | HALLMARK\_SPERMATOGENESIS |  | 59 | 0.28 | 0.93 | 0.588 | 0.669 | 1.000 | 1232 | tags=17%, list=12%, signal=19% |
| 32 | HALLMARK\_HEDGEHOG\_SIGNALING |  | 27 | 0.31 | 0.89 | 0.631 | 0.755 | 1.000 | 1131 | tags=22%, list=11%, signal=25% |
| 33 | HALLMARK\_MITOTIC\_SPINDLE |  | 182 | 0.22 | 0.88 | 0.789 | 0.741 | 1.000 | 1858 | tags=24%, list=19%, signal=29% |
| 34 | HALLMARK\_IL6\_JAK\_STAT3\_SIGNALING |  | 35 | 0.25 | 0.77 | 0.851 | 0.915 | 1.000 | 1569 | tags=29%, list=16%, signal=34% |
Table: Gene sets enriched in phenotype **na**[plain text format]****

  
