# Supplementary material for: Modeling glioblastoma heterogeneity as a dynamic network of cell states
Source: Mol Syst Biol. 2021 Sep 16;17(9):e10105. doi: 10.15252/msb.202010105 (PMC8444284; doi:10.15252/msb.202010105)
Supplement: Supplementary file 5 — Source Data for Figure 3 [file MSB-17-e10105-s001.zip › Figure3A_sourcedata/GSEA_3065/hallmarks_state1.GseaPreranked.1623416262439/HALLMARK_ADIPOGENESIS.html]

Details for gene set HALLMARK\_ADIPOGENESIS[GSEA]

|  || Dataset | state1 |
| Phenotype | NoPhenotypeAvailable |
| Upregulated in class | na\_pos |
| GeneSet | HALLMARK\_ADIPOGENESIS |
| Enrichment Score (ES) | 0.32877705 |
| Normalized Enrichment Score (NES) | 1.2541436 |
| Nominal p-value | 0.042986427 |
| FDR q-value | 0.19601256 |
| FWER p-Value | 0.904 |
Table: GSEA Results Summary

  

Fig 1: Enrichment plot: HALLMARK\_ADIPOGENESIS      
 Profile of the Running ES Score & Positions of GeneSet Members on the Rank Ordered List

  

| PROBE | GENE SYMBOL | GENE\_TITLE | RANK IN GENE LIST | RANK METRIC SCORE | RUNNING ES | CORE ENRICHMENT || 1 | CAVIN1 |  |  | 18 | 0.641 | 0.0367 | Yes |
| 2 | TKT |  |  | 90 | 0.384 | 0.0525 | Yes |
| 3 | MRPL15 |  |  | 165 | 0.313 | 0.0637 | Yes |
| 4 | UQCR10 |  |  | 177 | 0.307 | 0.0810 | Yes |
| 5 | SOD1 |  |  | 186 | 0.303 | 0.0984 | Yes |
| 6 | GPX4 |  |  | 234 | 0.283 | 0.1106 | Yes |
| 7 | MYLK |  |  | 260 | 0.275 | 0.1245 | Yes |
| 8 | ECHS1 |  |  | 283 | 0.266 | 0.1383 | Yes |
| 9 | COX8A |  |  | 301 | 0.260 | 0.1521 | Yes |
| 10 | MDH2 |  |  | 333 | 0.248 | 0.1639 | Yes |
| 11 | UQCRQ |  |  | 438 | 0.220 | 0.1664 | Yes |
| 12 | ARL4A |  |  | 497 | 0.206 | 0.1728 | Yes |
| 13 | CYC1 |  |  | 511 | 0.203 | 0.1837 | Yes |
| 14 | COX7B |  |  | 526 | 0.200 | 0.1943 | Yes |
| 15 | SDHB |  |  | 606 | 0.185 | 0.1973 | Yes |
| 16 | MGLL |  |  | 633 | 0.181 | 0.2055 | Yes |
| 17 | DDT |  |  | 636 | 0.181 | 0.2162 | Yes |
| 18 | ELOVL6 |  |  | 690 | 0.174 | 0.2212 | Yes |
| 19 | GHITM |  |  | 726 | 0.168 | 0.2277 | Yes |
| 20 | ATP5PO |  |  | 772 | 0.162 | 0.2327 | Yes |
| 21 | UQCRC1 |  |  | 773 | 0.161 | 0.2424 | Yes |
| 22 | CHCHD10 |  |  | 849 | 0.151 | 0.2438 | Yes |
| 23 | NDUFB7 |  |  | 854 | 0.151 | 0.2525 | Yes |
| 24 | NDUFAB1 |  |  | 887 | 0.146 | 0.2580 | Yes |
| 25 | GRPEL1 |  |  | 904 | 0.144 | 0.2650 | Yes |
| 26 | UQCR11 |  |  | 918 | 0.143 | 0.2722 | Yes |
| 27 | RAB34 |  |  | 947 | 0.140 | 0.2778 | Yes |
| 28 | YWHAG |  |  | 984 | 0.135 | 0.2822 | Yes |
| 29 | PRDX3 |  |  | 1003 | 0.133 | 0.2884 | Yes |
| 30 | ATL2 |  |  | 1033 | 0.130 | 0.2932 | Yes |
| 31 | GADD45A |  |  | 1082 | 0.124 | 0.2958 | Yes |
| 32 | TALDO1 |  |  | 1097 | 0.123 | 0.3017 | Yes |
| 33 | AK2 |  |  | 1167 | 0.116 | 0.3016 | Yes |
| 34 | MTCH2 |  |  | 1193 | 0.113 | 0.3058 | Yes |
| 35 | IDH3A |  |  | 1199 | 0.112 | 0.3121 | Yes |
| 36 | PLIN2 |  |  | 1226 | 0.110 | 0.3160 | Yes |
| 37 | QDPR |  |  | 1242 | 0.109 | 0.3210 | Yes |
| 38 | CMPK1 |  |  | 1284 | 0.105 | 0.3231 | Yes |
| 39 | FAH |  |  | 1378 | 0.097 | 0.3194 | Yes |
| 40 | ACAA2 |  |  | 1425 | 0.094 | 0.3203 | Yes |
| 41 | RNF11 |  |  | 1499 | 0.089 | 0.3181 | Yes |
| 42 | CRAT |  |  | 1515 | 0.088 | 0.3219 | Yes |
| 43 | DHCR7 |  |  | 1553 | 0.086 | 0.3232 | Yes |
| 44 | DNAJC15 |  |  | 1660 | 0.078 | 0.3170 | Yes |
| 45 | ATP1B3 |  |  | 1673 | 0.077 | 0.3204 | Yes |
| 46 | ESRRA |  |  | 1708 | 0.075 | 0.3214 | Yes |
| 47 | GBE1 |  |  | 1769 | 0.071 | 0.3195 | Yes |
| 48 | G3BP2 |  |  | 1771 | 0.071 | 0.3237 | Yes |
| 49 | SUCLG1 |  |  | 1795 | 0.070 | 0.3255 | Yes |
| 50 | TANK |  |  | 1815 | 0.069 | 0.3277 | Yes |
| 51 | UCP2 |  |  | 1845 | 0.067 | 0.3288 | Yes |
| 52 | ALDOA |  |  | 1936 | 0.063 | 0.3233 | No |
| 53 | COQ9 |  |  | 2149 | 0.053 | 0.3047 | No |
| 54 | PREB |  |  | 2156 | 0.053 | 0.3073 | No |
| 55 | NDUFS3 |  |  | 2186 | 0.052 | 0.3074 | No |
| 56 | COX6A1 |  |  | 2260 | 0.049 | 0.3028 | No |
| 57 | DLAT |  |  | 2282 | 0.048 | 0.3035 | No |
| 58 | COQ5 |  |  | 2295 | 0.047 | 0.3051 | No |
| 59 | NDUFA5 |  |  | 2339 | 0.046 | 0.3034 | No |
| 60 | ECH1 |  |  | 2355 | 0.045 | 0.3046 | No |
| 61 | PIM3 |  |  | 2582 | 0.037 | 0.2836 | No |
| 62 | PFKL |  |  | 2621 | 0.035 | 0.2818 | No |
| 63 | ETFB |  |  | 2629 | 0.035 | 0.2832 | No |
| 64 | UCK1 |  |  | 2638 | 0.035 | 0.2845 | No |
| 65 | BCL2L13 |  |  | 2644 | 0.035 | 0.2860 | No |
| 66 | UBQLN1 |  |  | 2649 | 0.035 | 0.2877 | No |
| 67 | SLC1A5 |  |  | 2665 | 0.034 | 0.2882 | No |
| 68 | SLC25A10 |  |  | 2722 | 0.032 | 0.2844 | No |
| 69 | NABP1 |  |  | 2782 | 0.031 | 0.2801 | No |
| 70 | SAMM50 |  |  | 2898 | 0.028 | 0.2700 | No |
| 71 | COQ3 |  |  | 2964 | 0.026 | 0.2648 | No |
| 72 | DLD |  |  | 2976 | 0.025 | 0.2652 | No |
| 73 | PFKFB3 |  |  | 3039 | 0.024 | 0.2603 | No |
| 74 | SCP2 |  |  | 3047 | 0.024 | 0.2610 | No |
| 75 | CS |  |  | 3094 | 0.023 | 0.2576 | No |
| 76 | IDH3G |  |  | 3238 | 0.020 | 0.2441 | No |
| 77 | JAGN1 |  |  | 3305 | 0.018 | 0.2384 | No |
| 78 | TOB1 |  |  | 3439 | 0.015 | 0.2256 | No |
| 79 | PEX14 |  |  | 3537 | 0.013 | 0.2164 | No |
| 80 | ARAF |  |  | 3643 | 0.011 | 0.2063 | No |
| 81 | HADH |  |  | 3732 | 0.009 | 0.1978 | No |
| 82 | RMDN3 |  |  | 3891 | 0.006 | 0.1819 | No |
| 83 | CHUK |  |  | 3957 | 0.005 | 0.1755 | No |
| 84 | TST |  |  | 4000 | 0.004 | 0.1714 | No |
| 85 | ANGPT1 |  |  | 4060 | 0.003 | 0.1656 | No |
| 86 | VEGFB |  |  | 4079 | 0.003 | 0.1639 | No |
| 87 | MGST3 |  |  | 4220 | 0.000 | 0.1495 | No |
| 88 | STOM |  |  | 4388 | -0.003 | 0.1325 | No |
| 89 | ITSN1 |  |  | 4527 | -0.005 | 0.1186 | No |
| 90 | CD151 |  |  | 4546 | -0.005 | 0.1171 | No |
| 91 | DRAM2 |  |  | 4641 | -0.007 | 0.1078 | No |
| 92 | UBC |  |  | 4739 | -0.008 | 0.0983 | No |
| 93 | RIOK3 |  |  | 4837 | -0.010 | 0.0889 | No |
| 94 | IMMT |  |  | 4844 | -0.010 | 0.0889 | No |
| 95 | ADIPOR2 |  |  | 4936 | -0.012 | 0.0802 | No |
| 96 | CAT |  |  | 4951 | -0.012 | 0.0795 | No |
| 97 | DECR1 |  |  | 5098 | -0.014 | 0.0653 | No |
| 98 | PHYH |  |  | 5120 | -0.014 | 0.0640 | No |
| 99 | SLC25A1 |  |  | 5164 | -0.015 | 0.0605 | No |
| 100 | SDHC |  |  | 5201 | -0.015 | 0.0577 | No |
| 101 | AIFM1 |  |  | 5241 | -0.016 | 0.0546 | No |
| 102 | CPT2 |  |  | 5269 | -0.016 | 0.0528 | No |
| 103 | SOWAHC |  |  | 5274 | -0.016 | 0.0534 | No |
| 104 | PGM1 |  |  | 5339 | -0.017 | 0.0479 | No |
| 105 | ACO2 |  |  | 5411 | -0.019 | 0.0417 | No |
| 106 | PPM1B |  |  | 5448 | -0.019 | 0.0391 | No |
| 107 | BAZ2A |  |  | 5450 | -0.019 | 0.0402 | No |
| 108 | ESYT1 |  |  | 5497 | -0.020 | 0.0367 | No |
| 109 | PEMT |  |  | 5528 | -0.021 | 0.0348 | No |
| 110 | GPAM |  |  | 5568 | -0.021 | 0.0321 | No |
| 111 | CCNG2 |  |  | 5718 | -0.024 | 0.0182 | No |
| 112 | ACADM |  |  | 5968 | -0.029 | -0.0057 | No |
| 113 | MIGA2 |  |  | 6027 | -0.030 | -0.0099 | No |
| 114 | NMT1 |  |  | 6122 | -0.031 | -0.0177 | No |
| 115 | MAP4K3 |  |  | 6252 | -0.034 | -0.0289 | No |
| 116 | ACLY |  |  | 6411 | -0.037 | -0.0430 | No |
| 117 | NKIRAS1 |  |  | 6569 | -0.040 | -0.0567 | No |
| 118 | CMBL |  |  | 6644 | -0.042 | -0.0618 | No |
| 119 | LPCAT3 |  |  | 6671 | -0.042 | -0.0620 | No |
| 120 | SLC5A6 |  |  | 6681 | -0.043 | -0.0603 | No |
| 121 | GPD2 |  |  | 6736 | -0.044 | -0.0632 | No |
| 122 | SSPN |  |  | 6940 | -0.048 | -0.0812 | No |
| 123 | REEP5 |  |  | 6947 | -0.049 | -0.0789 | No |
| 124 | RETSAT |  |  | 6959 | -0.049 | -0.0771 | No |
| 125 | DBT |  |  | 7000 | -0.050 | -0.0782 | No |
| 126 | SLC19A1 |  |  | 7027 | -0.051 | -0.0778 | No |
| 127 | DNAJB9 |  |  | 7191 | -0.055 | -0.0913 | No |
| 128 | MCCC1 |  |  | 7195 | -0.055 | -0.0882 | No |
| 129 | SCARB1 |  |  | 7359 | -0.060 | -0.1014 | No |
| 130 | PDCD4 |  |  | 7452 | -0.062 | -0.1072 | No |
| 131 | GPAT4 |  |  | 7617 | -0.067 | -0.1201 | No |
| 132 | CDKN2C |  |  | 7660 | -0.068 | -0.1203 | No |
| 133 | ACOX1 |  |  | 7691 | -0.069 | -0.1192 | No |
| 134 | DGAT1 |  |  | 7770 | -0.072 | -0.1229 | No |
| 135 | ELMOD3 |  |  | 8003 | -0.081 | -0.1419 | No |
| 136 | AGPAT3 |  |  | 8118 | -0.086 | -0.1484 | No |
| 137 | DHRS7 |  |  | 8136 | -0.087 | -0.1450 | No |
| 138 | APOE |  |  | 8322 | -0.095 | -0.1583 | No |
| 139 | ALDH2 |  |  | 8436 | -0.101 | -0.1638 | No |
| 140 | SORBS1 |  |  | 8508 | -0.106 | -0.1648 | No |
| 141 | ABCA1 |  |  | 8590 | -0.111 | -0.1664 | No |
| 142 | ABCB8 |  |  | 8621 | -0.113 | -0.1627 | No |
| 143 | ITGA7 |  |  | 8639 | -0.114 | -0.1577 | No |
| 144 | IFNGR1 |  |  | 8689 | -0.116 | -0.1557 | No |
| 145 | BCL6 |  |  | 8706 | -0.118 | -0.1503 | No |
| 146 | DHRS7B |  |  | 8743 | -0.121 | -0.1467 | No |
| 147 | PTCD3 |  |  | 8758 | -0.122 | -0.1408 | No |
| 148 | ADCY6 |  |  | 8807 | -0.125 | -0.1382 | No |
| 149 | SLC27A1 |  |  | 8936 | -0.137 | -0.1431 | No |
| 150 | HIBCH |  |  | 9087 | -0.151 | -0.1495 | No |
| 151 | LIFR |  |  | 9216 | -0.168 | -0.1525 | No |
| 152 | POR |  |  | 9415 | -0.204 | -0.1606 | No |
| 153 | PPP1R15B |  |  | 9494 | -0.224 | -0.1552 | No |
| 154 | PHLDB1 |  |  | 9671 | -0.294 | -0.1556 | No |
| 155 | APLP2 |  |  | 9736 | -0.344 | -0.1415 | No |
| 156 | IDH1 |  |  | 9737 | -0.345 | -0.1207 | No |
| 157 | RTN3 |  |  | 9792 | -0.413 | -0.1014 | No |
| 158 | SPARCL1 |  |  | 9798 | -0.423 | -0.0765 | No |
| 159 | LAMA4 |  |  | 9800 | -0.425 | -0.0510 | No |
| 160 | LPL |  |  | 9802 | -0.428 | -0.0254 | No |
| 161 | COL4A1 |  |  | 9834 | -0.552 | 0.0046 | No |
Table: GSEA details [plain text format]

  

Fig 2: HALLMARK\_ADIPOGENESIS: Random ES distribution      
 Gene set null distribution of ES for **HALLMARK\_ADIPOGENESIS**

  
