# Supplementary material for: Modeling glioblastoma heterogeneity as a dynamic network of cell states
Source: Mol Syst Biol. 2021 Sep 16;17(9):e10105. doi: 10.15252/msb.202010105 (PMC8444284; doi:10.15252/msb.202010105)
Supplement: Supplementary file 5 — Source Data for Figure 3 [file MSB-17-e10105-s001.zip › Figure3A_sourcedata/GSEA_3065/hallmarks_state1.GseaPreranked.1623416262439/HALLMARK_ANDROGEN_RESPONSE.html]

Details for gene set HALLMARK\_ANDROGEN\_RESPONSE[GSEA]

|  || Dataset | state1 |
| Phenotype | NoPhenotypeAvailable |
| Upregulated in class | na\_pos |
| GeneSet | HALLMARK\_ANDROGEN\_RESPONSE |
| Enrichment Score (ES) | 0.39236373 |
| Normalized Enrichment Score (NES) | 1.353279 |
| Nominal p-value | 0.05882353 |
| FDR q-value | 0.1111071 |
| FWER p-Value | 0.658 |
Table: GSEA Results Summary

  

Fig 1: Enrichment plot: HALLMARK\_ANDROGEN\_RESPONSE      
 Profile of the Running ES Score & Positions of GeneSet Members on the Rank Ordered List

  

| PROBE | GENE SYMBOL | GENE\_TITLE | RANK IN GENE LIST | RANK METRIC SCORE | RUNNING ES | CORE ENRICHMENT || 1 | PMEPA1 |  |  | 12 | 0.687 | 0.0615 | Yes |
| 2 | SMS |  |  | 23 | 0.619 | 0.1170 | Yes |
| 3 | CCND1 |  |  | 29 | 0.566 | 0.1682 | Yes |
| 4 | DBI |  |  | 110 | 0.352 | 0.1922 | Yes |
| 5 | ADRM1 |  |  | 160 | 0.317 | 0.2161 | Yes |
| 6 | HOMER2 |  |  | 209 | 0.291 | 0.2378 | Yes |
| 7 | ACTN1 |  |  | 230 | 0.284 | 0.2617 | Yes |
| 8 | UBE2J1 |  |  | 467 | 0.212 | 0.2570 | Yes |
| 9 | MYL12A |  |  | 495 | 0.207 | 0.2731 | Yes |
| 10 | AKT1 |  |  | 567 | 0.191 | 0.2833 | Yes |
| 11 | AKAP12 |  |  | 605 | 0.185 | 0.2964 | Yes |
| 12 | VAPA |  |  | 731 | 0.167 | 0.2989 | Yes |
| 13 | CENPN |  |  | 764 | 0.163 | 0.3106 | Yes |
| 14 | NDRG1 |  |  | 765 | 0.163 | 0.3254 | Yes |
| 15 | PA2G4 |  |  | 780 | 0.160 | 0.3386 | Yes |
| 16 | B4GALT1 |  |  | 802 | 0.157 | 0.3508 | Yes |
| 17 | UBE2I |  |  | 834 | 0.153 | 0.3616 | Yes |
| 18 | TSC22D1 |  |  | 881 | 0.147 | 0.3703 | Yes |
| 19 | IQGAP2 |  |  | 987 | 0.135 | 0.3719 | Yes |
| 20 | CDK6 |  |  | 999 | 0.134 | 0.3830 | Yes |
| 21 | XRCC6 |  |  | 1025 | 0.131 | 0.3924 | Yes |
| 22 | CCND3 |  |  | 1389 | 0.096 | 0.3641 | No |
| 23 | PDLIM5 |  |  | 1528 | 0.087 | 0.3580 | No |
| 24 | UAP1 |  |  | 1780 | 0.071 | 0.3389 | No |
| 25 | SRP19 |  |  | 2001 | 0.060 | 0.3219 | No |
| 26 | TPD52 |  |  | 2098 | 0.055 | 0.3171 | No |
| 27 | RPS6KA3 |  |  | 2353 | 0.045 | 0.2953 | No |
| 28 | ELL2 |  |  | 2543 | 0.038 | 0.2795 | No |
| 29 | INPP4B |  |  | 2636 | 0.035 | 0.2733 | No |
| 30 | ARID5B |  |  | 2743 | 0.032 | 0.2654 | No |
| 31 | ZMIZ1 |  |  | 2887 | 0.028 | 0.2534 | No |
| 32 | ANKH |  |  | 3076 | 0.023 | 0.2363 | No |
| 33 | GSR |  |  | 3274 | 0.019 | 0.2179 | No |
| 34 | SORD |  |  | 3535 | 0.013 | 0.1926 | No |
| 35 | INSIG1 |  |  | 3553 | 0.013 | 0.1920 | No |
| 36 | CAMKK2 |  |  | 3594 | 0.012 | 0.1890 | No |
| 37 | STK39 |  |  | 3877 | 0.007 | 0.1609 | No |
| 38 | TNFAIP8 |  |  | 3901 | 0.006 | 0.1591 | No |
| 39 | SRF |  |  | 3906 | 0.006 | 0.1592 | No |
| 40 | SEC24D |  |  | 4162 | 0.001 | 0.1333 | No |
| 41 | RAB4A |  |  | 4359 | -0.002 | 0.1135 | No |
| 42 | NCOA4 |  |  | 4384 | -0.002 | 0.1113 | No |
| 43 | NGLY1 |  |  | 4989 | -0.012 | 0.0509 | No |
| 44 | ELK4 |  |  | 5127 | -0.014 | 0.0382 | No |
| 45 | ABCC4 |  |  | 5260 | -0.016 | 0.0262 | No |
| 46 | RRP12 |  |  | 5826 | -0.026 | -0.0290 | No |
| 47 | DHCR24 |  |  | 5843 | -0.026 | -0.0283 | No |
| 48 | PGM3 |  |  | 5937 | -0.028 | -0.0352 | No |
| 49 | IDI1 |  |  | 6154 | -0.032 | -0.0543 | No |
| 50 | FKBP5 |  |  | 6519 | -0.039 | -0.0879 | No |
| 51 | XRCC5 |  |  | 6701 | -0.043 | -0.1024 | No |
| 52 | PIAS1 |  |  | 6780 | -0.045 | -0.1063 | No |
| 53 | AZGP1 |  |  | 7142 | -0.054 | -0.1382 | No |
| 54 | DNAJB9 |  |  | 7191 | -0.055 | -0.1380 | No |
| 55 | GNAI3 |  |  | 7684 | -0.069 | -0.1819 | No |
| 56 | PLPP1 |  |  | 7870 | -0.076 | -0.1938 | No |
| 57 | ELOVL5 |  |  | 8115 | -0.086 | -0.2108 | No |
| 58 | CDC14B |  |  | 8123 | -0.086 | -0.2037 | No |
| 59 | ZBTB10 |  |  | 8173 | -0.089 | -0.2006 | No |
| 60 | APPBP2 |  |  | 8442 | -0.101 | -0.2186 | No |
| 61 | SLC38A2 |  |  | 8536 | -0.107 | -0.2183 | No |
| 62 | FADS1 |  |  | 9044 | -0.147 | -0.2567 | No |
| 63 | LMAN1 |  |  | 9094 | -0.151 | -0.2478 | No |
| 64 | LIFR |  |  | 9216 | -0.168 | -0.2448 | No |
| 65 | SLC26A2 |  |  | 9331 | -0.186 | -0.2394 | No |
| 66 | TMEM50A |  |  | 9336 | -0.187 | -0.2228 | No |
| 67 | ACSL3 |  |  | 9338 | -0.187 | -0.2058 | No |
| 68 | SPCS3 |  |  | 9609 | -0.263 | -0.2093 | No |
| 69 | HMGCR |  |  | 9640 | -0.277 | -0.1871 | No |
| 70 | ITGAV |  |  | 9650 | -0.282 | -0.1623 | No |
| 71 | HMGCS1 |  |  | 9718 | -0.329 | -0.1391 | No |
| 72 | SCD |  |  | 9762 | -0.370 | -0.1098 | No |
| 73 | B2M |  |  | 9791 | -0.408 | -0.0753 | No |
| 74 | SAT1 |  |  | 9807 | -0.445 | -0.0362 | No |
| 75 | ABHD2 |  |  | 9812 | -0.476 | 0.0068 | No |
Table: GSEA details [plain text format]

  

Fig 2: HALLMARK\_ANDROGEN\_RESPONSE: Random ES distribution      
 Gene set null distribution of ES for **HALLMARK\_ANDROGEN\_RESPONSE**

  
