# Supplementary material for: Modeling glioblastoma heterogeneity as a dynamic network of cell states
Source: Mol Syst Biol. 2021 Sep 16;17(9):e10105. doi: 10.15252/msb.202010105 (PMC8444284; doi:10.15252/msb.202010105)
Supplement: Supplementary file 5 — Source Data for Figure 3 [file MSB-17-e10105-s001.zip › Figure3A_sourcedata/GSEA_3065/hallmarks_state1.GseaPreranked.1623416262439/HALLMARK_ANGIOGENESIS.html]

Details for gene set HALLMARK\_ANGIOGENESIS[GSEA]

|  || Dataset | state1 |
| Phenotype | NoPhenotypeAvailable |
| Upregulated in class | na\_neg |
| GeneSet | HALLMARK\_ANGIOGENESIS |
| Enrichment Score (ES) | -0.5414474 |
| Normalized Enrichment Score (NES) | -1.4158024 |
| Nominal p-value | 0.07113821 |
| FDR q-value | 0.44582027 |
| FWER p-Value | 0.546 |
Table: GSEA Results Summary

  

Fig 1: Enrichment plot: HALLMARK\_ANGIOGENESIS      
 Profile of the Running ES Score & Positions of GeneSet Members on the Rank Ordered List

  

| PROBE | GENE SYMBOL | GENE\_TITLE | RANK IN GENE LIST | RANK METRIC SCORE | RUNNING ES | CORE ENRICHMENT || 1 | POSTN |  |  | 11 | 0.689 | 0.1360 | No |
| 2 | SPP1 |  |  | 17 | 0.642 | 0.2632 | No |
| 3 | TNFRSF21 |  |  | 1222 | 0.110 | 0.1630 | No |
| 4 | NRP1 |  |  | 1691 | 0.076 | 0.1307 | No |
| 5 | S100A4 |  |  | 2114 | 0.054 | 0.0987 | No |
| 6 | TIMP1 |  |  | 2497 | 0.040 | 0.0678 | No |
| 7 | MSX1 |  |  | 3279 | 0.019 | -0.0077 | No |
| 8 | VAV2 |  |  | 6116 | -0.031 | -0.2892 | No |
| 9 | PTK2 |  |  | 7170 | -0.055 | -0.3851 | No |
| 10 | JAG1 |  |  | 7426 | -0.061 | -0.3988 | No |
| 11 | STC1 |  |  | 7777 | -0.072 | -0.4199 | No |
| 12 | FSTL1 |  |  | 8976 | -0.141 | -0.5134 | Yes |
| 13 | VCAN |  |  | 9151 | -0.158 | -0.4995 | Yes |
| 14 | FGFR1 |  |  | 9157 | -0.159 | -0.4683 | Yes |
| 15 | VEGFA |  |  | 9220 | -0.168 | -0.4411 | Yes |
| 16 | PDGFA |  |  | 9341 | -0.188 | -0.4159 | Yes |
| 17 | COL5A2 |  |  | 9551 | -0.240 | -0.3894 | Yes |
| 18 | ITGAV |  |  | 9650 | -0.282 | -0.3432 | Yes |
| 19 | LRPAP1 |  |  | 9691 | -0.305 | -0.2865 | Yes |
| 20 | LPL |  |  | 9802 | -0.428 | -0.2125 | Yes |
| 21 | APP |  |  | 9819 | -0.495 | -0.1155 | Yes |
| 22 | CCND2 |  |  | 9842 | -0.611 | 0.0038 | Yes |
Table: GSEA details [plain text format]

  

Fig 2: HALLMARK\_ANGIOGENESIS: Random ES distribution      
 Gene set null distribution of ES for **HALLMARK\_ANGIOGENESIS**

  
