# Supplementary material for: Modeling glioblastoma heterogeneity as a dynamic network of cell states
Source: Mol Syst Biol. 2021 Sep 16;17(9):e10105. doi: 10.15252/msb.202010105 (PMC8444284; doi:10.15252/msb.202010105)
Supplement: Supplementary file 5 — Source Data for Figure 3 [file MSB-17-e10105-s001.zip › Figure3A_sourcedata/GSEA_3065/hallmarks_state1.GseaPreranked.1623416262439/HALLMARK_APOPTOSIS.html]

Details for gene set HALLMARK\_APOPTOSIS[GSEA]

|  || Dataset | state1 |
| Phenotype | NoPhenotypeAvailable |
| Upregulated in class | na\_pos |
| GeneSet | HALLMARK\_APOPTOSIS |
| Enrichment Score (ES) | 0.35292226 |
| Normalized Enrichment Score (NES) | 1.2933227 |
| Nominal p-value | 0.06798246 |
| FDR q-value | 0.16936128 |
| FWER p-Value | 0.826 |
Table: GSEA Results Summary

  

Fig 1: Enrichment plot: HALLMARK\_APOPTOSIS      
 Profile of the Running ES Score & Positions of GeneSet Members on the Rank Ordered List

  

| PROBE | GENE SYMBOL | GENE\_TITLE | RANK IN GENE LIST | RANK METRIC SCORE | RUNNING ES | CORE ENRICHMENT || 1 | LMNA |  |  | 9 | 0.701 | 0.0433 | Yes |
| 2 | CCND1 |  |  | 29 | 0.566 | 0.0770 | Yes |
| 3 | ANXA1 |  |  | 30 | 0.564 | 0.1126 | Yes |
| 4 | CAV1 |  |  | 34 | 0.536 | 0.1461 | Yes |
| 5 | PMAIP1 |  |  | 93 | 0.380 | 0.1642 | Yes |
| 6 | TNFRSF12A |  |  | 104 | 0.364 | 0.1861 | Yes |
| 7 | LGALS3 |  |  | 117 | 0.348 | 0.2069 | Yes |
| 8 | SOD1 |  |  | 186 | 0.303 | 0.2190 | Yes |
| 9 | GSN |  |  | 213 | 0.290 | 0.2347 | Yes |
| 10 | GPX4 |  |  | 234 | 0.283 | 0.2505 | Yes |
| 11 | CDKN1A |  |  | 262 | 0.274 | 0.2650 | Yes |
| 12 | JUN |  |  | 387 | 0.229 | 0.2668 | Yes |
| 13 | EMP1 |  |  | 473 | 0.211 | 0.2714 | Yes |
| 14 | BID |  |  | 527 | 0.200 | 0.2786 | Yes |
| 15 | HSPB1 |  |  | 531 | 0.199 | 0.2908 | Yes |
| 16 | TSPO |  |  | 648 | 0.179 | 0.2902 | Yes |
| 17 | SQSTM1 |  |  | 701 | 0.172 | 0.2958 | Yes |
| 18 | BCL2L1 |  |  | 755 | 0.165 | 0.3008 | Yes |
| 19 | BMP2 |  |  | 760 | 0.164 | 0.3107 | Yes |
| 20 | HMOX1 |  |  | 784 | 0.160 | 0.3184 | Yes |
| 21 | DAP |  |  | 823 | 0.154 | 0.3242 | Yes |
| 22 | EBP |  |  | 829 | 0.154 | 0.3334 | Yes |
| 23 | CASP3 |  |  | 923 | 0.142 | 0.3329 | Yes |
| 24 | SLC20A1 |  |  | 1019 | 0.132 | 0.3314 | Yes |
| 25 | BAX |  |  | 1029 | 0.130 | 0.3388 | Yes |
| 26 | VDAC2 |  |  | 1080 | 0.124 | 0.3415 | Yes |
| 27 | GADD45A |  |  | 1082 | 0.124 | 0.3492 | Yes |
| 28 | ETF1 |  |  | 1121 | 0.120 | 0.3529 | Yes |
| 29 | CASP7 |  |  | 1205 | 0.112 | 0.3515 | No |
| 30 | DNAJA1 |  |  | 1324 | 0.101 | 0.3457 | No |
| 31 | ISG20 |  |  | 1538 | 0.087 | 0.3294 | No |
| 32 | CASP6 |  |  | 1564 | 0.085 | 0.3322 | No |
| 33 | IRF1 |  |  | 1569 | 0.085 | 0.3372 | No |
| 34 | HMGB2 |  |  | 1570 | 0.085 | 0.3425 | No |
| 35 | PLPPR4 |  |  | 1802 | 0.069 | 0.3232 | No |
| 36 | LEF1 |  |  | 1861 | 0.067 | 0.3215 | No |
| 37 | RHOB |  |  | 1914 | 0.064 | 0.3203 | No |
| 38 | DDIT3 |  |  | 2008 | 0.059 | 0.3145 | No |
| 39 | CASP4 |  |  | 2054 | 0.057 | 0.3135 | No |
| 40 | CD44 |  |  | 2193 | 0.051 | 0.3026 | No |
| 41 | GADD45B |  |  | 2252 | 0.049 | 0.2997 | No |
| 42 | BCL10 |  |  | 2364 | 0.044 | 0.2911 | No |
| 43 | CDC25B |  |  | 2370 | 0.044 | 0.2934 | No |
| 44 | TIMP1 |  |  | 2497 | 0.040 | 0.2830 | No |
| 45 | BTG3 |  |  | 2514 | 0.039 | 0.2838 | No |
| 46 | NEDD9 |  |  | 2528 | 0.039 | 0.2849 | No |
| 47 | FDXR |  |  | 2642 | 0.035 | 0.2756 | No |
| 48 | CASP8 |  |  | 2688 | 0.033 | 0.2730 | No |
| 49 | ATF3 |  |  | 2855 | 0.029 | 0.2579 | No |
| 50 | CFLAR |  |  | 2958 | 0.026 | 0.2491 | No |
| 51 | CASP9 |  |  | 2979 | 0.025 | 0.2486 | No |
| 52 | DAP3 |  |  | 3034 | 0.024 | 0.2446 | No |
| 53 | ANKH |  |  | 3076 | 0.023 | 0.2419 | No |
| 54 | ENO2 |  |  | 3161 | 0.021 | 0.2346 | No |
| 55 | GSR |  |  | 3274 | 0.019 | 0.2243 | No |
| 56 | PAK1 |  |  | 3411 | 0.016 | 0.2114 | No |
| 57 | SOD2 |  |  | 3646 | 0.011 | 0.1881 | No |
| 58 | CYLD |  |  | 3714 | 0.010 | 0.1818 | No |
| 59 | RARA |  |  | 3735 | 0.009 | 0.1804 | No |
| 60 | FAS |  |  | 3812 | 0.008 | 0.1731 | No |
| 61 | DFFA |  |  | 4177 | 0.001 | 0.1359 | No |
| 62 | PPP2R5B |  |  | 4271 | -0.001 | 0.1264 | No |
| 63 | WEE1 |  |  | 4385 | -0.003 | 0.1150 | No |
| 64 | RELA |  |  | 4420 | -0.003 | 0.1117 | No |
| 65 | CDKN1B |  |  | 4655 | -0.007 | 0.0881 | No |
| 66 | MADD |  |  | 4895 | -0.011 | 0.0644 | No |
| 67 | PSEN2 |  |  | 5019 | -0.013 | 0.0526 | No |
| 68 | PDGFRB |  |  | 5041 | -0.013 | 0.0513 | No |
| 69 | TAP1 |  |  | 5226 | -0.016 | 0.0334 | No |
| 70 | RNASEL |  |  | 5284 | -0.017 | 0.0286 | No |
| 71 | BCL2L2 |  |  | 5383 | -0.018 | 0.0197 | No |
| 72 | DPYD |  |  | 5403 | -0.018 | 0.0189 | No |
| 73 | PPP3R1 |  |  | 5493 | -0.020 | 0.0111 | No |
| 74 | BCL2L11 |  |  | 5827 | -0.026 | -0.0214 | No |
| 75 | CDK2 |  |  | 5964 | -0.029 | -0.0335 | No |
| 76 | SATB1 |  |  | 6070 | -0.030 | -0.0424 | No |
| 77 | DNM1L |  |  | 6273 | -0.034 | -0.0609 | No |
| 78 | PEA15 |  |  | 6830 | -0.046 | -0.1150 | No |
| 79 | RETSAT |  |  | 6959 | -0.049 | -0.1250 | No |
| 80 | ROCK1 |  |  | 7045 | -0.051 | -0.1304 | No |
| 81 | MMP2 |  |  | 7051 | -0.051 | -0.1277 | No |
| 82 | TIMP3 |  |  | 7100 | -0.053 | -0.1293 | No |
| 83 | PTK2 |  |  | 7170 | -0.055 | -0.1329 | No |
| 84 | PDCD4 |  |  | 7452 | -0.062 | -0.1578 | No |
| 85 | TOP2A |  |  | 7678 | -0.069 | -0.1765 | No |
| 86 | ADD1 |  |  | 7750 | -0.072 | -0.1792 | No |
| 87 | CREBBP |  |  | 7776 | -0.072 | -0.1772 | No |
| 88 | XIAP |  |  | 7867 | -0.076 | -0.1817 | No |
| 89 | CASP2 |  |  | 7880 | -0.077 | -0.1781 | No |
| 90 | MCL1 |  |  | 8011 | -0.082 | -0.1862 | No |
| 91 | SC5D |  |  | 8206 | -0.090 | -0.2004 | No |
| 92 | RHOT2 |  |  | 8215 | -0.090 | -0.1956 | No |
| 93 | BCAP31 |  |  | 8279 | -0.093 | -0.1961 | No |
| 94 | PSEN1 |  |  | 8386 | -0.098 | -0.2008 | No |
| 95 | ERBB2 |  |  | 8409 | -0.100 | -0.1967 | No |
| 96 | TGFB2 |  |  | 8497 | -0.105 | -0.1990 | No |
| 97 | IGF2R |  |  | 8525 | -0.107 | -0.1951 | No |
| 98 | IFNGR1 |  |  | 8689 | -0.116 | -0.2044 | No |
| 99 | BNIP3L |  |  | 8707 | -0.118 | -0.1987 | No |
| 100 | FEZ1 |  |  | 8932 | -0.137 | -0.2130 | No |
| 101 | F2R |  |  | 8950 | -0.138 | -0.2060 | No |
| 102 | SPTAN1 |  |  | 9199 | -0.165 | -0.2210 | No |
| 103 | DNAJC3 |  |  | 9282 | -0.177 | -0.2182 | No |
| 104 | CTNNB1 |  |  | 9294 | -0.179 | -0.2080 | No |
| 105 | BTG2 |  |  | 9304 | -0.181 | -0.1975 | No |
| 106 | PPT1 |  |  | 9318 | -0.184 | -0.1873 | No |
| 107 | BRCA1 |  |  | 9600 | -0.258 | -0.1998 | No |
| 108 | ERBB3 |  |  | 9601 | -0.258 | -0.1835 | No |
| 109 | TIMP2 |  |  | 9634 | -0.274 | -0.1695 | No |
| 110 | TXNIP |  |  | 9639 | -0.276 | -0.1524 | No |
| 111 | IFITM3 |  |  | 9643 | -0.277 | -0.1353 | No |
| 112 | CLU |  |  | 9795 | -0.418 | -0.1243 | No |
| 113 | SAT1 |  |  | 9807 | -0.445 | -0.0974 | No |
| 114 | APP |  |  | 9819 | -0.495 | -0.0673 | No |
| 115 | PLAT |  |  | 9833 | -0.550 | -0.0339 | No |
| 116 | CCND2 |  |  | 9842 | -0.611 | 0.0038 | No |
Table: GSEA details [plain text format]

  

Fig 2: HALLMARK\_APOPTOSIS: Random ES distribution      
 Gene set null distribution of ES for **HALLMARK\_APOPTOSIS**

  
