# Supplementary material for: Modeling glioblastoma heterogeneity as a dynamic network of cell states
Source: Mol Syst Biol. 2021 Sep 16;17(9):e10105. doi: 10.15252/msb.202010105 (PMC8444284; doi:10.15252/msb.202010105)
Supplement: Supplementary file 5 — Source Data for Figure 3 [file MSB-17-e10105-s001.zip › Figure3A_sourcedata/GSEA_3065/hallmarks_state1.GseaPreranked.1623416262439/HALLMARK_BILE_ACID_METABOLISM.html]

Details for gene set HALLMARK\_BILE\_ACID\_METABOLISM[GSEA]

|  || Dataset | state1 |
| Phenotype | NoPhenotypeAvailable |
| Upregulated in class | na\_neg |
| GeneSet | HALLMARK\_BILE\_ACID\_METABOLISM |
| Enrichment Score (ES) | -0.3199678 |
| Normalized Enrichment Score (NES) | -1.0392342 |
| Nominal p-value | 0.38404453 |
| FDR q-value | 0.619465 |
| FWER p-Value | 1.0 |
Table: GSEA Results Summary

  

Fig 1: Enrichment plot: HALLMARK\_BILE\_ACID\_METABOLISM      
 Profile of the Running ES Score & Positions of GeneSet Members on the Rank Ordered List

  

| PROBE | GENE SYMBOL | GENE\_TITLE | RANK IN GENE LIST | RANK METRIC SCORE | RUNNING ES | CORE ENRICHMENT || 1 | SOD1 |  |  | 186 | 0.303 | 0.0476 | No |
| 2 | PRDX5 |  |  | 640 | 0.180 | 0.0410 | No |
| 3 | OPTN |  |  | 822 | 0.154 | 0.0565 | No |
| 4 | ACSL1 |  |  | 962 | 0.138 | 0.0728 | No |
| 5 | RBP1 |  |  | 965 | 0.138 | 0.1028 | No |
| 6 | GCLM |  |  | 1238 | 0.109 | 0.0991 | No |
| 7 | PXMP2 |  |  | 1573 | 0.085 | 0.0837 | No |
| 8 | BCAR3 |  |  | 1854 | 0.067 | 0.0699 | No |
| 9 | NEDD4 |  |  | 2495 | 0.040 | 0.0135 | No |
| 10 | FDXR |  |  | 2642 | 0.035 | 0.0062 | No |
| 11 | SCP2 |  |  | 3047 | 0.024 | -0.0297 | No |
| 12 | PECR |  |  | 3184 | 0.021 | -0.0390 | No |
| 13 | ISOC1 |  |  | 3358 | 0.017 | -0.0529 | No |
| 14 | PEX19 |  |  | 3465 | 0.015 | -0.0605 | No |
| 15 | PEX6 |  |  | 4041 | 0.003 | -0.1183 | No |
| 16 | ABCD1 |  |  | 4131 | 0.002 | -0.1269 | No |
| 17 | CYP27A1 |  |  | 4336 | -0.002 | -0.1474 | No |
| 18 | SLC27A5 |  |  | 4568 | -0.006 | -0.1696 | No |
| 19 | HACL1 |  |  | 4765 | -0.008 | -0.1877 | No |
| 20 | CAT |  |  | 4951 | -0.012 | -0.2040 | No |
| 21 | PHYH |  |  | 5120 | -0.014 | -0.2180 | No |
| 22 | ALDH9A1 |  |  | 5173 | -0.015 | -0.2200 | No |
| 23 | SLC29A1 |  |  | 5199 | -0.015 | -0.2191 | No |
| 24 | SLC35B2 |  |  | 5276 | -0.016 | -0.2232 | No |
| 25 | PEX16 |  |  | 5564 | -0.021 | -0.2478 | No |
| 26 | DHCR24 |  |  | 5843 | -0.026 | -0.2703 | No |
| 27 | ABCD3 |  |  | 6145 | -0.032 | -0.2940 | No |
| 28 | IDI1 |  |  | 6154 | -0.032 | -0.2878 | No |
| 29 | PEX13 |  |  | 6354 | -0.036 | -0.3002 | No |
| 30 | NPC1 |  |  | 6363 | -0.036 | -0.2932 | No |
| 31 | ATXN1 |  |  | 6560 | -0.040 | -0.3045 | No |
| 32 | CROT |  |  | 6586 | -0.040 | -0.2981 | No |
| 33 | SLC22A18 |  |  | 6599 | -0.041 | -0.2905 | No |
| 34 | NUDT12 |  |  | 6603 | -0.041 | -0.2818 | No |
| 35 | GNPAT |  |  | 6663 | -0.042 | -0.2786 | No |
| 36 | RETSAT |  |  | 6959 | -0.049 | -0.2979 | No |
| 37 | RXRA |  |  | 7177 | -0.055 | -0.3079 | Yes |
| 38 | LONP2 |  |  | 7238 | -0.056 | -0.3017 | Yes |
| 39 | ABCA2 |  |  | 7374 | -0.060 | -0.3023 | Yes |
| 40 | PFKM |  |  | 7421 | -0.061 | -0.2936 | Yes |
| 41 | ABCA3 |  |  | 7467 | -0.062 | -0.2845 | Yes |
| 42 | IDH2 |  |  | 7549 | -0.065 | -0.2785 | Yes |
| 43 | PEX26 |  |  | 7569 | -0.066 | -0.2660 | Yes |
| 44 | MLYCD |  |  | 8016 | -0.082 | -0.2935 | Yes |
| 45 | CYP46A1 |  |  | 8073 | -0.084 | -0.2807 | Yes |
| 46 | SLC23A2 |  |  | 8297 | -0.094 | -0.2827 | Yes |
| 47 | GSTK1 |  |  | 8422 | -0.100 | -0.2733 | Yes |
| 48 | PNPLA8 |  |  | 8435 | -0.101 | -0.2524 | Yes |
| 49 | ABCA1 |  |  | 8590 | -0.111 | -0.2437 | Yes |
| 50 | PEX1 |  |  | 8591 | -0.111 | -0.2194 | Yes |
| 51 | HSD17B11 |  |  | 8769 | -0.123 | -0.2104 | Yes |
| 52 | FADS1 |  |  | 9044 | -0.147 | -0.2061 | Yes |
| 53 | ABCA5 |  |  | 9062 | -0.148 | -0.1753 | Yes |
| 54 | AR |  |  | 9065 | -0.148 | -0.1429 | Yes |
| 55 | FADS2 |  |  | 9078 | -0.150 | -0.1112 | Yes |
| 56 | HSD17B4 |  |  | 9277 | -0.176 | -0.0926 | Yes |
| 57 | IDH1 |  |  | 9737 | -0.345 | -0.0636 | Yes |
| 58 | EFHC1 |  |  | 9748 | -0.355 | 0.0133 | Yes |
Table: GSEA details [plain text format]

  

Fig 2: HALLMARK\_BILE\_ACID\_METABOLISM: Random ES distribution      
 Gene set null distribution of ES for **HALLMARK\_BILE\_ACID\_METABOLISM**

  
