# Supplementary material for: Modeling glioblastoma heterogeneity as a dynamic network of cell states
Source: Mol Syst Biol. 2021 Sep 16;17(9):e10105. doi: 10.15252/msb.202010105 (PMC8444284; doi:10.15252/msb.202010105)
Supplement: Supplementary file 5 — Source Data for Figure 3 [file MSB-17-e10105-s001.zip › Figure3A_sourcedata/GSEA_3065/hallmarks_state1.GseaPreranked.1623416262439/HALLMARK_CHOLESTEROL_HOMEOSTASIS.html]

Details for gene set HALLMARK\_CHOLESTEROL\_HOMEOSTASIS[GSEA]

|  || Dataset | state1 |
| Phenotype | NoPhenotypeAvailable |
| Upregulated in class | na\_neg |
| GeneSet | HALLMARK\_CHOLESTEROL\_HOMEOSTASIS |
| Enrichment Score (ES) | -0.39201775 |
| Normalized Enrichment Score (NES) | -1.279854 |
| Nominal p-value | 0.10093458 |
| FDR q-value | 0.42452675 |
| FWER p-Value | 0.896 |
Table: GSEA Results Summary

  

Fig 1: Enrichment plot: HALLMARK\_CHOLESTEROL\_HOMEOSTASIS      
 Profile of the Running ES Score & Positions of GeneSet Members on the Rank Ordered List

  

| PROBE | GENE SYMBOL | GENE\_TITLE | RANK IN GENE LIST | RANK METRIC SCORE | RUNNING ES | CORE ENRICHMENT || 1 | S100A11 |  |  | 51 | 0.475 | 0.0494 | No |
| 2 | ACTG1 |  |  | 94 | 0.380 | 0.0888 | No |
| 3 | TNFRSF12A |  |  | 104 | 0.364 | 0.1298 | No |
| 4 | LGALS3 |  |  | 117 | 0.348 | 0.1685 | No |
| 5 | ACAT2 |  |  | 385 | 0.229 | 0.1677 | No |
| 6 | FDFT1 |  |  | 635 | 0.181 | 0.1631 | No |
| 7 | ANXA5 |  |  | 709 | 0.171 | 0.1753 | No |
| 8 | EBP |  |  | 829 | 0.154 | 0.1809 | No |
| 9 | PCYT2 |  |  | 869 | 0.148 | 0.1940 | No |
| 10 | ETHE1 |  |  | 1064 | 0.126 | 0.1887 | No |
| 11 | ATF5 |  |  | 1326 | 0.101 | 0.1737 | No |
| 12 | ERRFI1 |  |  | 1423 | 0.094 | 0.1747 | No |
| 13 | PLAUR |  |  | 1463 | 0.091 | 0.1813 | No |
| 14 | FABP5 |  |  | 1470 | 0.091 | 0.1911 | No |
| 15 | DHCR7 |  |  | 1553 | 0.086 | 0.1926 | No |
| 16 | GNAI1 |  |  | 2013 | 0.059 | 0.1527 | No |
| 17 | MVD |  |  | 2220 | 0.050 | 0.1375 | No |
| 18 | PMVK |  |  | 2246 | 0.049 | 0.1406 | No |
| 19 | ECH1 |  |  | 2355 | 0.045 | 0.1348 | No |
| 20 | PLSCR1 |  |  | 2379 | 0.044 | 0.1375 | No |
| 21 | STX5 |  |  | 2693 | 0.033 | 0.1094 | No |
| 22 | ATF3 |  |  | 2855 | 0.029 | 0.0963 | No |
| 23 | MVK |  |  | 2873 | 0.028 | 0.0978 | No |
| 24 | ACSS2 |  |  | 3588 | 0.012 | 0.0265 | No |
| 25 | CHKA |  |  | 3687 | 0.010 | 0.0176 | No |
| 26 | LSS |  |  | 3836 | 0.007 | 0.0034 | No |
| 27 | GPX8 |  |  | 3993 | 0.004 | -0.0120 | No |
| 28 | NFIL3 |  |  | 4056 | 0.003 | -0.0179 | No |
| 29 | FDPS |  |  | 4450 | -0.004 | -0.0575 | No |
| 30 | GLDC |  |  | 4543 | -0.005 | -0.0663 | No |
| 31 | STARD4 |  |  | 4548 | -0.005 | -0.0661 | No |
| 32 | LDLR |  |  | 4731 | -0.008 | -0.0837 | No |
| 33 | CXCL16 |  |  | 4892 | -0.011 | -0.0987 | No |
| 34 | NSDHL |  |  | 5391 | -0.018 | -0.1474 | No |
| 35 | PDK3 |  |  | 5462 | -0.020 | -0.1523 | No |
| 36 | HSD17B7 |  |  | 5466 | -0.020 | -0.1503 | No |
| 37 | IDI1 |  |  | 6154 | -0.032 | -0.2166 | No |
| 38 | CPEB2 |  |  | 6458 | -0.038 | -0.2432 | No |
| 39 | ANTXR2 |  |  | 6986 | -0.050 | -0.2911 | No |
| 40 | CYP51A1 |  |  | 7249 | -0.057 | -0.3113 | No |
| 41 | ABCA2 |  |  | 7374 | -0.060 | -0.3171 | No |
| 42 | JAG1 |  |  | 7426 | -0.061 | -0.3152 | No |
| 43 | TP53INP1 |  |  | 7767 | -0.072 | -0.3416 | No |
| 44 | PNRC1 |  |  | 8006 | -0.081 | -0.3565 | No |
| 45 | TMEM97 |  |  | 8161 | -0.088 | -0.3621 | No |
| 46 | SC5D |  |  | 8206 | -0.090 | -0.3563 | No |
| 47 | LGMN |  |  | 8379 | -0.098 | -0.3625 | No |
| 48 | SEMA3B |  |  | 8556 | -0.109 | -0.3680 | No |
| 49 | FASN |  |  | 8694 | -0.117 | -0.3685 | No |
| 50 | ATXN2 |  |  | 8926 | -0.136 | -0.3763 | Yes |
| 51 | SREBF2 |  |  | 8943 | -0.138 | -0.3621 | Yes |
| 52 | FADS2 |  |  | 9078 | -0.150 | -0.3585 | Yes |
| 53 | GUSB |  |  | 9165 | -0.161 | -0.3488 | Yes |
| 54 | CTNNB1 |  |  | 9294 | -0.179 | -0.3412 | Yes |
| 55 | TM7SF2 |  |  | 9450 | -0.213 | -0.3325 | Yes |
| 56 | HMGCR |  |  | 9640 | -0.277 | -0.3200 | Yes |
| 57 | HMGCS1 |  |  | 9718 | -0.329 | -0.2901 | Yes |
| 58 | SCD |  |  | 9762 | -0.370 | -0.2520 | Yes |
| 59 | SQLE |  |  | 9790 | -0.408 | -0.2079 | Yes |
| 60 | CLU |  |  | 9795 | -0.418 | -0.1602 | Yes |
| 61 | LPL |  |  | 9802 | -0.428 | -0.1116 | Yes |
| 62 | ALCAM |  |  | 9818 | -0.493 | -0.0564 | Yes |
| 63 | CD9 |  |  | 9832 | -0.544 | 0.0048 | Yes |
Table: GSEA details [plain text format]

  

Fig 2: HALLMARK\_CHOLESTEROL\_HOMEOSTASIS: Random ES distribution      
 Gene set null distribution of ES for **HALLMARK\_CHOLESTEROL\_HOMEOSTASIS**

  
