# Supplementary material for: Modeling glioblastoma heterogeneity as a dynamic network of cell states
Source: Mol Syst Biol. 2021 Sep 16;17(9):e10105. doi: 10.15252/msb.202010105 (PMC8444284; doi:10.15252/msb.202010105)
Supplement: Supplementary file 5 — Source Data for Figure 3 [file MSB-17-e10105-s001.zip › Figure3A_sourcedata/GSEA_3065/hallmarks_state1.GseaPreranked.1623416262439/HALLMARK_COMPLEMENT.html]

Details for gene set HALLMARK\_COMPLEMENT[GSEA]

|  || Dataset | state1 |
| Phenotype | NoPhenotypeAvailable |
| Upregulated in class | na\_neg |
| GeneSet | HALLMARK\_COMPLEMENT |
| Enrichment Score (ES) | -0.30179477 |
| Normalized Enrichment Score (NES) | -1.0675169 |
| Nominal p-value | 0.3433396 |
| FDR q-value | 0.693186 |
| FWER p-Value | 1.0 |
Table: GSEA Results Summary

  

Fig 1: Enrichment plot: HALLMARK\_COMPLEMENT      
 Profile of the Running ES Score & Positions of GeneSet Members on the Rank Ordered List

  

| PROBE | GENE SYMBOL | GENE\_TITLE | RANK IN GENE LIST | RANK METRIC SCORE | RUNNING ES | CORE ENRICHMENT || 1 | S100A13 |  |  | 20 | 0.627 | 0.0444 | No |
| 2 | PFN1 |  |  | 26 | 0.591 | 0.0876 | No |
| 3 | CTSC |  |  | 43 | 0.498 | 0.1229 | No |
| 4 | CTSV |  |  | 45 | 0.491 | 0.1592 | No |
| 5 | LGALS3 |  |  | 117 | 0.348 | 0.1777 | No |
| 6 | ATOX1 |  |  | 179 | 0.306 | 0.1942 | No |
| 7 | CALM1 |  |  | 446 | 0.217 | 0.1830 | No |
| 8 | CSRP1 |  |  | 504 | 0.205 | 0.1924 | No |
| 9 | PDP1 |  |  | 536 | 0.198 | 0.2039 | No |
| 10 | ANXA5 |  |  | 709 | 0.171 | 0.1990 | No |
| 11 | PSMB9 |  |  | 745 | 0.166 | 0.2077 | No |
| 12 | GNB2 |  |  | 767 | 0.162 | 0.2176 | No |
| 13 | PREP |  |  | 828 | 0.154 | 0.2228 | No |
| 14 | CASP3 |  |  | 923 | 0.142 | 0.2237 | No |
| 15 | DUSP5 |  |  | 1114 | 0.121 | 0.2133 | No |
| 16 | PPP4C |  |  | 1165 | 0.116 | 0.2168 | No |
| 17 | GRB2 |  |  | 1195 | 0.113 | 0.2222 | No |
| 18 | CASP7 |  |  | 1205 | 0.112 | 0.2295 | No |
| 19 | SERPINE1 |  |  | 1250 | 0.108 | 0.2330 | No |
| 20 | FDX1 |  |  | 1274 | 0.106 | 0.2385 | No |
| 21 | IRF2 |  |  | 1299 | 0.103 | 0.2437 | No |
| 22 | DUSP6 |  |  | 1401 | 0.095 | 0.2404 | No |
| 23 | LAP3 |  |  | 1418 | 0.094 | 0.2458 | No |
| 24 | PLAUR |  |  | 1463 | 0.091 | 0.2481 | No |
| 25 | IRF1 |  |  | 1569 | 0.085 | 0.2436 | No |
| 26 | CEBPB |  |  | 1680 | 0.076 | 0.2380 | No |
| 27 | RHOG |  |  | 1768 | 0.071 | 0.2344 | No |
| 28 | EHD1 |  |  | 1846 | 0.067 | 0.2315 | No |
| 29 | CASP4 |  |  | 2054 | 0.057 | 0.2146 | No |
| 30 | LYN |  |  | 2272 | 0.048 | 0.1959 | No |
| 31 | GNAI2 |  |  | 2281 | 0.048 | 0.1987 | No |
| 32 | PPP2CB |  |  | 2344 | 0.045 | 0.1957 | No |
| 33 | PLSCR1 |  |  | 2379 | 0.044 | 0.1955 | No |
| 34 | RABIF |  |  | 2439 | 0.042 | 0.1925 | No |
| 35 | USP14 |  |  | 2445 | 0.041 | 0.1951 | No |
| 36 | MAFF |  |  | 2492 | 0.040 | 0.1933 | No |
| 37 | TIMP1 |  |  | 2497 | 0.040 | 0.1958 | No |
| 38 | MSRB1 |  |  | 2513 | 0.039 | 0.1972 | No |
| 39 | PCLO |  |  | 2539 | 0.038 | 0.1975 | No |
| 40 | PRDM4 |  |  | 2652 | 0.034 | 0.1886 | No |
| 41 | FN1 |  |  | 2705 | 0.033 | 0.1857 | No |
| 42 | SH2B3 |  |  | 2725 | 0.032 | 0.1861 | No |
| 43 | STX4 |  |  | 2752 | 0.031 | 0.1858 | No |
| 44 | CALM3 |  |  | 2882 | 0.028 | 0.1747 | No |
| 45 | PRKCD |  |  | 2926 | 0.027 | 0.1722 | No |
| 46 | CASP9 |  |  | 2979 | 0.025 | 0.1688 | No |
| 47 | USP16 |  |  | 3016 | 0.025 | 0.1669 | No |
| 48 | APOBEC3F |  |  | 3051 | 0.024 | 0.1652 | No |
| 49 | IRF7 |  |  | 3585 | 0.012 | 0.1116 | No |
| 50 | PDGFB |  |  | 3613 | 0.012 | 0.1097 | No |
| 51 | APOBEC3G |  |  | 3647 | 0.011 | 0.1071 | No |
| 52 | XPNPEP1 |  |  | 3846 | 0.007 | 0.0874 | No |
| 53 | CDK5R1 |  |  | 3866 | 0.007 | 0.0859 | No |
| 54 | GMFB |  |  | 4399 | -0.003 | 0.0317 | No |
| 55 | GCA |  |  | 4757 | -0.008 | -0.0042 | No |
| 56 | RNF4 |  |  | 4856 | -0.010 | -0.0135 | No |
| 57 | CTSB |  |  | 4873 | -0.011 | -0.0143 | No |
| 58 | RCE1 |  |  | 5095 | -0.014 | -0.0359 | No |
| 59 | SIRT6 |  |  | 5217 | -0.016 | -0.0471 | No |
| 60 | LTA4H |  |  | 5586 | -0.022 | -0.0832 | No |
| 61 | BRPF3 |  |  | 5639 | -0.023 | -0.0868 | No |
| 62 | KIF2A |  |  | 5712 | -0.024 | -0.0924 | No |
| 63 | PIK3CA |  |  | 5747 | -0.024 | -0.0941 | No |
| 64 | GNG2 |  |  | 6136 | -0.032 | -0.1314 | No |
| 65 | L3MBTL4 |  |  | 6218 | -0.033 | -0.1373 | No |
| 66 | RBSN |  |  | 6265 | -0.034 | -0.1394 | No |
| 67 | USP15 |  |  | 6284 | -0.034 | -0.1387 | No |
| 68 | MMP14 |  |  | 6548 | -0.039 | -0.1627 | No |
| 69 | GPD2 |  |  | 6736 | -0.044 | -0.1786 | No |
| 70 | DYRK2 |  |  | 6791 | -0.045 | -0.1808 | No |
| 71 | CTSO |  |  | 6919 | -0.048 | -0.1902 | No |
| 72 | CBLB |  |  | 7070 | -0.052 | -0.2017 | No |
| 73 | LIPA |  |  | 7165 | -0.055 | -0.2073 | No |
| 74 | VCPIP1 |  |  | 7185 | -0.055 | -0.2052 | No |
| 75 | CPQ |  |  | 7511 | -0.064 | -0.2337 | No |
| 76 | CTSL |  |  | 7676 | -0.069 | -0.2454 | No |
| 77 | GNAI3 |  |  | 7684 | -0.069 | -0.2410 | No |
| 78 | DGKH |  |  | 7702 | -0.070 | -0.2375 | No |
| 79 | USP8 |  |  | 7753 | -0.072 | -0.2373 | No |
| 80 | AKAP10 |  |  | 7856 | -0.075 | -0.2422 | No |
| 81 | TFPI2 |  |  | 8026 | -0.082 | -0.2534 | No |
| 82 | LGMN |  |  | 8379 | -0.098 | -0.2821 | No |
| 83 | PSEN1 |  |  | 8386 | -0.098 | -0.2755 | No |
| 84 | RAF1 |  |  | 8419 | -0.100 | -0.2713 | No |
| 85 | GNB4 |  |  | 8485 | -0.104 | -0.2703 | No |
| 86 | CD46 |  |  | 8491 | -0.105 | -0.2630 | No |
| 87 | CA2 |  |  | 8636 | -0.113 | -0.2694 | No |
| 88 | MT3 |  |  | 8954 | -0.139 | -0.2915 | Yes |
| 89 | ADAM9 |  |  | 8977 | -0.141 | -0.2833 | Yes |
| 90 | MMP15 |  |  | 9003 | -0.143 | -0.2753 | Yes |
| 91 | CD59 |  |  | 9097 | -0.152 | -0.2736 | Yes |
| 92 | CTSD |  |  | 9287 | -0.178 | -0.2798 | Yes |
| 93 | C1R |  |  | 9298 | -0.180 | -0.2674 | Yes |
| 94 | ZEB1 |  |  | 9492 | -0.224 | -0.2706 | Yes |
| 95 | HSPA5 |  |  | 9533 | -0.234 | -0.2574 | Yes |
| 96 | FYN |  |  | 9575 | -0.250 | -0.2431 | Yes |
| 97 | DOCK10 |  |  | 9599 | -0.258 | -0.2263 | Yes |
| 98 | TIMP2 |  |  | 9634 | -0.274 | -0.2095 | Yes |
| 99 | CPM |  |  | 9638 | -0.276 | -0.1894 | Yes |
| 100 | LRP1 |  |  | 9771 | -0.381 | -0.1747 | Yes |
| 101 | COL4A2 |  |  | 9786 | -0.402 | -0.1464 | Yes |
| 102 | CLU |  |  | 9795 | -0.418 | -0.1162 | Yes |
| 103 | LAMP2 |  |  | 9796 | -0.419 | -0.0852 | Yes |
| 104 | PLAT |  |  | 9833 | -0.550 | -0.0481 | Yes |
| 105 | PRCP |  |  | 9855 | -0.712 | 0.0025 | Yes |
Table: GSEA details [plain text format]

  

Fig 2: HALLMARK\_COMPLEMENT: Random ES distribution      
 Gene set null distribution of ES for **HALLMARK\_COMPLEMENT**

  
