# Supplementary material for: Modeling glioblastoma heterogeneity as a dynamic network of cell states
Source: Mol Syst Biol. 2021 Sep 16;17(9):e10105. doi: 10.15252/msb.202010105 (PMC8444284; doi:10.15252/msb.202010105)
Supplement: Supplementary file 5 — Source Data for Figure 3 [file MSB-17-e10105-s001.zip › Figure3A_sourcedata/GSEA_3065/hallmarks_state1.GseaPreranked.1623416262439/HALLMARK_DNA_REPAIR.html]

Details for gene set HALLMARK\_DNA\_REPAIR[GSEA]

|  || Dataset | state1 |
| Phenotype | NoPhenotypeAvailable |
| Upregulated in class | na\_pos |
| GeneSet | HALLMARK\_DNA\_REPAIR |
| Enrichment Score (ES) | 0.42877966 |
| Normalized Enrichment Score (NES) | 1.6133974 |
| Nominal p-value | 0.0022421526 |
| FDR q-value | 0.019398743 |
| FWER p-Value | 0.088 |
Table: GSEA Results Summary

  

Fig 1: Enrichment plot: HALLMARK\_DNA\_REPAIR      
 Profile of the Running ES Score & Positions of GeneSet Members on the Rank Ordered List

  

| PROBE | GENE SYMBOL | GENE\_TITLE | RANK IN GENE LIST | RANK METRIC SCORE | RUNNING ES | CORE ENRICHMENT || 1 | ADRM1 |  |  | 160 | 0.317 | 0.0115 | Yes |
| 2 | GPX4 |  |  | 234 | 0.283 | 0.0289 | Yes |
| 3 | MPC2 |  |  | 266 | 0.273 | 0.0497 | Yes |
| 4 | HPRT1 |  |  | 289 | 0.263 | 0.0705 | Yes |
| 5 | TAF10 |  |  | 338 | 0.246 | 0.0872 | Yes |
| 6 | ALYREF |  |  | 371 | 0.234 | 0.1045 | Yes |
| 7 | GUK1 |  |  | 374 | 0.233 | 0.1248 | Yes |
| 8 | EDF1 |  |  | 380 | 0.231 | 0.1446 | Yes |
| 9 | RBX1 |  |  | 389 | 0.228 | 0.1639 | Yes |
| 10 | NME1 |  |  | 395 | 0.227 | 0.1833 | Yes |
| 11 | POLR2E |  |  | 520 | 0.201 | 0.1883 | Yes |
| 12 | DGUOK |  |  | 555 | 0.194 | 0.2019 | Yes |
| 13 | COX17 |  |  | 565 | 0.191 | 0.2178 | Yes |
| 14 | GTF2A2 |  |  | 581 | 0.189 | 0.2329 | Yes |
| 15 | TAF9 |  |  | 596 | 0.186 | 0.2478 | Yes |
| 16 | GTF2H5 |  |  | 654 | 0.178 | 0.2577 | Yes |
| 17 | APRT |  |  | 711 | 0.171 | 0.2669 | Yes |
| 18 | POLR2J |  |  | 742 | 0.166 | 0.2785 | Yes |
| 19 | MRPL40 |  |  | 838 | 0.153 | 0.2822 | Yes |
| 20 | POLR2I |  |  | 878 | 0.147 | 0.2912 | Yes |
| 21 | POLR2K |  |  | 896 | 0.146 | 0.3022 | Yes |
| 22 | POLE4 |  |  | 910 | 0.143 | 0.3135 | Yes |
| 23 | MPG |  |  | 915 | 0.143 | 0.3257 | Yes |
| 24 | POLR1D |  |  | 928 | 0.142 | 0.3370 | Yes |
| 25 | SAC3D1 |  |  | 976 | 0.137 | 0.3442 | Yes |
| 26 | NME4 |  |  | 992 | 0.134 | 0.3544 | Yes |
| 27 | TSG101 |  |  | 1026 | 0.131 | 0.3625 | Yes |
| 28 | RPA3 |  |  | 1039 | 0.129 | 0.3727 | Yes |
| 29 | SSRP1 |  |  | 1051 | 0.128 | 0.3828 | Yes |
| 30 | IMPDH2 |  |  | 1060 | 0.127 | 0.3932 | Yes |
| 31 | RFC2 |  |  | 1094 | 0.123 | 0.4006 | Yes |
| 32 | RALA |  |  | 1145 | 0.118 | 0.4059 | Yes |
| 33 | ERCC1 |  |  | 1187 | 0.113 | 0.4117 | Yes |
| 34 | POLR2H |  |  | 1200 | 0.112 | 0.4203 | Yes |
| 35 | TAF12 |  |  | 1311 | 0.102 | 0.4180 | Yes |
| 36 | TYMS |  |  | 1501 | 0.089 | 0.4065 | Yes |
| 37 | NELFE |  |  | 1518 | 0.088 | 0.4126 | Yes |
| 38 | SURF1 |  |  | 1565 | 0.085 | 0.4153 | Yes |
| 39 | POLR2C |  |  | 1634 | 0.080 | 0.4154 | Yes |
| 40 | NELFB |  |  | 1669 | 0.077 | 0.4187 | Yes |
| 41 | RAE1 |  |  | 1737 | 0.073 | 0.4183 | Yes |
| 42 | TAF6 |  |  | 1755 | 0.072 | 0.4229 | Yes |
| 43 | SUPT4H1 |  |  | 1760 | 0.072 | 0.4288 | Yes |
| 44 | GTF2F1 |  |  | 1880 | 0.066 | 0.4224 | No |
| 45 | ZWINT |  |  | 1923 | 0.064 | 0.4237 | No |
| 46 | SNAPC5 |  |  | 1942 | 0.063 | 0.4274 | No |
| 47 | POLB |  |  | 2152 | 0.053 | 0.4106 | No |
| 48 | AK3 |  |  | 2163 | 0.052 | 0.4142 | No |
| 49 | RPA2 |  |  | 2385 | 0.044 | 0.3954 | No |
| 50 | FEN1 |  |  | 2420 | 0.043 | 0.3956 | No |
| 51 | GMPR2 |  |  | 2468 | 0.041 | 0.3944 | No |
| 52 | GTF2H1 |  |  | 2503 | 0.040 | 0.3944 | No |
| 53 | ELOA |  |  | 2560 | 0.038 | 0.3919 | No |
| 54 | CSTF3 |  |  | 2662 | 0.034 | 0.3846 | No |
| 55 | CCNO |  |  | 2893 | 0.028 | 0.3634 | No |
| 56 | TARBP2 |  |  | 2986 | 0.025 | 0.3562 | No |
| 57 | DUT |  |  | 3005 | 0.025 | 0.3565 | No |
| 58 | NUDT21 |  |  | 3013 | 0.025 | 0.3580 | No |
| 59 | POLR3GL |  |  | 3021 | 0.024 | 0.3594 | No |
| 60 | SF3A3 |  |  | 3130 | 0.022 | 0.3503 | No |
| 61 | ITPA |  |  | 3202 | 0.020 | 0.3448 | No |
| 62 | POLR2D |  |  | 3234 | 0.020 | 0.3433 | No |
| 63 | GTF2B |  |  | 3342 | 0.017 | 0.3339 | No |
| 64 | PCNA |  |  | 3540 | 0.013 | 0.3148 | No |
| 65 | POLR3C |  |  | 3554 | 0.013 | 0.3146 | No |
| 66 | ELL |  |  | 3655 | 0.011 | 0.3053 | No |
| 67 | RFC5 |  |  | 3697 | 0.010 | 0.3019 | No |
| 68 | ADA |  |  | 3919 | 0.006 | 0.2798 | No |
| 69 | TAF13 |  |  | 4065 | 0.003 | 0.2652 | No |
| 70 | GTF2H3 |  |  | 4124 | 0.002 | 0.2594 | No |
| 71 | AAAS |  |  | 4210 | 0.001 | 0.2507 | No |
| 72 | BRF2 |  |  | 4429 | -0.003 | 0.2287 | No |
| 73 | POLA2 |  |  | 4497 | -0.004 | 0.2222 | No |
| 74 | TMED2 |  |  | 4551 | -0.006 | 0.2172 | No |
| 75 | POLR2G |  |  | 4600 | -0.006 | 0.2129 | No |
| 76 | GTF3C5 |  |  | 4667 | -0.007 | 0.2067 | No |
| 77 | POLR2F |  |  | 4700 | -0.007 | 0.2041 | No |
| 78 | POLD1 |  |  | 4718 | -0.008 | 0.2030 | No |
| 79 | UMPS |  |  | 4759 | -0.008 | 0.1996 | No |
| 80 | ERCC8 |  |  | 4857 | -0.010 | 0.1906 | No |
| 81 | POLR1C |  |  | 4896 | -0.011 | 0.1876 | No |
| 82 | SNAPC4 |  |  | 5111 | -0.014 | 0.1669 | No |
| 83 | SUPT5H |  |  | 5288 | -0.017 | 0.1503 | No |
| 84 | ERCC3 |  |  | 5408 | -0.019 | 0.1398 | No |
| 85 | UPF3B |  |  | 5509 | -0.020 | 0.1313 | No |
| 86 | TP53 |  |  | 5557 | -0.021 | 0.1283 | No |
| 87 | TK2 |  |  | 5756 | -0.025 | 0.1102 | No |
| 88 | VPS37B |  |  | 5829 | -0.026 | 0.1050 | No |
| 89 | CANT1 |  |  | 5935 | -0.028 | 0.0967 | No |
| 90 | NT5C3A |  |  | 5965 | -0.029 | 0.0963 | No |
| 91 | RFC3 |  |  | 6030 | -0.030 | 0.0923 | No |
| 92 | RRM2B |  |  | 6045 | -0.030 | 0.0935 | No |
| 93 | NT5C |  |  | 6076 | -0.030 | 0.0931 | No |
| 94 | CETN2 |  |  | 6331 | -0.035 | 0.0701 | No |
| 95 | TAF1C |  |  | 6370 | -0.036 | 0.0694 | No |
| 96 | DCTN4 |  |  | 6526 | -0.039 | 0.0569 | No |
| 97 | VPS37D |  |  | 6580 | -0.040 | 0.0550 | No |
| 98 | POLL |  |  | 6718 | -0.043 | 0.0448 | No |
| 99 | ERCC4 |  |  | 6735 | -0.044 | 0.0470 | No |
| 100 | GSDME |  |  | 6833 | -0.046 | 0.0410 | No |
| 101 | ERCC2 |  |  | 6845 | -0.046 | 0.0439 | No |
| 102 | DDB1 |  |  | 6857 | -0.046 | 0.0469 | No |
| 103 | NFX1 |  |  | 6914 | -0.048 | 0.0453 | No |
| 104 | XPC |  |  | 6915 | -0.048 | 0.0495 | No |
| 105 | POLD3 |  |  | 7016 | -0.051 | 0.0437 | No |
| 106 | SRSF6 |  |  | 7028 | -0.051 | 0.0471 | No |
| 107 | POLH |  |  | 7029 | -0.051 | 0.0515 | No |
| 108 | POLA1 |  |  | 7180 | -0.055 | 0.0410 | No |
| 109 | VPS28 |  |  | 7215 | -0.056 | 0.0424 | No |
| 110 | PRIM1 |  |  | 7308 | -0.058 | 0.0381 | No |
| 111 | NELFCD |  |  | 7396 | -0.061 | 0.0345 | No |
| 112 | USP11 |  |  | 7439 | -0.061 | 0.0356 | No |
| 113 | LIG1 |  |  | 7482 | -0.063 | 0.0368 | No |
| 114 | EIF1B |  |  | 7516 | -0.064 | 0.0390 | No |
| 115 | POLR2A |  |  | 7582 | -0.066 | 0.0381 | No |
| 116 | BCAM |  |  | 7587 | -0.066 | 0.0435 | No |
| 117 | NCBP2 |  |  | 7645 | -0.068 | 0.0436 | No |
| 118 | SMAD5 |  |  | 8035 | -0.083 | 0.0110 | No |
| 119 | NME3 |  |  | 8096 | -0.085 | 0.0123 | No |
| 120 | DDB2 |  |  | 8121 | -0.086 | 0.0174 | No |
| 121 | BCAP31 |  |  | 8279 | -0.093 | 0.0095 | No |
| 122 | POM121 |  |  | 8282 | -0.094 | 0.0176 | No |
| 123 | RFC4 |  |  | 8292 | -0.094 | 0.0249 | No |
| 124 | RNMT |  |  | 8472 | -0.103 | 0.0156 | No |
| 125 | DAD1 |  |  | 8655 | -0.114 | 0.0070 | No |
| 126 | SDCBP |  |  | 8790 | -0.124 | 0.0042 | No |
| 127 | ADCY6 |  |  | 8807 | -0.125 | 0.0135 | No |
| 128 | NUDT9 |  |  | 8809 | -0.125 | 0.0245 | No |
| 129 | ARL6IP1 |  |  | 9038 | -0.146 | 0.0139 | No |
| 130 | SEC61A1 |  |  | 9343 | -0.188 | -0.0007 | No |
| 131 | DGCR8 |  |  | 9359 | -0.191 | 0.0146 | No |
| 132 | REV3L |  |  | 9431 | -0.208 | 0.0255 | No |
| 133 | PDE4B |  |  | 9522 | -0.231 | 0.0366 | No |
Table: GSEA details [plain text format]

  

Fig 2: HALLMARK\_DNA\_REPAIR: Random ES distribution      
 Gene set null distribution of ES for **HALLMARK\_DNA\_REPAIR**

  
