# Supplementary material for: Modeling glioblastoma heterogeneity as a dynamic network of cell states
Source: Mol Syst Biol. 2021 Sep 16;17(9):e10105. doi: 10.15252/msb.202010105 (PMC8444284; doi:10.15252/msb.202010105)
Supplement: Supplementary file 5 — Source Data for Figure 3 [file MSB-17-e10105-s001.zip › Figure3A_sourcedata/GSEA_3065/hallmarks_state1.GseaPreranked.1623416262439/HALLMARK_E2F_TARGETS.html]

Details for gene set HALLMARK\_E2F\_TARGETS[GSEA]

|  || Dataset | state1 |
| Phenotype | NoPhenotypeAvailable |
| Upregulated in class | na\_pos |
| GeneSet | HALLMARK\_E2F\_TARGETS |
| Enrichment Score (ES) | 0.40771848 |
| Normalized Enrichment Score (NES) | 1.5880425 |
| Nominal p-value | 0.0 |
| FDR q-value | 0.019735577 |
| FWER p-Value | 0.114 |
Table: GSEA Results Summary

  

Fig 1: Enrichment plot: HALLMARK\_E2F\_TARGETS      
 Profile of the Running ES Score & Positions of GeneSet Members on the Rank Ordered List

  

| PROBE | GENE SYMBOL | GENE\_TITLE | RANK IN GENE LIST | RANK METRIC SCORE | RUNNING ES | CORE ENRICHMENT || 1 | HMGA1 |  |  | 8 | 0.736 | 0.0401 | Yes |
| 2 | UBE2S |  |  | 19 | 0.634 | 0.0742 | Yes |
| 3 | PTTG1 |  |  | 40 | 0.520 | 0.1010 | Yes |
| 4 | RANBP1 |  |  | 154 | 0.320 | 0.1071 | Yes |
| 5 | SNRPB |  |  | 172 | 0.310 | 0.1226 | Yes |
| 6 | CDKN3 |  |  | 178 | 0.307 | 0.1391 | Yes |
| 7 | RAN |  |  | 194 | 0.297 | 0.1541 | Yes |
| 8 | CKS2 |  |  | 195 | 0.297 | 0.1706 | Yes |
| 9 | BIRC5 |  |  | 202 | 0.295 | 0.1863 | Yes |
| 10 | POP7 |  |  | 236 | 0.282 | 0.1986 | Yes |
| 11 | POLD2 |  |  | 241 | 0.280 | 0.2137 | Yes |
| 12 | JPT1 |  |  | 248 | 0.277 | 0.2285 | Yes |
| 13 | CDKN1A |  |  | 262 | 0.274 | 0.2424 | Yes |
| 14 | DEK |  |  | 296 | 0.261 | 0.2535 | Yes |
| 15 | CKS1B |  |  | 346 | 0.242 | 0.2618 | Yes |
| 16 | NME1 |  |  | 395 | 0.227 | 0.2695 | Yes |
| 17 | TUBB |  |  | 398 | 0.226 | 0.2819 | Yes |
| 18 | TUBG1 |  |  | 439 | 0.219 | 0.2899 | Yes |
| 19 | CDC20 |  |  | 469 | 0.212 | 0.2987 | Yes |
| 20 | AURKA |  |  | 550 | 0.196 | 0.3013 | Yes |
| 21 | DCTPP1 |  |  | 566 | 0.191 | 0.3103 | Yes |
| 22 | STMN1 |  |  | 763 | 0.163 | 0.2991 | Yes |
| 23 | PA2G4 |  |  | 780 | 0.160 | 0.3064 | Yes |
| 24 | NAA38 |  |  | 865 | 0.149 | 0.3060 | Yes |
| 25 | MTHFD2 |  |  | 899 | 0.145 | 0.3107 | Yes |
| 26 | HMGB3 |  |  | 907 | 0.144 | 0.3179 | Yes |
| 27 | POLE4 |  |  | 910 | 0.143 | 0.3257 | Yes |
| 28 | CDCA3 |  |  | 994 | 0.134 | 0.3246 | Yes |
| 29 | XRCC6 |  |  | 1025 | 0.131 | 0.3288 | Yes |
| 30 | RPA3 |  |  | 1039 | 0.129 | 0.3346 | Yes |
| 31 | SSRP1 |  |  | 1051 | 0.128 | 0.3406 | Yes |
| 32 | TK1 |  |  | 1056 | 0.127 | 0.3472 | Yes |
| 33 | PHF5A |  |  | 1071 | 0.125 | 0.3528 | Yes |
| 34 | RFC2 |  |  | 1094 | 0.123 | 0.3573 | Yes |
| 35 | AK2 |  |  | 1167 | 0.116 | 0.3564 | Yes |
| 36 | SLBP |  |  | 1219 | 0.111 | 0.3572 | Yes |
| 37 | CCNB2 |  |  | 1232 | 0.110 | 0.3621 | Yes |
| 38 | EIF2S1 |  |  | 1243 | 0.109 | 0.3671 | Yes |
| 39 | DDX39A |  |  | 1255 | 0.108 | 0.3720 | Yes |
| 40 | KIF4A |  |  | 1303 | 0.103 | 0.3728 | Yes |
| 41 | ASF1A |  |  | 1355 | 0.099 | 0.3730 | Yes |
| 42 | HUS1 |  |  | 1388 | 0.096 | 0.3751 | Yes |
| 43 | MAD2L1 |  |  | 1394 | 0.095 | 0.3799 | Yes |
| 44 | PLK1 |  |  | 1403 | 0.095 | 0.3843 | Yes |
| 45 | UBE2T |  |  | 1440 | 0.093 | 0.3858 | Yes |
| 46 | TBRG4 |  |  | 1442 | 0.093 | 0.3908 | Yes |
| 47 | DEPDC1 |  |  | 1478 | 0.090 | 0.3922 | Yes |
| 48 | HMMR |  |  | 1480 | 0.090 | 0.3971 | Yes |
| 49 | HNRNPD |  |  | 1509 | 0.088 | 0.3991 | Yes |
| 50 | CENPE |  |  | 1545 | 0.086 | 0.4003 | Yes |
| 51 | HMGB2 |  |  | 1570 | 0.085 | 0.4025 | Yes |
| 52 | ANP32E |  |  | 1706 | 0.075 | 0.3928 | Yes |
| 53 | NAP1L1 |  |  | 1717 | 0.074 | 0.3958 | Yes |
| 54 | GSPT1 |  |  | 1797 | 0.070 | 0.3916 | Yes |
| 55 | SYNCRIP |  |  | 1817 | 0.069 | 0.3934 | Yes |
| 56 | EXOSC8 |  |  | 1851 | 0.067 | 0.3937 | Yes |
| 57 | KIF22 |  |  | 1858 | 0.067 | 0.3968 | Yes |
| 58 | TCF19 |  |  | 1866 | 0.067 | 0.3998 | Yes |
| 59 | RAD51C |  |  | 1874 | 0.066 | 0.4028 | Yes |
| 60 | USP1 |  |  | 1921 | 0.064 | 0.4016 | Yes |
| 61 | TACC3 |  |  | 1949 | 0.063 | 0.4023 | Yes |
| 62 | RACGAP1 |  |  | 1994 | 0.060 | 0.4011 | Yes |
| 63 | LYAR |  |  | 1997 | 0.060 | 0.4042 | Yes |
| 64 | CDK4 |  |  | 2000 | 0.060 | 0.4073 | Yes |
| 65 | DLGAP5 |  |  | 2028 | 0.058 | 0.4077 | Yes |
| 66 | NCAPD2 |  |  | 2121 | 0.054 | 0.4012 | No |
| 67 | TIPIN |  |  | 2159 | 0.053 | 0.4003 | No |
| 68 | CDCA8 |  |  | 2166 | 0.052 | 0.4026 | No |
| 69 | KIF2C |  |  | 2224 | 0.050 | 0.3995 | No |
| 70 | UBR7 |  |  | 2306 | 0.047 | 0.3938 | No |
| 71 | SMC4 |  |  | 2308 | 0.047 | 0.3963 | No |
| 72 | TRIP13 |  |  | 2314 | 0.047 | 0.3983 | No |
| 73 | CENPM |  |  | 2348 | 0.045 | 0.3974 | No |
| 74 | CDC25B |  |  | 2370 | 0.044 | 0.3977 | No |
| 75 | RPA2 |  |  | 2385 | 0.044 | 0.3987 | No |
| 76 | NOLC1 |  |  | 2417 | 0.043 | 0.3979 | No |
| 77 | PAICS |  |  | 2456 | 0.041 | 0.3963 | No |
| 78 | MYBL2 |  |  | 2549 | 0.038 | 0.3889 | No |
| 79 | SPAG5 |  |  | 2566 | 0.038 | 0.3893 | No |
| 80 | DNMT1 |  |  | 2576 | 0.037 | 0.3905 | No |
| 81 | CTPS1 |  |  | 2594 | 0.036 | 0.3907 | No |
| 82 | UNG |  |  | 2727 | 0.032 | 0.3789 | No |
| 83 | MKI67 |  |  | 2760 | 0.031 | 0.3773 | No |
| 84 | PSMC3IP |  |  | 2766 | 0.031 | 0.3785 | No |
| 85 | RAD1 |  |  | 2856 | 0.029 | 0.3709 | No |
| 86 | ASF1B |  |  | 2929 | 0.027 | 0.3650 | No |
| 87 | CIT |  |  | 2993 | 0.025 | 0.3599 | No |
| 88 | CCNE1 |  |  | 2995 | 0.025 | 0.3612 | No |
| 89 | DUT |  |  | 3005 | 0.025 | 0.3616 | No |
| 90 | NUDT21 |  |  | 3013 | 0.025 | 0.3623 | No |
| 91 | RBBP7 |  |  | 3019 | 0.024 | 0.3631 | No |
| 92 | ORC2 |  |  | 3085 | 0.023 | 0.3577 | No |
| 93 | TMPO |  |  | 3093 | 0.023 | 0.3582 | No |
| 94 | DCK |  |  | 3176 | 0.021 | 0.3509 | No |
| 95 | RPA1 |  |  | 3239 | 0.020 | 0.3456 | No |
| 96 | PRPS1 |  |  | 3388 | 0.016 | 0.3312 | No |
| 97 | CDK1 |  |  | 3396 | 0.016 | 0.3314 | No |
| 98 | SPC24 |  |  | 3404 | 0.016 | 0.3315 | No |
| 99 | PPP1R8 |  |  | 3471 | 0.014 | 0.3255 | No |
| 100 | PCNA |  |  | 3540 | 0.013 | 0.3192 | No |
| 101 | ZW10 |  |  | 3571 | 0.012 | 0.3168 | No |
| 102 | SRSF2 |  |  | 3783 | 0.008 | 0.2955 | No |
| 103 | CNOT9 |  |  | 3826 | 0.008 | 0.2916 | No |
| 104 | MYC |  |  | 3964 | 0.005 | 0.2777 | No |
| 105 | DONSON |  |  | 4072 | 0.003 | 0.2668 | No |
| 106 | DSCC1 |  |  | 4360 | -0.002 | 0.2373 | No |
| 107 | WEE1 |  |  | 4385 | -0.003 | 0.2350 | No |
| 108 | POLA2 |  |  | 4497 | -0.004 | 0.2238 | No |
| 109 | BRMS1L |  |  | 4530 | -0.005 | 0.2208 | No |
| 110 | MELK |  |  | 4558 | -0.006 | 0.2183 | No |
| 111 | MCM3 |  |  | 4633 | -0.007 | 0.2110 | No |
| 112 | CDKN1B |  |  | 4655 | -0.007 | 0.2092 | No |
| 113 | SHMT1 |  |  | 4701 | -0.007 | 0.2050 | No |
| 114 | POLD1 |  |  | 4718 | -0.008 | 0.2038 | No |
| 115 | SPC25 |  |  | 4740 | -0.008 | 0.2021 | No |
| 116 | AURKB |  |  | 4764 | -0.008 | 0.2001 | No |
| 117 | RAD21 |  |  | 4916 | -0.011 | 0.1852 | No |
| 118 | RAD50 |  |  | 5001 | -0.013 | 0.1772 | No |
| 119 | TIMELESS |  |  | 5058 | -0.013 | 0.1722 | No |
| 120 | MXD3 |  |  | 5096 | -0.014 | 0.1691 | No |
| 121 | GINS3 |  |  | 5119 | -0.014 | 0.1677 | No |
| 122 | BUB1B |  |  | 5187 | -0.015 | 0.1616 | No |
| 123 | TRA2B |  |  | 5321 | -0.017 | 0.1488 | No |
| 124 | CHEK1 |  |  | 5330 | -0.017 | 0.1489 | No |
| 125 | PLK4 |  |  | 5414 | -0.019 | 0.1414 | No |
| 126 | KPNA2 |  |  | 5461 | -0.020 | 0.1377 | No |
| 127 | TP53 |  |  | 5557 | -0.021 | 0.1291 | No |
| 128 | MCM5 |  |  | 5588 | -0.022 | 0.1272 | No |
| 129 | NUP153 |  |  | 5636 | -0.023 | 0.1236 | No |
| 130 | CDC25A |  |  | 5789 | -0.025 | 0.1093 | No |
| 131 | PSIP1 |  |  | 5794 | -0.025 | 0.1103 | No |
| 132 | EED |  |  | 6012 | -0.029 | 0.0895 | No |
| 133 | RFC3 |  |  | 6030 | -0.030 | 0.0894 | No |
| 134 | DIAPH3 |  |  | 6103 | -0.031 | 0.0837 | No |
| 135 | PRIM2 |  |  | 6118 | -0.031 | 0.0840 | No |
| 136 | GINS1 |  |  | 6149 | -0.032 | 0.0827 | No |
| 137 | NOP56 |  |  | 6205 | -0.033 | 0.0788 | No |
| 138 | RRM2 |  |  | 6214 | -0.033 | 0.0799 | No |
| 139 | NBN |  |  | 6228 | -0.034 | 0.0804 | No |
| 140 | CTCF |  |  | 6233 | -0.034 | 0.0818 | No |
| 141 | MCM2 |  |  | 6282 | -0.034 | 0.0788 | No |
| 142 | IPO7 |  |  | 6555 | -0.040 | 0.0529 | No |
| 143 | PPM1D |  |  | 6609 | -0.041 | 0.0497 | No |
| 144 | PAN2 |  |  | 6656 | -0.042 | 0.0473 | No |
| 145 | RFC1 |  |  | 6732 | -0.044 | 0.0420 | No |
| 146 | CSE1L |  |  | 6810 | -0.045 | 0.0365 | No |
| 147 | STAG1 |  |  | 6838 | -0.046 | 0.0363 | No |
| 148 | POLE |  |  | 6848 | -0.046 | 0.0379 | No |
| 149 | RNASEH2A |  |  | 6891 | -0.047 | 0.0362 | No |
| 150 | SRSF1 |  |  | 6896 | -0.047 | 0.0384 | No |
| 151 | ING3 |  |  | 6942 | -0.048 | 0.0364 | No |
| 152 | POLD3 |  |  | 7016 | -0.051 | 0.0317 | No |
| 153 | MLH1 |  |  | 7099 | -0.053 | 0.0262 | No |
| 154 | SMC3 |  |  | 7149 | -0.054 | 0.0241 | No |
| 155 | LMNB1 |  |  | 7166 | -0.055 | 0.0255 | No |
| 156 | EZH2 |  |  | 7260 | -0.057 | 0.0191 | No |
| 157 | CCP110 |  |  | 7307 | -0.058 | 0.0176 | No |
| 158 | MCM7 |  |  | 7397 | -0.061 | 0.0117 | No |
| 159 | MCM6 |  |  | 7414 | -0.061 | 0.0135 | No |
| 160 | LIG1 |  |  | 7482 | -0.063 | 0.0100 | No |
| 161 | MCM4 |  |  | 7658 | -0.068 | -0.0043 | No |
| 162 | CDKN2C |  |  | 7660 | -0.068 | -0.0006 | No |
| 163 | TOP2A |  |  | 7678 | -0.069 | 0.0015 | No |
| 164 | ILF3 |  |  | 7823 | -0.074 | -0.0092 | No |
| 165 | XPO1 |  |  | 7863 | -0.076 | -0.0091 | No |
| 166 | KIF18B |  |  | 7868 | -0.076 | -0.0052 | No |
| 167 | NUP205 |  |  | 7962 | -0.080 | -0.0104 | No |
| 168 | BARD1 |  |  | 7980 | -0.080 | -0.0077 | No |
| 169 | SMC6 |  |  | 8143 | -0.087 | -0.0196 | No |
| 170 | BRCA2 |  |  | 8192 | -0.089 | -0.0196 | No |
| 171 | RAD51AP1 |  |  | 8227 | -0.091 | -0.0181 | No |
| 172 | PDS5B |  |  | 8300 | -0.094 | -0.0203 | No |
| 173 | WDR90 |  |  | 8314 | -0.095 | -0.0163 | No |
| 174 | PMS2 |  |  | 8350 | -0.097 | -0.0146 | No |
| 175 | MSH2 |  |  | 8392 | -0.099 | -0.0133 | No |
| 176 | MRE11 |  |  | 8514 | -0.106 | -0.0199 | No |
| 177 | MMS22L |  |  | 8547 | -0.108 | -0.0172 | No |
| 178 | SMC1A |  |  | 8681 | -0.116 | -0.0245 | No |
| 179 | TFRC |  |  | 8715 | -0.119 | -0.0213 | No |
| 180 | CBX5 |  |  | 8780 | -0.124 | -0.0211 | No |
| 181 | ORC6 |  |  | 8888 | -0.133 | -0.0247 | No |
| 182 | GINS4 |  |  | 8919 | -0.136 | -0.0203 | No |
| 183 | ATAD2 |  |  | 8948 | -0.138 | -0.0155 | No |
| 184 | LBR |  |  | 9264 | -0.174 | -0.0383 | No |
| 185 | NUP107 |  |  | 9293 | -0.179 | -0.0313 | No |
| 186 | HELLS |  |  | 9406 | -0.201 | -0.0317 | No |
| 187 | NASP |  |  | 9437 | -0.208 | -0.0232 | No |
| 188 | PRDX4 |  |  | 9457 | -0.215 | -0.0132 | No |
| 189 | PNN |  |  | 9517 | -0.230 | -0.0066 | No |
| 190 | PRKDC |  |  | 9539 | -0.236 | 0.0044 | No |
| 191 | BRCA1 |  |  | 9600 | -0.258 | 0.0125 | No |
| 192 | LUC7L3 |  |  | 9667 | -0.292 | 0.0219 | No |
Table: GSEA details [plain text format]

  

Fig 2: HALLMARK\_E2F\_TARGETS: Random ES distribution      
 Gene set null distribution of ES for **HALLMARK\_E2F\_TARGETS**

  
