# Supplementary material for: Modeling glioblastoma heterogeneity as a dynamic network of cell states
Source: Mol Syst Biol. 2021 Sep 16;17(9):e10105. doi: 10.15252/msb.202010105 (PMC8444284; doi:10.15252/msb.202010105)
Supplement: Supplementary file 5 — Source Data for Figure 3 [file MSB-17-e10105-s001.zip › Figure3A_sourcedata/GSEA_3065/hallmarks_state1.GseaPreranked.1623416262439/HALLMARK_EPITHELIAL_MESENCHYMAL_TRANSITION.html]

Details for gene set HALLMARK\_EPITHELIAL\_MESENCHYMAL\_TRANSITION[GSEA]

|  || Dataset | state1 |
| Phenotype | NoPhenotypeAvailable |
| Upregulated in class | na\_pos |
| GeneSet | HALLMARK\_EPITHELIAL\_MESENCHYMAL\_TRANSITION |
| Enrichment Score (ES) | 0.36594656 |
| Normalized Enrichment Score (NES) | 1.3764403 |
| Nominal p-value | 0.022522522 |
| FDR q-value | 0.09808234 |
| FWER p-Value | 0.582 |
Table: GSEA Results Summary

  

Fig 1: Enrichment plot: HALLMARK\_EPITHELIAL\_MESENCHYMAL\_TRANSITION      
 Profile of the Running ES Score & Positions of GeneSet Members on the Rank Ordered List

  

| PROBE | GENE SYMBOL | GENE\_TITLE | RANK IN GENE LIST | RANK METRIC SCORE | RUNNING ES | CORE ENRICHMENT || 1 | LGALS1 |  |  | 4 | 0.828 | 0.0347 | Yes |
| 2 | SCG2 |  |  | 5 | 0.825 | 0.0696 | Yes |
| 3 | POSTN |  |  | 11 | 0.689 | 0.0983 | Yes |
| 4 | PMEPA1 |  |  | 12 | 0.687 | 0.1273 | Yes |
| 5 | IGFBP3 |  |  | 15 | 0.664 | 0.1552 | Yes |
| 6 | DKK1 |  |  | 16 | 0.656 | 0.1830 | Yes |
| 7 | SPP1 |  |  | 17 | 0.642 | 0.2102 | Yes |
| 8 | TPM4 |  |  | 22 | 0.623 | 0.2362 | Yes |
| 9 | PTHLH |  |  | 31 | 0.556 | 0.2589 | Yes |
| 10 | DPYSL3 |  |  | 74 | 0.411 | 0.2720 | Yes |
| 11 | PDLIM4 |  |  | 89 | 0.385 | 0.2868 | Yes |
| 12 | TNFRSF12A |  |  | 104 | 0.364 | 0.3008 | Yes |
| 13 | GLIPR1 |  |  | 111 | 0.352 | 0.3151 | Yes |
| 14 | TPM1 |  |  | 132 | 0.335 | 0.3272 | Yes |
| 15 | TAGLN |  |  | 147 | 0.326 | 0.3396 | Yes |
| 16 | VIM |  |  | 175 | 0.308 | 0.3498 | Yes |
| 17 | LOXL1 |  |  | 244 | 0.279 | 0.3547 | Yes |
| 18 | MYLK |  |  | 260 | 0.275 | 0.3648 | Yes |
| 19 | JUN |  |  | 387 | 0.229 | 0.3615 | Yes |
| 20 | NT5E |  |  | 462 | 0.213 | 0.3630 | Yes |
| 21 | COL1A2 |  |  | 517 | 0.201 | 0.3659 | Yes |
| 22 | COL6A2 |  |  | 661 | 0.177 | 0.3588 | No |
| 23 | TPM2 |  |  | 753 | 0.165 | 0.3564 | No |
| 24 | CAPG |  |  | 866 | 0.149 | 0.3512 | No |
| 25 | BDNF |  |  | 963 | 0.138 | 0.3473 | No |
| 26 | CALD1 |  |  | 968 | 0.137 | 0.3527 | No |
| 27 | NTM |  |  | 1002 | 0.134 | 0.3549 | No |
| 28 | GADD45A |  |  | 1082 | 0.124 | 0.3521 | No |
| 29 | CXCL12 |  |  | 1115 | 0.121 | 0.3539 | No |
| 30 | THBS1 |  |  | 1118 | 0.121 | 0.3588 | No |
| 31 | FLNA |  |  | 1212 | 0.111 | 0.3540 | No |
| 32 | SERPINE1 |  |  | 1250 | 0.108 | 0.3548 | No |
| 33 | PRRX1 |  |  | 1430 | 0.094 | 0.3404 | No |
| 34 | PFN2 |  |  | 1462 | 0.091 | 0.3411 | No |
| 35 | PLAUR |  |  | 1463 | 0.091 | 0.3450 | No |
| 36 | SDC1 |  |  | 1482 | 0.090 | 0.3469 | No |
| 37 | WNT5A |  |  | 1503 | 0.089 | 0.3486 | No |
| 38 | MEST |  |  | 1582 | 0.084 | 0.3442 | No |
| 39 | ECM1 |  |  | 1655 | 0.078 | 0.3401 | No |
| 40 | ACTA2 |  |  | 1800 | 0.069 | 0.3283 | No |
| 41 | LOX |  |  | 1825 | 0.068 | 0.3287 | No |
| 42 | RHOB |  |  | 1914 | 0.064 | 0.3224 | No |
| 43 | SFRP1 |  |  | 1944 | 0.063 | 0.3221 | No |
| 44 | TGFB1 |  |  | 2040 | 0.058 | 0.3148 | No |
| 45 | SDC4 |  |  | 2111 | 0.054 | 0.3099 | No |
| 46 | CD44 |  |  | 2193 | 0.051 | 0.3038 | No |
| 47 | GADD45B |  |  | 2252 | 0.049 | 0.2999 | No |
| 48 | ITGA5 |  |  | 2302 | 0.047 | 0.2969 | No |
| 49 | TIMP1 |  |  | 2497 | 0.040 | 0.2787 | No |
| 50 | GEM |  |  | 2691 | 0.033 | 0.2603 | No |
| 51 | FN1 |  |  | 2705 | 0.033 | 0.2603 | No |
| 52 | CDH6 |  |  | 2796 | 0.031 | 0.2524 | No |
| 53 | ENO2 |  |  | 3161 | 0.021 | 0.2159 | No |
| 54 | MSX1 |  |  | 3279 | 0.019 | 0.2047 | No |
| 55 | DST |  |  | 3373 | 0.017 | 0.1959 | No |
| 56 | TGFBI |  |  | 3688 | 0.010 | 0.1641 | No |
| 57 | FAS |  |  | 3812 | 0.008 | 0.1518 | No |
| 58 | ITGB1 |  |  | 3956 | 0.005 | 0.1374 | No |
| 59 | FSTL3 |  |  | 4084 | 0.003 | 0.1245 | No |
| 60 | FGF2 |  |  | 4570 | -0.006 | 0.0750 | No |
| 61 | FBN2 |  |  | 4789 | -0.009 | 0.0530 | No |
| 62 | PVR |  |  | 4826 | -0.009 | 0.0497 | No |
| 63 | COL5A3 |  |  | 4894 | -0.011 | 0.0433 | No |
| 64 | PDGFRB |  |  | 5041 | -0.013 | 0.0289 | No |
| 65 | LOXL2 |  |  | 5423 | -0.019 | -0.0094 | No |
| 66 | PLOD1 |  |  | 5662 | -0.023 | -0.0329 | No |
| 67 | P3H1 |  |  | 6130 | -0.031 | -0.0794 | No |
| 68 | GAS1 |  |  | 6510 | -0.039 | -0.1167 | No |
| 69 | MMP14 |  |  | 6548 | -0.039 | -0.1188 | No |
| 70 | FERMT2 |  |  | 6579 | -0.040 | -0.1202 | No |
| 71 | FUCA1 |  |  | 6606 | -0.041 | -0.1211 | No |
| 72 | LAMC1 |  |  | 6974 | -0.049 | -0.1567 | No |
| 73 | WIPF1 |  |  | 7001 | -0.050 | -0.1572 | No |
| 74 | MMP2 |  |  | 7051 | -0.051 | -0.1601 | No |
| 75 | TIMP3 |  |  | 7100 | -0.053 | -0.1628 | No |
| 76 | FBN1 |  |  | 7190 | -0.055 | -0.1695 | No |
| 77 | MCM7 |  |  | 7397 | -0.061 | -0.1881 | No |
| 78 | EMP3 |  |  | 7404 | -0.061 | -0.1862 | No |
| 79 | SERPINH1 |  |  | 7462 | -0.062 | -0.1894 | No |
| 80 | GJA1 |  |  | 7471 | -0.062 | -0.1876 | No |
| 81 | ELN |  |  | 7510 | -0.064 | -0.1888 | No |
| 82 | CAP2 |  |  | 7580 | -0.066 | -0.1931 | No |
| 83 | QSOX1 |  |  | 7628 | -0.067 | -0.1950 | No |
| 84 | COL5A1 |  |  | 7642 | -0.067 | -0.1935 | No |
| 85 | EFEMP2 |  |  | 7651 | -0.068 | -0.1915 | No |
| 86 | LAMA1 |  |  | 7949 | -0.079 | -0.2186 | No |
| 87 | BMP1 |  |  | 8021 | -0.082 | -0.2224 | No |
| 88 | TFPI2 |  |  | 8026 | -0.082 | -0.2193 | No |
| 89 | PPIB |  |  | 8099 | -0.085 | -0.2231 | No |
| 90 | GPX7 |  |  | 8128 | -0.086 | -0.2223 | No |
| 91 | SNTB1 |  |  | 8141 | -0.087 | -0.2199 | No |
| 92 | COLGALT1 |  |  | 8276 | -0.093 | -0.2297 | No |
| 93 | ID2 |  |  | 8360 | -0.097 | -0.2341 | No |
| 94 | NNMT |  |  | 8484 | -0.104 | -0.2423 | No |
| 95 | COL11A1 |  |  | 8666 | -0.115 | -0.2560 | No |
| 96 | ADAM12 |  |  | 8684 | -0.116 | -0.2528 | No |
| 97 | SERPINE2 |  |  | 8736 | -0.120 | -0.2529 | No |
| 98 | COPA |  |  | 8814 | -0.126 | -0.2555 | No |
| 99 | FSTL1 |  |  | 8976 | -0.141 | -0.2661 | No |
| 100 | PCOLCE |  |  | 9016 | -0.144 | -0.2640 | No |
| 101 | FBLN1 |  |  | 9043 | -0.146 | -0.2605 | No |
| 102 | CD59 |  |  | 9097 | -0.152 | -0.2595 | No |
| 103 | SLC6A8 |  |  | 9101 | -0.152 | -0.2533 | No |
| 104 | THY1 |  |  | 9119 | -0.153 | -0.2486 | No |
| 105 | VCAN |  |  | 9151 | -0.158 | -0.2451 | No |
| 106 | COL16A1 |  |  | 9160 | -0.160 | -0.2391 | No |
| 107 | PLOD2 |  |  | 9169 | -0.161 | -0.2331 | No |
| 108 | CDH2 |  |  | 9187 | -0.163 | -0.2279 | No |
| 109 | IGFBP2 |  |  | 9209 | -0.167 | -0.2230 | No |
| 110 | VEGFA |  |  | 9220 | -0.168 | -0.2169 | No |
| 111 | NOTCH2 |  |  | 9279 | -0.177 | -0.2154 | No |
| 112 | PMP22 |  |  | 9392 | -0.199 | -0.2184 | No |
| 113 | CALU |  |  | 9418 | -0.205 | -0.2123 | No |
| 114 | HTRA1 |  |  | 9515 | -0.229 | -0.2125 | No |
| 115 | SGCB |  |  | 9520 | -0.231 | -0.2031 | No |
| 116 | PLOD3 |  |  | 9529 | -0.233 | -0.1941 | No |
| 117 | CADM1 |  |  | 9532 | -0.234 | -0.1844 | No |
| 118 | COL5A2 |  |  | 9551 | -0.240 | -0.1761 | No |
| 119 | CDH11 |  |  | 9570 | -0.248 | -0.1674 | No |
| 120 | TNC |  |  | 9605 | -0.262 | -0.1598 | No |
| 121 | APLP1 |  |  | 9615 | -0.264 | -0.1496 | No |
| 122 | ITGAV |  |  | 9650 | -0.282 | -0.1411 | No |
| 123 | ITGB5 |  |  | 9676 | -0.296 | -0.1312 | No |
| 124 | SPARC |  |  | 9738 | -0.347 | -0.1227 | No |
| 125 | GPC1 |  |  | 9751 | -0.356 | -0.1089 | No |
| 126 | LRP1 |  |  | 9771 | -0.381 | -0.0947 | No |
| 127 | COL4A2 |  |  | 9786 | -0.402 | -0.0791 | No |
| 128 | SAT1 |  |  | 9807 | -0.445 | -0.0623 | No |
| 129 | PTX3 |  |  | 9827 | -0.517 | -0.0424 | No |
| 130 | COL4A1 |  |  | 9834 | -0.552 | -0.0197 | No |
| 131 | MATN2 |  |  | 9837 | -0.571 | 0.0043 | No |
Table: GSEA details [plain text format]

  

Fig 2: HALLMARK\_EPITHELIAL\_MESENCHYMAL\_TRANSITION: Random ES distribution      
 Gene set null distribution of ES for **HALLMARK\_EPITHELIAL\_MESENCHYMAL\_TRANSITION**

  
