# Supplementary material for: Modeling glioblastoma heterogeneity as a dynamic network of cell states
Source: Mol Syst Biol. 2021 Sep 16;17(9):e10105. doi: 10.15252/msb.202010105 (PMC8444284; doi:10.15252/msb.202010105)
Supplement: Supplementary file 5 — Source Data for Figure 3 [file MSB-17-e10105-s001.zip › Figure3A_sourcedata/GSEA_3065/hallmarks_state1.GseaPreranked.1623416262439/HALLMARK_ESTROGEN_RESPONSE_EARLY.html]

Details for gene set HALLMARK\_ESTROGEN\_RESPONSE\_EARLY[GSEA]

|  || Dataset | state1 |
| Phenotype | NoPhenotypeAvailable |
| Upregulated in class | na\_neg |
| GeneSet | HALLMARK\_ESTROGEN\_RESPONSE\_EARLY |
| Enrichment Score (ES) | -0.27626792 |
| Normalized Enrichment Score (NES) | -0.98929775 |
| Nominal p-value | 0.4686907 |
| FDR q-value | 0.6447678 |
| FWER p-Value | 1.0 |
Table: GSEA Results Summary

  

Fig 1: Enrichment plot: HALLMARK\_ESTROGEN\_RESPONSE\_EARLY      
 Profile of the Running ES Score & Positions of GeneSet Members on the Rank Ordered List

  

| PROBE | GENE SYMBOL | GENE\_TITLE | RANK IN GENE LIST | RANK METRIC SCORE | RUNNING ES | CORE ENRICHMENT || 1 | CCND1 |  |  | 29 | 0.566 | 0.0386 | No |
| 2 | PMAIP1 |  |  | 93 | 0.380 | 0.0600 | No |
| 3 | MYOF |  |  | 115 | 0.348 | 0.0834 | No |
| 4 | FHL2 |  |  | 141 | 0.328 | 0.1049 | No |
| 5 | DYNLT3 |  |  | 149 | 0.325 | 0.1280 | No |
| 6 | SLC2A1 |  |  | 235 | 0.283 | 0.1401 | No |
| 7 | TPBG |  |  | 315 | 0.256 | 0.1508 | No |
| 8 | SYNGR1 |  |  | 348 | 0.241 | 0.1652 | No |
| 9 | NBL1 |  |  | 413 | 0.223 | 0.1750 | No |
| 10 | INPP5F |  |  | 518 | 0.201 | 0.1791 | No |
| 11 | SH3BP5 |  |  | 529 | 0.199 | 0.1927 | No |
| 12 | FKBP4 |  |  | 547 | 0.196 | 0.2054 | No |
| 13 | TFAP2C |  |  | 602 | 0.186 | 0.2135 | No |
| 14 | FDFT1 |  |  | 635 | 0.181 | 0.2235 | No |
| 15 | SVIL |  |  | 676 | 0.175 | 0.2323 | No |
| 16 | UGCG |  |  | 691 | 0.173 | 0.2435 | No |
| 17 | B4GALT1 |  |  | 802 | 0.157 | 0.2438 | No |
| 18 | STC2 |  |  | 922 | 0.142 | 0.2420 | No |
| 19 | BLVRB |  |  | 945 | 0.140 | 0.2501 | No |
| 20 | OLFML3 |  |  | 1083 | 0.124 | 0.2452 | No |
| 21 | CXCL12 |  |  | 1115 | 0.121 | 0.2509 | No |
| 22 | TIPARP |  |  | 1142 | 0.118 | 0.2569 | No |
| 23 | NCOR2 |  |  | 1294 | 0.104 | 0.2491 | No |
| 24 | SLC7A5 |  |  | 1312 | 0.102 | 0.2548 | No |
| 25 | PRSS23 |  |  | 1356 | 0.099 | 0.2577 | No |
| 26 | TIAM1 |  |  | 1395 | 0.095 | 0.2608 | No |
| 27 | NXT1 |  |  | 1448 | 0.093 | 0.2623 | No |
| 28 | DHCR7 |  |  | 1553 | 0.086 | 0.2579 | No |
| 29 | BAG1 |  |  | 1614 | 0.082 | 0.2578 | No |
| 30 | CHPT1 |  |  | 1808 | 0.069 | 0.2431 | No |
| 31 | SLC1A1 |  |  | 1828 | 0.068 | 0.2461 | No |
| 32 | ARL3 |  |  | 1833 | 0.068 | 0.2507 | No |
| 33 | CD44 |  |  | 2193 | 0.051 | 0.2177 | No |
| 34 | RBBP8 |  |  | 2212 | 0.051 | 0.2196 | No |
| 35 | PPIF |  |  | 2457 | 0.041 | 0.1976 | No |
| 36 | NRIP1 |  |  | 2471 | 0.041 | 0.1993 | No |
| 37 | ITPK1 |  |  | 2758 | 0.031 | 0.1723 | No |
| 38 | NADSYN1 |  |  | 2804 | 0.030 | 0.1699 | No |
| 39 | TSKU |  |  | 2843 | 0.029 | 0.1681 | No |
| 40 | SLC9A3R1 |  |  | 2862 | 0.029 | 0.1684 | No |
| 41 | FOXC1 |  |  | 3009 | 0.025 | 0.1553 | No |
| 42 | KAZN |  |  | 3327 | 0.018 | 0.1241 | No |
| 43 | SNX24 |  |  | 3347 | 0.017 | 0.1234 | No |
| 44 | MREG |  |  | 3361 | 0.017 | 0.1233 | No |
| 45 | MAST4 |  |  | 3414 | 0.016 | 0.1191 | No |
| 46 | TOB1 |  |  | 3439 | 0.015 | 0.1178 | No |
| 47 | UNC119 |  |  | 3459 | 0.015 | 0.1169 | No |
| 48 | PAPSS2 |  |  | 3712 | 0.010 | 0.0918 | No |
| 49 | RARA |  |  | 3735 | 0.009 | 0.0903 | No |
| 50 | MYC |  |  | 3964 | 0.005 | 0.0673 | No |
| 51 | FLNB |  |  | 4168 | 0.001 | 0.0466 | No |
| 52 | SEC14L2 |  |  | 4262 | -0.000 | 0.0371 | No |
| 53 | KLF10 |  |  | 4486 | -0.004 | 0.0146 | No |
| 54 | ALDH3B1 |  |  | 4547 | -0.005 | 0.0088 | No |
| 55 | MED24 |  |  | 4672 | -0.007 | -0.0033 | No |
| 56 | KDM4B |  |  | 5024 | -0.013 | -0.0383 | No |
| 57 | MYBBP1A |  |  | 5147 | -0.015 | -0.0497 | No |
| 58 | TGIF2 |  |  | 5192 | -0.015 | -0.0531 | No |
| 59 | SIAH2 |  |  | 5328 | -0.017 | -0.0657 | No |
| 60 | ELF1 |  |  | 5444 | -0.019 | -0.0760 | No |
| 61 | MYBL1 |  |  | 5455 | -0.019 | -0.0756 | No |
| 62 | XBP1 |  |  | 5510 | -0.020 | -0.0797 | No |
| 63 | AKAP1 |  |  | 5582 | -0.022 | -0.0854 | No |
| 64 | WFS1 |  |  | 5763 | -0.025 | -0.1020 | No |
| 65 | RRP12 |  |  | 5826 | -0.026 | -0.1065 | No |
| 66 | CANT1 |  |  | 5935 | -0.028 | -0.1154 | No |
| 67 | ISG20L2 |  |  | 5946 | -0.028 | -0.1144 | No |
| 68 | BHLHE40 |  |  | 6102 | -0.031 | -0.1280 | No |
| 69 | ADCY1 |  |  | 6440 | -0.037 | -0.1598 | No |
| 70 | MED13L |  |  | 6507 | -0.039 | -0.1637 | No |
| 71 | FKBP5 |  |  | 6519 | -0.039 | -0.1620 | No |
| 72 | RPS6KA2 |  |  | 6745 | -0.044 | -0.1818 | No |
| 73 | FAM102A |  |  | 6869 | -0.046 | -0.1910 | No |
| 74 | REEP1 |  |  | 6897 | -0.047 | -0.1903 | No |
| 75 | RAB31 |  |  | 7256 | -0.057 | -0.2228 | No |
| 76 | OLFM1 |  |  | 7262 | -0.057 | -0.2191 | No |
| 77 | SCARB1 |  |  | 7359 | -0.060 | -0.2246 | No |
| 78 | ABCA3 |  |  | 7467 | -0.062 | -0.2309 | No |
| 79 | GJA1 |  |  | 7471 | -0.062 | -0.2267 | No |
| 80 | FARP1 |  |  | 7591 | -0.066 | -0.2340 | No |
| 81 | CELSR1 |  |  | 7680 | -0.069 | -0.2380 | No |
| 82 | SLC1A4 |  |  | 7745 | -0.072 | -0.2393 | No |
| 83 | ABLIM1 |  |  | 7806 | -0.074 | -0.2400 | No |
| 84 | AFF1 |  |  | 7824 | -0.074 | -0.2363 | No |
| 85 | ENDOD1 |  |  | 7904 | -0.078 | -0.2387 | No |
| 86 | AMFR |  |  | 8054 | -0.083 | -0.2479 | No |
| 87 | ADCY9 |  |  | 8107 | -0.086 | -0.2469 | No |
| 88 | ELOVL5 |  |  | 8115 | -0.086 | -0.2413 | No |
| 89 | PDLIM3 |  |  | 8278 | -0.093 | -0.2511 | No |
| 90 | CA12 |  |  | 8291 | -0.094 | -0.2454 | No |
| 91 | GLA |  |  | 8303 | -0.095 | -0.2396 | No |
| 92 | ADD3 |  |  | 8338 | -0.096 | -0.2360 | No |
| 93 | WWC1 |  |  | 8400 | -0.099 | -0.2350 | No |
| 94 | IGF1R |  |  | 8469 | -0.103 | -0.2344 | No |
| 95 | SEMA3B |  |  | 8556 | -0.109 | -0.2352 | No |
| 96 | FASN |  |  | 8694 | -0.117 | -0.2407 | No |
| 97 | MAPT |  |  | 8749 | -0.121 | -0.2373 | No |
| 98 | AR |  |  | 9065 | -0.148 | -0.2587 | No |
| 99 | CELSR2 |  |  | 9238 | -0.171 | -0.2637 | Yes |
| 100 | LRIG1 |  |  | 9256 | -0.173 | -0.2528 | Yes |
| 101 | HES1 |  |  | 9280 | -0.177 | -0.2422 | Yes |
| 102 | SLC26A2 |  |  | 9331 | -0.186 | -0.2336 | Yes |
| 103 | NAV2 |  |  | 9505 | -0.227 | -0.2347 | Yes |
| 104 | SLC39A6 |  |  | 9508 | -0.227 | -0.2182 | Yes |
| 105 | SLC16A1 |  |  | 9509 | -0.227 | -0.2016 | Yes |
| 106 | IL6ST |  |  | 9519 | -0.231 | -0.1856 | Yes |
| 107 | FOS |  |  | 9566 | -0.247 | -0.1722 | Yes |
| 108 | ABAT |  |  | 9646 | -0.278 | -0.1599 | Yes |
| 109 | PODXL |  |  | 9731 | -0.340 | -0.1435 | Yes |
| 110 | RHOBTB3 |  |  | 9789 | -0.406 | -0.1196 | Yes |
| 111 | RETREG1 |  |  | 9810 | -0.466 | -0.0874 | Yes |
| 112 | ABHD2 |  |  | 9812 | -0.476 | -0.0526 | Yes |
| 113 | TUBB2B |  |  | 9864 | -0.809 | 0.0015 | Yes |
Table: GSEA details [plain text format]

  

Fig 2: HALLMARK\_ESTROGEN\_RESPONSE\_EARLY: Random ES distribution      
 Gene set null distribution of ES for **HALLMARK\_ESTROGEN\_RESPONSE\_EARLY**

  
