# Supplementary material for: Modeling glioblastoma heterogeneity as a dynamic network of cell states
Source: Mol Syst Biol. 2021 Sep 16;17(9):e10105. doi: 10.15252/msb.202010105 (PMC8444284; doi:10.15252/msb.202010105)
Supplement: Supplementary file 5 — Source Data for Figure 3 [file MSB-17-e10105-s001.zip › Figure3A_sourcedata/GSEA_3065/hallmarks_state1.GseaPreranked.1623416262439/HALLMARK_ESTROGEN_RESPONSE_LATE.html]

Details for gene set HALLMARK\_ESTROGEN\_RESPONSE\_LATE[GSEA]

|  || Dataset | state1 |
| Phenotype | NoPhenotypeAvailable |
| Upregulated in class | na\_pos |
| GeneSet | HALLMARK\_ESTROGEN\_RESPONSE\_LATE |
| Enrichment Score (ES) | 0.326827 |
| Normalized Enrichment Score (NES) | 1.2019333 |
| Nominal p-value | 0.13360325 |
| FDR q-value | 0.24087994 |
| FWER p-Value | 0.967 |
Table: GSEA Results Summary

  

Fig 1: Enrichment plot: HALLMARK\_ESTROGEN\_RESPONSE\_LATE      
 Profile of the Running ES Score & Positions of GeneSet Members on the Rank Ordered List

  

| PROBE | GENE SYMBOL | GENE\_TITLE | RANK IN GENE LIST | RANK METRIC SCORE | RUNNING ES | CORE ENRICHMENT || 1 | CCND1 |  |  | 29 | 0.566 | 0.0416 | Yes |
| 2 | CAV1 |  |  | 34 | 0.536 | 0.0835 | Yes |
| 3 | MYOF |  |  | 115 | 0.348 | 0.1027 | Yes |
| 4 | DYNLT3 |  |  | 149 | 0.325 | 0.1250 | Yes |
| 5 | HOMER2 |  |  | 209 | 0.291 | 0.1419 | Yes |
| 6 | HPRT1 |  |  | 289 | 0.263 | 0.1545 | Yes |
| 7 | TPBG |  |  | 315 | 0.256 | 0.1722 | Yes |
| 8 | NBL1 |  |  | 413 | 0.223 | 0.1798 | Yes |
| 9 | PCP4 |  |  | 423 | 0.222 | 0.1964 | Yes |
| 10 | CDC20 |  |  | 469 | 0.212 | 0.2085 | Yes |
| 11 | DCXR |  |  | 474 | 0.211 | 0.2247 | Yes |
| 12 | FKBP4 |  |  | 547 | 0.196 | 0.2329 | Yes |
| 13 | TFAP2C |  |  | 602 | 0.186 | 0.2420 | Yes |
| 14 | COX6C |  |  | 617 | 0.183 | 0.2550 | Yes |
| 15 | FDFT1 |  |  | 635 | 0.181 | 0.2675 | Yes |
| 16 | TSPAN13 |  |  | 806 | 0.156 | 0.2624 | Yes |
| 17 | PERP |  |  | 876 | 0.148 | 0.2670 | Yes |
| 18 | ALDH3A2 |  |  | 913 | 0.143 | 0.2746 | Yes |
| 19 | CXCL14 |  |  | 925 | 0.142 | 0.2846 | Yes |
| 20 | BLVRB |  |  | 945 | 0.140 | 0.2938 | Yes |
| 21 | CXCL12 |  |  | 1115 | 0.121 | 0.2860 | Yes |
| 22 | NCOR2 |  |  | 1294 | 0.104 | 0.2760 | Yes |
| 23 | SLC7A5 |  |  | 1312 | 0.102 | 0.2823 | Yes |
| 24 | MDK |  |  | 1323 | 0.101 | 0.2892 | Yes |
| 25 | PRSS23 |  |  | 1356 | 0.099 | 0.2938 | Yes |
| 26 | CKB |  |  | 1368 | 0.098 | 0.3004 | Yes |
| 27 | TIAM1 |  |  | 1395 | 0.095 | 0.3052 | Yes |
| 28 | NXT1 |  |  | 1448 | 0.093 | 0.3072 | Yes |
| 29 | FABP5 |  |  | 1470 | 0.091 | 0.3122 | Yes |
| 30 | ISG20 |  |  | 1538 | 0.087 | 0.3122 | Yes |
| 31 | DHCR7 |  |  | 1553 | 0.086 | 0.3175 | Yes |
| 32 | MEST |  |  | 1582 | 0.084 | 0.3212 | Yes |
| 33 | BAG1 |  |  | 1614 | 0.082 | 0.3245 | Yes |
| 34 | ASS1 |  |  | 1675 | 0.077 | 0.3244 | Yes |
| 35 | ACOX2 |  |  | 1710 | 0.075 | 0.3268 | Yes |
| 36 | CHPT1 |  |  | 1808 | 0.069 | 0.3223 | No |
| 37 | ARL3 |  |  | 1833 | 0.068 | 0.3252 | No |
| 38 | LSR |  |  | 2102 | 0.055 | 0.3021 | No |
| 39 | CD44 |  |  | 2193 | 0.051 | 0.2969 | No |
| 40 | RBBP8 |  |  | 2212 | 0.051 | 0.2991 | No |
| 41 | PPIF |  |  | 2457 | 0.041 | 0.2773 | No |
| 42 | NRIP1 |  |  | 2471 | 0.041 | 0.2792 | No |
| 43 | JAK1 |  |  | 2494 | 0.040 | 0.2801 | No |
| 44 | BTG3 |  |  | 2514 | 0.039 | 0.2812 | No |
| 45 | GINS2 |  |  | 2567 | 0.038 | 0.2789 | No |
| 46 | ETFB |  |  | 2629 | 0.035 | 0.2754 | No |
| 47 | ITPK1 |  |  | 2758 | 0.031 | 0.2647 | No |
| 48 | GALE |  |  | 2848 | 0.029 | 0.2579 | No |
| 49 | SLC9A3R1 |  |  | 2862 | 0.029 | 0.2589 | No |
| 50 | KIF20A |  |  | 2897 | 0.028 | 0.2576 | No |
| 51 | RABEP1 |  |  | 2913 | 0.027 | 0.2581 | No |
| 52 | FOXC1 |  |  | 3009 | 0.025 | 0.2504 | No |
| 53 | MOCS2 |  |  | 3276 | 0.019 | 0.2246 | No |
| 54 | TOB1 |  |  | 3439 | 0.015 | 0.2092 | No |
| 55 | SORD |  |  | 3535 | 0.013 | 0.2005 | No |
| 56 | PAPSS2 |  |  | 3712 | 0.010 | 0.1832 | No |
| 57 | TST |  |  | 4000 | 0.004 | 0.1542 | No |
| 58 | PRKAR2B |  |  | 4073 | 0.003 | 0.1470 | No |
| 59 | UGDH |  |  | 4106 | 0.002 | 0.1439 | No |
| 60 | FLNB |  |  | 4168 | 0.001 | 0.1378 | No |
| 61 | SCUBE2 |  |  | 4482 | -0.004 | 0.1061 | No |
| 62 | NAB2 |  |  | 4488 | -0.004 | 0.1059 | No |
| 63 | ALDH3B1 |  |  | 4547 | -0.005 | 0.1004 | No |
| 64 | SLC2A8 |  |  | 4629 | -0.007 | 0.0926 | No |
| 65 | XRCC3 |  |  | 4796 | -0.009 | 0.0763 | No |
| 66 | IMPA2 |  |  | 5115 | -0.014 | 0.0448 | No |
| 67 | SLC29A1 |  |  | 5199 | -0.015 | 0.0376 | No |
| 68 | SIAH2 |  |  | 5328 | -0.017 | 0.0258 | No |
| 69 | STIL |  |  | 5357 | -0.018 | 0.0243 | No |
| 70 | PLK4 |  |  | 5414 | -0.019 | 0.0201 | No |
| 71 | XBP1 |  |  | 5510 | -0.020 | 0.0119 | No |
| 72 | UNC13B |  |  | 5702 | -0.024 | -0.0058 | No |
| 73 | WFS1 |  |  | 5763 | -0.025 | -0.0100 | No |
| 74 | HSPA4L |  |  | 5938 | -0.028 | -0.0256 | No |
| 75 | LARGE1 |  |  | 6016 | -0.029 | -0.0312 | No |
| 76 | ATP2B4 |  |  | 6357 | -0.036 | -0.0632 | No |
| 77 | FKBP5 |  |  | 6519 | -0.039 | -0.0766 | No |
| 78 | METTL3 |  |  | 6657 | -0.042 | -0.0873 | No |
| 79 | RPS6KA2 |  |  | 6745 | -0.044 | -0.0928 | No |
| 80 | FAM102A |  |  | 6869 | -0.046 | -0.1017 | No |
| 81 | RNASEH2A |  |  | 6891 | -0.047 | -0.1002 | No |
| 82 | DLG5 |  |  | 7127 | -0.054 | -0.1200 | No |
| 83 | CDC6 |  |  | 7140 | -0.054 | -0.1169 | No |
| 84 | RAB31 |  |  | 7256 | -0.057 | -0.1243 | No |
| 85 | OLFM1 |  |  | 7262 | -0.057 | -0.1203 | No |
| 86 | SCARB1 |  |  | 7359 | -0.060 | -0.1254 | No |
| 87 | PDCD4 |  |  | 7452 | -0.062 | -0.1300 | No |
| 88 | ABCA3 |  |  | 7467 | -0.062 | -0.1265 | No |
| 89 | IDH2 |  |  | 7549 | -0.065 | -0.1297 | No |
| 90 | FARP1 |  |  | 7591 | -0.066 | -0.1287 | No |
| 91 | TOP2A |  |  | 7678 | -0.069 | -0.1321 | No |
| 92 | SLC1A4 |  |  | 7745 | -0.072 | -0.1332 | No |
| 93 | AFF1 |  |  | 7824 | -0.074 | -0.1354 | No |
| 94 | TFPI2 |  |  | 8026 | -0.082 | -0.1495 | No |
| 95 | AMFR |  |  | 8054 | -0.083 | -0.1457 | No |
| 96 | ELOVL5 |  |  | 8115 | -0.086 | -0.1450 | No |
| 97 | PDLIM3 |  |  | 8278 | -0.093 | -0.1543 | No |
| 98 | CA12 |  |  | 8291 | -0.094 | -0.1481 | No |
| 99 | GLA |  |  | 8303 | -0.095 | -0.1417 | No |
| 100 | ADD3 |  |  | 8338 | -0.096 | -0.1376 | No |
| 101 | ID2 |  |  | 8360 | -0.097 | -0.1321 | No |
| 102 | SEMA3B |  |  | 8556 | -0.109 | -0.1436 | No |
| 103 | CA2 |  |  | 8636 | -0.113 | -0.1427 | No |
| 104 | SNX10 |  |  | 8641 | -0.114 | -0.1341 | No |
| 105 | MAPT |  |  | 8749 | -0.121 | -0.1355 | No |
| 106 | CPE |  |  | 9147 | -0.157 | -0.1638 | No |
| 107 | DNAJC1 |  |  | 9228 | -0.169 | -0.1586 | No |
| 108 | CELSR2 |  |  | 9238 | -0.171 | -0.1460 | No |
| 109 | PLXNB1 |  |  | 9326 | -0.186 | -0.1403 | No |
| 110 | SLC26A2 |  |  | 9331 | -0.186 | -0.1260 | No |
| 111 | SLC16A1 |  |  | 9509 | -0.227 | -0.1262 | No |
| 112 | IL6ST |  |  | 9519 | -0.231 | -0.1090 | No |
| 113 | FOS |  |  | 9566 | -0.247 | -0.0942 | No |
| 114 | FGFR3 |  |  | 9621 | -0.266 | -0.0788 | No |
| 115 | EMP2 |  |  | 9696 | -0.310 | -0.0619 | No |
| 116 | ABHD2 |  |  | 9812 | -0.476 | -0.0362 | No |
| 117 | CD9 |  |  | 9832 | -0.544 | 0.0048 | No |
Table: GSEA details [plain text format]

  

Fig 2: HALLMARK\_ESTROGEN\_RESPONSE\_LATE: Random ES distribution      
 Gene set null distribution of ES for **HALLMARK\_ESTROGEN\_RESPONSE\_LATE**

  
