# Supplementary material for: Modeling glioblastoma heterogeneity as a dynamic network of cell states
Source: Mol Syst Biol. 2021 Sep 16;17(9):e10105. doi: 10.15252/msb.202010105 (PMC8444284; doi:10.15252/msb.202010105)
Supplement: Supplementary file 5 — Source Data for Figure 3 [file MSB-17-e10105-s001.zip › Figure3A_sourcedata/GSEA_3065/hallmarks_state1.GseaPreranked.1623416262439/HALLMARK_FATTY_ACID_METABOLISM.html]

Details for gene set HALLMARK\_FATTY\_ACID\_METABOLISM[GSEA]

|  || Dataset | state1 |
| Phenotype | NoPhenotypeAvailable |
| Upregulated in class | na\_pos |
| GeneSet | HALLMARK\_FATTY\_ACID\_METABOLISM |
| Enrichment Score (ES) | 0.45959792 |
| Normalized Enrichment Score (NES) | 1.6772656 |
| Nominal p-value | 0.0 |
| FDR q-value | 0.012474808 |
| FWER p-Value | 0.049 |
Table: GSEA Results Summary

  

Fig 1: Enrichment plot: HALLMARK\_FATTY\_ACID\_METABOLISM      
 Profile of the Running ES Score & Positions of GeneSet Members on the Rank Ordered List

  

| PROBE | GENE SYMBOL | GENE\_TITLE | RANK IN GENE LIST | RANK METRIC SCORE | RUNNING ES | CORE ENRICHMENT || 1 | LGALS1 |  |  | 4 | 0.828 | 0.0693 | Yes |
| 2 | ODC1 |  |  | 7 | 0.744 | 0.1318 | Yes |
| 3 | SMS |  |  | 23 | 0.619 | 0.1824 | Yes |
| 4 | S100A10 |  |  | 33 | 0.540 | 0.2269 | Yes |
| 5 | PRDX6 |  |  | 79 | 0.398 | 0.2558 | Yes |
| 6 | LDHA |  |  | 80 | 0.394 | 0.2889 | Yes |
| 7 | ECHS1 |  |  | 283 | 0.266 | 0.2906 | Yes |
| 8 | PCBD1 |  |  | 302 | 0.260 | 0.3107 | Yes |
| 9 | MDH2 |  |  | 333 | 0.248 | 0.3285 | Yes |
| 10 | YWHAH |  |  | 365 | 0.237 | 0.3453 | Yes |
| 11 | ACAT2 |  |  | 385 | 0.229 | 0.3626 | Yes |
| 12 | PTS |  |  | 390 | 0.228 | 0.3814 | Yes |
| 13 | PSME1 |  |  | 519 | 0.201 | 0.3853 | Yes |
| 14 | MIF |  |  | 524 | 0.200 | 0.4017 | Yes |
| 15 | MGLL |  |  | 633 | 0.181 | 0.4059 | Yes |
| 16 | KMT5A |  |  | 752 | 0.165 | 0.4077 | Yes |
| 17 | CBR1 |  |  | 792 | 0.158 | 0.4170 | Yes |
| 18 | G0S2 |  |  | 903 | 0.145 | 0.4179 | Yes |
| 19 | ALDH3A2 |  |  | 913 | 0.143 | 0.4291 | Yes |
| 20 | CBR3 |  |  | 954 | 0.139 | 0.4367 | Yes |
| 21 | ACSL1 |  |  | 962 | 0.138 | 0.4476 | Yes |
| 22 | BLVRA |  |  | 1204 | 0.112 | 0.4324 | Yes |
| 23 | HCCS |  |  | 1210 | 0.111 | 0.4412 | Yes |
| 24 | PDHB |  |  | 1277 | 0.106 | 0.4434 | Yes |
| 25 | ACSL4 |  |  | 1293 | 0.104 | 0.4506 | Yes |
| 26 | PDHA1 |  |  | 1370 | 0.098 | 0.4511 | Yes |
| 27 | ACAA2 |  |  | 1425 | 0.094 | 0.4535 | Yes |
| 28 | FH |  |  | 1455 | 0.092 | 0.4582 | Yes |
| 29 | CRAT |  |  | 1515 | 0.088 | 0.4596 | Yes |
| 30 | ECI1 |  |  | 1698 | 0.076 | 0.4473 | No |
| 31 | IDH3B |  |  | 1707 | 0.075 | 0.4528 | No |
| 32 | SUCLG1 |  |  | 1795 | 0.070 | 0.4498 | No |
| 33 | UBE2L6 |  |  | 1852 | 0.067 | 0.4497 | No |
| 34 | HSD17B10 |  |  | 1922 | 0.064 | 0.4480 | No |
| 35 | ALDOA |  |  | 1936 | 0.063 | 0.4520 | No |
| 36 | ECI2 |  |  | 2031 | 0.058 | 0.4473 | No |
| 37 | SDHD |  |  | 2039 | 0.058 | 0.4514 | No |
| 38 | GLUL |  |  | 2130 | 0.054 | 0.4467 | No |
| 39 | MDH1 |  |  | 2144 | 0.053 | 0.4499 | No |
| 40 | UROD |  |  | 2183 | 0.052 | 0.4503 | No |
| 41 | ECH1 |  |  | 2355 | 0.045 | 0.4366 | No |
| 42 | SUCLA2 |  |  | 2377 | 0.044 | 0.4381 | No |
| 43 | GRHPR |  |  | 2414 | 0.043 | 0.4381 | No |
| 44 | HSPH1 |  |  | 2441 | 0.042 | 0.4389 | No |
| 45 | SDHA |  |  | 2540 | 0.038 | 0.4321 | No |
| 46 | HSP90AA1 |  |  | 2562 | 0.038 | 0.4331 | No |
| 47 | NCAPH2 |  |  | 2656 | 0.034 | 0.4265 | No |
| 48 | CPOX |  |  | 2971 | 0.026 | 0.3965 | No |
| 49 | DLD |  |  | 2976 | 0.025 | 0.3982 | No |
| 50 | RAP1GDS1 |  |  | 3001 | 0.025 | 0.3979 | No |
| 51 | APEX1 |  |  | 3102 | 0.022 | 0.3895 | No |
| 52 | CRYZ |  |  | 3105 | 0.022 | 0.3912 | No |
| 53 | ENO2 |  |  | 3161 | 0.021 | 0.3874 | No |
| 54 | METAP1 |  |  | 3194 | 0.020 | 0.3858 | No |
| 55 | IDH3G |  |  | 3238 | 0.020 | 0.3831 | No |
| 56 | UROS |  |  | 3264 | 0.019 | 0.3821 | No |
| 57 | HMGCL |  |  | 3267 | 0.019 | 0.3835 | No |
| 58 | GABARAPL1 |  |  | 3328 | 0.018 | 0.3788 | No |
| 59 | AUH |  |  | 3332 | 0.018 | 0.3800 | No |
| 60 | NTHL1 |  |  | 3386 | 0.016 | 0.3759 | No |
| 61 | ADSL |  |  | 3403 | 0.016 | 0.3756 | No |
| 62 | CCDC58 |  |  | 3472 | 0.014 | 0.3699 | No |
| 63 | ACOT8 |  |  | 3495 | 0.014 | 0.3688 | No |
| 64 | HADH |  |  | 3732 | 0.009 | 0.3454 | No |
| 65 | ACAA1 |  |  | 3782 | 0.008 | 0.3411 | No |
| 66 | SUCLG2 |  |  | 3841 | 0.007 | 0.3358 | No |
| 67 | BCKDHB |  |  | 3913 | 0.006 | 0.3290 | No |
| 68 | UGDH |  |  | 4106 | 0.002 | 0.3096 | No |
| 69 | DLST |  |  | 4280 | -0.001 | 0.2919 | No |
| 70 | ALAD |  |  | 4291 | -0.001 | 0.2910 | No |
| 71 | ACADVL |  |  | 4425 | -0.003 | 0.2777 | No |
| 72 | AADAT |  |  | 4446 | -0.004 | 0.2759 | No |
| 73 | MCEE |  |  | 4901 | -0.011 | 0.2304 | No |
| 74 | ADIPOR2 |  |  | 4936 | -0.012 | 0.2279 | No |
| 75 | PPARA |  |  | 5030 | -0.013 | 0.2194 | No |
| 76 | EPHX1 |  |  | 5080 | -0.014 | 0.2156 | No |
| 77 | DECR1 |  |  | 5098 | -0.014 | 0.2150 | No |
| 78 | ALDH9A1 |  |  | 5173 | -0.015 | 0.2087 | No |
| 79 | SDHC |  |  | 5201 | -0.015 | 0.2072 | No |
| 80 | CPT2 |  |  | 5269 | -0.016 | 0.2018 | No |
| 81 | NSDHL |  |  | 5391 | -0.018 | 0.1909 | No |
| 82 | ACO2 |  |  | 5411 | -0.019 | 0.1905 | No |
| 83 | HSD17B7 |  |  | 5466 | -0.020 | 0.1867 | No |
| 84 | DHCR24 |  |  | 5843 | -0.026 | 0.1504 | No |
| 85 | ACADM |  |  | 5968 | -0.029 | 0.1401 | No |
| 86 | IDI1 |  |  | 6154 | -0.032 | 0.1238 | No |
| 87 | NBN |  |  | 6228 | -0.034 | 0.1192 | No |
| 88 | D2HGDH |  |  | 6653 | -0.042 | 0.0793 | No |
| 89 | GPD2 |  |  | 6736 | -0.044 | 0.0746 | No |
| 90 | RETSAT |  |  | 6959 | -0.049 | 0.0560 | No |
| 91 | OSTC |  |  | 7112 | -0.053 | 0.0449 | No |
| 92 | ETFDH |  |  | 7288 | -0.058 | 0.0319 | No |
| 93 | GSTZ1 |  |  | 7315 | -0.058 | 0.0341 | No |
| 94 | BPHL |  |  | 7344 | -0.059 | 0.0362 | No |
| 95 | GCDH |  |  | 7477 | -0.062 | 0.0280 | No |
| 96 | ACOX1 |  |  | 7691 | -0.069 | 0.0120 | No |
| 97 | MLYCD |  |  | 8016 | -0.082 | -0.0143 | No |
| 98 | ELOVL5 |  |  | 8115 | -0.086 | -0.0171 | No |
| 99 | HSDL2 |  |  | 8225 | -0.090 | -0.0206 | No |
| 100 | CA2 |  |  | 8636 | -0.113 | -0.0530 | No |
| 101 | FASN |  |  | 8694 | -0.117 | -0.0490 | No |
| 102 | HADHB |  |  | 8718 | -0.119 | -0.0414 | No |
| 103 | HSD17B11 |  |  | 8769 | -0.123 | -0.0361 | No |
| 104 | ERP29 |  |  | 8915 | -0.135 | -0.0396 | No |
| 105 | HIBCH |  |  | 9087 | -0.151 | -0.0444 | No |
| 106 | HSD17B4 |  |  | 9277 | -0.176 | -0.0489 | No |
| 107 | PTPRG |  |  | 9370 | -0.194 | -0.0420 | No |
| 108 | RDH11 |  |  | 9422 | -0.205 | -0.0299 | No |
| 109 | SERINC1 |  |  | 9530 | -0.233 | -0.0212 | No |
| 110 | HMGCS1 |  |  | 9718 | -0.329 | -0.0127 | No |
| 111 | IDH1 |  |  | 9737 | -0.345 | 0.0145 | No |
Table: GSEA details [plain text format]

  

Fig 2: HALLMARK\_FATTY\_ACID\_METABOLISM: Random ES distribution      
 Gene set null distribution of ES for **HALLMARK\_FATTY\_ACID\_METABOLISM**

  
