# Supplementary material for: Modeling glioblastoma heterogeneity as a dynamic network of cell states
Source: Mol Syst Biol. 2021 Sep 16;17(9):e10105. doi: 10.15252/msb.202010105 (PMC8444284; doi:10.15252/msb.202010105)
Supplement: Supplementary file 5 — Source Data for Figure 3 [file MSB-17-e10105-s001.zip › Figure3A_sourcedata/GSEA_3065/hallmarks_state1.GseaPreranked.1623416262439/HALLMARK_G2M_CHECKPOINT.html]

Details for gene set HALLMARK\_G2M\_CHECKPOINT[GSEA]

|  || Dataset | state1 |
| Phenotype | NoPhenotypeAvailable |
| Upregulated in class | na\_pos |
| GeneSet | HALLMARK\_G2M\_CHECKPOINT |
| Enrichment Score (ES) | 0.40333852 |
| Normalized Enrichment Score (NES) | 1.5821322 |
| Nominal p-value | 0.0 |
| FDR q-value | 0.019189669 |
| FWER p-Value | 0.123 |
Table: GSEA Results Summary

  

Fig 1: Enrichment plot: HALLMARK\_G2M\_CHECKPOINT      
 Profile of the Running ES Score & Positions of GeneSet Members on the Rank Ordered List

  

| PROBE | GENE SYMBOL | GENE\_TITLE | RANK IN GENE LIST | RANK METRIC SCORE | RUNNING ES | CORE ENRICHMENT || 1 | ODC1 |  |  | 7 | 0.744 | 0.0427 | Yes |
| 2 | HMGA1 |  |  | 8 | 0.736 | 0.0857 | Yes |
| 3 | UBE2S |  |  | 19 | 0.634 | 0.1216 | Yes |
| 4 | CCND1 |  |  | 29 | 0.566 | 0.1537 | Yes |
| 5 | PTTG1 |  |  | 40 | 0.520 | 0.1830 | Yes |
| 6 | UBE2C |  |  | 125 | 0.340 | 0.1942 | Yes |
| 7 | MT2A |  |  | 161 | 0.317 | 0.2091 | Yes |
| 8 | HMGN2 |  |  | 164 | 0.313 | 0.2271 | Yes |
| 9 | CDKN3 |  |  | 178 | 0.307 | 0.2437 | Yes |
| 10 | CKS2 |  |  | 195 | 0.297 | 0.2594 | Yes |
| 11 | BIRC5 |  |  | 202 | 0.295 | 0.2760 | Yes |
| 12 | DTYMK |  |  | 205 | 0.292 | 0.2928 | Yes |
| 13 | JPT1 |  |  | 248 | 0.277 | 0.3046 | Yes |
| 14 | CKS1B |  |  | 346 | 0.242 | 0.3087 | Yes |
| 15 | CDC20 |  |  | 469 | 0.212 | 0.3085 | Yes |
| 16 | AMD1 |  |  | 482 | 0.210 | 0.3195 | Yes |
| 17 | AURKA |  |  | 550 | 0.196 | 0.3240 | Yes |
| 18 | TPX2 |  |  | 556 | 0.194 | 0.3348 | Yes |
| 19 | G3BP1 |  |  | 611 | 0.184 | 0.3400 | Yes |
| 20 | SNRPD1 |  |  | 647 | 0.179 | 0.3469 | Yes |
| 21 | KIF5B |  |  | 678 | 0.175 | 0.3540 | Yes |
| 22 | KMT5A |  |  | 752 | 0.165 | 0.3561 | Yes |
| 23 | STMN1 |  |  | 763 | 0.163 | 0.3646 | Yes |
| 24 | HMGB3 |  |  | 907 | 0.144 | 0.3582 | Yes |
| 25 | TROAP |  |  | 1134 | 0.119 | 0.3419 | Yes |
| 26 | MEIS2 |  |  | 1230 | 0.110 | 0.3385 | Yes |
| 27 | CCNB2 |  |  | 1232 | 0.110 | 0.3448 | Yes |
| 28 | DDX39A |  |  | 1255 | 0.108 | 0.3488 | Yes |
| 29 | KIF4A |  |  | 1303 | 0.103 | 0.3499 | Yes |
| 30 | SLC7A5 |  |  | 1312 | 0.102 | 0.3551 | Yes |
| 31 | TFDP1 |  |  | 1318 | 0.102 | 0.3605 | Yes |
| 32 | ATF5 |  |  | 1326 | 0.101 | 0.3657 | Yes |
| 33 | HUS1 |  |  | 1388 | 0.096 | 0.3650 | Yes |
| 34 | MAD2L1 |  |  | 1394 | 0.095 | 0.3700 | Yes |
| 35 | PLK1 |  |  | 1403 | 0.095 | 0.3748 | Yes |
| 36 | HMMR |  |  | 1480 | 0.090 | 0.3722 | Yes |
| 37 | HNRNPD |  |  | 1509 | 0.088 | 0.3744 | Yes |
| 38 | NCL |  |  | 1512 | 0.088 | 0.3794 | Yes |
| 39 | CBX1 |  |  | 1521 | 0.088 | 0.3837 | Yes |
| 40 | CENPE |  |  | 1545 | 0.086 | 0.3863 | Yes |
| 41 | KIF23 |  |  | 1638 | 0.080 | 0.3815 | Yes |
| 42 | PBK |  |  | 1651 | 0.079 | 0.3848 | Yes |
| 43 | CDC27 |  |  | 1688 | 0.076 | 0.3856 | Yes |
| 44 | NUSAP1 |  |  | 1736 | 0.073 | 0.3850 | Yes |
| 45 | CENPA |  |  | 1788 | 0.070 | 0.3838 | Yes |
| 46 | PRC1 |  |  | 1794 | 0.070 | 0.3874 | Yes |
| 47 | GSPT1 |  |  | 1797 | 0.070 | 0.3913 | Yes |
| 48 | SYNCRIP |  |  | 1817 | 0.069 | 0.3933 | Yes |
| 49 | CCNA2 |  |  | 1835 | 0.068 | 0.3955 | Yes |
| 50 | KIF22 |  |  | 1858 | 0.067 | 0.3972 | Yes |
| 51 | PML |  |  | 1879 | 0.066 | 0.3990 | Yes |
| 52 | TACC3 |  |  | 1949 | 0.063 | 0.3955 | Yes |
| 53 | RACGAP1 |  |  | 1994 | 0.060 | 0.3945 | Yes |
| 54 | CDK4 |  |  | 2000 | 0.060 | 0.3974 | Yes |
| 55 | SAP30 |  |  | 2022 | 0.059 | 0.3987 | Yes |
| 56 | TGFB1 |  |  | 2040 | 0.058 | 0.4003 | Yes |
| 57 | DKC1 |  |  | 2044 | 0.057 | 0.4033 | Yes |
| 58 | KPNB1 |  |  | 2179 | 0.052 | 0.3925 | No |
| 59 | MNAT1 |  |  | 2209 | 0.051 | 0.3925 | No |
| 60 | KIF2C |  |  | 2224 | 0.050 | 0.3940 | No |
| 61 | CHMP1A |  |  | 2240 | 0.050 | 0.3953 | No |
| 62 | SMC4 |  |  | 2308 | 0.047 | 0.3912 | No |
| 63 | UCK2 |  |  | 2321 | 0.046 | 0.3926 | No |
| 64 | CDC25B |  |  | 2370 | 0.044 | 0.3902 | No |
| 65 | RPA2 |  |  | 2385 | 0.044 | 0.3913 | No |
| 66 | NOLC1 |  |  | 2417 | 0.043 | 0.3906 | No |
| 67 | MYBL2 |  |  | 2549 | 0.038 | 0.3793 | No |
| 68 | E2F4 |  |  | 2554 | 0.038 | 0.3812 | No |
| 69 | GINS2 |  |  | 2567 | 0.038 | 0.3821 | No |
| 70 | RAD23B |  |  | 2654 | 0.034 | 0.3752 | No |
| 71 | DBF4 |  |  | 2751 | 0.031 | 0.3672 | No |
| 72 | MKI67 |  |  | 2760 | 0.031 | 0.3682 | No |
| 73 | NEK2 |  |  | 2834 | 0.030 | 0.3624 | No |
| 74 | FBXO5 |  |  | 2870 | 0.028 | 0.3604 | No |
| 75 | BUB1 |  |  | 2888 | 0.028 | 0.3603 | No |
| 76 | DR1 |  |  | 2900 | 0.027 | 0.3608 | No |
| 77 | TMPO |  |  | 3093 | 0.023 | 0.3423 | No |
| 78 | PAFAH1B1 |  |  | 3141 | 0.022 | 0.3387 | No |
| 79 | ODF2 |  |  | 3157 | 0.021 | 0.3384 | No |
| 80 | HOXC10 |  |  | 3175 | 0.021 | 0.3378 | No |
| 81 | CUL1 |  |  | 3336 | 0.017 | 0.3223 | No |
| 82 | CDK1 |  |  | 3396 | 0.016 | 0.3172 | No |
| 83 | BCL3 |  |  | 3622 | 0.011 | 0.2946 | No |
| 84 | MAPK14 |  |  | 3651 | 0.011 | 0.2924 | No |
| 85 | TTK |  |  | 3724 | 0.009 | 0.2855 | No |
| 86 | SRSF2 |  |  | 3783 | 0.008 | 0.2800 | No |
| 87 | TOP1 |  |  | 3789 | 0.008 | 0.2800 | No |
| 88 | HIF1A |  |  | 3955 | 0.005 | 0.2632 | No |
| 89 | MYC |  |  | 3964 | 0.005 | 0.2627 | No |
| 90 | DMD |  |  | 4140 | 0.002 | 0.2448 | No |
| 91 | EWSR1 |  |  | 4279 | -0.001 | 0.2306 | No |
| 92 | HSPA8 |  |  | 4292 | -0.001 | 0.2294 | No |
| 93 | CUL5 |  |  | 4373 | -0.002 | 0.2213 | No |
| 94 | PRMT5 |  |  | 4376 | -0.002 | 0.2212 | No |
| 95 | TRAIP |  |  | 4405 | -0.003 | 0.2185 | No |
| 96 | POLA2 |  |  | 4497 | -0.004 | 0.2094 | No |
| 97 | MCM3 |  |  | 4633 | -0.007 | 0.1958 | No |
| 98 | CDKN1B |  |  | 4655 | -0.007 | 0.1941 | No |
| 99 | ORC5 |  |  | 4744 | -0.008 | 0.1855 | No |
| 100 | AURKB |  |  | 4764 | -0.008 | 0.1840 | No |
| 101 | RBM14 |  |  | 4801 | -0.009 | 0.1808 | No |
| 102 | CUL4A |  |  | 4808 | -0.009 | 0.1807 | No |
| 103 | SLC7A1 |  |  | 4822 | -0.009 | 0.1799 | No |
| 104 | BUB3 |  |  | 4828 | -0.010 | 0.1799 | No |
| 105 | INCENP |  |  | 4861 | -0.010 | 0.1772 | No |
| 106 | CCNF |  |  | 4868 | -0.010 | 0.1772 | No |
| 107 | CUL3 |  |  | 4869 | -0.010 | 0.1778 | No |
| 108 | RAD21 |  |  | 4916 | -0.011 | 0.1737 | No |
| 109 | SLC12A2 |  |  | 4938 | -0.012 | 0.1723 | No |
| 110 | CHAF1A |  |  | 4943 | -0.012 | 0.1725 | No |
| 111 | KATNA1 |  |  | 4983 | -0.012 | 0.1692 | No |
| 112 | HIRA |  |  | 5045 | -0.013 | 0.1637 | No |
| 113 | KIF20B |  |  | 5169 | -0.015 | 0.1519 | No |
| 114 | LIG3 |  |  | 5180 | -0.015 | 0.1517 | No |
| 115 | TRA2B |  |  | 5321 | -0.017 | 0.1383 | No |
| 116 | CHEK1 |  |  | 5330 | -0.017 | 0.1385 | No |
| 117 | STIL |  |  | 5357 | -0.018 | 0.1368 | No |
| 118 | PLK4 |  |  | 5414 | -0.019 | 0.1321 | No |
| 119 | SS18 |  |  | 5429 | -0.019 | 0.1318 | No |
| 120 | KPNA2 |  |  | 5461 | -0.020 | 0.1297 | No |
| 121 | MCM5 |  |  | 5588 | -0.022 | 0.1180 | No |
| 122 | NUP98 |  |  | 5623 | -0.022 | 0.1158 | No |
| 123 | YTHDC1 |  |  | 5681 | -0.023 | 0.1113 | No |
| 124 | RASAL2 |  |  | 5684 | -0.023 | 0.1124 | No |
| 125 | CDC25A |  |  | 5789 | -0.025 | 0.1032 | No |
| 126 | NUP50 |  |  | 5803 | -0.025 | 0.1033 | No |
| 127 | SFPQ |  |  | 6115 | -0.031 | 0.0730 | No |
| 128 | PRIM2 |  |  | 6118 | -0.031 | 0.0746 | No |
| 129 | SMAD3 |  |  | 6222 | -0.033 | 0.0660 | No |
| 130 | CTCF |  |  | 6233 | -0.034 | 0.0669 | No |
| 131 | NDC80 |  |  | 6240 | -0.034 | 0.0682 | No |
| 132 | E2F1 |  |  | 6247 | -0.034 | 0.0696 | No |
| 133 | MCM2 |  |  | 6282 | -0.034 | 0.0681 | No |
| 134 | ABL1 |  |  | 6318 | -0.035 | 0.0665 | No |
| 135 | KIF11 |  |  | 6477 | -0.038 | 0.0524 | No |
| 136 | KNL1 |  |  | 6527 | -0.039 | 0.0497 | No |
| 137 | FANCC |  |  | 6538 | -0.039 | 0.0509 | No |
| 138 | UPF1 |  |  | 6693 | -0.043 | 0.0376 | No |
| 139 | CDC7 |  |  | 6788 | -0.045 | 0.0305 | No |
| 140 | STAG1 |  |  | 6838 | -0.046 | 0.0281 | No |
| 141 | POLE |  |  | 6848 | -0.046 | 0.0298 | No |
| 142 | SRSF1 |  |  | 6896 | -0.047 | 0.0277 | No |
| 143 | CENPF |  |  | 6902 | -0.047 | 0.0300 | No |
| 144 | TENT4A |  |  | 6958 | -0.049 | 0.0272 | No |
| 145 | SMARCC1 |  |  | 6990 | -0.050 | 0.0269 | No |
| 146 | FOXN3 |  |  | 7031 | -0.051 | 0.0257 | No |
| 147 | E2F3 |  |  | 7064 | -0.052 | 0.0254 | No |
| 148 | PRPF4B |  |  | 7096 | -0.053 | 0.0253 | No |
| 149 | MAP3K20 |  |  | 7115 | -0.054 | 0.0266 | No |
| 150 | CDC6 |  |  | 7140 | -0.054 | 0.0273 | No |
| 151 | LMNB1 |  |  | 7166 | -0.055 | 0.0279 | No |
| 152 | EZH2 |  |  | 7260 | -0.057 | 0.0216 | No |
| 153 | MCM6 |  |  | 7414 | -0.061 | 0.0094 | No |
| 154 | SRSF10 |  |  | 7557 | -0.065 | -0.0015 | No |
| 155 | CCNT1 |  |  | 7646 | -0.068 | -0.0066 | No |
| 156 | CDKN2C |  |  | 7660 | -0.068 | -0.0040 | No |
| 157 | TOP2A |  |  | 7678 | -0.069 | -0.0017 | No |
| 158 | WRN |  |  | 7731 | -0.071 | -0.0029 | No |
| 159 | CDC45 |  |  | 7751 | -0.072 | -0.0007 | No |
| 160 | MEIS1 |  |  | 7794 | -0.073 | -0.0008 | No |
| 161 | ILF3 |  |  | 7823 | -0.074 | 0.0007 | No |
| 162 | XPO1 |  |  | 7863 | -0.076 | 0.0011 | No |
| 163 | PURA |  |  | 7893 | -0.077 | 0.0026 | No |
| 164 | BARD1 |  |  | 7980 | -0.080 | -0.0016 | No |
| 165 | POLQ |  |  | 8022 | -0.082 | -0.0010 | No |
| 166 | SLC38A1 |  |  | 8034 | -0.083 | 0.0026 | No |
| 167 | RBL1 |  |  | 8134 | -0.087 | -0.0025 | No |
| 168 | TLE3 |  |  | 8155 | -0.087 | 0.0005 | No |
| 169 | BRCA2 |  |  | 8192 | -0.089 | 0.0020 | No |
| 170 | NUMA1 |  |  | 8218 | -0.090 | 0.0047 | No |
| 171 | TNPO2 |  |  | 8235 | -0.091 | 0.0084 | No |
| 172 | CASP8AP2 |  |  | 8275 | -0.093 | 0.0098 | No |
| 173 | PDS5B |  |  | 8300 | -0.094 | 0.0128 | No |
| 174 | NSD2 |  |  | 8351 | -0.097 | 0.0133 | No |
| 175 | KIF15 |  |  | 8542 | -0.108 | -0.0000 | No |
| 176 | MTF2 |  |  | 8595 | -0.111 | 0.0011 | No |
| 177 | HNRNPU |  |  | 8616 | -0.112 | 0.0056 | No |
| 178 | SMC1A |  |  | 8681 | -0.116 | 0.0058 | No |
| 179 | SMC2 |  |  | 8721 | -0.119 | 0.0087 | No |
| 180 | ARID4A |  |  | 8864 | -0.130 | 0.0016 | No |
| 181 | ORC6 |  |  | 8888 | -0.133 | 0.0070 | No |
| 182 | LBR |  |  | 9264 | -0.174 | -0.0215 | No |
| 183 | NOTCH2 |  |  | 9279 | -0.177 | -0.0126 | No |
| 184 | NASP |  |  | 9437 | -0.208 | -0.0167 | No |
| 185 | ATRX |  |  | 9654 | -0.284 | -0.0224 | No |
| 186 | MARCKS |  |  | 9763 | -0.370 | -0.0119 | No |
| 187 | SQLE |  |  | 9790 | -0.408 | 0.0092 | No |
Table: GSEA details [plain text format]

  

Fig 2: HALLMARK\_G2M\_CHECKPOINT: Random ES distribution      
 Gene set null distribution of ES for **HALLMARK\_G2M\_CHECKPOINT**

  
