# Supplementary material for: Modeling glioblastoma heterogeneity as a dynamic network of cell states
Source: Mol Syst Biol. 2021 Sep 16;17(9):e10105. doi: 10.15252/msb.202010105 (PMC8444284; doi:10.15252/msb.202010105)
Supplement: Supplementary file 5 — Source Data for Figure 3 [file MSB-17-e10105-s001.zip › Figure3A_sourcedata/GSEA_3065/hallmarks_state1.GseaPreranked.1623416262439/HALLMARK_GLYCOLYSIS.html]

Details for gene set HALLMARK\_GLYCOLYSIS[GSEA]

|  || Dataset | state1 |
| Phenotype | NoPhenotypeAvailable |
| Upregulated in class | na\_pos |
| GeneSet | HALLMARK\_GLYCOLYSIS |
| Enrichment Score (ES) | 0.39229155 |
| Normalized Enrichment Score (NES) | 1.5023302 |
| Nominal p-value | 0.002016129 |
| FDR q-value | 0.033182338 |
| FWER p-Value | 0.236 |
Table: GSEA Results Summary

  

Fig 1: Enrichment plot: HALLMARK\_GLYCOLYSIS      
 Profile of the Running ES Score & Positions of GeneSet Members on the Rank Ordered List

  

| PROBE | GENE SYMBOL | GENE\_TITLE | RANK IN GENE LIST | RANK METRIC SCORE | RUNNING ES | CORE ENRICHMENT || 1 | TXN |  |  | 13 | 0.686 | 0.0364 | Yes |
| 2 | PKM |  |  | 14 | 0.676 | 0.0736 | Yes |
| 3 | IGFBP3 |  |  | 15 | 0.664 | 0.1101 | Yes |
| 4 | TPI1 |  |  | 52 | 0.473 | 0.1324 | Yes |
| 5 | LDHA |  |  | 80 | 0.394 | 0.1513 | Yes |
| 6 | PYGB |  |  | 84 | 0.388 | 0.1723 | Yes |
| 7 | ENO1 |  |  | 99 | 0.373 | 0.1914 | Yes |
| 8 | PPIA |  |  | 150 | 0.324 | 0.2041 | Yes |
| 9 | G6PD |  |  | 171 | 0.310 | 0.2190 | Yes |
| 10 | CHST2 |  |  | 182 | 0.305 | 0.2347 | Yes |
| 11 | SOD1 |  |  | 186 | 0.303 | 0.2511 | Yes |
| 12 | PGAM1 |  |  | 190 | 0.302 | 0.2674 | Yes |
| 13 | TPBG |  |  | 315 | 0.256 | 0.2688 | Yes |
| 14 | RBCK1 |  |  | 329 | 0.250 | 0.2812 | Yes |
| 15 | MDH2 |  |  | 333 | 0.248 | 0.2945 | Yes |
| 16 | MET |  |  | 451 | 0.215 | 0.2943 | Yes |
| 17 | NT5E |  |  | 462 | 0.213 | 0.3050 | Yes |
| 18 | MIF |  |  | 524 | 0.200 | 0.3097 | Yes |
| 19 | CXCR4 |  |  | 532 | 0.199 | 0.3200 | Yes |
| 20 | FKBP4 |  |  | 547 | 0.196 | 0.3293 | Yes |
| 21 | AURKA |  |  | 550 | 0.196 | 0.3399 | Yes |
| 22 | PFKP |  |  | 594 | 0.187 | 0.3457 | Yes |
| 23 | CITED2 |  |  | 683 | 0.175 | 0.3463 | Yes |
| 24 | STMN1 |  |  | 763 | 0.163 | 0.3471 | Yes |
| 25 | B4GALT1 |  |  | 802 | 0.157 | 0.3518 | Yes |
| 26 | PSMC4 |  |  | 803 | 0.157 | 0.3605 | Yes |
| 27 | HAX1 |  |  | 808 | 0.156 | 0.3686 | Yes |
| 28 | PGK1 |  |  | 875 | 0.148 | 0.3700 | Yes |
| 29 | STC2 |  |  | 922 | 0.142 | 0.3731 | Yes |
| 30 | GLRX |  |  | 927 | 0.142 | 0.3805 | Yes |
| 31 | POLR3K |  |  | 952 | 0.140 | 0.3857 | Yes |
| 32 | HDLBP |  |  | 988 | 0.135 | 0.3895 | Yes |
| 33 | SDC2 |  |  | 1049 | 0.128 | 0.3904 | Yes |
| 34 | TALDO1 |  |  | 1097 | 0.123 | 0.3923 | Yes |
| 35 | GOT1 |  |  | 1344 | 0.100 | 0.3725 | No |
| 36 | EGFR |  |  | 1474 | 0.091 | 0.3642 | No |
| 37 | DEPDC1 |  |  | 1478 | 0.090 | 0.3689 | No |
| 38 | HMMR |  |  | 1480 | 0.090 | 0.3737 | No |
| 39 | SDC1 |  |  | 1482 | 0.090 | 0.3786 | No |
| 40 | ISG20 |  |  | 1538 | 0.087 | 0.3777 | No |
| 41 | CASP6 |  |  | 1564 | 0.085 | 0.3798 | No |
| 42 | CENPA |  |  | 1788 | 0.070 | 0.3608 | No |
| 43 | FAM162A |  |  | 1805 | 0.069 | 0.3629 | No |
| 44 | PGLS |  |  | 1807 | 0.069 | 0.3666 | No |
| 45 | NDUFV3 |  |  | 1847 | 0.067 | 0.3663 | No |
| 46 | ADORA2B |  |  | 1891 | 0.066 | 0.3655 | No |
| 47 | P4HA2 |  |  | 1910 | 0.065 | 0.3672 | No |
| 48 | ALDOA |  |  | 1936 | 0.063 | 0.3681 | No |
| 49 | GMPPA |  |  | 1978 | 0.061 | 0.3672 | No |
| 50 | SAP30 |  |  | 2022 | 0.059 | 0.3660 | No |
| 51 | TGFA |  |  | 2063 | 0.057 | 0.3650 | No |
| 52 | NOL3 |  |  | 2087 | 0.055 | 0.3657 | No |
| 53 | MDH1 |  |  | 2144 | 0.053 | 0.3629 | No |
| 54 | AK3 |  |  | 2163 | 0.052 | 0.3639 | No |
| 55 | CD44 |  |  | 2193 | 0.051 | 0.3637 | No |
| 56 | PPP2CB |  |  | 2344 | 0.045 | 0.3508 | No |
| 57 | B4GALT7 |  |  | 2465 | 0.041 | 0.3407 | No |
| 58 | GOT2 |  |  | 2496 | 0.040 | 0.3398 | No |
| 59 | SLC25A10 |  |  | 2722 | 0.032 | 0.3185 | No |
| 60 | GMPPB |  |  | 2813 | 0.030 | 0.3109 | No |
| 61 | GALE |  |  | 2848 | 0.029 | 0.3090 | No |
| 62 | KIF20A |  |  | 2897 | 0.028 | 0.3056 | No |
| 63 | AKR1A1 |  |  | 2959 | 0.026 | 0.3007 | No |
| 64 | DLD |  |  | 2976 | 0.025 | 0.3005 | No |
| 65 | KDELR3 |  |  | 3057 | 0.023 | 0.2935 | No |
| 66 | B4GALT2 |  |  | 3075 | 0.023 | 0.2930 | No |
| 67 | ENO2 |  |  | 3161 | 0.021 | 0.2855 | No |
| 68 | GFPT1 |  |  | 3163 | 0.021 | 0.2865 | No |
| 69 | RRAGD |  |  | 3229 | 0.020 | 0.2809 | No |
| 70 | PMM2 |  |  | 3349 | 0.017 | 0.2696 | No |
| 71 | PRPS1 |  |  | 3388 | 0.016 | 0.2666 | No |
| 72 | CDK1 |  |  | 3396 | 0.016 | 0.2668 | No |
| 73 | CYB5A |  |  | 3529 | 0.013 | 0.2539 | No |
| 74 | TGFBI |  |  | 3688 | 0.010 | 0.2383 | No |
| 75 | GNPDA1 |  |  | 4281 | -0.001 | 0.1774 | No |
| 76 | GNE |  |  | 4298 | -0.001 | 0.1759 | No |
| 77 | GYS1 |  |  | 4410 | -0.003 | 0.1646 | No |
| 78 | NANP |  |  | 4437 | -0.003 | 0.1621 | No |
| 79 | ECD |  |  | 4567 | -0.006 | 0.1492 | No |
| 80 | PGM2 |  |  | 4604 | -0.006 | 0.1458 | No |
| 81 | MED24 |  |  | 4672 | -0.007 | 0.1393 | No |
| 82 | RPE |  |  | 4769 | -0.008 | 0.1299 | No |
| 83 | GALK1 |  |  | 4872 | -0.010 | 0.1200 | No |
| 84 | MPI |  |  | 4897 | -0.011 | 0.1181 | No |
| 85 | EXT1 |  |  | 5106 | -0.014 | 0.0975 | No |
| 86 | LHPP |  |  | 5114 | -0.014 | 0.0976 | No |
| 87 | ALDH9A1 |  |  | 5173 | -0.015 | 0.0925 | No |
| 88 | SDHC |  |  | 5201 | -0.015 | 0.0905 | No |
| 89 | CHST6 |  |  | 5316 | -0.017 | 0.0798 | No |
| 90 | NSDHL |  |  | 5391 | -0.018 | 0.0731 | No |
| 91 | PDK3 |  |  | 5462 | -0.020 | 0.0670 | No |
| 92 | PHKA2 |  |  | 5478 | -0.020 | 0.0666 | No |
| 93 | GLCE |  |  | 5505 | -0.020 | 0.0650 | No |
| 94 | ERO1A |  |  | 5520 | -0.020 | 0.0647 | No |
| 95 | ARPP19 |  |  | 5555 | -0.021 | 0.0624 | No |
| 96 | GCLC |  |  | 5628 | -0.022 | 0.0562 | No |
| 97 | PLOD1 |  |  | 5662 | -0.023 | 0.0541 | No |
| 98 | COPB2 |  |  | 5695 | -0.023 | 0.0521 | No |
| 99 | KIF2A |  |  | 5712 | -0.024 | 0.0517 | No |
| 100 | BPNT1 |  |  | 5979 | -0.029 | 0.0260 | No |
| 101 | GALK2 |  |  | 6183 | -0.033 | 0.0069 | No |
| 102 | HOMER1 |  |  | 6236 | -0.034 | 0.0034 | No |
| 103 | FUT8 |  |  | 6330 | -0.035 | -0.0042 | No |
| 104 | ALG1 |  |  | 6409 | -0.037 | -0.0103 | No |
| 105 | B3GALT6 |  |  | 6425 | -0.037 | -0.0098 | No |
| 106 | VLDLR |  |  | 6518 | -0.039 | -0.0171 | No |
| 107 | DPYSL4 |  |  | 6529 | -0.039 | -0.0160 | No |
| 108 | DDIT4 |  |  | 6713 | -0.043 | -0.0324 | No |
| 109 | SRD5A3 |  |  | 6752 | -0.044 | -0.0339 | No |
| 110 | B3GAT3 |  |  | 7146 | -0.054 | -0.0713 | No |
| 111 | PAXIP1 |  |  | 7445 | -0.061 | -0.0986 | No |
| 112 | COG2 |  |  | 7488 | -0.063 | -0.0994 | No |
| 113 | ME2 |  |  | 7611 | -0.067 | -0.1083 | No |
| 114 | QSOX1 |  |  | 7628 | -0.067 | -0.1063 | No |
| 115 | COL5A1 |  |  | 7642 | -0.067 | -0.1039 | No |
| 116 | SLC37A4 |  |  | 7693 | -0.069 | -0.1052 | No |
| 117 | STC1 |  |  | 7777 | -0.072 | -0.1098 | No |
| 118 | MXI1 |  |  | 7832 | -0.075 | -0.1112 | No |
| 119 | AGL |  |  | 7897 | -0.077 | -0.1135 | No |
| 120 | CHPF2 |  |  | 7933 | -0.079 | -0.1128 | No |
| 121 | IDUA |  |  | 8039 | -0.083 | -0.1191 | No |
| 122 | IL13RA1 |  |  | 8058 | -0.083 | -0.1163 | No |
| 123 | CLN6 |  |  | 8132 | -0.087 | -0.1191 | No |
| 124 | PYGL |  |  | 8196 | -0.089 | -0.1207 | No |
| 125 | UGP2 |  |  | 8287 | -0.094 | -0.1247 | No |
| 126 | PAM |  |  | 8343 | -0.096 | -0.1251 | No |
| 127 | EXT2 |  |  | 8381 | -0.098 | -0.1235 | No |
| 128 | ANKZF1 |  |  | 8423 | -0.100 | -0.1222 | No |
| 129 | ALDH7A1 |  |  | 8467 | -0.103 | -0.1210 | No |
| 130 | GPC4 |  |  | 8624 | -0.113 | -0.1308 | No |
| 131 | SLC35A3 |  |  | 8644 | -0.114 | -0.1265 | No |
| 132 | B4GALT4 |  |  | 8665 | -0.115 | -0.1222 | No |
| 133 | SLC25A13 |  |  | 8672 | -0.115 | -0.1165 | No |
| 134 | HS2ST1 |  |  | 8696 | -0.117 | -0.1124 | No |
| 135 | P4HA1 |  |  | 8810 | -0.125 | -0.1171 | No |
| 136 | CAPN5 |  |  | 8902 | -0.134 | -0.1191 | No |
| 137 | CHPF |  |  | 8922 | -0.136 | -0.1136 | No |
| 138 | B3GAT1 |  |  | 8986 | -0.142 | -0.1123 | No |
| 139 | VCAN |  |  | 9151 | -0.158 | -0.1204 | No |
| 140 | GUSB |  |  | 9165 | -0.161 | -0.1129 | No |
| 141 | PLOD2 |  |  | 9169 | -0.161 | -0.1044 | No |
| 142 | VEGFA |  |  | 9220 | -0.168 | -0.1002 | No |
| 143 | TPST1 |  |  | 9261 | -0.173 | -0.0948 | No |
| 144 | CHST12 |  |  | 9334 | -0.187 | -0.0919 | No |
| 145 | ZNF292 |  |  | 9395 | -0.200 | -0.0871 | No |
| 146 | NASP |  |  | 9437 | -0.208 | -0.0799 | No |
| 147 | HSPA5 |  |  | 9533 | -0.234 | -0.0768 | No |
| 148 | IRS2 |  |  | 9641 | -0.277 | -0.0726 | No |
| 149 | AGRN |  |  | 9726 | -0.335 | -0.0628 | No |
| 150 | IDH1 |  |  | 9737 | -0.345 | -0.0448 | No |
| 151 | GPC1 |  |  | 9751 | -0.356 | -0.0266 | No |
| 152 | SOX9 |  |  | 9754 | -0.358 | -0.0071 | No |
| 153 | SDC3 |  |  | 9756 | -0.360 | 0.0126 | No |
Table: GSEA details [plain text format]

  

Fig 2: HALLMARK\_GLYCOLYSIS: Random ES distribution      
 Gene set null distribution of ES for **HALLMARK\_GLYCOLYSIS**

  
