# Supplementary material for: Modeling glioblastoma heterogeneity as a dynamic network of cell states
Source: Mol Syst Biol. 2021 Sep 16;17(9):e10105. doi: 10.15252/msb.202010105 (PMC8444284; doi:10.15252/msb.202010105)
Supplement: Supplementary file 5 — Source Data for Figure 3 [file MSB-17-e10105-s001.zip › Figure3A_sourcedata/GSEA_3065/hallmarks_state1.GseaPreranked.1623416262439/HALLMARK_HEME_METABOLISM.html]

Details for gene set HALLMARK\_HEME\_METABOLISM[GSEA]

|  || Dataset | state1 |
| Phenotype | NoPhenotypeAvailable |
| Upregulated in class | na\_neg |
| GeneSet | HALLMARK\_HEME\_METABOLISM |
| Enrichment Score (ES) | -0.23952104 |
| Normalized Enrichment Score (NES) | -0.87213415 |
| Nominal p-value | 0.7865385 |
| FDR q-value | 0.73367155 |
| FWER p-Value | 1.0 |
Table: GSEA Results Summary

  

Fig 1: Enrichment plot: HALLMARK\_HEME\_METABOLISM      
 Profile of the Running ES Score & Positions of GeneSet Members on the Rank Ordered List

  

| PROBE | GENE SYMBOL | GENE\_TITLE | RANK IN GENE LIST | RANK METRIC SCORE | RUNNING ES | CORE ENRICHMENT || 1 | CAST |  |  | 35 | 0.534 | 0.0446 | No |
| 2 | SLC2A1 |  |  | 235 | 0.283 | 0.0497 | No |
| 3 | GLRX5 |  |  | 255 | 0.276 | 0.0726 | No |
| 4 | RAD23A |  |  | 277 | 0.269 | 0.0947 | No |
| 5 | SLC7A11 |  |  | 459 | 0.214 | 0.0955 | No |
| 6 | TCEA1 |  |  | 481 | 0.210 | 0.1123 | No |
| 7 | HDGF |  |  | 512 | 0.203 | 0.1275 | No |
| 8 | GYPC |  |  | 770 | 0.162 | 0.1157 | No |
| 9 | PRDX2 |  |  | 794 | 0.157 | 0.1276 | No |
| 10 | OPTN |  |  | 822 | 0.154 | 0.1388 | No |
| 11 | BLVRB |  |  | 945 | 0.140 | 0.1389 | No |
| 12 | ADIPOR1 |  |  | 998 | 0.134 | 0.1457 | No |
| 13 | TSPAN5 |  |  | 1041 | 0.129 | 0.1530 | No |
| 14 | RBM38 |  |  | 1123 | 0.120 | 0.1555 | No |
| 15 | ISCA1 |  |  | 1158 | 0.117 | 0.1626 | No |
| 16 | BLVRA |  |  | 1204 | 0.112 | 0.1681 | No |
| 17 | GCLM |  |  | 1238 | 0.109 | 0.1746 | No |
| 18 | OSBP2 |  |  | 1345 | 0.099 | 0.1727 | No |
| 19 | CCND3 |  |  | 1389 | 0.096 | 0.1769 | No |
| 20 | HAGH |  |  | 1420 | 0.094 | 0.1824 | No |
| 21 | UBAC1 |  |  | 1587 | 0.083 | 0.1728 | No |
| 22 | MAP2K3 |  |  | 1643 | 0.079 | 0.1744 | No |
| 23 | CDC27 |  |  | 1688 | 0.076 | 0.1767 | No |
| 24 | FBXO9 |  |  | 1789 | 0.070 | 0.1728 | No |
| 25 | PGLS |  |  | 1807 | 0.069 | 0.1773 | No |
| 26 | UCP2 |  |  | 1845 | 0.067 | 0.1796 | No |
| 27 | ARL2BP |  |  | 1853 | 0.067 | 0.1849 | No |
| 28 | SLC25A38 |  |  | 1906 | 0.065 | 0.1854 | No |
| 29 | P4HA2 |  |  | 1910 | 0.065 | 0.1910 | No |
| 30 | ANK1 |  |  | 1911 | 0.065 | 0.1968 | No |
| 31 | HMBS |  |  | 1947 | 0.063 | 0.1989 | No |
| 32 | MFHAS1 |  |  | 2167 | 0.052 | 0.1811 | No |
| 33 | UROD |  |  | 2183 | 0.052 | 0.1843 | No |
| 34 | SLC10A3 |  |  | 2184 | 0.052 | 0.1889 | No |
| 35 | GMPS |  |  | 2197 | 0.051 | 0.1923 | No |
| 36 | PSMD9 |  |  | 2383 | 0.044 | 0.1773 | No |
| 37 | CDR2 |  |  | 2435 | 0.042 | 0.1758 | No |
| 38 | ELL2 |  |  | 2543 | 0.038 | 0.1683 | No |
| 39 | NEK7 |  |  | 2548 | 0.038 | 0.1713 | No |
| 40 | MPP1 |  |  | 2614 | 0.036 | 0.1679 | No |
| 41 | CCDC28A |  |  | 2698 | 0.033 | 0.1623 | No |
| 42 | SEC14L1 |  |  | 2735 | 0.032 | 0.1615 | No |
| 43 | HTATIP2 |  |  | 2934 | 0.027 | 0.1436 | No |
| 44 | MKRN1 |  |  | 2937 | 0.026 | 0.1458 | No |
| 45 | SMOX |  |  | 2956 | 0.026 | 0.1463 | No |
| 46 | CPOX |  |  | 2971 | 0.026 | 0.1471 | No |
| 47 | HTRA2 |  |  | 3132 | 0.022 | 0.1327 | No |
| 48 | UROS |  |  | 3264 | 0.019 | 0.1210 | No |
| 49 | FECH |  |  | 3301 | 0.018 | 0.1189 | No |
| 50 | TRAK2 |  |  | 3428 | 0.015 | 0.1074 | No |
| 51 | ADD2 |  |  | 3565 | 0.013 | 0.0945 | No |
| 52 | ATG4A |  |  | 3648 | 0.011 | 0.0871 | No |
| 53 | TOP1 |  |  | 3789 | 0.008 | 0.0735 | No |
| 54 | YPEL5 |  |  | 3803 | 0.008 | 0.0729 | No |
| 55 | GDE1 |  |  | 4025 | 0.004 | 0.0506 | No |
| 56 | HEBP1 |  |  | 4052 | 0.003 | 0.0482 | No |
| 57 | BMP2K |  |  | 4093 | 0.003 | 0.0443 | No |
| 58 | XPO7 |  |  | 4170 | 0.001 | 0.0366 | No |
| 59 | MGST3 |  |  | 4220 | 0.000 | 0.0316 | No |
| 60 | PPP2R5B |  |  | 4271 | -0.001 | 0.0266 | No |
| 61 | ALAD |  |  | 4291 | -0.001 | 0.0247 | No |
| 62 | NCOA4 |  |  | 4384 | -0.002 | 0.0155 | No |
| 63 | FBXO34 |  |  | 4549 | -0.006 | -0.0008 | No |
| 64 | RNF123 |  |  | 4575 | -0.006 | -0.0029 | No |
| 65 | PPOX |  |  | 4596 | -0.006 | -0.0043 | No |
| 66 | NR3C1 |  |  | 4804 | -0.009 | -0.0248 | No |
| 67 | MOSPD1 |  |  | 4816 | -0.009 | -0.0251 | No |
| 68 | RIOK3 |  |  | 4837 | -0.010 | -0.0262 | No |
| 69 | FOXJ2 |  |  | 4845 | -0.010 | -0.0260 | No |
| 70 | CTSB |  |  | 4873 | -0.011 | -0.0279 | No |
| 71 | CAT |  |  | 4951 | -0.012 | -0.0347 | No |
| 72 | PICALM |  |  | 5039 | -0.013 | -0.0424 | No |
| 73 | BPGM |  |  | 5064 | -0.013 | -0.0437 | No |
| 74 | EIF2AK1 |  |  | 5136 | -0.015 | -0.0496 | No |
| 75 | DCAF11 |  |  | 5182 | -0.015 | -0.0529 | No |
| 76 | ASNS |  |  | 5370 | -0.018 | -0.0705 | No |
| 77 | CIR1 |  |  | 5547 | -0.021 | -0.0866 | No |
| 78 | GCLC |  |  | 5628 | -0.022 | -0.0928 | No |
| 79 | BACH1 |  |  | 5690 | -0.023 | -0.0970 | No |
| 80 | GAPVD1 |  |  | 5766 | -0.025 | -0.1024 | No |
| 81 | RANBP10 |  |  | 6091 | -0.031 | -0.1329 | No |
| 82 | ARHGEF12 |  |  | 6107 | -0.031 | -0.1316 | No |
| 83 | CTNS |  |  | 6235 | -0.034 | -0.1416 | No |
| 84 | USP15 |  |  | 6284 | -0.034 | -0.1434 | No |
| 85 | SYNJ1 |  |  | 6541 | -0.039 | -0.1662 | No |
| 86 | BTRC |  |  | 6608 | -0.041 | -0.1692 | No |
| 87 | TFDP2 |  |  | 6740 | -0.044 | -0.1787 | No |
| 88 | SIDT2 |  |  | 6774 | -0.044 | -0.1781 | No |
| 89 | LRP10 |  |  | 6814 | -0.045 | -0.1780 | No |
| 90 | DCUN1D1 |  |  | 7006 | -0.050 | -0.1931 | No |
| 91 | KLF3 |  |  | 7152 | -0.054 | -0.2030 | No |
| 92 | VEZF1 |  |  | 7182 | -0.055 | -0.2010 | No |
| 93 | DCAF10 |  |  | 7248 | -0.057 | -0.2026 | No |
| 94 | MBOAT2 |  |  | 7285 | -0.058 | -0.2011 | No |
| 95 | EZH1 |  |  | 7314 | -0.058 | -0.1987 | No |
| 96 | ALDH6A1 |  |  | 7321 | -0.059 | -0.1940 | No |
| 97 | SLC30A1 |  |  | 7332 | -0.059 | -0.1897 | No |
| 98 | MINPP1 |  |  | 7498 | -0.063 | -0.2009 | No |
| 99 | IGSF3 |  |  | 7566 | -0.065 | -0.2019 | No |
| 100 | BCAM |  |  | 7587 | -0.066 | -0.1980 | No |
| 101 | ADD1 |  |  | 7750 | -0.072 | -0.2082 | No |
| 102 | MXI1 |  |  | 7832 | -0.075 | -0.2097 | No |
| 103 | ENDOD1 |  |  | 7904 | -0.078 | -0.2100 | No |
| 104 | TMEM9B |  |  | 7929 | -0.078 | -0.2054 | No |
| 105 | AGPAT4 |  |  | 7947 | -0.079 | -0.2000 | No |
| 106 | NARF |  |  | 8010 | -0.082 | -0.1990 | No |
| 107 | EPB41 |  |  | 8232 | -0.091 | -0.2135 | No |
| 108 | BSG |  |  | 8399 | -0.099 | -0.2216 | No |
| 109 | FBXO7 |  |  | 8575 | -0.110 | -0.2296 | Yes |
| 110 | CA2 |  |  | 8636 | -0.113 | -0.2255 | Yes |
| 111 | DAAM1 |  |  | 8691 | -0.117 | -0.2205 | Yes |
| 112 | BNIP3L |  |  | 8707 | -0.118 | -0.2114 | Yes |
| 113 | TFRC |  |  | 8715 | -0.119 | -0.2014 | Yes |
| 114 | KAT2B |  |  | 8754 | -0.122 | -0.1943 | Yes |
| 115 | NNT |  |  | 8770 | -0.123 | -0.1847 | Yes |
| 116 | SDCBP |  |  | 8790 | -0.124 | -0.1755 | Yes |
| 117 | NFE2L1 |  |  | 8845 | -0.128 | -0.1695 | Yes |
| 118 | PIGQ |  |  | 8891 | -0.133 | -0.1621 | Yes |
| 119 | SLC11A2 |  |  | 8894 | -0.134 | -0.1502 | Yes |
| 120 | ATP6V0A1 |  |  | 9018 | -0.144 | -0.1498 | Yes |
| 121 | MARK3 |  |  | 9052 | -0.147 | -0.1399 | Yes |
| 122 | SLC25A37 |  |  | 9067 | -0.149 | -0.1279 | Yes |
| 123 | LPIN2 |  |  | 9075 | -0.150 | -0.1151 | Yes |
| 124 | SLC6A8 |  |  | 9101 | -0.152 | -0.1039 | Yes |
| 125 | BTG2 |  |  | 9304 | -0.181 | -0.1083 | Yes |
| 126 | RNF19A |  |  | 9358 | -0.191 | -0.0965 | Yes |
| 127 | FOXO3 |  |  | 9503 | -0.226 | -0.0909 | Yes |
| 128 | RBM5 |  |  | 9506 | -0.227 | -0.0706 | Yes |
| 129 | CLCN3 |  |  | 9586 | -0.255 | -0.0557 | Yes |
| 130 | TNRC6B |  |  | 9589 | -0.256 | -0.0328 | Yes |
| 131 | NUDT4 |  |  | 9630 | -0.272 | -0.0124 | Yes |
| 132 | LAMP2 |  |  | 9796 | -0.419 | 0.0085 | Yes |
Table: GSEA details [plain text format]

  

Fig 2: HALLMARK\_HEME\_METABOLISM: Random ES distribution      
 Gene set null distribution of ES for **HALLMARK\_HEME\_METABOLISM**

  
