# Supplementary material for: Modeling glioblastoma heterogeneity as a dynamic network of cell states
Source: Mol Syst Biol. 2021 Sep 16;17(9):e10105. doi: 10.15252/msb.202010105 (PMC8444284; doi:10.15252/msb.202010105)
Supplement: Supplementary file 5 — Source Data for Figure 3 [file MSB-17-e10105-s001.zip › Figure3A_sourcedata/GSEA_3065/hallmarks_state1.GseaPreranked.1623416262439/HALLMARK_HYPOXIA.html]

Details for gene set HALLMARK\_HYPOXIA[GSEA]

|  || Dataset | state1 |
| Phenotype | NoPhenotypeAvailable |
| Upregulated in class | na\_pos |
| GeneSet | HALLMARK\_HYPOXIA |
| Enrichment Score (ES) | 0.39497423 |
| Normalized Enrichment Score (NES) | 1.5127825 |
| Nominal p-value | 0.0021645022 |
| FDR q-value | 0.03261807 |
| FWER p-Value | 0.218 |
Table: GSEA Results Summary

  

Fig 1: Enrichment plot: HALLMARK\_HYPOXIA      
 Profile of the Running ES Score & Positions of GeneSet Members on the Rank Ordered List

  

| PROBE | GENE SYMBOL | GENE\_TITLE | RANK IN GENE LIST | RANK METRIC SCORE | RUNNING ES | CORE ENRICHMENT || 1 | IGFBP3 |  |  | 15 | 0.664 | 0.0323 | Yes |
| 2 | CAVIN1 |  |  | 18 | 0.641 | 0.0648 | Yes |
| 3 | CSRP2 |  |  | 28 | 0.574 | 0.0931 | Yes |
| 4 | CAV1 |  |  | 34 | 0.536 | 0.1199 | Yes |
| 5 | ANXA2 |  |  | 48 | 0.482 | 0.1432 | Yes |
| 6 | TPI1 |  |  | 52 | 0.473 | 0.1670 | Yes |
| 7 | LDHA |  |  | 80 | 0.394 | 0.1843 | Yes |
| 8 | ENO1 |  |  | 99 | 0.373 | 0.2014 | Yes |
| 9 | GAPDH |  |  | 121 | 0.346 | 0.2169 | Yes |
| 10 | HOXB9 |  |  | 133 | 0.333 | 0.2328 | Yes |
| 11 | MYH9 |  |  | 140 | 0.328 | 0.2488 | Yes |
| 12 | CAVIN3 |  |  | 143 | 0.326 | 0.2653 | Yes |
| 13 | MT2A |  |  | 161 | 0.317 | 0.2796 | Yes |
| 14 | CHST2 |  |  | 182 | 0.305 | 0.2931 | Yes |
| 15 | SLC2A1 |  |  | 235 | 0.283 | 0.3022 | Yes |
| 16 | SRPX |  |  | 242 | 0.280 | 0.3158 | Yes |
| 17 | CDKN1A |  |  | 262 | 0.274 | 0.3279 | Yes |
| 18 | TPBG |  |  | 315 | 0.256 | 0.3356 | Yes |
| 19 | JUN |  |  | 387 | 0.229 | 0.3400 | Yes |
| 20 | MIF |  |  | 524 | 0.200 | 0.3362 | Yes |
| 21 | CXCR4 |  |  | 532 | 0.199 | 0.3456 | Yes |
| 22 | PFKP |  |  | 594 | 0.187 | 0.3489 | Yes |
| 23 | AKAP12 |  |  | 605 | 0.185 | 0.3573 | Yes |
| 24 | IDS |  |  | 625 | 0.182 | 0.3647 | Yes |
| 25 | PRDX5 |  |  | 640 | 0.180 | 0.3724 | Yes |
| 26 | CITED2 |  |  | 683 | 0.175 | 0.3770 | Yes |
| 27 | NDRG1 |  |  | 765 | 0.163 | 0.3770 | Yes |
| 28 | HMOX1 |  |  | 784 | 0.160 | 0.3833 | Yes |
| 29 | PGK1 |  |  | 875 | 0.148 | 0.3816 | Yes |
| 30 | STC2 |  |  | 922 | 0.142 | 0.3841 | Yes |
| 31 | GLRX |  |  | 927 | 0.142 | 0.3909 | Yes |
| 32 | HDLBP |  |  | 988 | 0.135 | 0.3916 | Yes |
| 33 | SDC2 |  |  | 1049 | 0.128 | 0.3920 | Yes |
| 34 | TIPARP |  |  | 1142 | 0.118 | 0.3886 | Yes |
| 35 | FOSL2 |  |  | 1143 | 0.118 | 0.3946 | Yes |
| 36 | PLIN2 |  |  | 1226 | 0.110 | 0.3918 | Yes |
| 37 | SERPINE1 |  |  | 1250 | 0.108 | 0.3950 | Yes |
| 38 | ERRFI1 |  |  | 1423 | 0.094 | 0.3821 | No |
| 39 | PLAUR |  |  | 1463 | 0.091 | 0.3828 | No |
| 40 | EGFR |  |  | 1474 | 0.091 | 0.3864 | No |
| 41 | ISG20 |  |  | 1538 | 0.087 | 0.3843 | No |
| 42 | CASP6 |  |  | 1564 | 0.085 | 0.3861 | No |
| 43 | GPI |  |  | 1615 | 0.081 | 0.3851 | No |
| 44 | NEDD4L |  |  | 1629 | 0.080 | 0.3879 | No |
| 45 | GBE1 |  |  | 1769 | 0.071 | 0.3772 | No |
| 46 | FAM162A |  |  | 1805 | 0.069 | 0.3772 | No |
| 47 | LOX |  |  | 1825 | 0.068 | 0.3787 | No |
| 48 | SLC6A6 |  |  | 1856 | 0.067 | 0.3790 | No |
| 49 | ADORA2B |  |  | 1891 | 0.066 | 0.3789 | No |
| 50 | P4HA2 |  |  | 1910 | 0.065 | 0.3803 | No |
| 51 | TPST2 |  |  | 1932 | 0.063 | 0.3814 | No |
| 52 | ALDOA |  |  | 1936 | 0.063 | 0.3843 | No |
| 53 | DDIT3 |  |  | 2008 | 0.059 | 0.3801 | No |
| 54 | SAP30 |  |  | 2022 | 0.059 | 0.3817 | No |
| 55 | TPD52 |  |  | 2098 | 0.055 | 0.3768 | No |
| 56 | SDC4 |  |  | 2111 | 0.054 | 0.3784 | No |
| 57 | S100A4 |  |  | 2114 | 0.054 | 0.3810 | No |
| 58 | HK1 |  |  | 2135 | 0.053 | 0.3816 | No |
| 59 | BTG1 |  |  | 2251 | 0.049 | 0.3723 | No |
| 60 | ADM |  |  | 2325 | 0.046 | 0.3672 | No |
| 61 | JMJD6 |  |  | 2405 | 0.043 | 0.3613 | No |
| 62 | GRHPR |  |  | 2414 | 0.043 | 0.3626 | No |
| 63 | MAFF |  |  | 2492 | 0.040 | 0.3568 | No |
| 64 | PFKL |  |  | 2621 | 0.035 | 0.3454 | No |
| 65 | DUSP1 |  |  | 2706 | 0.033 | 0.3385 | No |
| 66 | ATF3 |  |  | 2855 | 0.029 | 0.3248 | No |
| 67 | PFKFB3 |  |  | 3039 | 0.024 | 0.3072 | No |
| 68 | KDELR3 |  |  | 3057 | 0.023 | 0.3066 | No |
| 69 | ENO2 |  |  | 3161 | 0.021 | 0.2971 | No |
| 70 | RRAGD |  |  | 3229 | 0.020 | 0.2913 | No |
| 71 | PPP1R15A |  |  | 3296 | 0.018 | 0.2854 | No |
| 72 | PDGFB |  |  | 3613 | 0.012 | 0.2536 | No |
| 73 | TGFBI |  |  | 3688 | 0.010 | 0.2465 | No |
| 74 | XPNPEP1 |  |  | 3846 | 0.007 | 0.2308 | No |
| 75 | NFIL3 |  |  | 4056 | 0.003 | 0.2095 | No |
| 76 | KLF6 |  |  | 4117 | 0.002 | 0.2034 | No |
| 77 | GYS1 |  |  | 4410 | -0.003 | 0.1736 | No |
| 78 | PGM2 |  |  | 4604 | -0.006 | 0.1541 | No |
| 79 | TMEM45A |  |  | 4646 | -0.007 | 0.1502 | No |
| 80 | CDKN1B |  |  | 4655 | -0.007 | 0.1498 | No |
| 81 | NR3C1 |  |  | 4804 | -0.009 | 0.1350 | No |
| 82 | CDKN1C |  |  | 4834 | -0.010 | 0.1326 | No |
| 83 | GALK1 |  |  | 4872 | -0.010 | 0.1293 | No |
| 84 | EXT1 |  |  | 5106 | -0.014 | 0.1061 | No |
| 85 | SLC25A1 |  |  | 5164 | -0.015 | 0.1010 | No |
| 86 | SIAH2 |  |  | 5328 | -0.017 | 0.0852 | No |
| 87 | PGM1 |  |  | 5339 | -0.017 | 0.0850 | No |
| 88 | PDK3 |  |  | 5462 | -0.020 | 0.0735 | No |
| 89 | KLF7 |  |  | 5480 | -0.020 | 0.0727 | No |
| 90 | ERO1A |  |  | 5520 | -0.020 | 0.0698 | No |
| 91 | CCNG2 |  |  | 5718 | -0.024 | 0.0508 | No |
| 92 | DTNA |  |  | 6001 | -0.029 | 0.0233 | No |
| 93 | LARGE1 |  |  | 6016 | -0.029 | 0.0234 | No |
| 94 | BHLHE40 |  |  | 6102 | -0.031 | 0.0162 | No |
| 95 | B3GALT6 |  |  | 6425 | -0.037 | -0.0149 | No |
| 96 | VLDLR |  |  | 6518 | -0.039 | -0.0224 | No |
| 97 | DPYSL4 |  |  | 6529 | -0.039 | -0.0214 | No |
| 98 | DDIT4 |  |  | 6713 | -0.043 | -0.0380 | No |
| 99 | NDST1 |  |  | 6898 | -0.047 | -0.0545 | No |
| 100 | ETS1 |  |  | 7047 | -0.051 | -0.0671 | No |
| 101 | ATP7A |  |  | 7206 | -0.056 | -0.0804 | No |
| 102 | SCARB1 |  |  | 7359 | -0.060 | -0.0930 | No |
| 103 | PHKG1 |  |  | 7504 | -0.064 | -0.1045 | No |
| 104 | RBPJ |  |  | 7588 | -0.066 | -0.1097 | No |
| 105 | COL5A1 |  |  | 7642 | -0.067 | -0.1117 | No |
| 106 | SLC37A4 |  |  | 7693 | -0.069 | -0.1133 | No |
| 107 | STC1 |  |  | 7777 | -0.072 | -0.1181 | No |
| 108 | KDM3A |  |  | 7808 | -0.074 | -0.1175 | No |
| 109 | MXI1 |  |  | 7832 | -0.075 | -0.1160 | No |
| 110 | NAGK |  |  | 7950 | -0.079 | -0.1240 | No |
| 111 | PNRC1 |  |  | 8006 | -0.081 | -0.1255 | No |
| 112 | CHST3 |  |  | 8240 | -0.091 | -0.1447 | No |
| 113 | ILVBL |  |  | 8256 | -0.092 | -0.1416 | No |
| 114 | UGP2 |  |  | 8287 | -0.094 | -0.1399 | No |
| 115 | CA12 |  |  | 8291 | -0.094 | -0.1354 | No |
| 116 | PAM |  |  | 8343 | -0.096 | -0.1357 | No |
| 117 | VHL |  |  | 8346 | -0.097 | -0.1310 | No |
| 118 | ANKZF1 |  |  | 8423 | -0.100 | -0.1337 | No |
| 119 | KLHL24 |  |  | 8561 | -0.109 | -0.1422 | No |
| 120 | GPC4 |  |  | 8624 | -0.113 | -0.1428 | No |
| 121 | BNIP3L |  |  | 8707 | -0.118 | -0.1452 | No |
| 122 | MAP3K1 |  |  | 8750 | -0.121 | -0.1433 | No |
| 123 | GAA |  |  | 8798 | -0.124 | -0.1418 | No |
| 124 | P4HA1 |  |  | 8810 | -0.125 | -0.1365 | No |
| 125 | SLC2A3 |  |  | 9156 | -0.159 | -0.1638 | No |
| 126 | VEGFA |  |  | 9220 | -0.168 | -0.1617 | No |
| 127 | ZNF292 |  |  | 9395 | -0.200 | -0.1694 | No |
| 128 | HEXA |  |  | 9438 | -0.208 | -0.1631 | No |
| 129 | FOXO3 |  |  | 9503 | -0.226 | -0.1581 | No |
| 130 | HSPA5 |  |  | 9533 | -0.234 | -0.1492 | No |
| 131 | FOS |  |  | 9566 | -0.247 | -0.1399 | No |
| 132 | PRKCA |  |  | 9569 | -0.248 | -0.1274 | No |
| 133 | IRS2 |  |  | 9641 | -0.277 | -0.1206 | No |
| 134 | GPC1 |  |  | 9751 | -0.356 | -0.1136 | No |
| 135 | SDC3 |  |  | 9756 | -0.360 | -0.0957 | No |
| 136 | HS3ST1 |  |  | 9777 | -0.389 | -0.0779 | No |
| 137 | WSB1 |  |  | 9836 | -0.554 | -0.0556 | No |
| 138 | BCAN |  |  | 9878 | -1.175 | 0.0001 | No |
Table: GSEA details [plain text format]

  

Fig 2: HALLMARK\_HYPOXIA: Random ES distribution      
 Gene set null distribution of ES for **HALLMARK\_HYPOXIA**

  
