# Supplementary material for: Modeling glioblastoma heterogeneity as a dynamic network of cell states
Source: Mol Syst Biol. 2021 Sep 16;17(9):e10105. doi: 10.15252/msb.202010105 (PMC8444284; doi:10.15252/msb.202010105)
Supplement: Supplementary file 5 — Source Data for Figure 3 [file MSB-17-e10105-s001.zip › Figure3A_sourcedata/GSEA_3065/hallmarks_state1.GseaPreranked.1623416262439/HALLMARK_INFLAMMATORY_RESPONSE.html]

Details for gene set HALLMARK\_INFLAMMATORY\_RESPONSE[GSEA]

|  || Dataset | state1 |
| Phenotype | NoPhenotypeAvailable |
| Upregulated in class | na\_neg |
| GeneSet | HALLMARK\_INFLAMMATORY\_RESPONSE |
| Enrichment Score (ES) | -0.31321633 |
| Normalized Enrichment Score (NES) | -1.0534209 |
| Nominal p-value | 0.36571428 |
| FDR q-value | 0.64898163 |
| FWER p-Value | 1.0 |
Table: GSEA Results Summary

  

Fig 1: Enrichment plot: HALLMARK\_INFLAMMATORY\_RESPONSE      
 Profile of the Running ES Score & Positions of GeneSet Members on the Rank Ordered List

  

| PROBE | GENE SYMBOL | GENE\_TITLE | RANK IN GENE LIST | RANK METRIC SCORE | RUNNING ES | CORE ENRICHMENT || 1 | DCBLD2 |  |  | 62 | 0.444 | 0.0457 | No |
| 2 | ADRM1 |  |  | 160 | 0.317 | 0.0729 | No |
| 3 | CHST2 |  |  | 182 | 0.305 | 0.1065 | No |
| 4 | CDKN1A |  |  | 262 | 0.274 | 0.1305 | No |
| 5 | TPBG |  |  | 315 | 0.256 | 0.1552 | No |
| 6 | MET |  |  | 451 | 0.215 | 0.1667 | No |
| 7 | RGS16 |  |  | 499 | 0.206 | 0.1860 | No |
| 8 | LY6E |  |  | 649 | 0.179 | 0.1918 | No |
| 9 | AXL |  |  | 704 | 0.172 | 0.2064 | No |
| 10 | KCNJ2 |  |  | 914 | 0.143 | 0.2018 | No |
| 11 | CD82 |  |  | 1024 | 0.131 | 0.2061 | No |
| 12 | TNFRSF1B |  |  | 1031 | 0.130 | 0.2207 | No |
| 13 | SERPINE1 |  |  | 1250 | 0.108 | 0.2111 | No |
| 14 | SCN1B |  |  | 1460 | 0.092 | 0.2005 | No |
| 15 | PLAUR |  |  | 1463 | 0.091 | 0.2110 | No |
| 16 | IFNGR2 |  |  | 1555 | 0.086 | 0.2118 | No |
| 17 | IRF1 |  |  | 1569 | 0.085 | 0.2204 | No |
| 18 | NAMPT |  |  | 1622 | 0.081 | 0.2246 | No |
| 19 | RHOG |  |  | 1768 | 0.071 | 0.2182 | No |
| 20 | ADORA2B |  |  | 1891 | 0.066 | 0.2134 | No |
| 21 | SRI |  |  | 2084 | 0.056 | 0.2003 | No |
| 22 | NFKBIA |  |  | 2153 | 0.053 | 0.1996 | No |
| 23 | AHR |  |  | 2195 | 0.051 | 0.2014 | No |
| 24 | LYN |  |  | 2272 | 0.048 | 0.1993 | No |
| 25 | ITGA5 |  |  | 2302 | 0.047 | 0.2019 | No |
| 26 | ADM |  |  | 2325 | 0.046 | 0.2050 | No |
| 27 | TIMP1 |  |  | 2497 | 0.040 | 0.1922 | No |
| 28 | SLC31A2 |  |  | 2602 | 0.036 | 0.1858 | No |
| 29 | EIF2AK2 |  |  | 2806 | 0.030 | 0.1687 | No |
| 30 | BST2 |  |  | 3116 | 0.022 | 0.1398 | No |
| 31 | NFKB1 |  |  | 3337 | 0.017 | 0.1194 | No |
| 32 | IRF7 |  |  | 3585 | 0.012 | 0.0956 | No |
| 33 | SGMS2 |  |  | 3738 | 0.009 | 0.0811 | No |
| 34 | HIF1A |  |  | 3955 | 0.005 | 0.0597 | No |
| 35 | MYC |  |  | 3964 | 0.005 | 0.0595 | No |
| 36 | PTPRE |  |  | 4002 | 0.004 | 0.0562 | No |
| 37 | KLF6 |  |  | 4117 | 0.002 | 0.0448 | No |
| 38 | RIPK2 |  |  | 4145 | 0.002 | 0.0423 | No |
| 39 | NMI |  |  | 4288 | -0.001 | 0.0279 | No |
| 40 | RELA |  |  | 4420 | -0.003 | 0.0149 | No |
| 41 | ABI1 |  |  | 4714 | -0.008 | -0.0141 | No |
| 42 | LDLR |  |  | 4731 | -0.008 | -0.0148 | No |
| 43 | SLC7A1 |  |  | 4822 | -0.009 | -0.0229 | No |
| 44 | PVR |  |  | 4826 | -0.009 | -0.0221 | No |
| 45 | NDP |  |  | 4946 | -0.012 | -0.0328 | No |
| 46 | ATP2B1 |  |  | 5540 | -0.021 | -0.0909 | No |
| 47 | SEMA4D |  |  | 5593 | -0.022 | -0.0936 | No |
| 48 | CALCRL |  |  | 6193 | -0.033 | -0.1509 | No |
| 49 | HRH1 |  |  | 6215 | -0.033 | -0.1492 | No |
| 50 | CSF1 |  |  | 6351 | -0.036 | -0.1588 | No |
| 51 | MMP14 |  |  | 6548 | -0.039 | -0.1741 | No |
| 52 | EMP3 |  |  | 7404 | -0.061 | -0.2542 | No |
| 53 | GNAI3 |  |  | 7684 | -0.069 | -0.2746 | No |
| 54 | SLC31A1 |  |  | 7694 | -0.070 | -0.2674 | No |
| 55 | ATP2C1 |  |  | 7738 | -0.071 | -0.2634 | No |
| 56 | ACVR2A |  |  | 7786 | -0.073 | -0.2597 | No |
| 57 | GABBR1 |  |  | 8312 | -0.095 | -0.3021 | Yes |
| 58 | PSEN1 |  |  | 8386 | -0.098 | -0.2980 | Yes |
| 59 | RAF1 |  |  | 8419 | -0.100 | -0.2896 | Yes |
| 60 | CX3CL1 |  |  | 8465 | -0.103 | -0.2821 | Yes |
| 61 | OSMR |  |  | 8515 | -0.106 | -0.2747 | Yes |
| 62 | TAPBP |  |  | 8522 | -0.106 | -0.2629 | Yes |
| 63 | ABCA1 |  |  | 8590 | -0.111 | -0.2567 | Yes |
| 64 | SELENOS |  |  | 8867 | -0.130 | -0.2696 | Yes |
| 65 | SLC11A2 |  |  | 8894 | -0.134 | -0.2566 | Yes |
| 66 | IFNAR1 |  |  | 9068 | -0.149 | -0.2568 | Yes |
| 67 | PDPN |  |  | 9284 | -0.177 | -0.2579 | Yes |
| 68 | BTG2 |  |  | 9304 | -0.181 | -0.2387 | Yes |
| 69 | PDE4B |  |  | 9522 | -0.231 | -0.2337 | Yes |
| 70 | ATP2A2 |  |  | 9610 | -0.263 | -0.2118 | Yes |
| 71 | TNFAIP6 |  |  | 9629 | -0.272 | -0.1818 | Yes |
| 72 | KIF1B |  |  | 9649 | -0.282 | -0.1507 | Yes |
| 73 | HAS2 |  |  | 9739 | -0.350 | -0.1188 | Yes |
| 74 | SLC4A4 |  |  | 9749 | -0.355 | -0.0781 | Yes |
| 75 | P2RX7 |  |  | 9755 | -0.360 | -0.0364 | Yes |
| 76 | ITGB8 |  |  | 9794 | -0.418 | 0.0087 | Yes |
Table: GSEA details [plain text format]

  

Fig 2: HALLMARK\_INFLAMMATORY\_RESPONSE: Random ES distribution      
 Gene set null distribution of ES for **HALLMARK\_INFLAMMATORY\_RESPONSE**

  
