# Supplementary material for: Modeling glioblastoma heterogeneity as a dynamic network of cell states
Source: Mol Syst Biol. 2021 Sep 16;17(9):e10105. doi: 10.15252/msb.202010105 (PMC8444284; doi:10.15252/msb.202010105)
Supplement: Supplementary file 5 — Source Data for Figure 3 [file MSB-17-e10105-s001.zip › Figure3A_sourcedata/GSEA_3065/hallmarks_state1.GseaPreranked.1623416262439/HALLMARK_INTERFERON_ALPHA_RESPONSE.html]

Details for gene set HALLMARK\_INTERFERON\_ALPHA\_RESPONSE[GSEA]

|  || Dataset | state1 |
| Phenotype | NoPhenotypeAvailable |
| Upregulated in class | na\_neg |
| GeneSet | HALLMARK\_INTERFERON\_ALPHA\_RESPONSE |
| Enrichment Score (ES) | -0.27784574 |
| Normalized Enrichment Score (NES) | -0.8904586 |
| Nominal p-value | 0.64915574 |
| FDR q-value | 0.7421836 |
| FWER p-Value | 1.0 |
Table: GSEA Results Summary

  

Fig 1: Enrichment plot: HALLMARK\_INTERFERON\_ALPHA\_RESPONSE      
 Profile of the Running ES Score & Positions of GeneSet Members on the Rank Ordered List

  

| PROBE | GENE SYMBOL | GENE\_TITLE | RANK IN GENE LIST | RANK METRIC SCORE | RUNNING ES | CORE ENRICHMENT || 1 | PSME2 |  |  | 112 | 0.351 | 0.0504 | No |
| 2 | GMPR |  |  | 223 | 0.287 | 0.0897 | No |
| 3 | PSME1 |  |  | 519 | 0.201 | 0.0951 | No |
| 4 | PSMA3 |  |  | 528 | 0.200 | 0.1294 | No |
| 5 | ISG15 |  |  | 535 | 0.198 | 0.1637 | No |
| 6 | LY6E |  |  | 649 | 0.179 | 0.1837 | No |
| 7 | PSMB9 |  |  | 745 | 0.166 | 0.2033 | No |
| 8 | PSMB8 |  |  | 1007 | 0.133 | 0.2002 | No |
| 9 | IRF2 |  |  | 1299 | 0.103 | 0.1887 | No |
| 10 | USP18 |  |  | 1342 | 0.100 | 0.2020 | No |
| 11 | LAP3 |  |  | 1418 | 0.094 | 0.2110 | No |
| 12 | IFI35 |  |  | 1468 | 0.091 | 0.2220 | No |
| 13 | ISG20 |  |  | 1538 | 0.087 | 0.2303 | No |
| 14 | IRF1 |  |  | 1569 | 0.085 | 0.2422 | No |
| 15 | UBE2L6 |  |  | 1852 | 0.067 | 0.2253 | No |
| 16 | OAS1 |  |  | 2081 | 0.056 | 0.2119 | No |
| 17 | PROCR |  |  | 2129 | 0.054 | 0.2166 | No |
| 18 | PLSCR1 |  |  | 2379 | 0.044 | 0.1990 | No |
| 19 | MX1 |  |  | 2411 | 0.043 | 0.2034 | No |
| 20 | CASP8 |  |  | 2688 | 0.033 | 0.1811 | No |
| 21 | TRIM25 |  |  | 2694 | 0.033 | 0.1864 | No |
| 22 | EIF2AK2 |  |  | 2806 | 0.030 | 0.1805 | No |
| 23 | BST2 |  |  | 3116 | 0.022 | 0.1529 | No |
| 24 | MVB12A |  |  | 3178 | 0.021 | 0.1504 | No |
| 25 | TRIM26 |  |  | 3231 | 0.020 | 0.1485 | No |
| 26 | NCOA7 |  |  | 3338 | 0.017 | 0.1408 | No |
| 27 | HERC6 |  |  | 3581 | 0.012 | 0.1183 | No |
| 28 | IRF7 |  |  | 3585 | 0.012 | 0.1201 | No |
| 29 | IFIT3 |  |  | 3805 | 0.008 | 0.0992 | No |
| 30 | TDRD7 |  |  | 4069 | 0.003 | 0.0729 | No |
| 31 | RIPK2 |  |  | 4145 | 0.002 | 0.0656 | No |
| 32 | NMI |  |  | 4288 | -0.001 | 0.0513 | No |
| 33 | TRIM14 |  |  | 4612 | -0.006 | 0.0195 | No |
| 34 | MOV10 |  |  | 4648 | -0.007 | 0.0172 | No |
| 35 | TRAFD1 |  |  | 4719 | -0.008 | 0.0114 | No |
| 36 | SLC25A28 |  |  | 4780 | -0.009 | 0.0068 | No |
| 37 | SAMD9L |  |  | 5021 | -0.013 | -0.0154 | No |
| 38 | NUB1 |  |  | 5089 | -0.014 | -0.0198 | No |
| 39 | TAP1 |  |  | 5226 | -0.016 | -0.0308 | No |
| 40 | ELF1 |  |  | 5444 | -0.019 | -0.0496 | No |
| 41 | OGFR |  |  | 5643 | -0.023 | -0.0657 | No |
| 42 | DDX60 |  |  | 5911 | -0.028 | -0.0880 | No |
| 43 | SP110 |  |  | 5963 | -0.029 | -0.0882 | No |
| 44 | CMTR1 |  |  | 6303 | -0.035 | -0.1166 | No |
| 45 | CSF1 |  |  | 6351 | -0.036 | -0.1151 | No |
| 46 | IFI44 |  |  | 6549 | -0.039 | -0.1282 | No |
| 47 | HELZ2 |  |  | 6854 | -0.046 | -0.1511 | No |
| 48 | IFITM2 |  |  | 7258 | -0.057 | -0.1821 | No |
| 49 | CNP |  |  | 7303 | -0.058 | -0.1764 | No |
| 50 | TRIM5 |  |  | 7598 | -0.066 | -0.1947 | No |
| 51 | PARP14 |  |  | 7988 | -0.081 | -0.2201 | No |
| 52 | SAMD9 |  |  | 8261 | -0.093 | -0.2315 | No |
| 53 | PNPT1 |  |  | 8313 | -0.095 | -0.2200 | No |
| 54 | PARP9 |  |  | 8650 | -0.114 | -0.2340 | No |
| 55 | STAT2 |  |  | 9081 | -0.150 | -0.2514 | Yes |
| 56 | CD47 |  |  | 9175 | -0.162 | -0.2322 | Yes |
| 57 | ADAR |  |  | 9255 | -0.173 | -0.2099 | Yes |
| 58 | LGALS3BP |  |  | 9447 | -0.212 | -0.1920 | Yes |
| 59 | TXNIP |  |  | 9639 | -0.276 | -0.1628 | Yes |
| 60 | IFITM3 |  |  | 9643 | -0.277 | -0.1143 | Yes |
| 61 | CD74 |  |  | 9766 | -0.376 | -0.0605 | Yes |
| 62 | B2M |  |  | 9791 | -0.408 | 0.0090 | Yes |
Table: GSEA details [plain text format]

  

Fig 2: HALLMARK\_INTERFERON\_ALPHA\_RESPONSE: Random ES distribution      
 Gene set null distribution of ES for **HALLMARK\_INTERFERON\_ALPHA\_RESPONSE**

  
