# Supplementary material for: Modeling glioblastoma heterogeneity as a dynamic network of cell states
Source: Mol Syst Biol. 2021 Sep 16;17(9):e10105. doi: 10.15252/msb.202010105 (PMC8444284; doi:10.15252/msb.202010105)
Supplement: Supplementary file 5 — Source Data for Figure 3 [file MSB-17-e10105-s001.zip › Figure3A_sourcedata/GSEA_3065/hallmarks_state1.GseaPreranked.1623416262439/HALLMARK_INTERFERON_GAMMA_RESPONSE.html]

Details for gene set HALLMARK\_INTERFERON\_GAMMA\_RESPONSE[GSEA]

|  || Dataset | state1 |
| Phenotype | NoPhenotypeAvailable |
| Upregulated in class | na\_pos |
| GeneSet | HALLMARK\_INTERFERON\_GAMMA\_RESPONSE |
| Enrichment Score (ES) | 0.3332827 |
| Normalized Enrichment Score (NES) | 1.2057811 |
| Nominal p-value | 0.13225058 |
| FDR q-value | 0.24676545 |
| FWER p-Value | 0.966 |
Table: GSEA Results Summary

  

Fig 1: Enrichment plot: HALLMARK\_INTERFERON\_GAMMA\_RESPONSE      
 Profile of the Running ES Score & Positions of GeneSet Members on the Rank Ordered List

  

| PROBE | GENE SYMBOL | GENE\_TITLE | RANK IN GENE LIST | RANK METRIC SCORE | RUNNING ES | CORE ENRICHMENT || 1 | UPP1 |  |  | 37 | 0.523 | 0.0474 | Yes |
| 2 | PSME2 |  |  | 112 | 0.351 | 0.0742 | Yes |
| 3 | MT2A |  |  | 161 | 0.317 | 0.1003 | Yes |
| 4 | VAMP5 |  |  | 246 | 0.278 | 0.1190 | Yes |
| 5 | CDKN1A |  |  | 262 | 0.274 | 0.1444 | Yes |
| 6 | RBCK1 |  |  | 329 | 0.250 | 0.1621 | Yes |
| 7 | METTL7B |  |  | 355 | 0.239 | 0.1830 | Yes |
| 8 | PSMB2 |  |  | 456 | 0.214 | 0.1937 | Yes |
| 9 | ARL4A |  |  | 497 | 0.206 | 0.2099 | Yes |
| 10 | PSME1 |  |  | 519 | 0.201 | 0.2274 | Yes |
| 11 | PSMA3 |  |  | 528 | 0.200 | 0.2461 | Yes |
| 12 | ISG15 |  |  | 535 | 0.198 | 0.2649 | Yes |
| 13 | PFKP |  |  | 594 | 0.187 | 0.2773 | Yes |
| 14 | LY6E |  |  | 649 | 0.179 | 0.2893 | Yes |
| 15 | PSMB9 |  |  | 745 | 0.166 | 0.2959 | Yes |
| 16 | MTHFD2 |  |  | 899 | 0.145 | 0.2945 | Yes |
| 17 | CASP3 |  |  | 923 | 0.142 | 0.3061 | Yes |
| 18 | PSMB8 |  |  | 1007 | 0.133 | 0.3106 | Yes |
| 19 | CASP7 |  |  | 1205 | 0.112 | 0.3014 | Yes |
| 20 | PSMA2 |  |  | 1218 | 0.111 | 0.3111 | Yes |
| 21 | IRF2 |  |  | 1299 | 0.103 | 0.3130 | Yes |
| 22 | USP18 |  |  | 1342 | 0.100 | 0.3185 | Yes |
| 23 | LAP3 |  |  | 1418 | 0.094 | 0.3200 | Yes |
| 24 | IFI35 |  |  | 1468 | 0.091 | 0.3240 | Yes |
| 25 | ISG20 |  |  | 1538 | 0.087 | 0.3254 | Yes |
| 26 | IRF1 |  |  | 1569 | 0.085 | 0.3307 | Yes |
| 27 | NAMPT |  |  | 1622 | 0.081 | 0.3333 | Yes |
| 28 | UBE2L6 |  |  | 1852 | 0.067 | 0.3164 | No |
| 29 | PML |  |  | 1879 | 0.066 | 0.3202 | No |
| 30 | PTPN1 |  |  | 1948 | 0.063 | 0.3195 | No |
| 31 | CASP4 |  |  | 2054 | 0.057 | 0.3143 | No |
| 32 | SRI |  |  | 2084 | 0.056 | 0.3168 | No |
| 33 | NFKBIA |  |  | 2153 | 0.053 | 0.3150 | No |
| 34 | BTG1 |  |  | 2251 | 0.049 | 0.3099 | No |
| 35 | OAS3 |  |  | 2342 | 0.046 | 0.3052 | No |
| 36 | PLSCR1 |  |  | 2379 | 0.044 | 0.3058 | No |
| 37 | MX1 |  |  | 2411 | 0.043 | 0.3068 | No |
| 38 | LYSMD2 |  |  | 2527 | 0.039 | 0.2988 | No |
| 39 | CASP8 |  |  | 2688 | 0.033 | 0.2857 | No |
| 40 | TRIM25 |  |  | 2694 | 0.033 | 0.2885 | No |
| 41 | ARID5B |  |  | 2743 | 0.032 | 0.2867 | No |
| 42 | EIF2AK2 |  |  | 2806 | 0.030 | 0.2833 | No |
| 43 | MYD88 |  |  | 2810 | 0.030 | 0.2859 | No |
| 44 | BST2 |  |  | 3116 | 0.022 | 0.2569 | No |
| 45 | PTPN2 |  |  | 3136 | 0.022 | 0.2571 | No |
| 46 | SAMHD1 |  |  | 3180 | 0.021 | 0.2547 | No |
| 47 | PSMB10 |  |  | 3189 | 0.021 | 0.2559 | No |
| 48 | MVP |  |  | 3193 | 0.020 | 0.2576 | No |
| 49 | TRIM26 |  |  | 3231 | 0.020 | 0.2558 | No |
| 50 | NFKB1 |  |  | 3337 | 0.017 | 0.2468 | No |
| 51 | ISOC1 |  |  | 3358 | 0.017 | 0.2464 | No |
| 52 | LATS2 |  |  | 3538 | 0.013 | 0.2293 | No |
| 53 | HERC6 |  |  | 3581 | 0.012 | 0.2262 | No |
| 54 | IRF7 |  |  | 3585 | 0.012 | 0.2271 | No |
| 55 | SOD2 |  |  | 3646 | 0.011 | 0.2220 | No |
| 56 | APOL6 |  |  | 3729 | 0.009 | 0.2146 | No |
| 57 | RIPK1 |  |  | 3739 | 0.009 | 0.2145 | No |
| 58 | IFIT3 |  |  | 3805 | 0.008 | 0.2087 | No |
| 59 | FAS |  |  | 3812 | 0.008 | 0.2088 | No |
| 60 | HIF1A |  |  | 3955 | 0.005 | 0.1948 | No |
| 61 | ZNFX1 |  |  | 4054 | 0.003 | 0.1851 | No |
| 62 | TDRD7 |  |  | 4069 | 0.003 | 0.1840 | No |
| 63 | RIPK2 |  |  | 4145 | 0.002 | 0.1765 | No |
| 64 | NMI |  |  | 4288 | -0.001 | 0.1620 | No |
| 65 | TRIM14 |  |  | 4612 | -0.006 | 0.1296 | No |
| 66 | TRAFD1 |  |  | 4719 | -0.008 | 0.1195 | No |
| 67 | SLC25A28 |  |  | 4780 | -0.009 | 0.1142 | No |
| 68 | SAMD9L |  |  | 5021 | -0.013 | 0.0910 | No |
| 69 | BPGM |  |  | 5064 | -0.013 | 0.0880 | No |
| 70 | NCOA3 |  |  | 5140 | -0.015 | 0.0817 | No |
| 71 | TAP1 |  |  | 5226 | -0.016 | 0.0746 | No |
| 72 | NUP93 |  |  | 5407 | -0.019 | 0.0580 | No |
| 73 | OGFR |  |  | 5643 | -0.023 | 0.0362 | No |
| 74 | TOR1B |  |  | 5786 | -0.025 | 0.0241 | No |
| 75 | DDX58 |  |  | 5907 | -0.028 | 0.0146 | No |
| 76 | DDX60 |  |  | 5911 | -0.028 | 0.0170 | No |
| 77 | SP110 |  |  | 5963 | -0.029 | 0.0146 | No |
| 78 | CMTR1 |  |  | 6303 | -0.035 | -0.0167 | No |
| 79 | IFI44 |  |  | 6549 | -0.039 | -0.0379 | No |
| 80 | HELZ2 |  |  | 6854 | -0.046 | -0.0645 | No |
| 81 | SSPN |  |  | 6940 | -0.048 | -0.0684 | No |
| 82 | IFNAR2 |  |  | 7037 | -0.051 | -0.0733 | No |
| 83 | IFITM2 |  |  | 7258 | -0.057 | -0.0902 | No |
| 84 | STAT3 |  |  | 7597 | -0.066 | -0.1183 | No |
| 85 | STAT1 |  |  | 7639 | -0.067 | -0.1159 | No |
| 86 | PARP14 |  |  | 7988 | -0.081 | -0.1436 | No |
| 87 | PNPT1 |  |  | 8313 | -0.095 | -0.1674 | No |
| 88 | SOCS3 |  |  | 8373 | -0.098 | -0.1639 | No |
| 89 | TAPBP |  |  | 8522 | -0.106 | -0.1686 | No |
| 90 | AUTS2 |  |  | 8583 | -0.111 | -0.1639 | No |
| 91 | STAT2 |  |  | 9081 | -0.150 | -0.2000 | No |
| 92 | ST3GAL5 |  |  | 9085 | -0.151 | -0.1855 | No |
| 93 | SPPL2A |  |  | 9100 | -0.152 | -0.1721 | No |
| 94 | RNF213 |  |  | 9120 | -0.153 | -0.1590 | No |
| 95 | ADAR |  |  | 9255 | -0.173 | -0.1558 | No |
| 96 | C1R |  |  | 9298 | -0.180 | -0.1424 | No |
| 97 | LGALS3BP |  |  | 9447 | -0.212 | -0.1368 | No |
| 98 | PDE4B |  |  | 9522 | -0.231 | -0.1217 | No |
| 99 | TNFAIP6 |  |  | 9629 | -0.272 | -0.1059 | No |
| 100 | TXNIP |  |  | 9639 | -0.276 | -0.0798 | No |
| 101 | IFITM3 |  |  | 9643 | -0.277 | -0.0529 | No |
| 102 | CD74 |  |  | 9766 | -0.376 | -0.0286 | No |
| 103 | B2M |  |  | 9791 | -0.408 | 0.0090 | No |
Table: GSEA details [plain text format]

  

Fig 2: HALLMARK\_INTERFERON\_GAMMA\_RESPONSE: Random ES distribution      
 Gene set null distribution of ES for **HALLMARK\_INTERFERON\_GAMMA\_RESPONSE**

  
