# Supplementary material for: Modeling glioblastoma heterogeneity as a dynamic network of cell states
Source: Mol Syst Biol. 2021 Sep 16;17(9):e10105. doi: 10.15252/msb.202010105 (PMC8444284; doi:10.15252/msb.202010105)
Supplement: Supplementary file 5 — Source Data for Figure 3 [file MSB-17-e10105-s001.zip › Figure3A_sourcedata/GSEA_3065/hallmarks_state1.GseaPreranked.1623416262439/HALLMARK_KRAS_SIGNALING_DN.html]

Details for gene set HALLMARK\_KRAS\_SIGNALING\_DN[GSEA]

|  || Dataset | state1 |
| Phenotype | NoPhenotypeAvailable |
| Upregulated in class | na\_neg |
| GeneSet | HALLMARK\_KRAS\_SIGNALING\_DN |
| Enrichment Score (ES) | -0.44411388 |
| Normalized Enrichment Score (NES) | -1.32217 |
| Nominal p-value | 0.09867173 |
| FDR q-value | 0.4717559 |
| FWER p-Value | 0.821 |
Table: GSEA Results Summary

  

Fig 1: Enrichment plot: HALLMARK\_KRAS\_SIGNALING\_DN      
 Profile of the Running ES Score & Positions of GeneSet Members on the Rank Ordered List

  

| PROBE | GENE SYMBOL | GENE\_TITLE | RANK IN GENE LIST | RANK METRIC SCORE | RUNNING ES | CORE ENRICHMENT || 1 | CHST2 |  |  | 182 | 0.305 | 0.0818 | No |
| 2 | GAMT |  |  | 843 | 0.152 | 0.0648 | No |
| 3 | COPZ2 |  |  | 1990 | 0.060 | -0.0318 | No |
| 4 | SHOX2 |  |  | 2091 | 0.055 | -0.0238 | No |
| 5 | MX1 |  |  | 2411 | 0.043 | -0.0420 | No |
| 6 | MFSD6 |  |  | 3302 | 0.018 | -0.1265 | No |
| 7 | PDK2 |  |  | 3920 | 0.006 | -0.1872 | No |
| 8 | NUDT11 |  |  | 4390 | -0.003 | -0.2340 | No |
| 9 | GTF3C5 |  |  | 4667 | -0.007 | -0.2598 | No |
| 10 | DTNB |  |  | 4687 | -0.007 | -0.2593 | No |
| 11 | CCDC106 |  |  | 4760 | -0.008 | -0.2639 | No |
| 12 | EFHD1 |  |  | 4776 | -0.008 | -0.2627 | No |
| 13 | FGGY |  |  | 4794 | -0.009 | -0.2615 | No |
| 14 | DCC |  |  | 4919 | -0.011 | -0.2704 | No |
| 15 | CAMK1D |  |  | 6057 | -0.030 | -0.3760 | No |
| 16 | CDKAL1 |  |  | 6308 | -0.035 | -0.3900 | No |
| 17 | SLC25A23 |  |  | 6512 | -0.039 | -0.3978 | No |
| 18 | SPHK2 |  |  | 6819 | -0.045 | -0.4140 | No |
| 19 | KMT2D |  |  | 6820 | -0.045 | -0.3991 | No |
| 20 | PTPRJ |  |  | 6938 | -0.048 | -0.3950 | No |
| 21 | VPS50 |  |  | 7056 | -0.052 | -0.3899 | No |
| 22 | C5 |  |  | 7355 | -0.059 | -0.4006 | No |
| 23 | LFNG |  |  | 7784 | -0.073 | -0.4202 | Yes |
| 24 | BARD1 |  |  | 7980 | -0.080 | -0.4135 | Yes |
| 25 | IDUA |  |  | 8039 | -0.083 | -0.3922 | Yes |
| 26 | SKIL |  |  | 8133 | -0.087 | -0.3732 | Yes |
| 27 | SLC38A3 |  |  | 8217 | -0.090 | -0.3519 | Yes |
| 28 | TCF7L1 |  |  | 8342 | -0.096 | -0.3328 | Yes |
| 29 | TGFB2 |  |  | 8497 | -0.105 | -0.3139 | Yes |
| 30 | SNN |  |  | 8815 | -0.126 | -0.3047 | Yes |
| 31 | YPEL1 |  |  | 8826 | -0.126 | -0.2641 | Yes |
| 32 | KLHDC8A |  |  | 9117 | -0.153 | -0.2431 | Yes |
| 33 | IGFBP2 |  |  | 9209 | -0.167 | -0.1974 | Yes |
| 34 | CELSR2 |  |  | 9238 | -0.171 | -0.1439 | Yes |
| 35 | BTG2 |  |  | 9304 | -0.181 | -0.0910 | Yes |
| 36 | KCNQ2 |  |  | 9335 | -0.187 | -0.0325 | Yes |
| 37 | FGFR3 |  |  | 9621 | -0.266 | 0.0262 | Yes |
Table: GSEA details [plain text format]

  

Fig 2: HALLMARK\_KRAS\_SIGNALING\_DN: Random ES distribution      
 Gene set null distribution of ES for **HALLMARK\_KRAS\_SIGNALING\_DN**

  
