# Supplementary material for: Modeling glioblastoma heterogeneity as a dynamic network of cell states
Source: Mol Syst Biol. 2021 Sep 16;17(9):e10105. doi: 10.15252/msb.202010105 (PMC8444284; doi:10.15252/msb.202010105)
Supplement: Supplementary file 5 — Source Data for Figure 3 [file MSB-17-e10105-s001.zip › Figure3A_sourcedata/GSEA_3065/hallmarks_state1.GseaPreranked.1623416262439/HALLMARK_MTORC1_SIGNALING.html]

Details for gene set HALLMARK\_MTORC1\_SIGNALING[GSEA]

|  || Dataset | state1 |
| Phenotype | NoPhenotypeAvailable |
| Upregulated in class | na\_pos |
| GeneSet | HALLMARK\_MTORC1\_SIGNALING |
| Enrichment Score (ES) | 0.40808582 |
| Normalized Enrichment Score (NES) | 1.5908431 |
| Nominal p-value | 0.0 |
| FDR q-value | 0.020842195 |
| FWER p-Value | 0.107 |
Table: GSEA Results Summary

  

Fig 1: Enrichment plot: HALLMARK\_MTORC1\_SIGNALING      
 Profile of the Running ES Score & Positions of GeneSet Members on the Rank Ordered List

  

| PROBE | GENE SYMBOL | GENE\_TITLE | RANK IN GENE LIST | RANK METRIC SCORE | RUNNING ES | CORE ENRICHMENT || 1 | CTSC |  |  | 43 | 0.498 | 0.0180 | Yes |
| 2 | TPI1 |  |  | 52 | 0.473 | 0.0385 | Yes |
| 3 | LDHA |  |  | 80 | 0.394 | 0.0535 | Yes |
| 4 | IGFBP5 |  |  | 88 | 0.385 | 0.0701 | Yes |
| 5 | TXNRD1 |  |  | 97 | 0.376 | 0.0863 | Yes |
| 6 | ENO1 |  |  | 99 | 0.373 | 0.1030 | Yes |
| 7 | GAPDH |  |  | 121 | 0.346 | 0.1164 | Yes |
| 8 | PPIA |  |  | 150 | 0.324 | 0.1281 | Yes |
| 9 | G6PD |  |  | 171 | 0.310 | 0.1400 | Yes |
| 10 | CCT6A |  |  | 181 | 0.305 | 0.1528 | Yes |
| 11 | SLC2A1 |  |  | 235 | 0.283 | 0.1601 | Yes |
| 12 | CDKN1A |  |  | 262 | 0.274 | 0.1698 | Yes |
| 13 | PDAP1 |  |  | 280 | 0.268 | 0.1801 | Yes |
| 14 | HPRT1 |  |  | 289 | 0.263 | 0.1911 | Yes |
| 15 | MLLT11 |  |  | 321 | 0.254 | 0.1994 | Yes |
| 16 | HSPD1 |  |  | 397 | 0.227 | 0.2019 | Yes |
| 17 | ARPC5L |  |  | 409 | 0.224 | 0.2108 | Yes |
| 18 | PSPH |  |  | 422 | 0.222 | 0.2196 | Yes |
| 19 | TUBG1 |  |  | 439 | 0.219 | 0.2278 | Yes |
| 20 | CACYBP |  |  | 458 | 0.214 | 0.2356 | Yes |
| 21 | SLC7A11 |  |  | 459 | 0.214 | 0.2452 | Yes |
| 22 | TCEA1 |  |  | 481 | 0.210 | 0.2525 | Yes |
| 23 | PPA1 |  |  | 498 | 0.206 | 0.2602 | Yes |
| 24 | PSMC2 |  |  | 506 | 0.205 | 0.2687 | Yes |
| 25 | PSMA3 |  |  | 528 | 0.200 | 0.2755 | Yes |
| 26 | CXCR4 |  |  | 532 | 0.199 | 0.2842 | Yes |
| 27 | HSPE1 |  |  | 545 | 0.197 | 0.2918 | Yes |
| 28 | AURKA |  |  | 550 | 0.196 | 0.3002 | Yes |
| 29 | PSMD13 |  |  | 559 | 0.193 | 0.3081 | Yes |
| 30 | PSMB5 |  |  | 599 | 0.186 | 0.3124 | Yes |
| 31 | EIF2S2 |  |  | 610 | 0.184 | 0.3197 | Yes |
| 32 | STIP1 |  |  | 623 | 0.182 | 0.3267 | Yes |
| 33 | ELOVL6 |  |  | 690 | 0.174 | 0.3277 | Yes |
| 34 | YKT6 |  |  | 698 | 0.172 | 0.3348 | Yes |
| 35 | SQSTM1 |  |  | 701 | 0.172 | 0.3423 | Yes |
| 36 | ACTR3 |  |  | 744 | 0.166 | 0.3455 | Yes |
| 37 | UBE2D3 |  |  | 782 | 0.160 | 0.3489 | Yes |
| 38 | PSMC4 |  |  | 803 | 0.157 | 0.3538 | Yes |
| 39 | ATP5MC1 |  |  | 820 | 0.154 | 0.3592 | Yes |
| 40 | EBP |  |  | 829 | 0.154 | 0.3653 | Yes |
| 41 | PSMA4 |  |  | 837 | 0.153 | 0.3714 | Yes |
| 42 | EEF1E1 |  |  | 857 | 0.150 | 0.3762 | Yes |
| 43 | PSMG1 |  |  | 871 | 0.148 | 0.3816 | Yes |
| 44 | PGK1 |  |  | 875 | 0.148 | 0.3879 | Yes |
| 45 | MTHFD2 |  |  | 899 | 0.145 | 0.3921 | Yes |
| 46 | GLRX |  |  | 927 | 0.142 | 0.3957 | Yes |
| 47 | SERP1 |  |  | 1030 | 0.130 | 0.3911 | Yes |
| 48 | RRP9 |  |  | 1072 | 0.125 | 0.3925 | Yes |
| 49 | ETF1 |  |  | 1121 | 0.120 | 0.3930 | Yes |
| 50 | CYB5B |  |  | 1162 | 0.117 | 0.3941 | Yes |
| 51 | PSMD12 |  |  | 1168 | 0.116 | 0.3988 | Yes |
| 52 | QDPR |  |  | 1242 | 0.109 | 0.3962 | Yes |
| 53 | DDX39A |  |  | 1255 | 0.108 | 0.3998 | Yes |
| 54 | PRDX1 |  |  | 1305 | 0.103 | 0.3994 | Yes |
| 55 | SLC7A5 |  |  | 1312 | 0.102 | 0.4034 | Yes |
| 56 | GOT1 |  |  | 1344 | 0.100 | 0.4047 | Yes |
| 57 | PLK1 |  |  | 1403 | 0.095 | 0.4030 | Yes |
| 58 | DHFR |  |  | 1413 | 0.094 | 0.4063 | Yes |
| 59 | ATP6V1D |  |  | 1441 | 0.093 | 0.4077 | Yes |
| 60 | DHCR7 |  |  | 1553 | 0.086 | 0.4001 | Yes |
| 61 | GPI |  |  | 1615 | 0.081 | 0.3975 | Yes |
| 62 | NAMPT |  |  | 1622 | 0.081 | 0.4006 | Yes |
| 63 | MAP2K3 |  |  | 1643 | 0.079 | 0.4021 | Yes |
| 64 | UFM1 |  |  | 1663 | 0.078 | 0.4036 | Yes |
| 65 | TOMM40 |  |  | 1705 | 0.075 | 0.4028 | Yes |
| 66 | CCNG1 |  |  | 1716 | 0.074 | 0.4051 | Yes |
| 67 | RAB1A |  |  | 1720 | 0.074 | 0.4081 | Yes |
| 68 | GBE1 |  |  | 1769 | 0.071 | 0.4064 | No |
| 69 | HSPA9 |  |  | 1793 | 0.070 | 0.4071 | No |
| 70 | SLC6A6 |  |  | 1856 | 0.067 | 0.4038 | No |
| 71 | UCHL5 |  |  | 1934 | 0.063 | 0.3987 | No |
| 72 | ALDOA |  |  | 1936 | 0.063 | 0.4014 | No |
| 73 | HMBS |  |  | 1947 | 0.063 | 0.4032 | No |
| 74 | DDIT3 |  |  | 2008 | 0.059 | 0.3997 | No |
| 75 | PSMD14 |  |  | 2025 | 0.058 | 0.4007 | No |
| 76 | TUBA4A |  |  | 2124 | 0.054 | 0.3930 | No |
| 77 | SDF2L1 |  |  | 2126 | 0.054 | 0.3954 | No |
| 78 | GMPS |  |  | 2197 | 0.051 | 0.3904 | No |
| 79 | PNO1 |  |  | 2235 | 0.050 | 0.3889 | No |
| 80 | PSME3 |  |  | 2277 | 0.048 | 0.3868 | No |
| 81 | SRD5A1 |  |  | 2330 | 0.046 | 0.3835 | No |
| 82 | PITPNB |  |  | 2434 | 0.042 | 0.3748 | No |
| 83 | EDEM1 |  |  | 2443 | 0.042 | 0.3758 | No |
| 84 | GTF2H1 |  |  | 2503 | 0.040 | 0.3715 | No |
| 85 | SYTL2 |  |  | 2522 | 0.039 | 0.3714 | No |
| 86 | PSMC6 |  |  | 2532 | 0.038 | 0.3722 | No |
| 87 | PFKL |  |  | 2621 | 0.035 | 0.3648 | No |
| 88 | COPS5 |  |  | 2635 | 0.035 | 0.3650 | No |
| 89 | FDXR |  |  | 2642 | 0.035 | 0.3659 | No |
| 90 | SLC1A5 |  |  | 2665 | 0.034 | 0.3652 | No |
| 91 | UNG |  |  | 2727 | 0.032 | 0.3604 | No |
| 92 | POLR3G |  |  | 2811 | 0.030 | 0.3532 | No |
| 93 | SLC9A3R1 |  |  | 2862 | 0.029 | 0.3493 | No |
| 94 | BUB1 |  |  | 2888 | 0.028 | 0.3480 | No |
| 95 | ACTR2 |  |  | 2953 | 0.026 | 0.3426 | No |
| 96 | NFYC |  |  | 3111 | 0.022 | 0.3274 | No |
| 97 | RPA1 |  |  | 3239 | 0.020 | 0.3152 | No |
| 98 | GSR |  |  | 3274 | 0.019 | 0.3125 | No |
| 99 | PPP1R15A |  |  | 3296 | 0.018 | 0.3112 | No |
| 100 | NFKBIB |  |  | 3480 | 0.014 | 0.2929 | No |
| 101 | SORD |  |  | 3535 | 0.013 | 0.2880 | No |
| 102 | INSIG1 |  |  | 3553 | 0.013 | 0.2868 | No |
| 103 | HSPA4 |  |  | 3742 | 0.009 | 0.2678 | No |
| 104 | CORO1A |  |  | 3747 | 0.009 | 0.2678 | No |
| 105 | BCAT1 |  |  | 3755 | 0.009 | 0.2675 | No |
| 106 | SHMT2 |  |  | 3865 | 0.007 | 0.2566 | No |
| 107 | FKBP2 |  |  | 3905 | 0.006 | 0.2528 | No |
| 108 | NFIL3 |  |  | 4056 | 0.003 | 0.2375 | No |
| 109 | PSAT1 |  |  | 4318 | -0.001 | 0.2107 | No |
| 110 | RIT1 |  |  | 4461 | -0.004 | 0.1962 | No |
| 111 | GGA2 |  |  | 4534 | -0.005 | 0.1890 | No |
| 112 | STARD4 |  |  | 4548 | -0.005 | 0.1879 | No |
| 113 | NUFIP1 |  |  | 4665 | -0.007 | 0.1763 | No |
| 114 | LDLR |  |  | 4731 | -0.008 | 0.1699 | No |
| 115 | USO1 |  |  | 4771 | -0.008 | 0.1663 | No |
| 116 | IMMT |  |  | 4844 | -0.010 | 0.1593 | No |
| 117 | CCNF |  |  | 4868 | -0.010 | 0.1574 | No |
| 118 | ADIPOR2 |  |  | 4936 | -0.012 | 0.1510 | No |
| 119 | PHGDH |  |  | 5207 | -0.016 | 0.1239 | No |
| 120 | PGM1 |  |  | 5339 | -0.017 | 0.1112 | No |
| 121 | ASNS |  |  | 5370 | -0.018 | 0.1089 | No |
| 122 | XBP1 |  |  | 5510 | -0.020 | 0.0955 | No |
| 123 | ERO1A |  |  | 5520 | -0.020 | 0.0955 | No |
| 124 | LTA4H |  |  | 5586 | -0.022 | 0.0897 | No |
| 125 | GCLC |  |  | 5628 | -0.022 | 0.0865 | No |
| 126 | CDC25A |  |  | 5789 | -0.025 | 0.0711 | No |
| 127 | DHCR24 |  |  | 5843 | -0.026 | 0.0669 | No |
| 128 | BHLHE40 |  |  | 6102 | -0.031 | 0.0416 | No |
| 129 | NMT1 |  |  | 6122 | -0.031 | 0.0411 | No |
| 130 | IDI1 |  |  | 6154 | -0.032 | 0.0393 | No |
| 131 | RRM2 |  |  | 6214 | -0.033 | 0.0348 | No |
| 132 | MCM2 |  |  | 6282 | -0.034 | 0.0294 | No |
| 133 | ACLY |  |  | 6411 | -0.037 | 0.0179 | No |
| 134 | VLDLR |  |  | 6518 | -0.039 | 0.0087 | No |
| 135 | TBK1 |  |  | 6598 | -0.041 | 0.0024 | No |
| 136 | DDIT4 |  |  | 6713 | -0.043 | -0.0074 | No |
| 137 | CYP51A1 |  |  | 7249 | -0.057 | -0.0600 | No |
| 138 | GSK3B |  |  | 7351 | -0.059 | -0.0678 | No |
| 139 | IFRD1 |  |  | 7353 | -0.059 | -0.0652 | No |
| 140 | MTHFD2L |  |  | 7377 | -0.060 | -0.0649 | No |
| 141 | SERPINH1 |  |  | 7462 | -0.062 | -0.0707 | No |
| 142 | ACACA |  |  | 7654 | -0.068 | -0.0874 | No |
| 143 | MCM4 |  |  | 7658 | -0.068 | -0.0846 | No |
| 144 | SLC37A4 |  |  | 7693 | -0.069 | -0.0850 | No |
| 145 | SLC1A4 |  |  | 7745 | -0.072 | -0.0870 | No |
| 146 | STC1 |  |  | 7777 | -0.072 | -0.0869 | No |
| 147 | NUP205 |  |  | 7962 | -0.080 | -0.1023 | No |
| 148 | ELOVL5 |  |  | 8115 | -0.086 | -0.1141 | No |
| 149 | TMEM97 |  |  | 8161 | -0.088 | -0.1148 | No |
| 150 | SSR1 |  |  | 8183 | -0.089 | -0.1130 | No |
| 151 | SC5D |  |  | 8206 | -0.090 | -0.1112 | No |
| 152 | GLA |  |  | 8303 | -0.095 | -0.1168 | No |
| 153 | ADD3 |  |  | 8338 | -0.096 | -0.1160 | No |
| 154 | LGMN |  |  | 8379 | -0.098 | -0.1157 | No |
| 155 | PIK3R3 |  |  | 8411 | -0.100 | -0.1144 | No |
| 156 | TFRC |  |  | 8715 | -0.119 | -0.1403 | No |
| 157 | M6PR |  |  | 8772 | -0.123 | -0.1405 | No |
| 158 | P4HA1 |  |  | 8810 | -0.125 | -0.1387 | No |
| 159 | SKAP2 |  |  | 8827 | -0.126 | -0.1346 | No |
| 160 | SEC11A |  |  | 9002 | -0.143 | -0.1462 | No |
| 161 | FADS1 |  |  | 9044 | -0.147 | -0.1438 | No |
| 162 | FADS2 |  |  | 9078 | -0.150 | -0.1404 | No |
| 163 | SLC2A3 |  |  | 9156 | -0.159 | -0.1412 | No |
| 164 | PLOD2 |  |  | 9169 | -0.161 | -0.1351 | No |
| 165 | RPN1 |  |  | 9243 | -0.171 | -0.1349 | No |
| 166 | BTG2 |  |  | 9304 | -0.181 | -0.1330 | No |
| 167 | ACSL3 |  |  | 9338 | -0.187 | -0.1279 | No |
| 168 | HSP90B1 |  |  | 9365 | -0.193 | -0.1219 | No |
| 169 | RDH11 |  |  | 9422 | -0.205 | -0.1184 | No |
| 170 | TM7SF2 |  |  | 9450 | -0.213 | -0.1116 | No |
| 171 | HSPA5 |  |  | 9533 | -0.234 | -0.1095 | No |
| 172 | CALR |  |  | 9537 | -0.235 | -0.0993 | No |
| 173 | ATP2A2 |  |  | 9610 | -0.263 | -0.0948 | No |
| 174 | HMGCR |  |  | 9640 | -0.277 | -0.0853 | No |
| 175 | HMGCS1 |  |  | 9718 | -0.329 | -0.0785 | No |
| 176 | IDH1 |  |  | 9737 | -0.345 | -0.0648 | No |
| 177 | SCD |  |  | 9762 | -0.370 | -0.0506 | No |
| 178 | SQLE |  |  | 9790 | -0.408 | -0.0350 | No |
| 179 | CANX |  |  | 9804 | -0.431 | -0.0169 | No |
| 180 | CD9 |  |  | 9832 | -0.544 | 0.0048 | No |
Table: GSEA details [plain text format]

  

Fig 2: HALLMARK\_MTORC1\_SIGNALING: Random ES distribution      
 Gene set null distribution of ES for **HALLMARK\_MTORC1\_SIGNALING**

  
