# Supplementary material for: Modeling glioblastoma heterogeneity as a dynamic network of cell states
Source: Mol Syst Biol. 2021 Sep 16;17(9):e10105. doi: 10.15252/msb.202010105 (PMC8444284; doi:10.15252/msb.202010105)
Supplement: Supplementary file 5 — Source Data for Figure 3 [file MSB-17-e10105-s001.zip › Figure3A_sourcedata/GSEA_3065/hallmarks_state1.GseaPreranked.1623416262439/HALLMARK_MYC_TARGETS_V1.html]

Details for gene set HALLMARK\_MYC\_TARGETS\_V1[GSEA]

|  || Dataset | state1 |
| Phenotype | NoPhenotypeAvailable |
| Upregulated in class | na\_pos |
| GeneSet | HALLMARK\_MYC\_TARGETS\_V1 |
| Enrichment Score (ES) | 0.6777614 |
| Normalized Enrichment Score (NES) | 2.6968386 |
| Nominal p-value | 0.0 |
| FDR q-value | 0.0 |
| FWER p-Value | 0.0 |
Table: GSEA Results Summary

  

Fig 1: Enrichment plot: HALLMARK\_MYC\_TARGETS\_V1      
 Profile of the Running ES Score & Positions of GeneSet Members on the Rank Ordered List

  

| PROBE | GENE SYMBOL | GENE\_TITLE | RANK IN GENE LIST | RANK METRIC SCORE | RUNNING ES | CORE ENRICHMENT || 1 | ODC1 |  |  | 7 | 0.744 | 0.0281 | Yes |
| 2 | SRM |  |  | 44 | 0.493 | 0.0435 | Yes |
| 3 | LDHA |  |  | 80 | 0.394 | 0.0552 | Yes |
| 4 | PSMA7 |  |  | 103 | 0.366 | 0.0671 | Yes |
| 5 | COX5A |  |  | 116 | 0.348 | 0.0793 | Yes |
| 6 | RPS2 |  |  | 124 | 0.342 | 0.0919 | Yes |
| 7 | NPM1 |  |  | 129 | 0.335 | 0.1044 | Yes |
| 8 | PPIA |  |  | 150 | 0.324 | 0.1149 | Yes |
| 9 | RPS6 |  |  | 151 | 0.321 | 0.1274 | Yes |
| 10 | RANBP1 |  |  | 154 | 0.320 | 0.1396 | Yes |
| 11 | RAN |  |  | 194 | 0.297 | 0.1471 | Yes |
| 12 | EEF1B2 |  |  | 197 | 0.296 | 0.1584 | Yes |
| 13 | RPS3 |  |  | 222 | 0.287 | 0.1670 | Yes |
| 14 | POLD2 |  |  | 241 | 0.280 | 0.1760 | Yes |
| 15 | C1QBP |  |  | 261 | 0.275 | 0.1847 | Yes |
| 16 | SLC25A3 |  |  | 275 | 0.269 | 0.1938 | Yes |
| 17 | SNRPG |  |  | 287 | 0.264 | 0.2029 | Yes |
| 18 | HPRT1 |  |  | 289 | 0.263 | 0.2130 | Yes |
| 19 | DEK |  |  | 296 | 0.261 | 0.2224 | Yes |
| 20 | PSMD8 |  |  | 298 | 0.260 | 0.2324 | Yes |
| 21 | RPL14 |  |  | 299 | 0.260 | 0.2425 | Yes |
| 22 | RPS5 |  |  | 300 | 0.260 | 0.2526 | Yes |
| 23 | PPM1G |  |  | 353 | 0.239 | 0.2565 | Yes |
| 24 | PTGES3 |  |  | 359 | 0.237 | 0.2652 | Yes |
| 25 | RPL18 |  |  | 383 | 0.230 | 0.2717 | Yes |
| 26 | UBE2L3 |  |  | 388 | 0.229 | 0.2802 | Yes |
| 27 | NME1 |  |  | 395 | 0.227 | 0.2884 | Yes |
| 28 | RPL22 |  |  | 396 | 0.227 | 0.2972 | Yes |
| 29 | HSPD1 |  |  | 397 | 0.227 | 0.3059 | Yes |
| 30 | TXNL4A |  |  | 401 | 0.226 | 0.3144 | Yes |
| 31 | CCT5 |  |  | 440 | 0.218 | 0.3189 | Yes |
| 32 | PSMB3 |  |  | 444 | 0.218 | 0.3271 | Yes |
| 33 | RPS10 |  |  | 450 | 0.215 | 0.3349 | Yes |
| 34 | RPLP0 |  |  | 452 | 0.215 | 0.3431 | Yes |
| 35 | PSMB2 |  |  | 456 | 0.214 | 0.3511 | Yes |
| 36 | CNBP |  |  | 465 | 0.212 | 0.3585 | Yes |
| 37 | EIF4G2 |  |  | 466 | 0.212 | 0.3667 | Yes |
| 38 | CDC20 |  |  | 469 | 0.212 | 0.3747 | Yes |
| 39 | TCP1 |  |  | 475 | 0.211 | 0.3824 | Yes |
| 40 | SNRPD2 |  |  | 496 | 0.206 | 0.3883 | Yes |
| 41 | RACK1 |  |  | 510 | 0.204 | 0.3949 | Yes |
| 42 | CYC1 |  |  | 511 | 0.203 | 0.4028 | Yes |
| 43 | HDGF |  |  | 512 | 0.203 | 0.4107 | Yes |
| 44 | SERBP1 |  |  | 516 | 0.202 | 0.4182 | Yes |
| 45 | HSP90AB1 |  |  | 521 | 0.201 | 0.4255 | Yes |
| 46 | NHP2 |  |  | 537 | 0.198 | 0.4317 | Yes |
| 47 | LSM7 |  |  | 540 | 0.198 | 0.4391 | Yes |
| 48 | HSPE1 |  |  | 545 | 0.197 | 0.4463 | Yes |
| 49 | CCT7 |  |  | 558 | 0.193 | 0.4526 | Yes |
| 50 | PSMA1 |  |  | 563 | 0.192 | 0.4596 | Yes |
| 51 | RPL6 |  |  | 572 | 0.190 | 0.4661 | Yes |
| 52 | VDAC3 |  |  | 590 | 0.187 | 0.4716 | Yes |
| 53 | EIF2S2 |  |  | 610 | 0.184 | 0.4768 | Yes |
| 54 | G3BP1 |  |  | 611 | 0.184 | 0.4839 | Yes |
| 55 | SNRPD3 |  |  | 616 | 0.183 | 0.4906 | Yes |
| 56 | YWHAQ |  |  | 618 | 0.183 | 0.4976 | Yes |
| 57 | EIF1AX |  |  | 624 | 0.182 | 0.5041 | Yes |
| 58 | RSL1D1 |  |  | 627 | 0.182 | 0.5110 | Yes |
| 59 | SET |  |  | 634 | 0.181 | 0.5174 | Yes |
| 60 | SNRPD1 |  |  | 647 | 0.179 | 0.5231 | Yes |
| 61 | PSMD7 |  |  | 724 | 0.168 | 0.5218 | Yes |
| 62 | ILF2 |  |  | 729 | 0.167 | 0.5278 | Yes |
| 63 | VDAC1 |  |  | 732 | 0.167 | 0.5341 | Yes |
| 64 | LSM2 |  |  | 734 | 0.167 | 0.5405 | Yes |
| 65 | EIF4H |  |  | 759 | 0.164 | 0.5444 | Yes |
| 66 | PA2G4 |  |  | 780 | 0.160 | 0.5485 | Yes |
| 67 | SSBP1 |  |  | 783 | 0.160 | 0.5545 | Yes |
| 68 | PSMC4 |  |  | 803 | 0.157 | 0.5586 | Yes |
| 69 | ERH |  |  | 807 | 0.156 | 0.5643 | Yes |
| 70 | PSMA4 |  |  | 837 | 0.153 | 0.5673 | Yes |
| 71 | HDDC2 |  |  | 844 | 0.152 | 0.5725 | Yes |
| 72 | GLO1 |  |  | 855 | 0.151 | 0.5774 | Yes |
| 73 | CCT4 |  |  | 858 | 0.150 | 0.5830 | Yes |
| 74 | PGK1 |  |  | 875 | 0.148 | 0.5870 | Yes |
| 75 | NDUFAB1 |  |  | 887 | 0.146 | 0.5915 | Yes |
| 76 | MRPL9 |  |  | 949 | 0.140 | 0.5907 | Yes |
| 77 | PHB2 |  |  | 985 | 0.135 | 0.5923 | Yes |
| 78 | ACP1 |  |  | 1000 | 0.134 | 0.5960 | Yes |
| 79 | PRDX3 |  |  | 1003 | 0.133 | 0.6010 | Yes |
| 80 | RPL34 |  |  | 1023 | 0.131 | 0.6041 | Yes |
| 81 | XRCC6 |  |  | 1025 | 0.131 | 0.6091 | Yes |
| 82 | SNRPA1 |  |  | 1032 | 0.130 | 0.6135 | Yes |
| 83 | VBP1 |  |  | 1044 | 0.129 | 0.6174 | Yes |
| 84 | PCBP1 |  |  | 1057 | 0.127 | 0.6211 | Yes |
| 85 | IMPDH2 |  |  | 1060 | 0.127 | 0.6258 | Yes |
| 86 | RRP9 |  |  | 1072 | 0.125 | 0.6295 | Yes |
| 87 | AP3S1 |  |  | 1075 | 0.125 | 0.6341 | Yes |
| 88 | SRSF3 |  |  | 1076 | 0.125 | 0.6389 | Yes |
| 89 | ETF1 |  |  | 1121 | 0.120 | 0.6391 | Yes |
| 90 | NOP16 |  |  | 1154 | 0.118 | 0.6403 | Yes |
| 91 | HNRNPC |  |  | 1184 | 0.114 | 0.6417 | Yes |
| 92 | PSMA2 |  |  | 1218 | 0.111 | 0.6426 | Yes |
| 93 | HNRNPR |  |  | 1231 | 0.110 | 0.6456 | Yes |
| 94 | EIF2S1 |  |  | 1243 | 0.109 | 0.6487 | Yes |
| 95 | EIF3D |  |  | 1251 | 0.108 | 0.6522 | Yes |
| 96 | PSMD3 |  |  | 1252 | 0.108 | 0.6564 | Yes |
| 97 | GNL3 |  |  | 1256 | 0.108 | 0.6602 | Yes |
| 98 | HNRNPA1 |  |  | 1313 | 0.102 | 0.6584 | Yes |
| 99 | TFDP1 |  |  | 1318 | 0.102 | 0.6619 | Yes |
| 100 | UBA2 |  |  | 1380 | 0.097 | 0.6594 | Yes |
| 101 | MAD2L1 |  |  | 1394 | 0.095 | 0.6618 | Yes |
| 102 | MRPS18B |  |  | 1398 | 0.095 | 0.6651 | Yes |
| 103 | CCT3 |  |  | 1412 | 0.094 | 0.6675 | Yes |
| 104 | PABPC4 |  |  | 1472 | 0.091 | 0.6649 | Yes |
| 105 | YWHAE |  |  | 1494 | 0.090 | 0.6662 | Yes |
| 106 | TYMS |  |  | 1501 | 0.089 | 0.6690 | Yes |
| 107 | PABPC1 |  |  | 1502 | 0.089 | 0.6725 | Yes |
| 108 | HNRNPD |  |  | 1509 | 0.088 | 0.6753 | Yes |
| 109 | AIMP2 |  |  | 1560 | 0.085 | 0.6734 | Yes |
| 110 | FBL |  |  | 1628 | 0.080 | 0.6696 | Yes |
| 111 | SNRPB2 |  |  | 1633 | 0.080 | 0.6723 | Yes |
| 112 | PHB |  |  | 1652 | 0.079 | 0.6735 | Yes |
| 113 | POLE3 |  |  | 1657 | 0.078 | 0.6761 | Yes |
| 114 | MRPL23 |  |  | 1682 | 0.076 | 0.6766 | Yes |
| 115 | NAP1L1 |  |  | 1717 | 0.074 | 0.6759 | Yes |
| 116 | EIF4A1 |  |  | 1764 | 0.071 | 0.6740 | Yes |
| 117 | ABCE1 |  |  | 1790 | 0.070 | 0.6741 | Yes |
| 118 | GSPT1 |  |  | 1797 | 0.070 | 0.6762 | Yes |
| 119 | SYNCRIP |  |  | 1817 | 0.069 | 0.6769 | Yes |
| 120 | CCNA2 |  |  | 1835 | 0.068 | 0.6778 | Yes |
| 121 | TRIM28 |  |  | 1867 | 0.067 | 0.6771 | No |
| 122 | SRPK1 |  |  | 1900 | 0.065 | 0.6764 | No |
| 123 | USP1 |  |  | 1921 | 0.064 | 0.6768 | No |
| 124 | PRPF31 |  |  | 1940 | 0.063 | 0.6774 | No |
| 125 | HNRNPA3 |  |  | 1989 | 0.060 | 0.6747 | No |
| 126 | CDK4 |  |  | 2000 | 0.060 | 0.6760 | No |
| 127 | PSMD14 |  |  | 2025 | 0.058 | 0.6758 | No |
| 128 | FAM120A |  |  | 2038 | 0.058 | 0.6768 | No |
| 129 | RUVBL2 |  |  | 2064 | 0.057 | 0.6764 | No |
| 130 | EIF3B |  |  | 2079 | 0.056 | 0.6771 | No |
| 131 | KPNB1 |  |  | 2179 | 0.052 | 0.6689 | No |
| 132 | EIF3J |  |  | 2271 | 0.048 | 0.6614 | No |
| 133 | RNPS1 |  |  | 2347 | 0.045 | 0.6554 | No |
| 134 | PSMD1 |  |  | 2352 | 0.045 | 0.6567 | No |
| 135 | DDX21 |  |  | 2406 | 0.043 | 0.6529 | No |
| 136 | NOLC1 |  |  | 2417 | 0.043 | 0.6536 | No |
| 137 | GOT2 |  |  | 2496 | 0.040 | 0.6470 | No |
| 138 | EIF4E |  |  | 2517 | 0.039 | 0.6465 | No |
| 139 | PSMC6 |  |  | 2532 | 0.038 | 0.6465 | No |
| 140 | PRPS2 |  |  | 2571 | 0.038 | 0.6441 | No |
| 141 | CTPS1 |  |  | 2594 | 0.036 | 0.6432 | No |
| 142 | SNRPA |  |  | 2597 | 0.036 | 0.6444 | No |
| 143 | COPS5 |  |  | 2635 | 0.035 | 0.6420 | No |
| 144 | EXOSC7 |  |  | 2646 | 0.035 | 0.6423 | No |
| 145 | RAD23B |  |  | 2654 | 0.034 | 0.6429 | No |
| 146 | TOMM70 |  |  | 2664 | 0.034 | 0.6433 | No |
| 147 | HDAC2 |  |  | 2724 | 0.032 | 0.6384 | No |
| 148 | DUT |  |  | 3005 | 0.025 | 0.6105 | No |
| 149 | STARD7 |  |  | 3048 | 0.024 | 0.6070 | No |
| 150 | ORC2 |  |  | 3085 | 0.023 | 0.6042 | No |
| 151 | APEX1 |  |  | 3102 | 0.022 | 0.6034 | No |
| 152 | CSTF2 |  |  | 3248 | 0.019 | 0.5892 | No |
| 153 | CUL1 |  |  | 3336 | 0.017 | 0.5809 | No |
| 154 | PCNA |  |  | 3540 | 0.013 | 0.5604 | No |
| 155 | SRSF2 |  |  | 3783 | 0.008 | 0.5358 | No |
| 156 | PWP1 |  |  | 3940 | 0.005 | 0.5199 | No |
| 157 | PSMA6 |  |  | 3954 | 0.005 | 0.5187 | No |
| 158 | SF3A1 |  |  | 3962 | 0.005 | 0.5182 | No |
| 159 | MYC |  |  | 3964 | 0.005 | 0.5183 | No |
| 160 | SRSF7 |  |  | 4413 | -0.003 | 0.4722 | No |
| 161 | BUB3 |  |  | 4828 | -0.010 | 0.4298 | No |
| 162 | XPOT |  |  | 5007 | -0.013 | 0.4119 | No |
| 163 | TUFM |  |  | 5247 | -0.016 | 0.3878 | No |
| 164 | TRA2B |  |  | 5321 | -0.017 | 0.3810 | No |
| 165 | KPNA2 |  |  | 5461 | -0.020 | 0.3674 | No |
| 166 | CCT2 |  |  | 5479 | -0.020 | 0.3664 | No |
| 167 | TARDBP |  |  | 5538 | -0.021 | 0.3612 | No |
| 168 | MCM5 |  |  | 5588 | -0.022 | 0.3570 | No |
| 169 | CDK2 |  |  | 5964 | -0.029 | 0.3194 | No |
| 170 | CAD |  |  | 6013 | -0.029 | 0.3156 | No |
| 171 | DHX15 |  |  | 6090 | -0.031 | 0.3089 | No |
| 172 | HNRNPA2B1 |  |  | 6142 | -0.032 | 0.3049 | No |
| 173 | NCBP1 |  |  | 6162 | -0.032 | 0.3041 | No |
| 174 | NOP56 |  |  | 6205 | -0.033 | 0.3011 | No |
| 175 | MCM2 |  |  | 6282 | -0.034 | 0.2946 | No |
| 176 | SRSF1 |  |  | 6896 | -0.047 | 0.2331 | No |
| 177 | SMARCC1 |  |  | 6990 | -0.050 | 0.2254 | No |
| 178 | CLNS1A |  |  | 7008 | -0.050 | 0.2256 | No |
| 179 | SF3B3 |  |  | 7232 | -0.056 | 0.2048 | No |
| 180 | SSB |  |  | 7242 | -0.056 | 0.2060 | No |
| 181 | IFRD1 |  |  | 7353 | -0.059 | 0.1970 | No |
| 182 | MCM7 |  |  | 7397 | -0.061 | 0.1949 | No |
| 183 | MCM6 |  |  | 7414 | -0.061 | 0.1956 | No |
| 184 | RRM1 |  |  | 7556 | -0.065 | 0.1836 | No |
| 185 | NCBP2 |  |  | 7645 | -0.068 | 0.1771 | No |
| 186 | MCM4 |  |  | 7658 | -0.068 | 0.1785 | No |
| 187 | CDC45 |  |  | 7751 | -0.072 | 0.1718 | No |
| 188 | XPO1 |  |  | 7863 | -0.076 | 0.1633 | No |
| 189 | CBX3 |  |  | 7976 | -0.080 | 0.1548 | No |
| 190 | UBE2E1 |  |  | 8237 | -0.091 | 0.1315 | No |
| 191 | RFC4 |  |  | 8292 | -0.094 | 0.1296 | No |
| 192 | HNRNPU |  |  | 8616 | -0.112 | 0.1006 | No |
| 193 | DDX18 |  |  | 8676 | -0.116 | 0.0990 | No |
| 194 | PRDX4 |  |  | 9457 | -0.215 | 0.0268 | No |
| 195 | CANX |  |  | 9804 | -0.431 | 0.0077 | No |
Table: GSEA details [plain text format]

  

Fig 2: HALLMARK\_MYC\_TARGETS\_V1: Random ES distribution      
 Gene set null distribution of ES for **HALLMARK\_MYC\_TARGETS\_V1**

  
