# Supplementary material for: Modeling glioblastoma heterogeneity as a dynamic network of cell states
Source: Mol Syst Biol. 2021 Sep 16;17(9):e10105. doi: 10.15252/msb.202010105 (PMC8444284; doi:10.15252/msb.202010105)
Supplement: Supplementary file 5 — Source Data for Figure 3 [file MSB-17-e10105-s001.zip › Figure3A_sourcedata/GSEA_3065/hallmarks_state1.GseaPreranked.1623416262439/HALLMARK_MYC_TARGETS_V2.html]

Details for gene set HALLMARK\_MYC\_TARGETS\_V2[GSEA]

|  || Dataset | state1 |
| Phenotype | NoPhenotypeAvailable |
| Upregulated in class | na\_pos |
| GeneSet | HALLMARK\_MYC\_TARGETS\_V2 |
| Enrichment Score (ES) | 0.5473719 |
| Normalized Enrichment Score (NES) | 1.7740992 |
| Nominal p-value | 0.0022675737 |
| FDR q-value | 0.007895333 |
| FWER p-Value | 0.02 |
Table: GSEA Results Summary

  

Fig 1: Enrichment plot: HALLMARK\_MYC\_TARGETS\_V2      
 Profile of the Running ES Score & Positions of GeneSet Members on the Rank Ordered List

  

| PROBE | GENE SYMBOL | GENE\_TITLE | RANK IN GENE LIST | RANK METRIC SCORE | RUNNING ES | CORE ENRICHMENT || 1 | SRM |  |  | 44 | 0.493 | 0.1259 | Yes |
| 2 | NPM1 |  |  | 129 | 0.335 | 0.2060 | Yes |
| 3 | HSPD1 |  |  | 397 | 0.227 | 0.2388 | Yes |
| 4 | HSPE1 |  |  | 545 | 0.197 | 0.2760 | Yes |
| 5 | DCTPP1 |  |  | 566 | 0.191 | 0.3244 | Yes |
| 6 | PA2G4 |  |  | 780 | 0.160 | 0.3451 | Yes |
| 7 | RRP9 |  |  | 1072 | 0.125 | 0.3485 | Yes |
| 8 | NOP16 |  |  | 1154 | 0.118 | 0.3714 | Yes |
| 9 | GNL3 |  |  | 1256 | 0.108 | 0.3896 | Yes |
| 10 | NDUFAF4 |  |  | 1276 | 0.106 | 0.4158 | Yes |
| 11 | PLK1 |  |  | 1403 | 0.095 | 0.4281 | Yes |
| 12 | TBRG4 |  |  | 1442 | 0.093 | 0.4487 | Yes |
| 13 | FARSA |  |  | 1487 | 0.090 | 0.4681 | Yes |
| 14 | AIMP2 |  |  | 1560 | 0.085 | 0.4833 | Yes |
| 15 | PHB |  |  | 1652 | 0.079 | 0.4949 | Yes |
| 16 | WDR74 |  |  | 1767 | 0.071 | 0.5021 | Yes |
| 17 | GRWD1 |  |  | 1976 | 0.061 | 0.4970 | Yes |
| 18 | CDK4 |  |  | 2000 | 0.060 | 0.5104 | Yes |
| 19 | BYSL |  |  | 2007 | 0.059 | 0.5255 | Yes |
| 20 | IMP4 |  |  | 2051 | 0.057 | 0.5363 | Yes |
| 21 | PES1 |  |  | 2288 | 0.048 | 0.5248 | Yes |
| 22 | EXOSC5 |  |  | 2296 | 0.047 | 0.5367 | Yes |
| 23 | NIP7 |  |  | 2395 | 0.044 | 0.5382 | Yes |
| 24 | NOLC1 |  |  | 2417 | 0.043 | 0.5474 | Yes |
| 25 | PPAN |  |  | 2668 | 0.034 | 0.5309 | No |
| 26 | UNG |  |  | 2727 | 0.032 | 0.5335 | No |
| 27 | MRTO4 |  |  | 2949 | 0.026 | 0.5179 | No |
| 28 | WDR43 |  |  | 3272 | 0.019 | 0.4901 | No |
| 29 | RABEPK |  |  | 3433 | 0.015 | 0.4779 | No |
| 30 | SORD |  |  | 3535 | 0.013 | 0.4711 | No |
| 31 | PUS1 |  |  | 3756 | 0.009 | 0.4510 | No |
| 32 | TFB2M |  |  | 3770 | 0.009 | 0.4520 | No |
| 33 | PPRC1 |  |  | 3863 | 0.007 | 0.4445 | No |
| 34 | MYC |  |  | 3964 | 0.005 | 0.4357 | No |
| 35 | SUPV3L1 |  |  | 3969 | 0.005 | 0.4365 | No |
| 36 | TCOF1 |  |  | 4010 | 0.004 | 0.4336 | No |
| 37 | NOC4L |  |  | 4215 | 0.001 | 0.4129 | No |
| 38 | PRMT3 |  |  | 4403 | -0.003 | 0.3947 | No |
| 39 | LAS1L |  |  | 4747 | -0.008 | 0.3619 | No |
| 40 | MYBBP1A |  |  | 5147 | -0.015 | 0.3252 | No |
| 41 | PLK4 |  |  | 5414 | -0.019 | 0.3031 | No |
| 42 | MCM5 |  |  | 5588 | -0.022 | 0.2912 | No |
| 43 | MPHOSPH10 |  |  | 5740 | -0.024 | 0.2822 | No |
| 44 | RRP12 |  |  | 5826 | -0.026 | 0.2804 | No |
| 45 | NOP56 |  |  | 6205 | -0.033 | 0.2507 | No |
| 46 | SLC19A1 |  |  | 7027 | -0.051 | 0.1806 | No |
| 47 | UTP20 |  |  | 7402 | -0.061 | 0.1586 | No |
| 48 | MCM4 |  |  | 7658 | -0.068 | 0.1506 | No |
| 49 | CBX3 |  |  | 7976 | -0.080 | 0.1396 | No |
| 50 | TMEM97 |  |  | 8161 | -0.088 | 0.1441 | No |
| 51 | DDX18 |  |  | 8676 | -0.116 | 0.1224 | No |
Table: GSEA details [plain text format]

  

Fig 2: HALLMARK\_MYC\_TARGETS\_V2: Random ES distribution      
 Gene set null distribution of ES for **HALLMARK\_MYC\_TARGETS\_V2**

  
