# Supplementary material for: Modeling glioblastoma heterogeneity as a dynamic network of cell states
Source: Mol Syst Biol. 2021 Sep 16;17(9):e10105. doi: 10.15252/msb.202010105 (PMC8444284; doi:10.15252/msb.202010105)
Supplement: Supplementary file 5 — Source Data for Figure 3 [file MSB-17-e10105-s001.zip › Figure3A_sourcedata/GSEA_3065/hallmarks_state1.GseaPreranked.1623416262439/HALLMARK_MYOGENESIS.html]

Details for gene set HALLMARK\_MYOGENESIS[GSEA]

|  || Dataset | state1 |
| Phenotype | NoPhenotypeAvailable |
| Upregulated in class | na\_neg |
| GeneSet | HALLMARK\_MYOGENESIS |
| Enrichment Score (ES) | -0.34684154 |
| Normalized Enrichment Score (NES) | -1.2317494 |
| Nominal p-value | 0.107334524 |
| FDR q-value | 0.35847655 |
| FWER p-Value | 0.955 |
Table: GSEA Results Summary

  

Fig 1: Enrichment plot: HALLMARK\_MYOGENESIS      
 Profile of the Running ES Score & Positions of GeneSet Members on the Rank Ordered List

  

| PROBE | GENE SYMBOL | GENE\_TITLE | RANK IN GENE LIST | RANK METRIC SCORE | RUNNING ES | CORE ENRICHMENT || 1 | NQO1 |  |  | 10 | 0.693 | 0.0473 | No |
| 2 | IGFBP3 |  |  | 15 | 0.664 | 0.0931 | No |
| 3 | VIPR1 |  |  | 42 | 0.501 | 0.1254 | No |
| 4 | MYH9 |  |  | 140 | 0.328 | 0.1383 | No |
| 5 | TAGLN |  |  | 147 | 0.326 | 0.1604 | No |
| 6 | GSN |  |  | 213 | 0.290 | 0.1740 | No |
| 7 | TPM3 |  |  | 225 | 0.286 | 0.1928 | No |
| 8 | MYLK |  |  | 260 | 0.275 | 0.2085 | No |
| 9 | CDKN1A |  |  | 262 | 0.274 | 0.2275 | No |
| 10 | PDLIM7 |  |  | 644 | 0.180 | 0.2011 | No |
| 11 | IGFBP7 |  |  | 658 | 0.177 | 0.2121 | No |
| 12 | COL6A2 |  |  | 661 | 0.177 | 0.2243 | No |
| 13 | SVIL |  |  | 676 | 0.175 | 0.2350 | No |
| 14 | CNN3 |  |  | 727 | 0.168 | 0.2416 | No |
| 15 | TPM2 |  |  | 753 | 0.165 | 0.2506 | No |
| 16 | STC2 |  |  | 922 | 0.142 | 0.2434 | No |
| 17 | ACSL1 |  |  | 962 | 0.138 | 0.2490 | No |
| 18 | WWTR1 |  |  | 1009 | 0.133 | 0.2536 | No |
| 19 | CKB |  |  | 1368 | 0.098 | 0.2238 | No |
| 20 | SOD3 |  |  | 1405 | 0.095 | 0.2267 | No |
| 21 | BIN1 |  |  | 1406 | 0.095 | 0.2333 | No |
| 22 | MYO1C |  |  | 1500 | 0.089 | 0.2300 | No |
| 23 | CRAT |  |  | 1515 | 0.088 | 0.2347 | No |
| 24 | BAG1 |  |  | 1614 | 0.082 | 0.2304 | No |
| 25 | GABARAPL2 |  |  | 1782 | 0.071 | 0.2183 | No |
| 26 | MYL6B |  |  | 2034 | 0.058 | 0.1966 | No |
| 27 | TGFB1 |  |  | 2040 | 0.058 | 0.2001 | No |
| 28 | FLII |  |  | 2136 | 0.053 | 0.1941 | No |
| 29 | SPEG |  |  | 2157 | 0.053 | 0.1957 | No |
| 30 | GADD45B |  |  | 2252 | 0.049 | 0.1895 | No |
| 31 | LPIN1 |  |  | 2380 | 0.044 | 0.1796 | No |
| 32 | KIFC3 |  |  | 2398 | 0.043 | 0.1809 | No |
| 33 | SH3BGR |  |  | 2572 | 0.037 | 0.1658 | No |
| 34 | FKBP1B |  |  | 2785 | 0.031 | 0.1463 | No |
| 35 | OCEL1 |  |  | 2824 | 0.030 | 0.1445 | No |
| 36 | MAPRE3 |  |  | 3440 | 0.015 | 0.0827 | No |
| 37 | PICK1 |  |  | 3486 | 0.014 | 0.0790 | No |
| 38 | MRAS |  |  | 3667 | 0.010 | 0.0614 | No |
| 39 | SMTN |  |  | 3761 | 0.009 | 0.0525 | No |
| 40 | SORBS3 |  |  | 3765 | 0.009 | 0.0528 | No |
| 41 | TEAD4 |  |  | 3907 | 0.006 | 0.0388 | No |
| 42 | ITGB1 |  |  | 3956 | 0.005 | 0.0342 | No |
| 43 | MAPK12 |  |  | 3963 | 0.005 | 0.0340 | No |
| 44 | DAPK2 |  |  | 3978 | 0.005 | 0.0329 | No |
| 45 | SYNGR2 |  |  | 4130 | 0.002 | 0.0176 | No |
| 46 | DMD |  |  | 4140 | 0.002 | 0.0168 | No |
| 47 | FDPS |  |  | 4450 | -0.004 | -0.0146 | No |
| 48 | RIT1 |  |  | 4461 | -0.004 | -0.0153 | No |
| 49 | FGF2 |  |  | 4570 | -0.006 | -0.0260 | No |
| 50 | PSEN2 |  |  | 5019 | -0.013 | -0.0709 | No |
| 51 | SH2B1 |  |  | 5027 | -0.013 | -0.0707 | No |
| 52 | MEF2D |  |  | 5259 | -0.016 | -0.0932 | No |
| 53 | CHRNB1 |  |  | 5367 | -0.018 | -0.1029 | No |
| 54 | PKIA |  |  | 5506 | -0.020 | -0.1156 | No |
| 55 | EFS |  |  | 5642 | -0.023 | -0.1278 | No |
| 56 | MEF2A |  |  | 5885 | -0.027 | -0.1507 | No |
| 57 | DTNA |  |  | 6001 | -0.029 | -0.1604 | No |
| 58 | LARGE1 |  |  | 6016 | -0.029 | -0.1598 | No |
| 59 | BHLHE40 |  |  | 6102 | -0.031 | -0.1663 | No |
| 60 | HDAC5 |  |  | 6550 | -0.039 | -0.2093 | No |
| 61 | SCHIP1 |  |  | 6575 | -0.040 | -0.2089 | No |
| 62 | PRNP |  |  | 6661 | -0.042 | -0.2147 | No |
| 63 | KCNH2 |  |  | 6689 | -0.043 | -0.2145 | No |
| 64 | DMPK |  |  | 6879 | -0.047 | -0.2305 | No |
| 65 | REEP1 |  |  | 6897 | -0.047 | -0.2290 | No |
| 66 | SSPN |  |  | 6940 | -0.048 | -0.2299 | No |
| 67 | IFRD1 |  |  | 7353 | -0.059 | -0.2679 | No |
| 68 | PFKM |  |  | 7421 | -0.061 | -0.2705 | No |
| 69 | ABLIM1 |  |  | 7806 | -0.074 | -0.3046 | No |
| 70 | AGL |  |  | 7897 | -0.077 | -0.3084 | No |
| 71 | ADCY9 |  |  | 8107 | -0.086 | -0.3238 | No |
| 72 | EIF4A2 |  |  | 8210 | -0.090 | -0.3280 | No |
| 73 | AKT2 |  |  | 8356 | -0.097 | -0.3361 | No |
| 74 | PDE4DIP |  |  | 8453 | -0.102 | -0.3388 | No |
| 75 | SORBS1 |  |  | 8508 | -0.106 | -0.3369 | No |
| 76 | MEF2C |  |  | 8606 | -0.112 | -0.3390 | Yes |
| 77 | ITGA7 |  |  | 8639 | -0.114 | -0.3344 | Yes |
| 78 | ADAM12 |  |  | 8684 | -0.116 | -0.3308 | Yes |
| 79 | GAA |  |  | 8798 | -0.124 | -0.3337 | Yes |
| 80 | ITGB4 |  |  | 8832 | -0.127 | -0.3282 | Yes |
| 81 | PLXNB2 |  |  | 8857 | -0.129 | -0.3217 | Yes |
| 82 | AEBP1 |  |  | 8877 | -0.131 | -0.3144 | Yes |
| 83 | SLC6A8 |  |  | 9101 | -0.152 | -0.3266 | Yes |
| 84 | SPTAN1 |  |  | 9199 | -0.165 | -0.3250 | Yes |
| 85 | TSC2 |  |  | 9271 | -0.175 | -0.3201 | Yes |
| 86 | EPHB3 |  |  | 9329 | -0.186 | -0.3130 | Yes |
| 87 | SIRT2 |  |  | 9386 | -0.198 | -0.3049 | Yes |
| 88 | FHL1 |  |  | 9398 | -0.200 | -0.2920 | Yes |
| 89 | NAV2 |  |  | 9505 | -0.227 | -0.2871 | Yes |
| 90 | ATP6AP1 |  |  | 9582 | -0.254 | -0.2772 | Yes |
| 91 | ERBB3 |  |  | 9601 | -0.258 | -0.2610 | Yes |
| 92 | ITGB5 |  |  | 9676 | -0.296 | -0.2480 | Yes |
| 93 | CRYAB |  |  | 9722 | -0.333 | -0.2294 | Yes |
| 94 | AGRN |  |  | 9726 | -0.335 | -0.2063 | Yes |
| 95 | SPARC |  |  | 9738 | -0.347 | -0.1832 | Yes |
| 96 | SCD |  |  | 9762 | -0.370 | -0.1598 | Yes |
| 97 | NOTCH1 |  |  | 9780 | -0.391 | -0.1343 | Yes |
| 98 | COL4A2 |  |  | 9786 | -0.402 | -0.1069 | Yes |
| 99 | CLU |  |  | 9795 | -0.418 | -0.0785 | Yes |
| 100 | APP |  |  | 9819 | -0.495 | -0.0463 | Yes |
| 101 | NCAM1 |  |  | 9862 | -0.751 | 0.0017 | Yes |
Table: GSEA details [plain text format]

  

Fig 2: HALLMARK\_MYOGENESIS: Random ES distribution      
 Gene set null distribution of ES for **HALLMARK\_MYOGENESIS**

  
