# Supplementary material for: Modeling glioblastoma heterogeneity as a dynamic network of cell states
Source: Mol Syst Biol. 2021 Sep 16;17(9):e10105. doi: 10.15252/msb.202010105 (PMC8444284; doi:10.15252/msb.202010105)
Supplement: Supplementary file 5 — Source Data for Figure 3 [file MSB-17-e10105-s001.zip › Figure3A_sourcedata/GSEA_3065/hallmarks_state1.GseaPreranked.1623416262439/HALLMARK_NOTCH_SIGNALING.html]

Details for gene set HALLMARK\_NOTCH\_SIGNALING[GSEA]

|  || Dataset | state1 |
| Phenotype | NoPhenotypeAvailable |
| Upregulated in class | na\_neg |
| GeneSet | HALLMARK\_NOTCH\_SIGNALING |
| Enrichment Score (ES) | -0.4434706 |
| Normalized Enrichment Score (NES) | -1.2057317 |
| Nominal p-value | 0.21252371 |
| FDR q-value | 0.3577245 |
| FWER p-Value | 0.977 |
Table: GSEA Results Summary

  

Fig 1: Enrichment plot: HALLMARK\_NOTCH\_SIGNALING      
 Profile of the Running ES Score & Positions of GeneSet Members on the Rank Ordered List

  

| PROBE | GENE SYMBOL | GENE\_TITLE | RANK IN GENE LIST | RANK METRIC SCORE | RUNNING ES | CORE ENRICHMENT || 1 | CCND1 |  |  | 29 | 0.566 | 0.1835 | No |
| 2 | RBX1 |  |  | 389 | 0.228 | 0.2223 | No |
| 3 | WNT5A |  |  | 1503 | 0.089 | 0.1387 | No |
| 4 | SKP1 |  |  | 1999 | 0.060 | 0.1082 | No |
| 5 | SAP30 |  |  | 2022 | 0.059 | 0.1253 | No |
| 6 | CUL1 |  |  | 3336 | 0.017 | -0.0022 | No |
| 7 | DTX2 |  |  | 3600 | 0.012 | -0.0251 | No |
| 8 | KAT2A |  |  | 4427 | -0.003 | -0.1078 | No |
| 9 | APH1A |  |  | 4452 | -0.004 | -0.1090 | No |
| 10 | FZD7 |  |  | 4561 | -0.006 | -0.1181 | No |
| 11 | TCF7L2 |  |  | 4707 | -0.008 | -0.1304 | No |
| 12 | PSEN2 |  |  | 5019 | -0.013 | -0.1576 | No |
| 13 | FBXW11 |  |  | 6201 | -0.033 | -0.2666 | No |
| 14 | JAG1 |  |  | 7426 | -0.061 | -0.3707 | No |
| 15 | FZD1 |  |  | 7685 | -0.069 | -0.3741 | No |
| 16 | LFNG |  |  | 7784 | -0.073 | -0.3601 | No |
| 17 | PSENEN |  |  | 8463 | -0.103 | -0.3951 | No |
| 18 | ST3GAL6 |  |  | 8941 | -0.138 | -0.3981 | Yes |
| 19 | NOTCH2 |  |  | 9279 | -0.177 | -0.3741 | Yes |
| 20 | HES1 |  |  | 9280 | -0.177 | -0.3159 | Yes |
| 21 | NOTCH3 |  |  | 9501 | -0.226 | -0.2638 | Yes |
| 22 | PRKCA |  |  | 9569 | -0.248 | -0.1888 | Yes |
| 23 | MAML2 |  |  | 9642 | -0.277 | -0.1048 | Yes |
| 24 | NOTCH1 |  |  | 9780 | -0.391 | 0.0100 | Yes |
Table: GSEA details [plain text format]

  

Fig 2: HALLMARK\_NOTCH\_SIGNALING: Random ES distribution      
 Gene set null distribution of ES for **HALLMARK\_NOTCH\_SIGNALING**

  
