# Supplementary material for: Modeling glioblastoma heterogeneity as a dynamic network of cell states
Source: Mol Syst Biol. 2021 Sep 16;17(9):e10105. doi: 10.15252/msb.202010105 (PMC8444284; doi:10.15252/msb.202010105)
Supplement: Supplementary file 5 — Source Data for Figure 3 [file MSB-17-e10105-s001.zip › Figure3A_sourcedata/GSEA_3065/hallmarks_state1.GseaPreranked.1623416262439/HALLMARK_OXIDATIVE_PHOSPHORYLATION.html]

Details for gene set HALLMARK\_OXIDATIVE\_PHOSPHORYLATION[GSEA]

|  || Dataset | state1 |
| Phenotype | NoPhenotypeAvailable |
| Upregulated in class | na\_pos |
| GeneSet | HALLMARK\_OXIDATIVE\_PHOSPHORYLATION |
| Enrichment Score (ES) | 0.64548063 |
| Normalized Enrichment Score (NES) | 2.5201385 |
| Nominal p-value | 0.0 |
| FDR q-value | 0.0 |
| FWER p-Value | 0.0 |
Table: GSEA Results Summary

  

Fig 1: Enrichment plot: HALLMARK\_OXIDATIVE\_PHOSPHORYLATION      
 Profile of the Running ES Score & Positions of GeneSet Members on the Rank Ordered List

  

| PROBE | GENE SYMBOL | GENE\_TITLE | RANK IN GENE LIST | RANK METRIC SCORE | RUNNING ES | CORE ENRICHMENT || 1 | LDHA |  |  | 80 | 0.394 | 0.0090 | Yes |
| 2 | NDUFS6 |  |  | 105 | 0.363 | 0.0223 | Yes |
| 3 | COX5A |  |  | 116 | 0.348 | 0.0365 | Yes |
| 4 | UQCRH |  |  | 144 | 0.326 | 0.0480 | Yes |
| 5 | SLC25A4 |  |  | 157 | 0.318 | 0.0606 | Yes |
| 6 | MRPL15 |  |  | 165 | 0.313 | 0.0736 | Yes |
| 7 | MRPL34 |  |  | 176 | 0.307 | 0.0860 | Yes |
| 8 | UQCR10 |  |  | 177 | 0.307 | 0.0994 | Yes |
| 9 | NDUFB4 |  |  | 200 | 0.295 | 0.1100 | Yes |
| 10 | CYCS |  |  | 203 | 0.294 | 0.1226 | Yes |
| 11 | GPX4 |  |  | 234 | 0.283 | 0.1319 | Yes |
| 12 | SLC25A5 |  |  | 254 | 0.276 | 0.1420 | Yes |
| 13 | UQCRFS1 |  |  | 265 | 0.273 | 0.1529 | Yes |
| 14 | SLC25A3 |  |  | 275 | 0.269 | 0.1637 | Yes |
| 15 | ECHS1 |  |  | 283 | 0.266 | 0.1746 | Yes |
| 16 | COX8A |  |  | 301 | 0.260 | 0.1842 | Yes |
| 17 | COX7A2 |  |  | 322 | 0.252 | 0.1932 | Yes |
| 18 | ATP5F1B |  |  | 330 | 0.250 | 0.2034 | Yes |
| 19 | MDH2 |  |  | 333 | 0.248 | 0.2141 | Yes |
| 20 | NDUFS8 |  |  | 345 | 0.242 | 0.2235 | Yes |
| 21 | NDUFA6 |  |  | 347 | 0.242 | 0.2340 | Yes |
| 22 | TOMM22 |  |  | 364 | 0.237 | 0.2426 | Yes |
| 23 | ATP5F1E |  |  | 386 | 0.229 | 0.2505 | Yes |
| 24 | COX5B |  |  | 400 | 0.226 | 0.2590 | Yes |
| 25 | TIMM13 |  |  | 403 | 0.225 | 0.2687 | Yes |
| 26 | NDUFV2 |  |  | 419 | 0.223 | 0.2768 | Yes |
| 27 | UQCRQ |  |  | 438 | 0.220 | 0.2846 | Yes |
| 28 | ATP5MG |  |  | 445 | 0.218 | 0.2935 | Yes |
| 29 | ATP5MF |  |  | 447 | 0.216 | 0.3028 | Yes |
| 30 | MRPS15 |  |  | 486 | 0.209 | 0.3080 | Yes |
| 31 | CYC1 |  |  | 511 | 0.203 | 0.3144 | Yes |
| 32 | ATP5F1C |  |  | 523 | 0.200 | 0.3221 | Yes |
| 33 | COX7B |  |  | 526 | 0.200 | 0.3306 | Yes |
| 34 | ATP5PF |  |  | 533 | 0.198 | 0.3386 | Yes |
| 35 | PDP1 |  |  | 536 | 0.198 | 0.3471 | Yes |
| 36 | ISCU |  |  | 542 | 0.198 | 0.3552 | Yes |
| 37 | NDUFB2 |  |  | 544 | 0.197 | 0.3637 | Yes |
| 38 | ATP6V1F |  |  | 560 | 0.193 | 0.3706 | Yes |
| 39 | COX17 |  |  | 565 | 0.191 | 0.3785 | Yes |
| 40 | MPC1 |  |  | 577 | 0.189 | 0.3857 | Yes |
| 41 | MRPS12 |  |  | 585 | 0.188 | 0.3932 | Yes |
| 42 | VDAC3 |  |  | 590 | 0.187 | 0.4009 | Yes |
| 43 | SDHB |  |  | 606 | 0.185 | 0.4075 | Yes |
| 44 | COX6C |  |  | 617 | 0.183 | 0.4145 | Yes |
| 45 | NDUFA8 |  |  | 656 | 0.178 | 0.4183 | Yes |
| 46 | COX6B1 |  |  | 663 | 0.176 | 0.4254 | Yes |
| 47 | COX4I1 |  |  | 675 | 0.176 | 0.4319 | Yes |
| 48 | SLC25A11 |  |  | 685 | 0.175 | 0.4386 | Yes |
| 49 | COX7C |  |  | 697 | 0.172 | 0.4450 | Yes |
| 50 | NDUFC1 |  |  | 706 | 0.171 | 0.4517 | Yes |
| 51 | ATP5PD |  |  | 728 | 0.168 | 0.4569 | Yes |
| 52 | VDAC1 |  |  | 732 | 0.167 | 0.4639 | Yes |
| 53 | NDUFB3 |  |  | 766 | 0.162 | 0.4675 | Yes |
| 54 | ATP5PO |  |  | 772 | 0.162 | 0.4741 | Yes |
| 55 | UQCRC1 |  |  | 773 | 0.161 | 0.4811 | Yes |
| 56 | ATP6V1E1 |  |  | 778 | 0.160 | 0.4877 | Yes |
| 57 | ATP5MC3 |  |  | 796 | 0.157 | 0.4928 | Yes |
| 58 | ATP5F1D |  |  | 818 | 0.155 | 0.4974 | Yes |
| 59 | ATP5MC1 |  |  | 820 | 0.154 | 0.5041 | Yes |
| 60 | NDUFA2 |  |  | 830 | 0.153 | 0.5099 | Yes |
| 61 | ALAS1 |  |  | 831 | 0.153 | 0.5165 | Yes |
| 62 | ATP5MC2 |  |  | 832 | 0.153 | 0.5232 | Yes |
| 63 | TIMM17A |  |  | 839 | 0.153 | 0.5293 | Yes |
| 64 | NDUFB7 |  |  | 854 | 0.151 | 0.5344 | Yes |
| 65 | MTX2 |  |  | 886 | 0.146 | 0.5376 | Yes |
| 66 | NDUFAB1 |  |  | 887 | 0.146 | 0.5440 | Yes |
| 67 | GRPEL1 |  |  | 904 | 0.144 | 0.5487 | Yes |
| 68 | UQCR11 |  |  | 918 | 0.143 | 0.5536 | Yes |
| 69 | SLC25A6 |  |  | 939 | 0.141 | 0.5577 | Yes |
| 70 | NQO2 |  |  | 971 | 0.137 | 0.5604 | Yes |
| 71 | PHB2 |  |  | 985 | 0.135 | 0.5650 | Yes |
| 72 | COX7A2L |  |  | 990 | 0.135 | 0.5705 | Yes |
| 73 | PRDX3 |  |  | 1003 | 0.133 | 0.5751 | Yes |
| 74 | BAX |  |  | 1029 | 0.130 | 0.5782 | Yes |
| 75 | NDUFB8 |  |  | 1058 | 0.127 | 0.5808 | Yes |
| 76 | VDAC2 |  |  | 1080 | 0.124 | 0.5841 | Yes |
| 77 | TIMM50 |  |  | 1127 | 0.119 | 0.5846 | Yes |
| 78 | NDUFB6 |  |  | 1138 | 0.119 | 0.5887 | Yes |
| 79 | MRPL11 |  |  | 1150 | 0.118 | 0.5927 | Yes |
| 80 | ATP5PB |  |  | 1152 | 0.118 | 0.5978 | Yes |
| 81 | ISCA1 |  |  | 1158 | 0.117 | 0.6024 | Yes |
| 82 | NDUFA1 |  |  | 1172 | 0.115 | 0.6061 | Yes |
| 83 | IDH3A |  |  | 1199 | 0.112 | 0.6083 | Yes |
| 84 | CASP7 |  |  | 1205 | 0.112 | 0.6127 | Yes |
| 85 | HCCS |  |  | 1210 | 0.111 | 0.6171 | Yes |
| 86 | FDX1 |  |  | 1274 | 0.106 | 0.6153 | Yes |
| 87 | PDHB |  |  | 1277 | 0.106 | 0.6197 | Yes |
| 88 | MRPS30 |  |  | 1287 | 0.105 | 0.6233 | Yes |
| 89 | PDHA1 |  |  | 1370 | 0.098 | 0.6192 | Yes |
| 90 | ATP6V0E1 |  |  | 1421 | 0.094 | 0.6181 | Yes |
| 91 | ACAA2 |  |  | 1425 | 0.094 | 0.6219 | Yes |
| 92 | ATP6V1D |  |  | 1441 | 0.093 | 0.6244 | Yes |
| 93 | FH |  |  | 1455 | 0.092 | 0.6271 | Yes |
| 94 | TIMM8B |  |  | 1473 | 0.091 | 0.6293 | Yes |
| 95 | TIMM10 |  |  | 1484 | 0.090 | 0.6322 | Yes |
| 96 | ATP5ME |  |  | 1524 | 0.087 | 0.6320 | Yes |
| 97 | ETFA |  |  | 1530 | 0.087 | 0.6353 | Yes |
| 98 | CYB5R3 |  |  | 1534 | 0.087 | 0.6388 | Yes |
| 99 | NDUFB1 |  |  | 1554 | 0.086 | 0.6406 | Yes |
| 100 | SURF1 |  |  | 1565 | 0.085 | 0.6433 | Yes |
| 101 | TIMM9 |  |  | 1590 | 0.083 | 0.6444 | Yes |
| 102 | GPI |  |  | 1615 | 0.081 | 0.6455 | Yes |
| 103 | ECI1 |  |  | 1698 | 0.076 | 0.6403 | No |
| 104 | IDH3B |  |  | 1707 | 0.075 | 0.6428 | No |
| 105 | HSPA9 |  |  | 1793 | 0.070 | 0.6370 | No |
| 106 | SUCLG1 |  |  | 1795 | 0.070 | 0.6400 | No |
| 107 | ATP5F1A |  |  | 1903 | 0.065 | 0.6318 | No |
| 108 | NDUFB5 |  |  | 1905 | 0.065 | 0.6345 | No |
| 109 | HSD17B10 |  |  | 1922 | 0.064 | 0.6357 | No |
| 110 | ATP6V1G1 |  |  | 1946 | 0.063 | 0.6360 | No |
| 111 | SDHD |  |  | 2039 | 0.058 | 0.6290 | No |
| 112 | MDH1 |  |  | 2144 | 0.053 | 0.6206 | No |
| 113 | NDUFV1 |  |  | 2161 | 0.052 | 0.6213 | No |
| 114 | NDUFS3 |  |  | 2186 | 0.052 | 0.6210 | No |
| 115 | COX6A1 |  |  | 2260 | 0.049 | 0.6156 | No |
| 116 | DLAT |  |  | 2282 | 0.048 | 0.6156 | No |
| 117 | NDUFA5 |  |  | 2339 | 0.046 | 0.6118 | No |
| 118 | ECH1 |  |  | 2355 | 0.045 | 0.6122 | No |
| 119 | SUCLA2 |  |  | 2377 | 0.044 | 0.6119 | No |
| 120 | FXN |  |  | 2409 | 0.043 | 0.6106 | No |
| 121 | NDUFS7 |  |  | 2430 | 0.042 | 0.6104 | No |
| 122 | MRPL35 |  |  | 2480 | 0.040 | 0.6071 | No |
| 123 | GOT2 |  |  | 2496 | 0.040 | 0.6073 | No |
| 124 | MRPS11 |  |  | 2533 | 0.038 | 0.6052 | No |
| 125 | SDHA |  |  | 2540 | 0.038 | 0.6063 | No |
| 126 | NDUFA9 |  |  | 2573 | 0.037 | 0.6046 | No |
| 127 | NDUFS4 |  |  | 2596 | 0.036 | 0.6039 | No |
| 128 | UQCRB |  |  | 2623 | 0.035 | 0.6028 | No |
| 129 | ETFB |  |  | 2629 | 0.035 | 0.6038 | No |
| 130 | TOMM70 |  |  | 2664 | 0.034 | 0.6018 | No |
| 131 | PDHX |  |  | 2669 | 0.034 | 0.6029 | No |
| 132 | NDUFA7 |  |  | 2748 | 0.032 | 0.5962 | No |
| 133 | OAT |  |  | 2778 | 0.031 | 0.5946 | No |
| 134 | ACAT1 |  |  | 2842 | 0.029 | 0.5893 | No |
| 135 | MFN2 |  |  | 2871 | 0.028 | 0.5877 | No |
| 136 | DLD |  |  | 2976 | 0.025 | 0.5781 | No |
| 137 | CS |  |  | 3094 | 0.023 | 0.5670 | No |
| 138 | HTRA2 |  |  | 3132 | 0.022 | 0.5641 | No |
| 139 | IDH3G |  |  | 3238 | 0.020 | 0.5541 | No |
| 140 | GLUD1 |  |  | 3290 | 0.018 | 0.5497 | No |
| 141 | COX11 |  |  | 3434 | 0.015 | 0.5356 | No |
| 142 | PMPCA |  |  | 3475 | 0.014 | 0.5321 | No |
| 143 | CYB5A |  |  | 3529 | 0.013 | 0.5272 | No |
| 144 | OGDH |  |  | 3589 | 0.012 | 0.5216 | No |
| 145 | ACAA1 |  |  | 3782 | 0.008 | 0.5021 | No |
| 146 | NDUFA4 |  |  | 3842 | 0.007 | 0.4964 | No |
| 147 | SUPV3L1 |  |  | 3969 | 0.005 | 0.4836 | No |
| 148 | MTRR |  |  | 4129 | 0.002 | 0.4672 | No |
| 149 | MGST3 |  |  | 4220 | 0.000 | 0.4580 | No |
| 150 | DLST |  |  | 4280 | -0.001 | 0.4519 | No |
| 151 | SLC25A20 |  |  | 4395 | -0.003 | 0.4403 | No |
| 152 | ACADVL |  |  | 4425 | -0.003 | 0.4374 | No |
| 153 | ACADSB |  |  | 4455 | -0.004 | 0.4346 | No |
| 154 | UQCRC2 |  |  | 4472 | -0.004 | 0.4331 | No |
| 155 | OXA1L |  |  | 4507 | -0.005 | 0.4298 | No |
| 156 | POLR2F |  |  | 4700 | -0.007 | 0.4103 | No |
| 157 | LDHB |  |  | 4712 | -0.008 | 0.4095 | No |
| 158 | COX10 |  |  | 4779 | -0.009 | 0.4030 | No |
| 159 | IMMT |  |  | 4844 | -0.010 | 0.3969 | No |
| 160 | MRPS22 |  |  | 4937 | -0.012 | 0.3879 | No |
| 161 | DECR1 |  |  | 5098 | -0.014 | 0.3720 | No |
| 162 | PHYH |  |  | 5120 | -0.014 | 0.3704 | No |
| 163 | COX15 |  |  | 5155 | -0.015 | 0.3676 | No |
| 164 | SDHC |  |  | 5201 | -0.015 | 0.3636 | No |
| 165 | AIFM1 |  |  | 5241 | -0.016 | 0.3603 | No |
| 166 | SLC25A12 |  |  | 5343 | -0.017 | 0.3506 | No |
| 167 | AFG3L2 |  |  | 5355 | -0.018 | 0.3502 | No |
| 168 | ACO2 |  |  | 5411 | -0.019 | 0.3454 | No |
| 169 | OPA1 |  |  | 5522 | -0.020 | 0.3349 | No |
| 170 | ACADM |  |  | 5968 | -0.029 | 0.2902 | No |
| 171 | RHOT1 |  |  | 6491 | -0.038 | 0.2380 | No |
| 172 | ATP6V1H |  |  | 6562 | -0.040 | 0.2325 | No |
| 173 | NDUFS2 |  |  | 6696 | -0.043 | 0.2207 | No |
| 174 | ATP6V1C1 |  |  | 6768 | -0.044 | 0.2153 | No |
| 175 | ATP6V0B |  |  | 6884 | -0.047 | 0.2054 | No |
| 176 | RETSAT |  |  | 6959 | -0.049 | 0.1999 | No |
| 177 | HADHA |  |  | 7089 | -0.053 | 0.1889 | No |
| 178 | NDUFS1 |  |  | 7229 | -0.056 | 0.1770 | No |
| 179 | ETFDH |  |  | 7288 | -0.058 | 0.1735 | No |
| 180 | ALDH6A1 |  |  | 7321 | -0.059 | 0.1728 | No |
| 181 | IDH2 |  |  | 7549 | -0.065 | 0.1522 | No |
| 182 | NDUFA3 |  |  | 7635 | -0.067 | 0.1464 | No |
| 183 | ABCB7 |  |  | 7891 | -0.077 | 0.1234 | No |
| 184 | RHOT2 |  |  | 8215 | -0.090 | 0.0940 | No |
| 185 | NDUFC2 |  |  | 8383 | -0.098 | 0.0810 | No |
| 186 | LRPPRC |  |  | 8646 | -0.114 | 0.0590 | No |
| 187 | HADHB |  |  | 8718 | -0.119 | 0.0568 | No |
| 188 | NNT |  |  | 8770 | -0.123 | 0.0569 | No |
| 189 | MAOB |  |  | 8834 | -0.127 | 0.0560 | No |
| 190 | BDH2 |  |  | 8927 | -0.136 | 0.0525 | No |
| 191 | POR |  |  | 9415 | -0.204 | 0.0111 | No |
| 192 | ATP1B1 |  |  | 9542 | -0.236 | 0.0084 | No |
| 193 | ATP6AP1 |  |  | 9582 | -0.254 | 0.0155 | No |
| 194 | IDH1 |  |  | 9737 | -0.345 | 0.0147 | No |
Table: GSEA details [plain text format]

  

Fig 2: HALLMARK\_OXIDATIVE\_PHOSPHORYLATION: Random ES distribution      
 Gene set null distribution of ES for **HALLMARK\_OXIDATIVE\_PHOSPHORYLATION**

  
