# Supplementary material for: Modeling glioblastoma heterogeneity as a dynamic network of cell states
Source: Mol Syst Biol. 2021 Sep 16;17(9):e10105. doi: 10.15252/msb.202010105 (PMC8444284; doi:10.15252/msb.202010105)
Supplement: Supplementary file 5 — Source Data for Figure 3 [file MSB-17-e10105-s001.zip › Figure3A_sourcedata/GSEA_3065/hallmarks_state1.GseaPreranked.1623416262439/HALLMARK_P53_PATHWAY.html]

Details for gene set HALLMARK\_P53\_PATHWAY[GSEA]

|  || Dataset | state1 |
| Phenotype | NoPhenotypeAvailable |
| Upregulated in class | na\_pos |
| GeneSet | HALLMARK\_P53\_PATHWAY |
| Enrichment Score (ES) | 0.3257559 |
| Normalized Enrichment Score (NES) | 1.2376424 |
| Nominal p-value | 0.09090909 |
| FDR q-value | 0.20718041 |
| FWER p-Value | 0.927 |
Table: GSEA Results Summary

  

Fig 1: Enrichment plot: HALLMARK\_P53\_PATHWAY      
 Profile of the Running ES Score & Positions of GeneSet Members on the Rank Ordered List

  

| PROBE | GENE SYMBOL | GENE\_TITLE | RANK IN GENE LIST | RANK METRIC SCORE | RUNNING ES | CORE ENRICHMENT || 1 | S100A10 |  |  | 33 | 0.540 | 0.0302 | Yes |
| 2 | UPP1 |  |  | 37 | 0.523 | 0.0624 | Yes |
| 3 | RPS12 |  |  | 73 | 0.411 | 0.0843 | Yes |
| 4 | HINT1 |  |  | 78 | 0.402 | 0.1089 | Yes |
| 5 | HRAS |  |  | 159 | 0.317 | 0.1204 | Yes |
| 6 | RPL36 |  |  | 206 | 0.292 | 0.1339 | Yes |
| 7 | PTPN14 |  |  | 224 | 0.287 | 0.1499 | Yes |
| 8 | CDKN1A |  |  | 262 | 0.274 | 0.1632 | Yes |
| 9 | IRAK1 |  |  | 332 | 0.249 | 0.1716 | Yes |
| 10 | TM4SF1 |  |  | 379 | 0.231 | 0.1813 | Yes |
| 11 | RPL18 |  |  | 383 | 0.230 | 0.1952 | Yes |
| 12 | JUN |  |  | 387 | 0.229 | 0.2092 | Yes |
| 13 | STEAP3 |  |  | 441 | 0.218 | 0.2173 | Yes |
| 14 | SLC7A11 |  |  | 459 | 0.214 | 0.2288 | Yes |
| 15 | DCXR |  |  | 474 | 0.211 | 0.2405 | Yes |
| 16 | RGS16 |  |  | 499 | 0.206 | 0.2509 | Yes |
| 17 | RACK1 |  |  | 510 | 0.204 | 0.2625 | Yes |
| 18 | ISCU |  |  | 542 | 0.198 | 0.2716 | Yes |
| 19 | PLK2 |  |  | 725 | 0.168 | 0.2634 | Yes |
| 20 | BMP2 |  |  | 760 | 0.164 | 0.2701 | Yes |
| 21 | NDRG1 |  |  | 765 | 0.163 | 0.2798 | Yes |
| 22 | HMOX1 |  |  | 784 | 0.160 | 0.2879 | Yes |
| 23 | PERP |  |  | 876 | 0.148 | 0.2877 | Yes |
| 24 | TSC22D1 |  |  | 881 | 0.147 | 0.2964 | Yes |
| 25 | CD82 |  |  | 1024 | 0.131 | 0.2900 | Yes |
| 26 | BAX |  |  | 1029 | 0.130 | 0.2977 | Yes |
| 27 | TAX1BP3 |  |  | 1081 | 0.124 | 0.3002 | Yes |
| 28 | GADD45A |  |  | 1082 | 0.124 | 0.3079 | Yes |
| 29 | PMM1 |  |  | 1105 | 0.122 | 0.3132 | Yes |
| 30 | ZNF365 |  |  | 1117 | 0.121 | 0.3196 | Yes |
| 31 | TPRKB |  |  | 1211 | 0.111 | 0.3170 | Yes |
| 32 | NINJ1 |  |  | 1234 | 0.109 | 0.3215 | Yes |
| 33 | TRIAP1 |  |  | 1374 | 0.098 | 0.3133 | Yes |
| 34 | CCND3 |  |  | 1389 | 0.096 | 0.3178 | Yes |
| 35 | EI24 |  |  | 1427 | 0.094 | 0.3199 | Yes |
| 36 | EPHA2 |  |  | 1452 | 0.092 | 0.3231 | Yes |
| 37 | SDC1 |  |  | 1482 | 0.090 | 0.3258 | Yes |
| 38 | SLC3A2 |  |  | 1646 | 0.079 | 0.3139 | No |
| 39 | CDKN2AIP |  |  | 1712 | 0.074 | 0.3119 | No |
| 40 | CCNG1 |  |  | 1716 | 0.074 | 0.3162 | No |
| 41 | PHLDA3 |  |  | 1738 | 0.073 | 0.3186 | No |
| 42 | FAM162A |  |  | 1805 | 0.069 | 0.3161 | No |
| 43 | RAD51C |  |  | 1874 | 0.066 | 0.3132 | No |
| 44 | WWP1 |  |  | 2006 | 0.059 | 0.3035 | No |
| 45 | DDIT3 |  |  | 2008 | 0.059 | 0.3070 | No |
| 46 | TGFB1 |  |  | 2040 | 0.058 | 0.3074 | No |
| 47 | TGFA |  |  | 2063 | 0.057 | 0.3087 | No |
| 48 | S100A4 |  |  | 2114 | 0.054 | 0.3069 | No |
| 49 | PROCR |  |  | 2129 | 0.054 | 0.3088 | No |
| 50 | BTG1 |  |  | 2251 | 0.049 | 0.2995 | No |
| 51 | BAK1 |  |  | 2374 | 0.044 | 0.2897 | No |
| 52 | FBXW7 |  |  | 2486 | 0.040 | 0.2808 | No |
| 53 | MKNK2 |  |  | 2595 | 0.036 | 0.2719 | No |
| 54 | FDXR |  |  | 2642 | 0.035 | 0.2694 | No |
| 55 | SERTAD3 |  |  | 2670 | 0.034 | 0.2687 | No |
| 56 | RPS27L |  |  | 2723 | 0.032 | 0.2654 | No |
| 57 | ATF3 |  |  | 2855 | 0.029 | 0.2537 | No |
| 58 | NUDT15 |  |  | 2985 | 0.025 | 0.2420 | No |
| 59 | MAPKAPK3 |  |  | 3036 | 0.024 | 0.2384 | No |
| 60 | TCN2 |  |  | 3143 | 0.022 | 0.2288 | No |
| 61 | APAF1 |  |  | 3294 | 0.018 | 0.2146 | No |
| 62 | PPP1R15A |  |  | 3296 | 0.018 | 0.2156 | No |
| 63 | RCHY1 |  |  | 3409 | 0.016 | 0.2051 | No |
| 64 | TOB1 |  |  | 3439 | 0.015 | 0.2030 | No |
| 65 | PCNA |  |  | 3540 | 0.013 | 0.1936 | No |
| 66 | RRP8 |  |  | 3787 | 0.008 | 0.1688 | No |
| 67 | FAS |  |  | 3812 | 0.008 | 0.1668 | No |
| 68 | CDK5R1 |  |  | 3866 | 0.007 | 0.1618 | No |
| 69 | ADA |  |  | 3919 | 0.006 | 0.1568 | No |
| 70 | PTPRE |  |  | 4002 | 0.004 | 0.1487 | No |
| 71 | RNF19B |  |  | 4290 | -0.001 | 0.1193 | No |
| 72 | ANKRA2 |  |  | 4294 | -0.001 | 0.1190 | No |
| 73 | STOM |  |  | 4388 | -0.003 | 0.1096 | No |
| 74 | VWA5A |  |  | 4421 | -0.003 | 0.1065 | No |
| 75 | CCNK |  |  | 4465 | -0.004 | 0.1023 | No |
| 76 | HDAC3 |  |  | 4468 | -0.004 | 0.1024 | No |
| 77 | PRKAB1 |  |  | 4489 | -0.004 | 0.1006 | No |
| 78 | RAB40C |  |  | 4586 | -0.006 | 0.0911 | No |
| 79 | DRAM1 |  |  | 4678 | -0.007 | 0.0822 | No |
| 80 | LDHB |  |  | 4712 | -0.008 | 0.0793 | No |
| 81 | TRAFD1 |  |  | 4719 | -0.008 | 0.0791 | No |
| 82 | CSRNP2 |  |  | 4841 | -0.010 | 0.0673 | No |
| 83 | DNTTIP2 |  |  | 4877 | -0.011 | 0.0644 | No |
| 84 | PLK3 |  |  | 5051 | -0.013 | 0.0475 | No |
| 85 | TSPYL2 |  |  | 5056 | -0.013 | 0.0479 | No |
| 86 | EPHX1 |  |  | 5080 | -0.014 | 0.0464 | No |
| 87 | SLC35D1 |  |  | 5178 | -0.015 | 0.0373 | No |
| 88 | PVT1 |  |  | 5203 | -0.015 | 0.0358 | No |
| 89 | TAP1 |  |  | 5226 | -0.016 | 0.0346 | No |
| 90 | AEN |  |  | 5353 | -0.018 | 0.0227 | No |
| 91 | TP53 |  |  | 5557 | -0.021 | 0.0032 | No |
| 92 | SESN1 |  |  | 5566 | -0.021 | 0.0037 | No |
| 93 | IER5 |  |  | 5645 | -0.023 | -0.0029 | No |
| 94 | DGKA |  |  | 5816 | -0.026 | -0.0188 | No |
| 95 | RALGDS |  |  | 5838 | -0.026 | -0.0194 | No |
| 96 | KIF13B |  |  | 5839 | -0.026 | -0.0177 | No |
| 97 | PITPNC1 |  |  | 5863 | -0.026 | -0.0184 | No |
| 98 | HSPA4L |  |  | 5938 | -0.028 | -0.0243 | No |
| 99 | PIDD1 |  |  | 5961 | -0.029 | -0.0248 | No |
| 100 | WRAP73 |  |  | 6139 | -0.032 | -0.0410 | No |
| 101 | NOL8 |  |  | 6275 | -0.034 | -0.0527 | No |
| 102 | ELP1 |  |  | 6496 | -0.038 | -0.0729 | No |
| 103 | RAP2B |  |  | 6561 | -0.040 | -0.0770 | No |
| 104 | FUCA1 |  |  | 6606 | -0.041 | -0.0790 | No |
| 105 | PPM1D |  |  | 6609 | -0.041 | -0.0767 | No |
| 106 | DDIT4 |  |  | 6713 | -0.043 | -0.0846 | No |
| 107 | HEXIM1 |  |  | 6835 | -0.046 | -0.0942 | No |
| 108 | XPC |  |  | 6915 | -0.048 | -0.0993 | No |
| 109 | RETSAT |  |  | 6959 | -0.049 | -0.1007 | No |
| 110 | SP1 |  |  | 7019 | -0.051 | -0.1036 | No |
| 111 | IP6K2 |  |  | 7026 | -0.051 | -0.1011 | No |
| 112 | POLH |  |  | 7029 | -0.051 | -0.0981 | No |
| 113 | RXRA |  |  | 7177 | -0.055 | -0.1098 | No |
| 114 | CGRRF1 |  |  | 7273 | -0.057 | -0.1160 | No |
| 115 | CCP110 |  |  | 7307 | -0.058 | -0.1158 | No |
| 116 | MDM2 |  |  | 7386 | -0.060 | -0.1200 | No |
| 117 | BAIAP2 |  |  | 7435 | -0.061 | -0.1211 | No |
| 118 | TRAF4 |  |  | 7503 | -0.064 | -0.1241 | No |
| 119 | CYFIP2 |  |  | 7946 | -0.079 | -0.1646 | No |
| 120 | BLCAP |  |  | 7983 | -0.080 | -0.1632 | No |
| 121 | MXD4 |  |  | 8080 | -0.084 | -0.1679 | No |
| 122 | DDB2 |  |  | 8121 | -0.086 | -0.1666 | No |
| 123 | POM121 |  |  | 8282 | -0.094 | -0.1772 | No |
| 124 | ABCC5 |  |  | 8787 | -0.124 | -0.2213 | No |
| 125 | ZFP36L1 |  |  | 8789 | -0.124 | -0.2137 | No |
| 126 | ITGB4 |  |  | 8832 | -0.127 | -0.2101 | No |
| 127 | PLXNB2 |  |  | 8857 | -0.129 | -0.2045 | No |
| 128 | RAD9A |  |  | 8905 | -0.134 | -0.2010 | No |
| 129 | PRMT2 |  |  | 8908 | -0.134 | -0.1929 | No |
| 130 | F2R |  |  | 8950 | -0.138 | -0.1885 | No |
| 131 | CTSD |  |  | 9287 | -0.178 | -0.2119 | No |
| 132 | ZMAT3 |  |  | 9300 | -0.181 | -0.2019 | No |
| 133 | BTG2 |  |  | 9304 | -0.181 | -0.1910 | No |
| 134 | PDGFA |  |  | 9341 | -0.188 | -0.1830 | No |
| 135 | SEC61A1 |  |  | 9343 | -0.188 | -0.1714 | No |
| 136 | TM7SF3 |  |  | 9409 | -0.202 | -0.1655 | No |
| 137 | CD81 |  |  | 9434 | -0.208 | -0.1551 | No |
| 138 | FOXO3 |  |  | 9503 | -0.226 | -0.1480 | No |
| 139 | CTSF |  |  | 9563 | -0.246 | -0.1388 | No |
| 140 | FOS |  |  | 9566 | -0.247 | -0.1236 | No |
| 141 | TXNIP |  |  | 9639 | -0.276 | -0.1139 | No |
| 142 | ABAT |  |  | 9646 | -0.278 | -0.0972 | No |
| 143 | NOTCH1 |  |  | 9780 | -0.391 | -0.0866 | No |
| 144 | SAT1 |  |  | 9807 | -0.445 | -0.0616 | No |
| 145 | APP |  |  | 9819 | -0.495 | -0.0319 | No |
| 146 | CCND2 |  |  | 9842 | -0.611 | 0.0038 | No |
Table: GSEA details [plain text format]

  

Fig 2: HALLMARK\_P53\_PATHWAY: Random ES distribution      
 Gene set null distribution of ES for **HALLMARK\_P53\_PATHWAY**

  
