# Supplementary material for: Modeling glioblastoma heterogeneity as a dynamic network of cell states
Source: Mol Syst Biol. 2021 Sep 16;17(9):e10105. doi: 10.15252/msb.202010105 (PMC8444284; doi:10.15252/msb.202010105)
Supplement: Supplementary file 5 — Source Data for Figure 3 [file MSB-17-e10105-s001.zip › Figure3A_sourcedata/GSEA_3065/hallmarks_state1.GseaPreranked.1623416262439/HALLMARK_PI3K_AKT_MTOR_SIGNALING.html]

Details for gene set HALLMARK\_PI3K\_AKT\_MTOR\_SIGNALING[GSEA]

|  || Dataset | state1 |
| Phenotype | NoPhenotypeAvailable |
| Upregulated in class | na\_pos |
| GeneSet | HALLMARK\_PI3K\_AKT\_MTOR\_SIGNALING |
| Enrichment Score (ES) | 0.482575 |
| Normalized Enrichment Score (NES) | 1.6825476 |
| Nominal p-value | 0.0 |
| FDR q-value | 0.014403103 |
| FWER p-Value | 0.047 |
Table: GSEA Results Summary

  

Fig 1: Enrichment plot: HALLMARK\_PI3K\_AKT\_MTOR\_SIGNALING      
 Profile of the Running ES Score & Positions of GeneSet Members on the Rank Ordered List

  

| PROBE | GENE SYMBOL | GENE\_TITLE | RANK IN GENE LIST | RANK METRIC SCORE | RUNNING ES | CORE ENRICHMENT || 1 | PFN1 |  |  | 26 | 0.591 | 0.0611 | Yes |
| 2 | CFL1 |  |  | 138 | 0.329 | 0.0852 | Yes |
| 3 | ARF1 |  |  | 156 | 0.319 | 0.1179 | Yes |
| 4 | HRAS |  |  | 159 | 0.317 | 0.1519 | Yes |
| 5 | ARHGDIA |  |  | 185 | 0.303 | 0.1820 | Yes |
| 6 | ARPC3 |  |  | 214 | 0.290 | 0.2104 | Yes |
| 7 | SLC2A1 |  |  | 235 | 0.283 | 0.2389 | Yes |
| 8 | CDKN1A |  |  | 262 | 0.274 | 0.2658 | Yes |
| 9 | PIN1 |  |  | 264 | 0.274 | 0.2953 | Yes |
| 10 | AP2M1 |  |  | 335 | 0.247 | 0.3148 | Yes |
| 11 | PPP1CA |  |  | 377 | 0.232 | 0.3357 | Yes |
| 12 | RAC1 |  |  | 472 | 0.211 | 0.3489 | Yes |
| 13 | CXCR4 |  |  | 532 | 0.199 | 0.3643 | Yes |
| 14 | AKT1 |  |  | 567 | 0.191 | 0.3814 | Yes |
| 15 | SQSTM1 |  |  | 701 | 0.172 | 0.3863 | Yes |
| 16 | CSNK2B |  |  | 733 | 0.167 | 0.4012 | Yes |
| 17 | ACTR3 |  |  | 744 | 0.166 | 0.4181 | Yes |
| 18 | YWHAB |  |  | 754 | 0.165 | 0.4350 | Yes |
| 19 | UBE2D3 |  |  | 782 | 0.160 | 0.4495 | Yes |
| 20 | PRKAR2A |  |  | 1011 | 0.133 | 0.4405 | Yes |
| 21 | MAPKAP1 |  |  | 1036 | 0.130 | 0.4520 | Yes |
| 22 | GRB2 |  |  | 1195 | 0.113 | 0.4481 | Yes |
| 23 | UBE2N |  |  | 1316 | 0.102 | 0.4468 | Yes |
| 24 | VAV3 |  |  | 1338 | 0.100 | 0.4555 | Yes |
| 25 | TIAM1 |  |  | 1395 | 0.095 | 0.4601 | Yes |
| 26 | PTPN11 |  |  | 1399 | 0.095 | 0.4700 | Yes |
| 27 | RALB |  |  | 1408 | 0.095 | 0.4794 | Yes |
| 28 | EGFR |  |  | 1474 | 0.091 | 0.4826 | Yes |
| 29 | MAP2K3 |  |  | 1643 | 0.079 | 0.4740 | No |
| 30 | AKT1S1 |  |  | 1855 | 0.067 | 0.4597 | No |
| 31 | RPS6KA1 |  |  | 1878 | 0.066 | 0.4646 | No |
| 32 | CDK4 |  |  | 2000 | 0.060 | 0.4586 | No |
| 33 | DDIT3 |  |  | 2008 | 0.059 | 0.4643 | No |
| 34 | MAPK9 |  |  | 2211 | 0.051 | 0.4492 | No |
| 35 | PPP2R1B |  |  | 2301 | 0.047 | 0.4452 | No |
| 36 | RPS6KA3 |  |  | 2353 | 0.045 | 0.4448 | No |
| 37 | CAB39 |  |  | 2440 | 0.042 | 0.4405 | No |
| 38 | EIF4E |  |  | 2517 | 0.039 | 0.4370 | No |
| 39 | MKNK1 |  |  | 2536 | 0.038 | 0.4393 | No |
| 40 | MKNK2 |  |  | 2595 | 0.036 | 0.4373 | No |
| 41 | MYD88 |  |  | 2810 | 0.030 | 0.4187 | No |
| 42 | PAK4 |  |  | 2859 | 0.029 | 0.4170 | No |
| 43 | ATF1 |  |  | 2933 | 0.027 | 0.4124 | No |
| 44 | ACTR2 |  |  | 2953 | 0.026 | 0.4132 | No |
| 45 | GRK2 |  |  | 3249 | 0.019 | 0.3852 | No |
| 46 | CDK1 |  |  | 3396 | 0.016 | 0.3721 | No |
| 47 | NFKBIB |  |  | 3480 | 0.014 | 0.3651 | No |
| 48 | ECSIT |  |  | 3502 | 0.014 | 0.3645 | No |
| 49 | TRAF2 |  |  | 3546 | 0.013 | 0.3615 | No |
| 50 | RIPK1 |  |  | 3739 | 0.009 | 0.3428 | No |
| 51 | RIT1 |  |  | 4461 | -0.004 | 0.2697 | No |
| 52 | CDKN1B |  |  | 4655 | -0.007 | 0.2507 | No |
| 53 | MAPK8 |  |  | 4962 | -0.012 | 0.2208 | No |
| 54 | DUSP3 |  |  | 5223 | -0.016 | 0.1959 | No |
| 55 | MAP2K6 |  |  | 5421 | -0.019 | 0.1778 | No |
| 56 | THEM4 |  |  | 5648 | -0.023 | 0.1572 | No |
| 57 | CDK2 |  |  | 5964 | -0.029 | 0.1282 | No |
| 58 | E2F1 |  |  | 6247 | -0.034 | 0.1030 | No |
| 59 | IRAK4 |  |  | 6320 | -0.035 | 0.0994 | No |
| 60 | PRKAG1 |  |  | 6346 | -0.036 | 0.1007 | No |
| 61 | PLCG1 |  |  | 6388 | -0.036 | 0.1004 | No |
| 62 | MAP3K7 |  |  | 6399 | -0.036 | 0.1034 | No |
| 63 | TBK1 |  |  | 6598 | -0.041 | 0.0875 | No |
| 64 | NCK1 |  |  | 6783 | -0.045 | 0.0736 | No |
| 65 | PIKFYVE |  |  | 6894 | -0.047 | 0.0674 | No |
| 66 | PRKAA2 |  |  | 7227 | -0.056 | 0.0396 | No |
| 67 | GSK3B |  |  | 7351 | -0.059 | 0.0334 | No |
| 68 | PLA2G12A |  |  | 7555 | -0.065 | 0.0197 | No |
| 69 | ACACA |  |  | 7654 | -0.068 | 0.0170 | No |
| 70 | MAPK1 |  |  | 7779 | -0.072 | 0.0122 | No |
| 71 | PTEN |  |  | 7881 | -0.077 | 0.0102 | No |
| 72 | SMAD2 |  |  | 7926 | -0.078 | 0.0141 | No |
| 73 | CLTC |  |  | 8290 | -0.094 | -0.0128 | No |
| 74 | ADCY2 |  |  | 8311 | -0.095 | -0.0046 | No |
| 75 | PIK3R3 |  |  | 8411 | -0.100 | -0.0039 | No |
| 76 | TNFRSF1A |  |  | 8416 | -0.100 | 0.0064 | No |
| 77 | RAF1 |  |  | 8419 | -0.100 | 0.0170 | No |
| 78 | STAT2 |  |  | 9081 | -0.150 | -0.0343 | No |
| 79 | TSC2 |  |  | 9271 | -0.175 | -0.0347 | No |
| 80 | HSP90B1 |  |  | 9365 | -0.193 | -0.0233 | No |
| 81 | MAPK10 |  |  | 9478 | -0.221 | -0.0110 | No |
| 82 | CALR |  |  | 9537 | -0.235 | 0.0084 | No |
| 83 | ITPR2 |  |  | 9560 | -0.244 | 0.0326 | No |
Table: GSEA details [plain text format]

  

Fig 2: HALLMARK\_PI3K\_AKT\_MTOR\_SIGNALING: Random ES distribution      
 Gene set null distribution of ES for **HALLMARK\_PI3K\_AKT\_MTOR\_SIGNALING**

  
