# Supplementary material for: Modeling glioblastoma heterogeneity as a dynamic network of cell states
Source: Mol Syst Biol. 2021 Sep 16;17(9):e10105. doi: 10.15252/msb.202010105 (PMC8444284; doi:10.15252/msb.202010105)
Supplement: Supplementary file 5 — Source Data for Figure 3 [file MSB-17-e10105-s001.zip › Figure3A_sourcedata/GSEA_3065/hallmarks_state1.GseaPreranked.1623416262439/HALLMARK_PROTEIN_SECRETION.html]

Details for gene set HALLMARK\_PROTEIN\_SECRETION[GSEA]

|  || Dataset | state1 |
| Phenotype | NoPhenotypeAvailable |
| Upregulated in class | na\_neg |
| GeneSet | HALLMARK\_PROTEIN\_SECRETION |
| Enrichment Score (ES) | -0.26097193 |
| Normalized Enrichment Score (NES) | -0.9066968 |
| Nominal p-value | 0.6427289 |
| FDR q-value | 0.7568429 |
| FWER p-Value | 1.0 |
Table: GSEA Results Summary

  

Fig 1: Enrichment plot: HALLMARK\_PROTEIN\_SECRETION      
 Profile of the Running ES Score & Positions of GeneSet Members on the Rank Ordered List

  

| PROBE | GENE SYMBOL | GENE\_TITLE | RANK IN GENE LIST | RANK METRIC SCORE | RUNNING ES | CORE ENRICHMENT || 1 | CTSC |  |  | 43 | 0.498 | 0.0530 | No |
| 2 | ARF1 |  |  | 156 | 0.319 | 0.0783 | No |
| 3 | SOD1 |  |  | 186 | 0.303 | 0.1103 | No |
| 4 | AP2S1 |  |  | 251 | 0.276 | 0.1356 | No |
| 5 | AP2M1 |  |  | 335 | 0.247 | 0.1556 | No |
| 6 | BNIP3 |  |  | 570 | 0.190 | 0.1536 | No |
| 7 | CLTA |  |  | 642 | 0.180 | 0.1671 | No |
| 8 | YKT6 |  |  | 698 | 0.172 | 0.1813 | No |
| 9 | GNAS |  |  | 1010 | 0.133 | 0.1648 | No |
| 10 | TSG101 |  |  | 1026 | 0.131 | 0.1783 | No |
| 11 | AP3S1 |  |  | 1075 | 0.125 | 0.1878 | No |
| 12 | CAV2 |  |  | 1146 | 0.118 | 0.1943 | No |
| 13 | COPE |  |  | 1169 | 0.116 | 0.2054 | No |
| 14 | VAMP3 |  |  | 1382 | 0.097 | 0.1949 | No |
| 15 | EGFR |  |  | 1474 | 0.091 | 0.1960 | No |
| 16 | ARCN1 |  |  | 1567 | 0.085 | 0.1964 | No |
| 17 | NAPA |  |  | 1683 | 0.076 | 0.1934 | No |
| 18 | ANP32E |  |  | 1706 | 0.075 | 0.1998 | No |
| 19 | NAPG |  |  | 1915 | 0.064 | 0.1860 | No |
| 20 | GOLGA4 |  |  | 1955 | 0.062 | 0.1892 | No |
| 21 | ARFGAP3 |  |  | 1991 | 0.060 | 0.1925 | No |
| 22 | VAMP7 |  |  | 2010 | 0.059 | 0.1975 | No |
| 23 | TPD52 |  |  | 2098 | 0.055 | 0.1950 | No |
| 24 | BET1 |  |  | 2160 | 0.053 | 0.1948 | No |
| 25 | RPS6KA3 |  |  | 2353 | 0.045 | 0.1804 | No |
| 26 | GOSR2 |  |  | 2463 | 0.041 | 0.1739 | No |
| 27 | VPS4B |  |  | 2558 | 0.038 | 0.1687 | No |
| 28 | OCRL |  |  | 2822 | 0.030 | 0.1453 | No |
| 29 | SEC31A |  |  | 3160 | 0.021 | 0.1133 | No |
| 30 | RAB22A |  |  | 3252 | 0.019 | 0.1062 | No |
| 31 | DST |  |  | 3373 | 0.017 | 0.0959 | No |
| 32 | SNX2 |  |  | 3451 | 0.015 | 0.0897 | No |
| 33 | ZW10 |  |  | 3571 | 0.012 | 0.0790 | No |
| 34 | SEC24D |  |  | 4162 | 0.001 | 0.0189 | No |
| 35 | ARFIP1 |  |  | 4206 | 0.001 | 0.0146 | No |
| 36 | SNAP23 |  |  | 4402 | -0.003 | -0.0050 | No |
| 37 | TMED2 |  |  | 4551 | -0.006 | -0.0195 | No |
| 38 | RAB14 |  |  | 4625 | -0.006 | -0.0262 | No |
| 39 | USO1 |  |  | 4771 | -0.008 | -0.0400 | No |
| 40 | GBF1 |  |  | 4986 | -0.012 | -0.0604 | No |
| 41 | SEC22B |  |  | 5034 | -0.013 | -0.0637 | No |
| 42 | VAMP4 |  |  | 5059 | -0.013 | -0.0646 | No |
| 43 | SCAMP3 |  |  | 5340 | -0.017 | -0.0912 | No |
| 44 | STX7 |  |  | 5373 | -0.018 | -0.0924 | No |
| 45 | COPB2 |  |  | 5695 | -0.023 | -0.1225 | No |
| 46 | ARFGEF2 |  |  | 5706 | -0.024 | -0.1208 | No |
| 47 | STAM |  |  | 5798 | -0.025 | -0.1272 | No |
| 48 | TOM1L1 |  |  | 5811 | -0.026 | -0.1255 | No |
| 49 | RAB9A |  |  | 5858 | -0.026 | -0.1271 | No |
| 50 | RER1 |  |  | 5866 | -0.027 | -0.1248 | No |
| 51 | AP1G1 |  |  | 6204 | -0.033 | -0.1554 | No |
| 52 | DNM1L |  |  | 6273 | -0.034 | -0.1584 | No |
| 53 | ATP6V1H |  |  | 6562 | -0.040 | -0.1832 | No |
| 54 | COPB1 |  |  | 6710 | -0.043 | -0.1933 | No |
| 55 | SSPN |  |  | 6940 | -0.048 | -0.2111 | No |
| 56 | ATP7A |  |  | 7206 | -0.056 | -0.2317 | No |
| 57 | STX16 |  |  | 7367 | -0.060 | -0.2412 | No |
| 58 | ARFGEF1 |  |  | 7417 | -0.061 | -0.2392 | No |
| 59 | COG2 |  |  | 7488 | -0.063 | -0.2391 | No |
| 60 | AP3B1 |  |  | 7599 | -0.066 | -0.2427 | No |
| 61 | MAPK1 |  |  | 7779 | -0.072 | -0.2526 | Yes |
| 62 | RAB5A |  |  | 7781 | -0.073 | -0.2444 | Yes |
| 63 | STX12 |  |  | 7782 | -0.073 | -0.2360 | Yes |
| 64 | MON2 |  |  | 7818 | -0.074 | -0.2310 | Yes |
| 65 | SCRN1 |  |  | 7886 | -0.077 | -0.2290 | Yes |
| 66 | SCAMP1 |  |  | 7888 | -0.077 | -0.2203 | Yes |
| 67 | ATP1A1 |  |  | 7974 | -0.080 | -0.2197 | Yes |
| 68 | ADAM10 |  |  | 8046 | -0.083 | -0.2174 | Yes |
| 69 | ICA1 |  |  | 8277 | -0.093 | -0.2301 | Yes |
| 70 | CLTC |  |  | 8290 | -0.094 | -0.2205 | Yes |
| 71 | GLA |  |  | 8303 | -0.095 | -0.2108 | Yes |
| 72 | PAM |  |  | 8343 | -0.096 | -0.2037 | Yes |
| 73 | RAB2A |  |  | 8354 | -0.097 | -0.1936 | Yes |
| 74 | VPS45 |  |  | 8499 | -0.105 | -0.1962 | Yes |
| 75 | TMX1 |  |  | 8524 | -0.107 | -0.1863 | Yes |
| 76 | IGF2R |  |  | 8525 | -0.107 | -0.1740 | Yes |
| 77 | ABCA1 |  |  | 8590 | -0.111 | -0.1678 | Yes |
| 78 | M6PR |  |  | 8772 | -0.123 | -0.1721 | Yes |
| 79 | CLN5 |  |  | 8854 | -0.129 | -0.1655 | Yes |
| 80 | AP2B1 |  |  | 9080 | -0.150 | -0.1712 | Yes |
| 81 | LMAN1 |  |  | 9094 | -0.151 | -0.1550 | Yes |
| 82 | CD63 |  |  | 9126 | -0.154 | -0.1404 | Yes |
| 83 | ERGIC3 |  |  | 9200 | -0.165 | -0.1288 | Yes |
| 84 | PPT1 |  |  | 9318 | -0.184 | -0.1196 | Yes |
| 85 | YIPF6 |  |  | 9473 | -0.220 | -0.1101 | Yes |
| 86 | CLCN3 |  |  | 9586 | -0.255 | -0.0922 | Yes |
| 87 | KIF1B |  |  | 9649 | -0.282 | -0.0660 | Yes |
| 88 | TMED10 |  |  | 9750 | -0.356 | -0.0352 | Yes |
| 89 | LAMP2 |  |  | 9796 | -0.419 | 0.0085 | Yes |
Table: GSEA details [plain text format]

  

Fig 2: HALLMARK\_PROTEIN\_SECRETION: Random ES distribution      
 Gene set null distribution of ES for **HALLMARK\_PROTEIN\_SECRETION**

  
