# Supplementary material for: Modeling glioblastoma heterogeneity as a dynamic network of cell states
Source: Mol Syst Biol. 2021 Sep 16;17(9):e10105. doi: 10.15252/msb.202010105 (PMC8444284; doi:10.15252/msb.202010105)
Supplement: Supplementary file 5 — Source Data for Figure 3 [file MSB-17-e10105-s001.zip › Figure3A_sourcedata/GSEA_3065/hallmarks_state1.GseaPreranked.1623416262439/HALLMARK_REACTIVE_OXYGEN_SPECIES_PATHWAY.html]

Details for gene set HALLMARK\_REACTIVE\_OXYGEN\_SPECIES\_PATHWAY[GSEA]

|  || Dataset | state1 |
| Phenotype | NoPhenotypeAvailable |
| Upregulated in class | na\_pos |
| GeneSet | HALLMARK\_REACTIVE\_OXYGEN\_SPECIES\_PATHWAY |
| Enrichment Score (ES) | 0.7104706 |
| Normalized Enrichment Score (NES) | 2.1619427 |
| Nominal p-value | 0.0 |
| FDR q-value | 0.0 |
| FWER p-Value | 0.0 |
Table: GSEA Results Summary

  

Fig 1: Enrichment plot: HALLMARK\_REACTIVE\_OXYGEN\_SPECIES\_PATHWAY      
 Profile of the Running ES Score & Positions of GeneSet Members on the Rank Ordered List

  

| PROBE | GENE SYMBOL | GENE\_TITLE | RANK IN GENE LIST | RANK METRIC SCORE | RUNNING ES | CORE ENRICHMENT || 1 | NQO1 |  |  | 10 | 0.693 | 0.1042 | Yes |
| 2 | TXN |  |  | 13 | 0.686 | 0.2083 | Yes |
| 3 | PRDX6 |  |  | 79 | 0.398 | 0.2622 | Yes |
| 4 | TXNRD1 |  |  | 97 | 0.376 | 0.3177 | Yes |
| 5 | FTL |  |  | 134 | 0.333 | 0.3646 | Yes |
| 6 | G6PD |  |  | 171 | 0.310 | 0.4080 | Yes |
| 7 | ATOX1 |  |  | 179 | 0.306 | 0.4538 | Yes |
| 8 | SOD1 |  |  | 186 | 0.303 | 0.4993 | Yes |
| 9 | NDUFB4 |  |  | 200 | 0.295 | 0.5427 | Yes |
| 10 | GPX4 |  |  | 234 | 0.283 | 0.5824 | Yes |
| 11 | NDUFA6 |  |  | 347 | 0.242 | 0.6077 | Yes |
| 12 | GLRX2 |  |  | 457 | 0.214 | 0.6291 | Yes |
| 13 | PFKP |  |  | 594 | 0.187 | 0.6437 | Yes |
| 14 | LAMTOR5 |  |  | 601 | 0.186 | 0.6713 | Yes |
| 15 | PRDX2 |  |  | 794 | 0.157 | 0.6758 | Yes |
| 16 | GLRX |  |  | 927 | 0.142 | 0.6839 | Yes |
| 17 | CDKN2D |  |  | 1213 | 0.111 | 0.6719 | Yes |
| 18 | GCLM |  |  | 1238 | 0.109 | 0.6860 | Yes |
| 19 | PRDX1 |  |  | 1305 | 0.103 | 0.6949 | Yes |
| 20 | HMOX2 |  |  | 1307 | 0.103 | 0.7105 | Yes |
| 21 | MSRA |  |  | 1694 | 0.076 | 0.6828 | No |
| 22 | PTPA |  |  | 1902 | 0.065 | 0.6716 | No |
| 23 | TXNRD2 |  |  | 2151 | 0.053 | 0.6544 | No |
| 24 | OXSR1 |  |  | 2247 | 0.049 | 0.6523 | No |
| 25 | GSR |  |  | 3274 | 0.019 | 0.5509 | No |
| 26 | SOD2 |  |  | 3646 | 0.011 | 0.5148 | No |
| 27 | STK25 |  |  | 4727 | -0.008 | 0.4063 | No |
| 28 | CAT |  |  | 4951 | -0.012 | 0.3854 | No |
| 29 | PDLIM1 |  |  | 5622 | -0.022 | 0.3207 | No |
| 30 | GCLC |  |  | 5628 | -0.022 | 0.3236 | No |
| 31 | SBNO2 |  |  | 6000 | -0.029 | 0.2903 | No |
| 32 | PRNP |  |  | 6661 | -0.042 | 0.2297 | No |
| 33 | NDUFS2 |  |  | 6696 | -0.043 | 0.2327 | No |
| 34 | ERCC2 |  |  | 6845 | -0.046 | 0.2247 | No |
| 35 | SCAF4 |  |  | 8197 | -0.089 | 0.1009 | No |
| 36 | JUNB |  |  | 8634 | -0.113 | 0.0738 | No |
| 37 | SELENOS |  |  | 8867 | -0.130 | 0.0701 | No |
| 38 | PRDX4 |  |  | 9457 | -0.215 | 0.0429 | No |
Table: GSEA details [plain text format]

  

Fig 2: HALLMARK\_REACTIVE\_OXYGEN\_SPECIES\_PATHWAY: Random ES distribution      
 Gene set null distribution of ES for **HALLMARK\_REACTIVE\_OXYGEN\_SPECIES\_PATHWAY**

  
