# Supplementary material for: Modeling glioblastoma heterogeneity as a dynamic network of cell states
Source: Mol Syst Biol. 2021 Sep 16;17(9):e10105. doi: 10.15252/msb.202010105 (PMC8444284; doi:10.15252/msb.202010105)
Supplement: Supplementary file 5 — Source Data for Figure 3 [file MSB-17-e10105-s001.zip › Figure3A_sourcedata/GSEA_3065/hallmarks_state1.GseaPreranked.1623416262439/HALLMARK_TNFA_SIGNALING_VIA_NFKB.html]

Details for gene set HALLMARK\_TNFA\_SIGNALING\_VIA\_NFKB[GSEA]

|  || Dataset | state1 |
| Phenotype | NoPhenotypeAvailable |
| Upregulated in class | na\_neg |
| GeneSet | HALLMARK\_TNFA\_SIGNALING\_VIA\_NFKB |
| Enrichment Score (ES) | -0.27907223 |
| Normalized Enrichment Score (NES) | -1.0112321 |
| Nominal p-value | 0.411985 |
| FDR q-value | 0.64202434 |
| FWER p-Value | 1.0 |
Table: GSEA Results Summary

  

Fig 1: Enrichment plot: HALLMARK\_TNFA\_SIGNALING\_VIA\_NFKB      
 Profile of the Running ES Score & Positions of GeneSet Members on the Rank Ordered List

  

| PROBE | GENE SYMBOL | GENE\_TITLE | RANK IN GENE LIST | RANK METRIC SCORE | RUNNING ES | CORE ENRICHMENT || 1 | PMEPA1 |  |  | 12 | 0.687 | 0.0501 | No |
| 2 | CCND1 |  |  | 29 | 0.566 | 0.0908 | No |
| 3 | PHLDA1 |  |  | 83 | 0.389 | 0.1144 | No |
| 4 | DUSP4 |  |  | 218 | 0.288 | 0.1222 | No |
| 5 | CDKN1A |  |  | 262 | 0.274 | 0.1383 | No |
| 6 | JUN |  |  | 387 | 0.229 | 0.1427 | No |
| 7 | EIF1 |  |  | 503 | 0.205 | 0.1463 | No |
| 8 | SQSTM1 |  |  | 701 | 0.172 | 0.1390 | No |
| 9 | PLK2 |  |  | 725 | 0.168 | 0.1491 | No |
| 10 | BMP2 |  |  | 760 | 0.164 | 0.1579 | No |
| 11 | B4GALT1 |  |  | 802 | 0.157 | 0.1654 | No |
| 12 | TSC22D1 |  |  | 881 | 0.147 | 0.1684 | No |
| 13 | G0S2 |  |  | 903 | 0.145 | 0.1771 | No |
| 14 | F2RL1 |  |  | 908 | 0.144 | 0.1874 | No |
| 15 | FOSL1 |  |  | 953 | 0.139 | 0.1933 | No |
| 16 | GADD45A |  |  | 1082 | 0.124 | 0.1895 | No |
| 17 | DUSP5 |  |  | 1114 | 0.121 | 0.1953 | No |
| 18 | ETS2 |  |  | 1131 | 0.119 | 0.2026 | No |
| 19 | TIPARP |  |  | 1142 | 0.118 | 0.2104 | No |
| 20 | FOSL2 |  |  | 1143 | 0.118 | 0.2193 | No |
| 21 | NINJ1 |  |  | 1234 | 0.109 | 0.2182 | No |
| 22 | YRDC |  |  | 1237 | 0.109 | 0.2262 | No |
| 23 | SERPINE1 |  |  | 1250 | 0.108 | 0.2331 | No |
| 24 | TNIP1 |  |  | 1270 | 0.107 | 0.2391 | No |
| 25 | PLAUR |  |  | 1463 | 0.091 | 0.2263 | No |
| 26 | PDLIM5 |  |  | 1528 | 0.087 | 0.2262 | No |
| 27 | IFNGR2 |  |  | 1555 | 0.086 | 0.2300 | No |
| 28 | IRF1 |  |  | 1569 | 0.085 | 0.2350 | No |
| 29 | NAMPT |  |  | 1622 | 0.081 | 0.2357 | No |
| 30 | MAP2K3 |  |  | 1643 | 0.079 | 0.2396 | No |
| 31 | CEBPB |  |  | 1680 | 0.076 | 0.2416 | No |
| 32 | PLAU |  |  | 1801 | 0.069 | 0.2345 | No |
| 33 | TANK |  |  | 1815 | 0.069 | 0.2383 | No |
| 34 | SERPINB8 |  |  | 1841 | 0.067 | 0.2408 | No |
| 35 | EHD1 |  |  | 1846 | 0.067 | 0.2454 | No |
| 36 | IER2 |  |  | 1899 | 0.065 | 0.2450 | No |
| 37 | RHOB |  |  | 1914 | 0.064 | 0.2483 | No |
| 38 | LAMB3 |  |  | 1988 | 0.060 | 0.2453 | No |
| 39 | TNIP2 |  |  | 2035 | 0.058 | 0.2449 | No |
| 40 | SDC4 |  |  | 2111 | 0.054 | 0.2413 | No |
| 41 | GFPT2 |  |  | 2127 | 0.054 | 0.2438 | No |
| 42 | NFKBIA |  |  | 2153 | 0.053 | 0.2452 | No |
| 43 | CD44 |  |  | 2193 | 0.051 | 0.2450 | No |
| 44 | BTG1 |  |  | 2251 | 0.049 | 0.2429 | No |
| 45 | GADD45B |  |  | 2252 | 0.049 | 0.2465 | No |
| 46 | RELB |  |  | 2338 | 0.046 | 0.2412 | No |
| 47 | MAFF |  |  | 2492 | 0.040 | 0.2285 | No |
| 48 | BTG3 |  |  | 2514 | 0.039 | 0.2293 | No |
| 49 | GEM |  |  | 2691 | 0.033 | 0.2137 | No |
| 50 | DUSP1 |  |  | 2706 | 0.033 | 0.2148 | No |
| 51 | ATF3 |  |  | 2855 | 0.029 | 0.2018 | No |
| 52 | CFLAR |  |  | 2958 | 0.026 | 0.1932 | No |
| 53 | PFKFB3 |  |  | 3039 | 0.024 | 0.1868 | No |
| 54 | PPP1R15A |  |  | 3296 | 0.018 | 0.1620 | No |
| 55 | NFKB1 |  |  | 3337 | 0.017 | 0.1592 | No |
| 56 | NFKBIE |  |  | 3376 | 0.016 | 0.1565 | No |
| 57 | BCL3 |  |  | 3622 | 0.011 | 0.1322 | No |
| 58 | SOD2 |  |  | 3646 | 0.011 | 0.1307 | No |
| 59 | TNFAIP8 |  |  | 3901 | 0.006 | 0.1051 | No |
| 60 | MYC |  |  | 3964 | 0.005 | 0.0992 | No |
| 61 | PTPRE |  |  | 4002 | 0.004 | 0.0957 | No |
| 62 | LITAF |  |  | 4039 | 0.003 | 0.0923 | No |
| 63 | NFIL3 |  |  | 4056 | 0.003 | 0.0909 | No |
| 64 | KLF6 |  |  | 4117 | 0.002 | 0.0849 | No |
| 65 | RIPK2 |  |  | 4145 | 0.002 | 0.0823 | No |
| 66 | RNF19B |  |  | 4290 | -0.001 | 0.0676 | No |
| 67 | RELA |  |  | 4420 | -0.003 | 0.0546 | No |
| 68 | KLF10 |  |  | 4486 | -0.004 | 0.0483 | No |
| 69 | DRAM1 |  |  | 4678 | -0.007 | 0.0292 | No |
| 70 | LDLR |  |  | 4731 | -0.008 | 0.0245 | No |
| 71 | TRIP10 |  |  | 4935 | -0.012 | 0.0045 | No |
| 72 | BIRC2 |  |  | 5042 | -0.013 | -0.0053 | No |
| 73 | DNAJB4 |  |  | 5195 | -0.015 | -0.0197 | No |
| 74 | TAP1 |  |  | 5226 | -0.016 | -0.0216 | No |
| 75 | ATP2B1 |  |  | 5540 | -0.021 | -0.0522 | No |
| 76 | IER5 |  |  | 5645 | -0.023 | -0.0611 | No |
| 77 | DDX58 |  |  | 5907 | -0.028 | -0.0858 | No |
| 78 | SLC2A6 |  |  | 5925 | -0.028 | -0.0854 | No |
| 79 | PANX1 |  |  | 6031 | -0.030 | -0.0940 | No |
| 80 | BHLHE40 |  |  | 6102 | -0.031 | -0.0988 | No |
| 81 | SMAD3 |  |  | 6222 | -0.033 | -0.1085 | No |
| 82 | CSF1 |  |  | 6351 | -0.036 | -0.1190 | No |
| 83 | TGIF1 |  |  | 6360 | -0.036 | -0.1171 | No |
| 84 | REL |  |  | 6930 | -0.048 | -0.1718 | No |
| 85 | KDM6B |  |  | 7083 | -0.052 | -0.1835 | No |
| 86 | JAG1 |  |  | 7426 | -0.061 | -0.2140 | No |
| 87 | NFE2L2 |  |  | 7575 | -0.066 | -0.2242 | No |
| 88 | PNRC1 |  |  | 8006 | -0.081 | -0.2622 | No |
| 89 | MCL1 |  |  | 8011 | -0.082 | -0.2565 | No |
| 90 | FJX1 |  |  | 8037 | -0.083 | -0.2529 | No |
| 91 | B4GALT5 |  |  | 8110 | -0.086 | -0.2539 | No |
| 92 | ZBTB10 |  |  | 8173 | -0.089 | -0.2536 | No |
| 93 | ID2 |  |  | 8360 | -0.097 | -0.2654 | No |
| 94 | NFAT5 |  |  | 8372 | -0.097 | -0.2592 | No |
| 95 | SOCS3 |  |  | 8373 | -0.098 | -0.2519 | No |
| 96 | DENND5A |  |  | 8396 | -0.099 | -0.2468 | No |
| 97 | ABCA1 |  |  | 8590 | -0.111 | -0.2583 | No |
| 98 | JUNB |  |  | 8634 | -0.113 | -0.2542 | No |
| 99 | BCL6 |  |  | 8706 | -0.118 | -0.2527 | No |
| 100 | EGR1 |  |  | 8797 | -0.124 | -0.2526 | No |
| 101 | SNN |  |  | 8815 | -0.126 | -0.2450 | No |
| 102 | RCAN1 |  |  | 9149 | -0.158 | -0.2673 | Yes |
| 103 | SLC2A3 |  |  | 9156 | -0.159 | -0.2560 | Yes |
| 104 | VEGFA |  |  | 9220 | -0.168 | -0.2499 | Yes |
| 105 | HES1 |  |  | 9280 | -0.177 | -0.2427 | Yes |
| 106 | BTG2 |  |  | 9304 | -0.181 | -0.2315 | Yes |
| 107 | CCNL1 |  |  | 9347 | -0.189 | -0.2217 | Yes |
| 108 | IL6ST |  |  | 9519 | -0.231 | -0.2220 | Yes |
| 109 | PDE4B |  |  | 9522 | -0.231 | -0.2049 | Yes |
| 110 | PLPP3 |  |  | 9549 | -0.239 | -0.1897 | Yes |
| 111 | FOS |  |  | 9566 | -0.247 | -0.1729 | Yes |
| 112 | TNC |  |  | 9605 | -0.262 | -0.1572 | Yes |
| 113 | TNFAIP6 |  |  | 9629 | -0.272 | -0.1393 | Yes |
| 114 | IRS2 |  |  | 9641 | -0.277 | -0.1197 | Yes |
| 115 | TUBB2A |  |  | 9669 | -0.293 | -0.1005 | Yes |
| 116 | CEBPD |  |  | 9675 | -0.295 | -0.0790 | Yes |
| 117 | MARCKS |  |  | 9763 | -0.370 | -0.0602 | Yes |
| 118 | SAT1 |  |  | 9807 | -0.445 | -0.0313 | Yes |
| 119 | PTX3 |  |  | 9827 | -0.517 | 0.0053 | Yes |
Table: GSEA details [plain text format]

  

Fig 2: HALLMARK\_TNFA\_SIGNALING\_VIA\_NFKB: Random ES distribution      
 Gene set null distribution of ES for **HALLMARK\_TNFA\_SIGNALING\_VIA\_NFKB**

  
