# Supplementary material for: Modeling glioblastoma heterogeneity as a dynamic network of cell states
Source: Mol Syst Biol. 2021 Sep 16;17(9):e10105. doi: 10.15252/msb.202010105 (PMC8444284; doi:10.15252/msb.202010105)
Supplement: Supplementary file 5 — Source Data for Figure 3 [file MSB-17-e10105-s001.zip › Figure3A_sourcedata/GSEA_3065/hallmarks_state1.GseaPreranked.1623416262439/HALLMARK_UV_RESPONSE_DN.html]

Details for gene set HALLMARK\_UV\_RESPONSE\_DN[GSEA]

|  || Dataset | state1 |
| Phenotype | NoPhenotypeAvailable |
| Upregulated in class | na\_neg |
| GeneSet | HALLMARK\_UV\_RESPONSE\_DN |
| Enrichment Score (ES) | -0.2660252 |
| Normalized Enrichment Score (NES) | -0.957074 |
| Nominal p-value | 0.5608856 |
| FDR q-value | 0.6731794 |
| FWER p-Value | 1.0 |
Table: GSEA Results Summary

  

Fig 1: Enrichment plot: HALLMARK\_UV\_RESPONSE\_DN      
 Profile of the Running ES Score & Positions of GeneSet Members on the Rank Ordered List

  

| PROBE | GENE SYMBOL | GENE\_TITLE | RANK IN GENE LIST | RANK METRIC SCORE | RUNNING ES | CORE ENRICHMENT || 1 | CAV1 |  |  | 34 | 0.536 | 0.0396 | No |
| 2 | ANXA2 |  |  | 48 | 0.482 | 0.0771 | No |
| 3 | SYNJ2 |  |  | 56 | 0.461 | 0.1135 | No |
| 4 | IGFBP5 |  |  | 88 | 0.385 | 0.1413 | No |
| 5 | CACNA1A |  |  | 107 | 0.360 | 0.1684 | No |
| 6 | FHL2 |  |  | 141 | 0.328 | 0.1914 | No |
| 7 | KCNMA1 |  |  | 253 | 0.276 | 0.2023 | No |
| 8 | MET |  |  | 451 | 0.215 | 0.1994 | No |
| 9 | COL1A2 |  |  | 517 | 0.201 | 0.2090 | No |
| 10 | MGLL |  |  | 633 | 0.181 | 0.2118 | No |
| 11 | TGFBR2 |  |  | 650 | 0.179 | 0.2245 | No |
| 12 | CITED2 |  |  | 683 | 0.175 | 0.2353 | No |
| 13 | BDNF |  |  | 963 | 0.138 | 0.2179 | No |
| 14 | SDC2 |  |  | 1049 | 0.128 | 0.2195 | No |
| 15 | DLG1 |  |  | 1217 | 0.111 | 0.2113 | No |
| 16 | SERPINE1 |  |  | 1250 | 0.108 | 0.2167 | No |
| 17 | ANXA4 |  |  | 1348 | 0.099 | 0.2148 | No |
| 18 | RND3 |  |  | 1386 | 0.096 | 0.2188 | No |
| 19 | RBPMS |  |  | 1404 | 0.095 | 0.2247 | No |
| 20 | PDLIM5 |  |  | 1528 | 0.087 | 0.2191 | No |
| 21 | AMPH |  |  | 1537 | 0.087 | 0.2253 | No |
| 22 | NRP1 |  |  | 1691 | 0.076 | 0.2157 | No |
| 23 | GCNT1 |  |  | 1696 | 0.076 | 0.2214 | No |
| 24 | ADORA2B |  |  | 1891 | 0.066 | 0.2068 | No |
| 25 | IRS1 |  |  | 2033 | 0.058 | 0.1970 | No |
| 26 | SRI |  |  | 2084 | 0.056 | 0.1964 | No |
| 27 | NEK7 |  |  | 2548 | 0.038 | 0.1521 | No |
| 28 | GRK5 |  |  | 2613 | 0.036 | 0.1484 | No |
| 29 | INPP4B |  |  | 2636 | 0.035 | 0.1489 | No |
| 30 | DUSP1 |  |  | 2706 | 0.033 | 0.1445 | No |
| 31 | ZMIZ1 |  |  | 2887 | 0.028 | 0.1283 | No |
| 32 | NFKB1 |  |  | 3337 | 0.017 | 0.0837 | No |
| 33 | RUNX1 |  |  | 3487 | 0.014 | 0.0696 | No |
| 34 | PEX14 |  |  | 3537 | 0.013 | 0.0656 | No |
| 35 | INSIG1 |  |  | 3553 | 0.013 | 0.0651 | No |
| 36 | MAPK14 |  |  | 3651 | 0.011 | 0.0561 | No |
| 37 | BCKDHB |  |  | 3913 | 0.006 | 0.0298 | No |
| 38 | SFMBT1 |  |  | 3941 | 0.005 | 0.0275 | No |
| 39 | MYC |  |  | 3964 | 0.005 | 0.0256 | No |
| 40 | PRKAR2B |  |  | 4073 | 0.003 | 0.0148 | No |
| 41 | PIAS3 |  |  | 4118 | 0.002 | 0.0105 | No |
| 42 | EFEMP1 |  |  | 4333 | -0.002 | -0.0113 | No |
| 43 | MRPS31 |  |  | 4370 | -0.002 | -0.0148 | No |
| 44 | SPOP |  |  | 4653 | -0.007 | -0.0431 | No |
| 45 | CDKN1B |  |  | 4655 | -0.007 | -0.0426 | No |
| 46 | LDLR |  |  | 4731 | -0.008 | -0.0497 | No |
| 47 | NR3C1 |  |  | 4804 | -0.009 | -0.0563 | No |
| 48 | SLC7A1 |  |  | 4822 | -0.009 | -0.0573 | No |
| 49 | RASA2 |  |  | 4860 | -0.010 | -0.0603 | No |
| 50 | PDGFRB |  |  | 5041 | -0.013 | -0.0777 | No |
| 51 | MTA1 |  |  | 5162 | -0.015 | -0.0887 | No |
| 52 | ATP2B1 |  |  | 5540 | -0.021 | -0.1257 | No |
| 53 | YTHDC1 |  |  | 5681 | -0.023 | -0.1381 | No |
| 54 | KALRN |  |  | 5692 | -0.023 | -0.1373 | No |
| 55 | SCAF8 |  |  | 5867 | -0.027 | -0.1529 | No |
| 56 | AGGF1 |  |  | 5975 | -0.029 | -0.1616 | No |
| 57 | BHLHE40 |  |  | 6102 | -0.031 | -0.1720 | No |
| 58 | VAV2 |  |  | 6116 | -0.031 | -0.1708 | No |
| 59 | SMAD3 |  |  | 6222 | -0.033 | -0.1789 | No |
| 60 | MIOS |  |  | 6312 | -0.035 | -0.1852 | No |
| 61 | ATP2B4 |  |  | 6357 | -0.036 | -0.1868 | No |
| 62 | VLDLR |  |  | 6518 | -0.039 | -0.2001 | No |
| 63 | ATXN1 |  |  | 6560 | -0.040 | -0.2011 | No |
| 64 | SCHIP1 |  |  | 6575 | -0.040 | -0.1993 | No |
| 65 | SLC22A18 |  |  | 6599 | -0.041 | -0.1984 | No |
| 66 | ID1 |  |  | 6749 | -0.044 | -0.2101 | No |
| 67 | CELF2 |  |  | 6823 | -0.045 | -0.2139 | No |
| 68 | DDAH1 |  |  | 6943 | -0.049 | -0.2222 | No |
| 69 | TENT4A |  |  | 6958 | -0.049 | -0.2197 | No |
| 70 | LAMC1 |  |  | 6974 | -0.049 | -0.2173 | No |
| 71 | AKT3 |  |  | 7063 | -0.052 | -0.2221 | No |
| 72 | BMPR1A |  |  | 7093 | -0.053 | -0.2208 | No |
| 73 | RXRA |  |  | 7177 | -0.055 | -0.2249 | No |
| 74 | MAP2K5 |  |  | 7441 | -0.061 | -0.2469 | No |
| 75 | GJA1 |  |  | 7471 | -0.062 | -0.2448 | No |
| 76 | PHF3 |  |  | 7490 | -0.063 | -0.2416 | No |
| 77 | CAP2 |  |  | 7580 | -0.066 | -0.2454 | No |
| 78 | TOGARAM1 |  |  | 7630 | -0.067 | -0.2450 | No |
| 79 | ATP2C1 |  |  | 7738 | -0.071 | -0.2503 | No |
| 80 | ACVR2A |  |  | 7786 | -0.073 | -0.2492 | No |
| 81 | DMAC2L |  |  | 7844 | -0.075 | -0.2490 | No |
| 82 | CDC42BPA |  |  | 7878 | -0.077 | -0.2462 | No |
| 83 | PTEN |  |  | 7881 | -0.077 | -0.2403 | No |
| 84 | PRDM2 |  |  | 7928 | -0.078 | -0.2387 | No |
| 85 | NR1D2 |  |  | 8120 | -0.086 | -0.2513 | No |
| 86 | MAGI2 |  |  | 8265 | -0.093 | -0.2586 | Yes |
| 87 | ICA1 |  |  | 8277 | -0.093 | -0.2522 | Yes |
| 88 | DYRK1A |  |  | 8330 | -0.096 | -0.2498 | Yes |
| 89 | ADD3 |  |  | 8338 | -0.096 | -0.2428 | Yes |
| 90 | ERBB2 |  |  | 8409 | -0.100 | -0.2419 | Yes |
| 91 | PIK3R3 |  |  | 8411 | -0.100 | -0.2340 | Yes |
| 92 | IGF1R |  |  | 8469 | -0.103 | -0.2315 | Yes |
| 93 | FZD2 |  |  | 8518 | -0.106 | -0.2279 | Yes |
| 94 | NIPBL |  |  | 8569 | -0.110 | -0.2242 | Yes |
| 95 | COL11A1 |  |  | 8666 | -0.115 | -0.2247 | Yes |
| 96 | ATRN |  |  | 8898 | -0.134 | -0.2376 | Yes |
| 97 | MAP1B |  |  | 8951 | -0.139 | -0.2318 | Yes |
| 98 | SYNE1 |  |  | 9206 | -0.166 | -0.2444 | Yes |
| 99 | NOTCH2 |  |  | 9279 | -0.177 | -0.2375 | Yes |
| 100 | SIPA1L1 |  |  | 9296 | -0.180 | -0.2247 | Yes |
| 101 | CDK13 |  |  | 9320 | -0.184 | -0.2122 | Yes |
| 102 | ARHGEF9 |  |  | 9378 | -0.196 | -0.2023 | Yes |
| 103 | PMP22 |  |  | 9392 | -0.199 | -0.1876 | Yes |
| 104 | TFPI |  |  | 9427 | -0.206 | -0.1745 | Yes |
| 105 | PLPP3 |  |  | 9549 | -0.239 | -0.1676 | Yes |
| 106 | COL5A2 |  |  | 9551 | -0.240 | -0.1484 | Yes |
| 107 | PRKCA |  |  | 9569 | -0.248 | -0.1301 | Yes |
| 108 | NFIB |  |  | 9572 | -0.249 | -0.1103 | Yes |
| 109 | FYN |  |  | 9575 | -0.250 | -0.0904 | Yes |
| 110 | TJP1 |  |  | 9592 | -0.257 | -0.0714 | Yes |
| 111 | APBB2 |  |  | 9645 | -0.277 | -0.0544 | Yes |
| 112 | ATRX |  |  | 9654 | -0.284 | -0.0323 | Yes |
| 113 | MMP16 |  |  | 9724 | -0.335 | -0.0124 | Yes |
| 114 | HAS2 |  |  | 9739 | -0.350 | 0.0143 | Yes |
Table: GSEA details [plain text format]

  

Fig 2: HALLMARK\_UV\_RESPONSE\_DN: Random ES distribution      
 Gene set null distribution of ES for **HALLMARK\_UV\_RESPONSE\_DN**

  
