# Supplementary material for: Modeling glioblastoma heterogeneity as a dynamic network of cell states
Source: Mol Syst Biol. 2021 Sep 16;17(9):e10105. doi: 10.15252/msb.202010105 (PMC8444284; doi:10.15252/msb.202010105)
Supplement: Supplementary file 5 — Source Data for Figure 3 [file MSB-17-e10105-s001.zip › Figure3A_sourcedata/GSEA_3065/hallmarks_state1.GseaPreranked.1623416262439/HALLMARK_UV_RESPONSE_UP.html]

Details for gene set HALLMARK\_UV\_RESPONSE\_UP[GSEA]

|  || Dataset | state1 |
| Phenotype | NoPhenotypeAvailable |
| Upregulated in class | na\_pos |
| GeneSet | HALLMARK\_UV\_RESPONSE\_UP |
| Enrichment Score (ES) | 0.3480408 |
| Normalized Enrichment Score (NES) | 1.2844086 |
| Nominal p-value | 0.052742615 |
| FDR q-value | 0.17033038 |
| FWER p-Value | 0.849 |
Table: GSEA Results Summary

  

Fig 1: Enrichment plot: HALLMARK\_UV\_RESPONSE\_UP      
 Profile of the Running ES Score & Positions of GeneSet Members on the Rank Ordered List

  

| PROBE | GENE SYMBOL | GENE\_TITLE | RANK IN GENE LIST | RANK METRIC SCORE | RUNNING ES | CORE ENRICHMENT || 1 | CCK |  |  | 0 | 0.961 | 0.0814 | Yes |
| 2 | CTSV |  |  | 45 | 0.491 | 0.1184 | Yes |
| 3 | SLC25A4 |  |  | 157 | 0.318 | 0.1340 | Yes |
| 4 | CLTB |  |  | 238 | 0.281 | 0.1496 | Yes |
| 5 | AP2S1 |  |  | 251 | 0.276 | 0.1718 | Yes |
| 6 | PDAP1 |  |  | 280 | 0.268 | 0.1916 | Yes |
| 7 | AMD1 |  |  | 482 | 0.210 | 0.1888 | Yes |
| 8 | BID |  |  | 527 | 0.200 | 0.2012 | Yes |
| 9 | FKBP4 |  |  | 547 | 0.196 | 0.2159 | Yes |
| 10 | ATP6V1F |  |  | 560 | 0.193 | 0.2309 | Yes |
| 11 | STIP1 |  |  | 623 | 0.182 | 0.2400 | Yes |
| 12 | PSMC3 |  |  | 692 | 0.173 | 0.2477 | Yes |
| 13 | YKT6 |  |  | 698 | 0.172 | 0.2618 | Yes |
| 14 | SQSTM1 |  |  | 701 | 0.172 | 0.2761 | Yes |
| 15 | BMP2 |  |  | 760 | 0.164 | 0.2841 | Yes |
| 16 | HMOX1 |  |  | 784 | 0.160 | 0.2952 | Yes |
| 17 | EIF5 |  |  | 793 | 0.158 | 0.3077 | Yes |
| 18 | ALAS1 |  |  | 831 | 0.153 | 0.3169 | Yes |
| 19 | GRPEL1 |  |  | 904 | 0.144 | 0.3217 | Yes |
| 20 | CASP3 |  |  | 923 | 0.142 | 0.3319 | Yes |
| 21 | CYB5B |  |  | 1162 | 0.117 | 0.3174 | Yes |
| 22 | RAB27A |  |  | 1182 | 0.114 | 0.3251 | Yes |
| 23 | POLR2H |  |  | 1200 | 0.112 | 0.3329 | Yes |
| 24 | SELENOW |  |  | 1298 | 0.103 | 0.3317 | Yes |
| 25 | PPP1R2 |  |  | 1304 | 0.103 | 0.3399 | Yes |
| 26 | DNAJA1 |  |  | 1324 | 0.101 | 0.3465 | Yes |
| 27 | CCND3 |  |  | 1389 | 0.096 | 0.3480 | Yes |
| 28 | DNAJB1 |  |  | 1477 | 0.090 | 0.3468 | No |
| 29 | IRF1 |  |  | 1569 | 0.085 | 0.3446 | No |
| 30 | POLE3 |  |  | 1657 | 0.078 | 0.3423 | No |
| 31 | MRPL23 |  |  | 1682 | 0.076 | 0.3463 | No |
| 32 | RHOB |  |  | 1914 | 0.064 | 0.3281 | No |
| 33 | CDC34 |  |  | 1919 | 0.064 | 0.3331 | No |
| 34 | ALDOA |  |  | 1936 | 0.063 | 0.3369 | No |
| 35 | SHOX2 |  |  | 2091 | 0.055 | 0.3258 | No |
| 36 | TUBA4A |  |  | 2124 | 0.054 | 0.3271 | No |
| 37 | NFKBIA |  |  | 2153 | 0.053 | 0.3287 | No |
| 38 | UROD |  |  | 2183 | 0.052 | 0.3301 | No |
| 39 | BTG1 |  |  | 2251 | 0.049 | 0.3274 | No |
| 40 | LYN |  |  | 2272 | 0.048 | 0.3294 | No |
| 41 | BAK1 |  |  | 2374 | 0.044 | 0.3228 | No |
| 42 | DDX21 |  |  | 2406 | 0.043 | 0.3232 | No |
| 43 | FEN1 |  |  | 2420 | 0.043 | 0.3255 | No |
| 44 | PPIF |  |  | 2457 | 0.041 | 0.3253 | No |
| 45 | BTG3 |  |  | 2514 | 0.039 | 0.3229 | No |
| 46 | ATF3 |  |  | 2855 | 0.029 | 0.2905 | No |
| 47 | PRKCD |  |  | 2926 | 0.027 | 0.2856 | No |
| 48 | SPR |  |  | 2966 | 0.026 | 0.2838 | No |
| 49 | CYB5R1 |  |  | 2970 | 0.026 | 0.2856 | No |
| 50 | CCNE1 |  |  | 2995 | 0.025 | 0.2853 | No |
| 51 | PPAT |  |  | 3095 | 0.023 | 0.2771 | No |
| 52 | ENO2 |  |  | 3161 | 0.021 | 0.2722 | No |
| 53 | MSX1 |  |  | 3279 | 0.019 | 0.2618 | No |
| 54 | NUP58 |  |  | 3478 | 0.014 | 0.2427 | No |
| 55 | SOD2 |  |  | 3646 | 0.011 | 0.2265 | No |
| 56 | KLHDC3 |  |  | 3676 | 0.010 | 0.2244 | No |
| 57 | CHKA |  |  | 3687 | 0.010 | 0.2242 | No |
| 58 | FURIN |  |  | 3725 | 0.009 | 0.2213 | No |
| 59 | CEBPG |  |  | 3769 | 0.009 | 0.2176 | No |
| 60 | ACAA1 |  |  | 3782 | 0.008 | 0.2171 | No |
| 61 | NXF1 |  |  | 3809 | 0.008 | 0.2151 | No |
| 62 | PRKACA |  |  | 3910 | 0.006 | 0.2053 | No |
| 63 | TST |  |  | 4000 | 0.004 | 0.1966 | No |
| 64 | ARRB2 |  |  | 4160 | 0.001 | 0.1804 | No |
| 65 | CDO1 |  |  | 4212 | 0.001 | 0.1752 | No |
| 66 | MGAT1 |  |  | 4228 | 0.000 | 0.1737 | No |
| 67 | RXRB |  |  | 4574 | -0.006 | 0.1389 | No |
| 68 | STK25 |  |  | 4727 | -0.008 | 0.1239 | No |
| 69 | CDKN1C |  |  | 4834 | -0.010 | 0.1139 | No |
| 70 | MARK2 |  |  | 4905 | -0.011 | 0.1077 | No |
| 71 | TGFBRAP1 |  |  | 4940 | -0.012 | 0.1052 | No |
| 72 | EPHX1 |  |  | 5080 | -0.014 | 0.0921 | No |
| 73 | SIGMAR1 |  |  | 5103 | -0.014 | 0.0910 | No |
| 74 | TAP1 |  |  | 5226 | -0.016 | 0.0798 | No |
| 75 | ASNS |  |  | 5370 | -0.018 | 0.0667 | No |
| 76 | WIZ |  |  | 5389 | -0.018 | 0.0664 | No |
| 77 | PRPF3 |  |  | 5495 | -0.020 | 0.0573 | No |
| 78 | CDC5L |  |  | 5504 | -0.020 | 0.0582 | No |
| 79 | BCL2L11 |  |  | 5827 | -0.026 | 0.0274 | No |
| 80 | CDK2 |  |  | 5964 | -0.029 | 0.0159 | No |
| 81 | PARP2 |  |  | 5986 | -0.029 | 0.0162 | No |
| 82 | GRINA |  |  | 6036 | -0.030 | 0.0137 | No |
| 83 | POLG2 |  |  | 6450 | -0.037 | -0.0255 | No |
| 84 | MMP14 |  |  | 6548 | -0.039 | -0.0321 | No |
| 85 | CREG1 |  |  | 6627 | -0.041 | -0.0366 | No |
| 86 | KCNH2 |  |  | 6689 | -0.043 | -0.0392 | No |
| 87 | GLS |  |  | 6754 | -0.044 | -0.0420 | No |
| 88 | ATP6V1C1 |  |  | 6768 | -0.044 | -0.0396 | No |
| 89 | PTPRD |  |  | 7218 | -0.056 | -0.0809 | No |
| 90 | OLFM1 |  |  | 7262 | -0.057 | -0.0805 | No |
| 91 | CNP |  |  | 7303 | -0.058 | -0.0796 | No |
| 92 | CLCN2 |  |  | 7354 | -0.059 | -0.0797 | No |
| 93 | E2F5 |  |  | 7540 | -0.064 | -0.0932 | No |
| 94 | DGAT1 |  |  | 7770 | -0.072 | -0.1106 | No |
| 95 | STARD3 |  |  | 7819 | -0.074 | -0.1092 | No |
| 96 | PDLIM3 |  |  | 8278 | -0.093 | -0.1482 | No |
| 97 | RFC4 |  |  | 8292 | -0.094 | -0.1416 | No |
| 98 | NPTXR |  |  | 8321 | -0.095 | -0.1364 | No |
| 99 | AGO2 |  |  | 8389 | -0.098 | -0.1350 | No |
| 100 | BSG |  |  | 8399 | -0.099 | -0.1275 | No |
| 101 | EIF2S3 |  |  | 8476 | -0.103 | -0.1265 | No |
| 102 | HNRNPU |  |  | 8616 | -0.112 | -0.1313 | No |
| 103 | TYRO3 |  |  | 8628 | -0.113 | -0.1229 | No |
| 104 | JUNB |  |  | 8634 | -0.113 | -0.1138 | No |
| 105 | CA2 |  |  | 8636 | -0.113 | -0.1043 | No |
| 106 | GGH |  |  | 8643 | -0.114 | -0.0953 | No |
| 107 | TFRC |  |  | 8715 | -0.119 | -0.0925 | No |
| 108 | NTRK3 |  |  | 8930 | -0.137 | -0.1029 | No |
| 109 | CHRNA5 |  |  | 9010 | -0.143 | -0.0988 | No |
| 110 | SLC6A8 |  |  | 9101 | -0.152 | -0.0952 | No |
| 111 | LHX2 |  |  | 9142 | -0.157 | -0.0860 | No |
| 112 | IGFBP2 |  |  | 9209 | -0.167 | -0.0786 | No |
| 113 | RPN1 |  |  | 9243 | -0.171 | -0.0675 | No |
| 114 | BTG2 |  |  | 9304 | -0.181 | -0.0583 | No |
| 115 | HYAL2 |  |  | 9308 | -0.183 | -0.0432 | No |
| 116 | PPT1 |  |  | 9318 | -0.184 | -0.0285 | No |
| 117 | HSPA13 |  |  | 9428 | -0.206 | -0.0222 | No |
| 118 | IL6ST |  |  | 9519 | -0.231 | -0.0119 | No |
| 119 | FOS |  |  | 9566 | -0.247 | 0.0042 | No |
| 120 | TMBIM6 |  |  | 9716 | -0.328 | 0.0167 | No |
Table: GSEA details [plain text format]

  

Fig 2: HALLMARK\_UV\_RESPONSE\_UP: Random ES distribution      
 Gene set null distribution of ES for **HALLMARK\_UV\_RESPONSE\_UP**

  
