# Supplementary material for: Modeling glioblastoma heterogeneity as a dynamic network of cell states
Source: Mol Syst Biol. 2021 Sep 16;17(9):e10105. doi: 10.15252/msb.202010105 (PMC8444284; doi:10.15252/msb.202010105)
Supplement: Supplementary file 5 — Source Data for Figure 3 [file MSB-17-e10105-s001.zip › Figure3A_sourcedata/GSEA_3065/hallmarks_state1.GseaPreranked.1623416262439/HALLMARK_WNT_BETA_CATENIN_SIGNALING.html]

Details for gene set HALLMARK\_WNT\_BETA\_CATENIN\_SIGNALING[GSEA]

|  || Dataset | state1 |
| Phenotype | NoPhenotypeAvailable |
| Upregulated in class | na\_neg |
| GeneSet | HALLMARK\_WNT\_BETA\_CATENIN\_SIGNALING |
| Enrichment Score (ES) | -0.44490144 |
| Normalized Enrichment Score (NES) | -1.2434667 |
| Nominal p-value | 0.18467583 |
| FDR q-value | 0.4103693 |
| FWER p-Value | 0.937 |
Table: GSEA Results Summary

  

Fig 1: Enrichment plot: HALLMARK\_WNT\_BETA\_CATENIN\_SIGNALING      
 Profile of the Running ES Score & Positions of GeneSet Members on the Rank Ordered List

  

| PROBE | GENE SYMBOL | GENE\_TITLE | RANK IN GENE LIST | RANK METRIC SCORE | RUNNING ES | CORE ENRICHMENT || 1 | DKK1 |  |  | 16 | 0.656 | 0.2001 | No |
| 2 | NCOR2 |  |  | 1294 | 0.104 | 0.1024 | No |
| 3 | LEF1 |  |  | 1861 | 0.067 | 0.0655 | No |
| 4 | GNAI1 |  |  | 2013 | 0.059 | 0.0684 | No |
| 5 | HDAC2 |  |  | 2724 | 0.032 | 0.0062 | No |
| 6 | AXIN1 |  |  | 3073 | 0.023 | -0.0220 | No |
| 7 | CUL1 |  |  | 3336 | 0.017 | -0.0433 | No |
| 8 | MYC |  |  | 3964 | 0.005 | -0.1054 | No |
| 9 | KAT2A |  |  | 4427 | -0.003 | -0.1513 | No |
| 10 | PSEN2 |  |  | 5019 | -0.013 | -0.2073 | No |
| 11 | SKP2 |  |  | 5067 | -0.014 | -0.2079 | No |
| 12 | DVL2 |  |  | 5158 | -0.015 | -0.2124 | No |
| 13 | TP53 |  |  | 5557 | -0.021 | -0.2463 | No |
| 14 | MAML1 |  |  | 5560 | -0.021 | -0.2400 | No |
| 15 | HDAC5 |  |  | 6550 | -0.039 | -0.3283 | No |
| 16 | TCF7 |  |  | 6790 | -0.045 | -0.3387 | No |
| 17 | NUMB |  |  | 6873 | -0.046 | -0.3328 | No |
| 18 | JAG1 |  |  | 7426 | -0.061 | -0.3700 | No |
| 19 | RBPJ |  |  | 7588 | -0.066 | -0.3661 | No |
| 20 | FZD1 |  |  | 7685 | -0.069 | -0.3546 | No |
| 21 | NCSTN |  |  | 8576 | -0.110 | -0.4111 | Yes |
| 22 | CSNK1E |  |  | 8730 | -0.120 | -0.3896 | Yes |
| 23 | PTCH1 |  |  | 8777 | -0.123 | -0.3564 | Yes |
| 24 | HEY1 |  |  | 9215 | -0.168 | -0.3491 | Yes |
| 25 | ADAM17 |  |  | 9248 | -0.172 | -0.2994 | Yes |
| 26 | CTNNB1 |  |  | 9294 | -0.179 | -0.2488 | Yes |
| 27 | NOTCH1 |  |  | 9780 | -0.391 | -0.1779 | Yes |
| 28 | CCND2 |  |  | 9842 | -0.611 | 0.0038 | Yes |
Table: GSEA details [plain text format]

  

Fig 2: HALLMARK\_WNT\_BETA\_CATENIN\_SIGNALING: Random ES distribution      
 Gene set null distribution of ES for **HALLMARK\_WNT\_BETA\_CATENIN\_SIGNALING**

  
