# Supplementary material for: Modeling glioblastoma heterogeneity as a dynamic network of cell states
Source: Mol Syst Biol. 2021 Sep 16;17(9):e10105. doi: 10.15252/msb.202010105 (PMC8444284; doi:10.15252/msb.202010105)
Supplement: Supplementary file 5 — Source Data for Figure 3 [file MSB-17-e10105-s001.zip › Figure3A_sourcedata/GSEA_3065/hallmarks_state1.GseaPreranked.1623416262439/index.html]

Index for xtools.gsea.GseaPreranked hallmarks\_state1.GseaPreranked.1623416262439

### GSEA Report for Dataset state1

#### Enrichment in phenotype: **na**

- 34 / 49 gene sets are upregulated in phenotype **na\_pos**- 21 gene sets are significant at FDR < 25%- 12 gene sets are significantly enriched at nominal pvalue < 1%- 14 gene sets are significantly enriched at nominal pvalue < 5%- Snapshot of enrichment results- Detailed enrichment results in html format- Detailed enrichment results in excel format (tab delimited text)- Guide to interpret results

#### Enrichment in phenotype: **na**

- 15 / 49 gene sets are upregulated in phenotype **na\_neg**- 0 gene sets are significantly enriched at FDR < 25%- 0 gene sets are significantly enriched at nominal pvalue < 1%- 0 gene sets are significantly enriched at nominal pvalue < 5%- Snapshot of enrichment results- Detailed enrichment results in html format- Detailed enrichment results in excel format (tab delimited text)- Guide to interpret results

#### Dataset details

- The dataset has 9880 features (genes)- No probe set => gene symbol collapsing was requested, so all 9880 features were used

#### Gene set details

- Gene set size filters (min=15, max=500) resulted in filtering out 1 / 50 gene sets- The remaining 49 gene sets were used in the analysis- List of gene sets used and their sizes (restricted to features in the specified dataset)

#### Gene markers for the **na\_pos** *versus* **na\_neg** comparison

- The dataset has 9880 features (genes)- Detailed rank ordered gene list for all features in the dataset

#### Global statistics and plots

- Plot of p-values *vs.* NES- Global ES histogram

#### Other

- Parameters used for this analysis

#### Comments

- Timestamp used as the random seed: 1623416264813

---

Report: hallmarks\_state1.GseaPreranked.1623416262439.rpt   by user: idala384

xtools.gsea.GseaPreranked [Fri, Jun 11, '21 2 PM 57]

Website: www.gsea-msigdb.org/gsea
Questions & Suggestions: Contact page
