# Supplementary material for: Modeling glioblastoma heterogeneity as a dynamic network of cell states
Source: Mol Syst Biol. 2021 Sep 16;17(9):e10105. doi: 10.15252/msb.202010105 (PMC8444284; doi:10.15252/msb.202010105)
Supplement: Supplementary file 5 — Source Data for Figure 3 [file MSB-17-e10105-s001.zip › Figure3A_sourcedata/GSEA_3065/hallmarks_state2.GseaPreranked.1623416269049/gsea_report_for_na_neg_1623416269049.html]

Report for na\_neg 1623416269049 [GSEA]

| GS  follow link to MSigDB | GS DETAILS | SIZE | ES | NES | NOM p-val | FDR q-val | FWER p-val | RANK AT MAX | LEADING EDGE || 1 | HALLMARK\_EPITHELIAL\_MESENCHYMAL\_TRANSITION | Details ... | 119 | -0.75 | -2.94 | 0.000 | 0.000 | 0.000 | 1426 | tags=70%, list=15%, signal=81% |
| 2 | HALLMARK\_MYOGENESIS | Details ... | 96 | -0.63 | -2.44 | 0.000 | 0.000 | 0.000 | 1908 | tags=55%, list=20%, signal=68% |
| 3 | HALLMARK\_ANGIOGENESIS | Details ... | 21 | -0.84 | -2.37 | 0.000 | 0.000 | 0.000 | 449 | tags=62%, list=5%, signal=65% |
| 4 | HALLMARK\_UV\_RESPONSE\_DN | Details ... | 103 | -0.60 | -2.36 | 0.000 | 0.000 | 0.000 | 1935 | tags=49%, list=20%, signal=60% |
| 5 | HALLMARK\_COAGULATION | Details ... | 55 | -0.63 | -2.18 | 0.000 | 0.000 | 0.000 | 798 | tags=35%, list=8%, signal=38% |
| 6 | HALLMARK\_HEDGEHOG\_SIGNALING | Details ... | 23 | -0.73 | -2.08 | 0.000 | 0.000 | 0.000 | 1303 | tags=48%, list=14%, signal=55% |
| 7 | HALLMARK\_HYPOXIA | Details ... | 130 | -0.51 | -2.06 | 0.000 | 0.000 | 0.000 | 1437 | tags=39%, list=15%, signal=46% |
| 8 | HALLMARK\_COMPLEMENT | Details ... | 93 | -0.53 | -2.06 | 0.000 | 0.000 | 0.000 | 805 | tags=25%, list=9%, signal=27% |
| 9 | HALLMARK\_INFLAMMATORY\_RESPONSE | Details ... | 70 | -0.56 | -2.02 | 0.000 | 0.000 | 0.000 | 2005 | tags=54%, list=21%, signal=68% |
| 10 | HALLMARK\_KRAS\_SIGNALING\_UP | Details ... | 86 | -0.53 | -2.00 | 0.000 | 0.000 | 0.000 | 1342 | tags=34%, list=14%, signal=39% |
| 11 | HALLMARK\_TNFA\_SIGNALING\_VIA\_NFKB | Details ... | 106 | -0.49 | -1.95 | 0.000 | 0.000 | 0.000 | 1780 | tags=41%, list=19%, signal=49% |
| 12 | HALLMARK\_IL6\_JAK\_STAT3\_SIGNALING | Details ... | 34 | -0.62 | -1.92 | 0.000 | 0.000 | 0.001 | 1879 | tags=53%, list=20%, signal=66% |
| 13 | HALLMARK\_CHOLESTEROL\_HOMEOSTASIS | Details ... | 63 | -0.52 | -1.84 | 0.000 | 0.002 | 0.009 | 1589 | tags=38%, list=17%, signal=45% |
| 14 | HALLMARK\_IL2\_STAT5\_SIGNALING | Details ... | 106 | -0.47 | -1.82 | 0.000 | 0.002 | 0.010 | 1044 | tags=28%, list=11%, signal=31% |
| 15 | HALLMARK\_APICAL\_SURFACE | Details ... | 22 | -0.61 | -1.76 | 0.003 | 0.003 | 0.018 | 574 | tags=27%, list=6%, signal=29% |
| 16 | HALLMARK\_ESTROGEN\_RESPONSE\_EARLY | Details ... | 104 | -0.42 | -1.65 | 0.000 | 0.007 | 0.043 | 1437 | tags=32%, list=15%, signal=37% |
| 17 | HALLMARK\_PROTEIN\_SECRETION | Details ... | 89 | -0.42 | -1.63 | 0.004 | 0.010 | 0.062 | 1544 | tags=33%, list=16%, signal=39% |
| 18 | HALLMARK\_APICAL\_JUNCTION | Details ... | 116 | -0.40 | -1.59 | 0.009 | 0.012 | 0.081 | 1674 | tags=34%, list=18%, signal=41% |
| 19 | HALLMARK\_P53\_PATHWAY | Details ... | 135 | -0.39 | -1.56 | 0.000 | 0.015 | 0.107 | 1292 | tags=28%, list=14%, signal=32% |
| 20 | HALLMARK\_APOPTOSIS | Details ... | 106 | -0.38 | -1.48 | 0.008 | 0.027 | 0.198 | 722 | tags=24%, list=8%, signal=25% |
| 21 | HALLMARK\_INTERFERON\_ALPHA\_RESPONSE |  | 55 | -0.42 | -1.47 | 0.010 | 0.028 | 0.214 | 2687 | tags=42%, list=28%, signal=58% |
| 22 | HALLMARK\_ANDROGEN\_RESPONSE |  | 72 | -0.36 | -1.33 | 0.052 | 0.079 | 0.495 | 712 | tags=24%, list=8%, signal=25% |
| 23 | HALLMARK\_HEME\_METABOLISM |  | 126 | -0.33 | -1.31 | 0.026 | 0.085 | 0.546 | 1606 | tags=25%, list=17%, signal=30% |
| 24 | HALLMARK\_TGF\_BETA\_SIGNALING |  | 43 | -0.37 | -1.24 | 0.115 | 0.132 | 0.726 | 1069 | tags=26%, list=11%, signal=29% |
| 25 | HALLMARK\_INTERFERON\_GAMMA\_RESPONSE |  | 94 | -0.33 | -1.24 | 0.085 | 0.128 | 0.731 | 1479 | tags=22%, list=16%, signal=26% |
| 26 | HALLMARK\_GLYCOLYSIS |  | 146 | -0.30 | -1.19 | 0.079 | 0.177 | 0.846 | 1044 | tags=23%, list=11%, signal=25% |
| 27 | HALLMARK\_WNT\_BETA\_CATENIN\_SIGNALING |  | 27 | -0.39 | -1.15 | 0.234 | 0.211 | 0.911 | 2184 | tags=44%, list=23%, signal=58% |
| 28 | HALLMARK\_KRAS\_SIGNALING\_DN |  | 32 | -0.37 | -1.14 | 0.251 | 0.227 | 0.936 | 772 | tags=19%, list=8%, signal=20% |
| 29 | HALLMARK\_NOTCH\_SIGNALING |  | 23 | -0.35 | -1.00 | 0.445 | 0.490 | 0.999 | 679 | tags=26%, list=7%, signal=28% |
| 30 | HALLMARK\_XENOBIOTIC\_METABOLISM |  | 100 | -0.25 | -0.98 | 0.522 | 0.522 | 1.000 | 1283 | tags=23%, list=14%, signal=26% |
| 31 | HALLMARK\_BILE\_ACID\_METABOLISM |  | 54 | -0.28 | -0.97 | 0.503 | 0.525 | 1.000 | 2433 | tags=41%, list=26%, signal=55% |
Table: Gene sets enriched in phenotype **na**[plain text format]****

  
