# Supplementary material for: Modeling glioblastoma heterogeneity as a dynamic network of cell states
Source: Mol Syst Biol. 2021 Sep 16;17(9):e10105. doi: 10.15252/msb.202010105 (PMC8444284; doi:10.15252/msb.202010105)
Supplement: Supplementary file 6 — Source Data for Figure 5 [file MSB-17-e10105-s004.zip › Figure5A_sourcedata/GSEA_3017/hallmarks_stateA.GseaPreranked.1621934654007/gsea_report_for_na_neg_1621934654007.html]

Report for na\_neg 1621934654007 [GSEA]

| GS  follow link to MSigDB | GS DETAILS | SIZE | ES | NES | NOM p-val | FDR q-val | FWER p-val | RANK AT MAX | LEADING EDGE || 1 | HALLMARK\_EPITHELIAL\_MESENCHYMAL\_TRANSITION | Details ... | 45 | -0.55 | -2.78 | 0.000 | 0.000 | 0.000 | 207 | tags=64%, list=21%, signal=78% |
| 2 | HALLMARK\_APOPTOSIS | Details ... | 26 | -0.54 | -2.40 | 0.000 | 0.000 | 0.000 | 194 | tags=58%, list=20%, signal=70% |
| 3 | HALLMARK\_UV\_RESPONSE\_DN | Details ... | 28 | -0.52 | -2.34 | 0.000 | 0.000 | 0.000 | 199 | tags=54%, list=20%, signal=65% |
| 4 | HALLMARK\_TNFA\_SIGNALING\_VIA\_NFKB | Details ... | 31 | -0.51 | -2.26 | 0.002 | 0.000 | 0.001 | 262 | tags=65%, list=27%, signal=85% |
| 5 | HALLMARK\_MYOGENESIS | Details ... | 19 | -0.52 | -1.99 | 0.006 | 0.011 | 0.049 | 216 | tags=63%, list=22%, signal=79% |
| 6 | HALLMARK\_INFLAMMATORY\_RESPONSE | Details ... | 20 | -0.44 | -1.77 | 0.014 | 0.035 | 0.174 | 226 | tags=50%, list=23%, signal=64% |
| 7 | HALLMARK\_P53\_PATHWAY | Details ... | 22 | -0.42 | -1.74 | 0.023 | 0.037 | 0.207 | 191 | tags=50%, list=19%, signal=61% |
| 8 | HALLMARK\_HYPOXIA | Details ... | 35 | -0.33 | -1.59 | 0.036 | 0.080 | 0.461 | 225 | tags=46%, list=23%, signal=57% |
| 9 | HALLMARK\_KRAS\_SIGNALING\_UP | Details ... | 19 | -0.31 | -1.26 | 0.196 | 0.322 | 0.940 | 412 | tags=68%, list=42%, signal=115% |
| 10 | HALLMARK\_IL2\_STAT5\_SIGNALING | Details ... | 20 | -0.29 | -1.15 | 0.296 | 0.411 | 0.975 | 62 | tags=20%, list=6%, signal=21% |
| 11 | HALLMARK\_ESTROGEN\_RESPONSE\_EARLY | Details ... | 19 | -0.26 | -1.02 | 0.444 | 0.553 | 0.998 | 166 | tags=32%, list=17%, signal=37% |
| 12 | HALLMARK\_ANDROGEN\_RESPONSE | Details ... | 17 | -0.27 | -1.00 | 0.476 | 0.536 | 1.000 | 112 | tags=24%, list=11%, signal=26% |
| 13 | HALLMARK\_MTORC1\_SIGNALING | Details ... | 30 | -0.18 | -0.83 | 0.639 | 0.726 | 1.000 | 20 | tags=7%, list=2%, signal=7% |
| 14 | HALLMARK\_APICAL\_JUNCTION | Details ... | 16 | -0.21 | -0.77 | 0.743 | 0.745 | 1.000 | 417 | tags=63%, list=42%, signal=106% |
Table: Gene sets enriched in phenotype **na**[plain text format]****

  
