# Supplementary material for: Modeling glioblastoma heterogeneity as a dynamic network of cell states
Source: Mol Syst Biol. 2021 Sep 16;17(9):e10105. doi: 10.15252/msb.202010105 (PMC8444284; doi:10.15252/msb.202010105)
Supplement: Supplementary file 6 — Source Data for Figure 5 [file MSB-17-e10105-s004.zip › Figure5A_sourcedata/GSEA_3017/hallmarks_stateA.GseaPreranked.1621934654007/gsea_report_for_na_pos_1621934654007.html]

Report for na\_pos 1621934654007 [GSEA]

| GS  follow link to MSigDB | GS DETAILS | SIZE | ES | NES | NOM p-val | FDR q-val | FWER p-val | RANK AT MAX | LEADING EDGE || 1 | HALLMARK\_G2M\_CHECKPOINT | Details ... | 62 | 0.49 | 3.19 | 0.000 | 0.000 | 0.000 | 197 | tags=52%, list=20%, signal=60% |
| 2 | HALLMARK\_E2F\_TARGETS | Details ... | 63 | 0.43 | 2.82 | 0.000 | 0.000 | 0.000 | 471 | tags=86%, list=48%, signal=153% |
| 3 | HALLMARK\_MYC\_TARGETS\_V1 | Details ... | 31 | 0.46 | 2.45 | 0.000 | 0.000 | 0.000 | 542 | tags=100%, list=55%, signal=215% |
| 4 | HALLMARK\_MITOTIC\_SPINDLE | Details ... | 48 | 0.39 | 2.43 | 0.000 | 0.000 | 0.000 | 270 | tags=56%, list=27%, signal=74% |
| 5 | HALLMARK\_GLYCOLYSIS | Details ... | 29 | 0.27 | 1.40 | 0.117 | 0.125 | 0.571 | 119 | tags=28%, list=12%, signal=30% |
| 6 | HALLMARK\_ESTROGEN\_RESPONSE\_LATE | Details ... | 25 | 0.22 | 1.07 | 0.352 | 0.368 | 0.948 | 151 | tags=24%, list=15%, signal=28% |
Table: Gene sets enriched in phenotype **na**[plain text format]****

  
