# Supplementary material for: Modeling glioblastoma heterogeneity as a dynamic network of cell states
Source: Mol Syst Biol. 2021 Sep 16;17(9):e10105. doi: 10.15252/msb.202010105 (PMC8444284; doi:10.15252/msb.202010105)
Supplement: Supplementary file 6 — Source Data for Figure 5 [file MSB-17-e10105-s004.zip › Figure5A_sourcedata/GSEA_3017/hallmarks_stateA.GseaPreranked.1621934654007/HALLMARK_ANDROGEN_RESPONSE.html]

Details for gene set HALLMARK\_ANDROGEN\_RESPONSE[GSEA]

|  || Dataset | state53017 |
| Phenotype | NoPhenotypeAvailable |
| Upregulated in class | na\_neg |
| GeneSet | HALLMARK\_ANDROGEN\_RESPONSE |
| Enrichment Score (ES) | -0.26673996 |
| Normalized Enrichment Score (NES) | -0.99806404 |
| Nominal p-value | 0.4761062 |
| FDR q-value | 0.5359065 |
| FWER p-Value | 1.0 |
Table: GSEA Results Summary

  

Fig 1: Enrichment plot: HALLMARK\_ANDROGEN\_RESPONSE      
 Profile of the Running ES Score & Positions of GeneSet Members on the Rank Ordered List

  

| PROBE | GENE SYMBOL | GENE\_TITLE | RANK IN GENE LIST | RANK METRIC SCORE | RUNNING ES | CORE ENRICHMENT || 1 | DBI |  |  | 17 | 0.683 | 0.0825 | No |
| 2 | SLC26A2 |  |  | 121 | 0.428 | 0.0390 | No |
| 3 | CENPN |  |  | 310 | 0.323 | -0.1074 | No |
| 4 | SORD |  |  | 317 | 0.320 | -0.0668 | No |
| 5 | IDI1 |  |  | 334 | 0.314 | -0.0372 | No |
| 6 | FKBP5 |  |  | 368 | 0.304 | -0.0267 | No |
| 7 | PA2G4 |  |  | 402 | 0.293 | -0.0179 | No |
| 8 | SMS |  |  | 463 | 0.278 | -0.0390 | No |
| 9 | UBE2I |  |  | 564 | 0.257 | -0.1043 | No |
| 10 | TPD52 |  |  | 566 | 0.257 | -0.0678 | No |
| 11 | ZMIZ1 |  |  | 573 | 0.255 | -0.0366 | No |
| 12 | ITGAV |  |  | 599 | -0.250 | -0.0258 | No |
| 13 | AKAP12 |  |  | 690 | -0.296 | -0.0752 | No |
| 14 | PMEPA1 |  |  | 877 | -0.497 | -0.1940 | Yes |
| 15 | SAT1 |  |  | 909 | -0.573 | -0.1421 | Yes |
| 16 | ARID5B |  |  | 915 | -0.586 | -0.0615 | Yes |
| 17 | CDK6 |  |  | 966 | -0.920 | 0.0216 | Yes |
Table: GSEA details [plain text format]

  

Fig 2: HALLMARK\_ANDROGEN\_RESPONSE: Random ES distribution      
 Gene set null distribution of ES for **HALLMARK\_ANDROGEN\_RESPONSE**

  
