# Supplementary material for: Modeling glioblastoma heterogeneity as a dynamic network of cell states
Source: Mol Syst Biol. 2021 Sep 16;17(9):e10105. doi: 10.15252/msb.202010105 (PMC8444284; doi:10.15252/msb.202010105)
Supplement: Supplementary file 6 — Source Data for Figure 5 [file MSB-17-e10105-s004.zip › Figure5A_sourcedata/GSEA_3017/hallmarks_stateA.GseaPreranked.1621934654007/HALLMARK_APICAL_JUNCTION.html]

Details for gene set HALLMARK\_APICAL\_JUNCTION[GSEA]

|  || Dataset | state53017 |
| Phenotype | NoPhenotypeAvailable |
| Upregulated in class | na\_neg |
| GeneSet | HALLMARK\_APICAL\_JUNCTION |
| Enrichment Score (ES) | -0.21130124 |
| Normalized Enrichment Score (NES) | -0.76816964 |
| Nominal p-value | 0.7432432 |
| FDR q-value | 0.7446764 |
| FWER p-Value | 1.0 |
Table: GSEA Results Summary

  

Fig 1: Enrichment plot: HALLMARK\_APICAL\_JUNCTION      
 Profile of the Running ES Score & Positions of GeneSet Members on the Rank Ordered List

  

| PROBE | GENE SYMBOL | GENE\_TITLE | RANK IN GENE LIST | RANK METRIC SCORE | RUNNING ES | CORE ENRICHMENT || 1 | ZYX |  |  | 136 | 0.416 | -0.0657 | No |
| 2 | EPB41L2 |  |  | 208 | 0.371 | -0.0727 | No |
| 3 | TIAL1 |  |  | 218 | 0.366 | -0.0167 | No |
| 4 | ITGB1 |  |  | 256 | 0.346 | 0.0069 | No |
| 5 | NECTIN3 |  |  | 374 | 0.302 | -0.0597 | No |
| 6 | ADAM23 |  |  | 449 | 0.281 | -0.0858 | No |
| 7 | PTEN |  |  | 572 | 0.255 | -0.1658 | Yes |
| 8 | CD99 |  |  | 604 | -0.252 | -0.1527 | Yes |
| 9 | DLG1 |  |  | 611 | -0.257 | -0.1131 | Yes |
| 10 | SDC3 |  |  | 637 | -0.264 | -0.0917 | Yes |
| 11 | IRS1 |  |  | 657 | -0.273 | -0.0627 | Yes |
| 12 | ARHGEF6 |  |  | 659 | -0.273 | -0.0150 | Yes |
| 13 | THY1 |  |  | 663 | -0.277 | 0.0313 | Yes |
| 14 | CDH4 |  |  | 794 | -0.376 | -0.0353 | Yes |
| 15 | CDH6 |  |  | 868 | -0.472 | -0.0264 | Yes |
| 16 | MMP2 |  |  | 953 | -0.829 | 0.0350 | Yes |
Table: GSEA details [plain text format]

  

Fig 2: HALLMARK\_APICAL\_JUNCTION: Random ES distribution      
 Gene set null distribution of ES for **HALLMARK\_APICAL\_JUNCTION**

  
