# Supplementary material for: Modeling glioblastoma heterogeneity as a dynamic network of cell states
Source: Mol Syst Biol. 2021 Sep 16;17(9):e10105. doi: 10.15252/msb.202010105 (PMC8444284; doi:10.15252/msb.202010105)
Supplement: Supplementary file 6 — Source Data for Figure 5 [file MSB-17-e10105-s004.zip › Figure5A_sourcedata/GSEA_3017/hallmarks_stateA.GseaPreranked.1621934654007/HALLMARK_APOPTOSIS.html]

Details for gene set HALLMARK\_APOPTOSIS[GSEA]

|  || Dataset | state53017 |
| Phenotype | NoPhenotypeAvailable |
| Upregulated in class | na\_neg |
| GeneSet | HALLMARK\_APOPTOSIS |
| Enrichment Score (ES) | -0.5438673 |
| Normalized Enrichment Score (NES) | -2.4008327 |
| Nominal p-value | 0.0 |
| FDR q-value | 0.0 |
| FWER p-Value | 0.0 |
Table: GSEA Results Summary

  

Fig 1: Enrichment plot: HALLMARK\_APOPTOSIS      
 Profile of the Running ES Score & Positions of GeneSet Members on the Rank Ordered List

  

| PROBE | GENE SYMBOL | GENE\_TITLE | RANK IN GENE LIST | RANK METRIC SCORE | RUNNING ES | CORE ENRICHMENT || 1 | VDAC2 |  |  | 54 | 0.514 | -0.0185 | No |
| 2 | TOP2A |  |  | 95 | 0.457 | -0.0267 | No |
| 3 | MGMT |  |  | 301 | 0.325 | -0.2161 | No |
| 4 | HSPB1 |  |  | 312 | 0.322 | -0.2030 | No |
| 5 | SLC20A1 |  |  | 361 | 0.306 | -0.2305 | No |
| 6 | BRCA1 |  |  | 373 | 0.302 | -0.2199 | No |
| 7 | HMGB2 |  |  | 424 | 0.288 | -0.2508 | No |
| 8 | DPYD |  |  | 630 | -0.262 | -0.4448 | No |
| 9 | PEA15 |  |  | 668 | -0.281 | -0.4627 | No |
| 10 | EMP1 |  |  | 724 | -0.322 | -0.4964 | No |
| 11 | TIMP2 |  |  | 737 | -0.332 | -0.4846 | No |
| 12 | SQSTM1 |  |  | 795 | -0.379 | -0.5162 | Yes |
| 13 | APP |  |  | 812 | -0.394 | -0.5041 | Yes |
| 14 | ATF3 |  |  | 836 | -0.428 | -0.4967 | Yes |
| 15 | TGFB2 |  |  | 840 | -0.432 | -0.4682 | Yes |
| 16 | BNIP3L |  |  | 841 | -0.432 | -0.4367 | Yes |
| 17 | JUN |  |  | 886 | -0.525 | -0.4441 | Yes |
| 18 | SAT1 |  |  | 909 | -0.573 | -0.4251 | Yes |
| 19 | NEDD9 |  |  | 923 | -0.625 | -0.3929 | Yes |
| 20 | CLU |  |  | 938 | -0.685 | -0.3574 | Yes |
| 21 | MMP2 |  |  | 953 | -0.829 | -0.3113 | Yes |
| 22 | IFITM3 |  |  | 954 | -0.830 | -0.2507 | Yes |
| 23 | CD44 |  |  | 957 | -0.851 | -0.1906 | Yes |
| 24 | CDKN1A |  |  | 969 | -0.987 | -0.1299 | Yes |
| 25 | LGALS3 |  |  | 970 | -0.989 | -0.0577 | Yes |
| 26 | ANXA1 |  |  | 972 | -1.018 | 0.0156 | Yes |
Table: GSEA details [plain text format]

  

Fig 2: HALLMARK\_APOPTOSIS: Random ES distribution      
 Gene set null distribution of ES for **HALLMARK\_APOPTOSIS**

  
