# Supplementary material for: Modeling glioblastoma heterogeneity as a dynamic network of cell states
Source: Mol Syst Biol. 2021 Sep 16;17(9):e10105. doi: 10.15252/msb.202010105 (PMC8444284; doi:10.15252/msb.202010105)
Supplement: Supplementary file 6 — Source Data for Figure 5 [file MSB-17-e10105-s004.zip › Figure5A_sourcedata/GSEA_3017/hallmarks_stateA.GseaPreranked.1621934654007/HALLMARK_E2F_TARGETS.html]

Details for gene set HALLMARK\_E2F\_TARGETS[GSEA]

|  || Dataset | state53017 |
| Phenotype | NoPhenotypeAvailable |
| Upregulated in class | na\_pos |
| GeneSet | HALLMARK\_E2F\_TARGETS |
| Enrichment Score (ES) | 0.42562902 |
| Normalized Enrichment Score (NES) | 2.8185694 |
| Nominal p-value | 0.0 |
| FDR q-value | 0.0 |
| FWER p-Value | 0.0 |
Table: GSEA Results Summary

  

Fig 1: Enrichment plot: HALLMARK\_E2F\_TARGETS      
 Profile of the Running ES Score & Positions of GeneSet Members on the Rank Ordered List

  

| PROBE | GENE SYMBOL | GENE\_TITLE | RANK IN GENE LIST | RANK METRIC SCORE | RUNNING ES | CORE ENRICHMENT || 1 | CDK1 |  |  | 3 | 1.006 | 0.0366 | Yes |
| 2 | MKI67 |  |  | 4 | 0.926 | 0.0733 | Yes |
| 3 | KPNA2 |  |  | 20 | 0.667 | 0.0835 | Yes |
| 4 | CKS2 |  |  | 40 | 0.587 | 0.0862 | Yes |
| 5 | HMMR |  |  | 43 | 0.559 | 0.1062 | Yes |
| 6 | CKS1B |  |  | 50 | 0.530 | 0.1207 | Yes |
| 7 | SMC3 |  |  | 58 | 0.504 | 0.1331 | Yes |
| 8 | CENPE |  |  | 62 | 0.496 | 0.1495 | Yes |
| 9 | MYBL2 |  |  | 69 | 0.486 | 0.1622 | Yes |
| 10 | DLGAP5 |  |  | 76 | 0.475 | 0.1746 | Yes |
| 11 | AURKA |  |  | 78 | 0.474 | 0.1923 | Yes |
| 12 | CDC20 |  |  | 80 | 0.472 | 0.2099 | Yes |
| 13 | UBE2T |  |  | 83 | 0.470 | 0.2263 | Yes |
| 14 | DUT |  |  | 87 | 0.466 | 0.2415 | Yes |
| 15 | PLK1 |  |  | 89 | 0.464 | 0.2588 | Yes |
| 16 | TOP2A |  |  | 95 | 0.457 | 0.2715 | Yes |
| 17 | AURKB |  |  | 108 | 0.446 | 0.2762 | Yes |
| 18 | CDCA8 |  |  | 115 | 0.437 | 0.2870 | Yes |
| 19 | DEPDC1 |  |  | 119 | 0.432 | 0.3009 | Yes |
| 20 | MELK |  |  | 123 | 0.427 | 0.3146 | Yes |
| 21 | JPT1 |  |  | 125 | 0.426 | 0.3303 | Yes |
| 22 | BUB1B |  |  | 129 | 0.421 | 0.3438 | Yes |
| 23 | HELLS |  |  | 135 | 0.417 | 0.3549 | Yes |
| 24 | CCNB2 |  |  | 147 | 0.411 | 0.3593 | Yes |
| 25 | MAD2L1 |  |  | 153 | 0.406 | 0.3699 | Yes |
| 26 | KIF2C |  |  | 165 | 0.397 | 0.3738 | Yes |
| 27 | MYC |  |  | 169 | 0.395 | 0.3862 | Yes |
| 28 | MCM7 |  |  | 171 | 0.394 | 0.4007 | Yes |
| 29 | KIF22 |  |  | 197 | 0.377 | 0.3886 | Yes |
| 30 | CDCA3 |  |  | 203 | 0.372 | 0.3979 | Yes |
| 31 | TRIP13 |  |  | 209 | 0.370 | 0.4072 | Yes |
| 32 | SMC4 |  |  | 217 | 0.367 | 0.4142 | Yes |
| 33 | SPC25 |  |  | 226 | 0.363 | 0.4199 | Yes |
| 34 | TUBB |  |  | 264 | 0.340 | 0.3934 | Yes |
| 35 | ORC6 |  |  | 283 | 0.333 | 0.3871 | Yes |
| 36 | MTHFD2 |  |  | 287 | 0.330 | 0.3969 | Yes |
| 37 | BRCA2 |  |  | 308 | 0.323 | 0.3881 | Yes |
| 38 | RFC3 |  |  | 337 | 0.314 | 0.3702 | Yes |
| 39 | BRCA1 |  |  | 373 | 0.302 | 0.3444 | Yes |
| 40 | RPA3 |  |  | 381 | 0.300 | 0.3487 | Yes |
| 41 | CSE1L |  |  | 382 | 0.300 | 0.3606 | Yes |
| 42 | RRM2 |  |  | 383 | 0.300 | 0.3724 | Yes |
| 43 | HMGA1 |  |  | 397 | 0.295 | 0.3701 | Yes |
| 44 | RACGAP1 |  |  | 399 | 0.293 | 0.3806 | Yes |
| 45 | PA2G4 |  |  | 402 | 0.293 | 0.3900 | Yes |
| 46 | HMGB2 |  |  | 424 | 0.288 | 0.3787 | Yes |
| 47 | NOLC1 |  |  | 425 | 0.288 | 0.3901 | Yes |
| 48 | POP7 |  |  | 427 | 0.287 | 0.4004 | Yes |
| 49 | NCAPD2 |  |  | 440 | 0.283 | 0.3987 | Yes |
| 50 | LMNB1 |  |  | 442 | 0.282 | 0.4087 | Yes |
| 51 | ANP32E |  |  | 453 | 0.280 | 0.4090 | Yes |
| 52 | GINS1 |  |  | 462 | 0.279 | 0.4114 | Yes |
| 53 | ATAD2 |  |  | 470 | 0.275 | 0.4147 | Yes |
| 54 | EZH2 |  |  | 471 | 0.275 | 0.4256 | Yes |
| 55 | KIF4A |  |  | 504 | 0.269 | 0.4017 | No |
| 56 | UBE2S |  |  | 507 | 0.269 | 0.4102 | No |
| 57 | NME1 |  |  | 509 | 0.269 | 0.4198 | No |
| 58 | RANBP1 |  |  | 536 | 0.263 | 0.4021 | No |
| 59 | CDKN3 |  |  | 539 | 0.263 | 0.4103 | No |
| 60 | SRSF2 |  |  | 542 | 0.262 | 0.4185 | No |
| 61 | DDX39A |  |  | 562 | 0.258 | 0.4082 | No |
| 62 | TMPO |  |  | 584 | 0.253 | 0.3955 | No |
| 63 | CDKN1A |  |  | 969 | -0.987 | 0.0195 | No |
Table: GSEA details [plain text format]

  

Fig 2: HALLMARK\_E2F\_TARGETS: Random ES distribution      
 Gene set null distribution of ES for **HALLMARK\_E2F\_TARGETS**

  
