# Supplementary material for: Modeling glioblastoma heterogeneity as a dynamic network of cell states
Source: Mol Syst Biol. 2021 Sep 16;17(9):e10105. doi: 10.15252/msb.202010105 (PMC8444284; doi:10.15252/msb.202010105)
Supplement: Supplementary file 6 — Source Data for Figure 5 [file MSB-17-e10105-s004.zip › Figure5A_sourcedata/GSEA_3017/hallmarks_stateA.GseaPreranked.1621934654007/HALLMARK_EPITHELIAL_MESENCHYMAL_TRANSITION.html]

Details for gene set HALLMARK\_EPITHELIAL\_MESENCHYMAL\_TRANSITION[GSEA]

|  || Dataset | state53017 |
| Phenotype | NoPhenotypeAvailable |
| Upregulated in class | na\_neg |
| GeneSet | HALLMARK\_EPITHELIAL\_MESENCHYMAL\_TRANSITION |
| Enrichment Score (ES) | -0.5522171 |
| Normalized Enrichment Score (NES) | -2.7835345 |
| Nominal p-value | 0.0 |
| FDR q-value | 0.0 |
| FWER p-Value | 0.0 |
Table: GSEA Results Summary

  

Fig 1: Enrichment plot: HALLMARK\_EPITHELIAL\_MESENCHYMAL\_TRANSITION      
 Profile of the Running ES Score & Positions of GeneSet Members on the Rank Ordered List

  

| PROBE | GENE SYMBOL | GENE\_TITLE | RANK IN GENE LIST | RANK METRIC SCORE | RUNNING ES | CORE ENRICHMENT || 1 | IGFBP3 |  |  | 26 | 0.641 | 0.0006 | No |
| 2 | VIM |  |  | 46 | 0.543 | 0.0043 | No |
| 3 | ADAM12 |  |  | 90 | 0.464 | -0.0209 | No |
| 4 | EMP3 |  |  | 101 | 0.452 | -0.0116 | No |
| 5 | TNC |  |  | 159 | 0.402 | -0.0544 | No |
| 6 | MCM7 |  |  | 171 | 0.394 | -0.0488 | No |
| 7 | SERPINE2 |  |  | 201 | 0.373 | -0.0632 | No |
| 8 | ITGB1 |  |  | 256 | 0.346 | -0.1052 | No |
| 9 | NT5E |  |  | 445 | 0.282 | -0.2922 | No |
| 10 | VEGFA |  |  | 546 | 0.261 | -0.3868 | No |
| 11 | ITGAV |  |  | 599 | -0.250 | -0.4310 | No |
| 12 | FLNA |  |  | 636 | -0.264 | -0.4575 | No |
| 13 | THY1 |  |  | 663 | -0.277 | -0.4729 | No |
| 14 | EFEMP2 |  |  | 686 | -0.294 | -0.4833 | No |
| 15 | ID2 |  |  | 727 | -0.323 | -0.5116 | No |
| 16 | NTM |  |  | 762 | -0.354 | -0.5321 | No |
| 17 | SPARC |  |  | 782 | -0.369 | -0.5360 | Yes |
| 18 | TPM1 |  |  | 784 | -0.369 | -0.5209 | Yes |
| 19 | BDNF |  |  | 790 | -0.374 | -0.5097 | Yes |
| 20 | GPC1 |  |  | 801 | -0.384 | -0.5035 | Yes |
| 21 | DST |  |  | 818 | -0.400 | -0.5028 | Yes |
| 22 | IGFBP2 |  |  | 822 | -0.404 | -0.4883 | Yes |
| 23 | GJA1 |  |  | 823 | -0.405 | -0.4705 | Yes |
| 24 | PRRX1 |  |  | 829 | -0.413 | -0.4576 | Yes |
| 25 | CD59 |  |  | 831 | -0.422 | -0.4402 | Yes |
| 26 | PLOD2 |  |  | 838 | -0.431 | -0.4276 | Yes |
| 27 | ELN |  |  | 852 | -0.445 | -0.4219 | Yes |
| 28 | CDH6 |  |  | 868 | -0.472 | -0.4171 | Yes |
| 29 | PMEPA1 |  |  | 877 | -0.497 | -0.4037 | Yes |
| 30 | JUN |  |  | 886 | -0.525 | -0.3892 | Yes |
| 31 | CALD1 |  |  | 889 | -0.527 | -0.3681 | Yes |
| 32 | ITGB5 |  |  | 903 | -0.558 | -0.3574 | Yes |
| 33 | SCG2 |  |  | 907 | -0.564 | -0.3358 | Yes |
| 34 | SAT1 |  |  | 909 | -0.573 | -0.3117 | Yes |
| 35 | CADM1 |  |  | 914 | -0.582 | -0.2904 | Yes |
| 36 | PTX3 |  |  | 919 | -0.603 | -0.2681 | Yes |
| 37 | DPYSL3 |  |  | 926 | -0.626 | -0.2470 | Yes |
| 38 | SPP1 |  |  | 927 | -0.630 | -0.2193 | Yes |
| 39 | APLP1 |  |  | 935 | -0.680 | -0.1968 | Yes |
| 40 | CDH2 |  |  | 947 | -0.762 | -0.1750 | Yes |
| 41 | SERPINE1 |  |  | 951 | -0.774 | -0.1442 | Yes |
| 42 | MMP2 |  |  | 953 | -0.829 | -0.1088 | Yes |
| 43 | CD44 |  |  | 957 | -0.851 | -0.0746 | Yes |
| 44 | COL5A2 |  |  | 958 | -0.856 | -0.0370 | Yes |
| 45 | PMP22 |  |  | 982 | -1.519 | 0.0053 | Yes |
Table: GSEA details [plain text format]

  

Fig 2: HALLMARK\_EPITHELIAL\_MESENCHYMAL\_TRANSITION: Random ES distribution      
 Gene set null distribution of ES for **HALLMARK\_EPITHELIAL\_MESENCHYMAL\_TRANSITION**

  
