# Supplementary material for: Modeling glioblastoma heterogeneity as a dynamic network of cell states
Source: Mol Syst Biol. 2021 Sep 16;17(9):e10105. doi: 10.15252/msb.202010105 (PMC8444284; doi:10.15252/msb.202010105)
Supplement: Supplementary file 6 — Source Data for Figure 5 [file MSB-17-e10105-s004.zip › Figure5A_sourcedata/GSEA_3017/hallmarks_stateA.GseaPreranked.1621934654007/HALLMARK_ESTROGEN_RESPONSE_EARLY.html]

Details for gene set HALLMARK\_ESTROGEN\_RESPONSE\_EARLY[GSEA]

|  || Dataset | state53017 |
| Phenotype | NoPhenotypeAvailable |
| Upregulated in class | na\_neg |
| GeneSet | HALLMARK\_ESTROGEN\_RESPONSE\_EARLY |
| Enrichment Score (ES) | -0.26259562 |
| Normalized Enrichment Score (NES) | -1.0177106 |
| Nominal p-value | 0.44366196 |
| FDR q-value | 0.55276436 |
| FWER p-Value | 0.998 |
Table: GSEA Results Summary

  

Fig 1: Enrichment plot: HALLMARK\_ESTROGEN\_RESPONSE\_EARLY      
 Profile of the Running ES Score & Positions of GeneSet Members on the Rank Ordered List

  

| PROBE | GENE SYMBOL | GENE\_TITLE | RANK IN GENE LIST | RANK METRIC SCORE | RUNNING ES | CORE ENRICHMENT || 1 | CA12 |  |  | 24 | 0.645 | 0.0562 | No |
| 2 | SLC26A2 |  |  | 121 | 0.428 | 0.0108 | No |
| 3 | MYC |  |  | 169 | 0.395 | 0.0118 | No |
| 4 | PPIF |  |  | 248 | 0.350 | -0.0248 | No |
| 5 | ADCY1 |  |  | 269 | 0.339 | -0.0029 | No |
| 6 | ARL3 |  |  | 318 | 0.319 | -0.0124 | No |
| 7 | FARP1 |  |  | 332 | 0.315 | 0.0137 | No |
| 8 | FKBP5 |  |  | 368 | 0.304 | 0.0157 | No |
| 9 | ABLIM1 |  |  | 452 | 0.280 | -0.0347 | No |
| 10 | INPP5F |  |  | 469 | 0.276 | -0.0167 | No |
| 11 | ABAT |  |  | 617 | -0.259 | -0.1359 | No |
| 12 | ELOVL2 |  |  | 735 | -0.329 | -0.2154 | Yes |
| 13 | HES1 |  |  | 736 | -0.331 | -0.1738 | Yes |
| 14 | GJA1 |  |  | 823 | -0.405 | -0.2117 | Yes |
| 15 | CHPT1 |  |  | 844 | -0.437 | -0.1775 | Yes |
| 16 | PRSS23 |  |  | 874 | -0.486 | -0.1465 | Yes |
| 17 | RAB31 |  |  | 916 | -0.586 | -0.1153 | Yes |
| 18 | PODXL |  |  | 928 | -0.636 | -0.0469 | Yes |
| 19 | CD44 |  |  | 957 | -0.851 | 0.0310 | Yes |
Table: GSEA details [plain text format]

  

Fig 2: HALLMARK\_ESTROGEN\_RESPONSE\_EARLY: Random ES distribution      
 Gene set null distribution of ES for **HALLMARK\_ESTROGEN\_RESPONSE\_EARLY**

  
