# Supplementary material for: Modeling glioblastoma heterogeneity as a dynamic network of cell states
Source: Mol Syst Biol. 2021 Sep 16;17(9):e10105. doi: 10.15252/msb.202010105 (PMC8444284; doi:10.15252/msb.202010105)
Supplement: Supplementary file 6 — Source Data for Figure 5 [file MSB-17-e10105-s004.zip › Figure5A_sourcedata/GSEA_3017/hallmarks_stateA.GseaPreranked.1621934654007/HALLMARK_ESTROGEN_RESPONSE_LATE.html]

Details for gene set HALLMARK\_ESTROGEN\_RESPONSE\_LATE[GSEA]

|  || Dataset | state53017 |
| Phenotype | NoPhenotypeAvailable |
| Upregulated in class | na\_pos |
| GeneSet | HALLMARK\_ESTROGEN\_RESPONSE\_LATE |
| Enrichment Score (ES) | 0.21782926 |
| Normalized Enrichment Score (NES) | 1.0678316 |
| Nominal p-value | 0.3517588 |
| FDR q-value | 0.36822182 |
| FWER p-Value | 0.948 |
Table: GSEA Results Summary

  

Fig 1: Enrichment plot: HALLMARK\_ESTROGEN\_RESPONSE\_LATE      
 Profile of the Running ES Score & Positions of GeneSet Members on the Rank Ordered List

  

| PROBE | GENE SYMBOL | GENE\_TITLE | RANK IN GENE LIST | RANK METRIC SCORE | RUNNING ES | CORE ENRICHMENT || 1 | FABP5 |  |  | 0 | 1.759 | 0.1559 | Yes |
| 2 | CA12 |  |  | 24 | 0.645 | 0.1892 | Yes |
| 3 | CDC20 |  |  | 80 | 0.472 | 0.1739 | Yes |
| 4 | TOP2A |  |  | 95 | 0.457 | 0.1999 | Yes |
| 5 | SLC26A2 |  |  | 121 | 0.428 | 0.2119 | Yes |
| 6 | DNAJC1 |  |  | 151 | 0.407 | 0.2178 | Yes |
| 7 | UGDH |  |  | 224 | 0.363 | 0.1753 | No |
| 8 | PPIF |  |  | 248 | 0.350 | 0.1824 | No |
| 9 | SORD |  |  | 317 | 0.320 | 0.1401 | No |
| 10 | ARL3 |  |  | 318 | 0.319 | 0.1685 | No |
| 11 | FARP1 |  |  | 332 | 0.315 | 0.1829 | No |
| 12 | FKBP5 |  |  | 368 | 0.304 | 0.1735 | No |
| 13 | CHST8 |  |  | 375 | 0.301 | 0.1940 | No |
| 14 | CDC6 |  |  | 391 | 0.297 | 0.2047 | No |
| 15 | KIF20A |  |  | 447 | 0.281 | 0.1726 | No |
| 16 | STIL |  |  | 545 | 0.261 | 0.0950 | No |
| 17 | CKB |  |  | 676 | -0.287 | -0.0146 | No |
| 18 | PRKAR2B |  |  | 726 | -0.323 | -0.0369 | No |
| 19 | ID2 |  |  | 727 | -0.323 | -0.0083 | No |
| 20 | TSPAN13 |  |  | 760 | -0.349 | -0.0105 | No |
| 21 | ALDH3A2 |  |  | 767 | -0.359 | 0.0150 | No |
| 22 | CHPT1 |  |  | 844 | -0.437 | -0.0251 | No |
| 23 | PRSS23 |  |  | 874 | -0.486 | -0.0122 | No |
| 24 | RAB31 |  |  | 916 | -0.586 | -0.0028 | No |
| 25 | CD44 |  |  | 957 | -0.851 | 0.0312 | No |
Table: GSEA details [plain text format]

  

Fig 2: HALLMARK\_ESTROGEN\_RESPONSE\_LATE: Random ES distribution      
 Gene set null distribution of ES for **HALLMARK\_ESTROGEN\_RESPONSE\_LATE**

  
