# Supplementary material for: Modeling glioblastoma heterogeneity as a dynamic network of cell states
Source: Mol Syst Biol. 2021 Sep 16;17(9):e10105. doi: 10.15252/msb.202010105 (PMC8444284; doi:10.15252/msb.202010105)
Supplement: Supplementary file 6 — Source Data for Figure 5 [file MSB-17-e10105-s004.zip › Figure5A_sourcedata/GSEA_3017/hallmarks_stateA.GseaPreranked.1621934654007/HALLMARK_G2M_CHECKPOINT.html]

Details for gene set HALLMARK\_G2M\_CHECKPOINT[GSEA]

|  || Dataset | state53017 |
| Phenotype | NoPhenotypeAvailable |
| Upregulated in class | na\_pos |
| GeneSet | HALLMARK\_G2M\_CHECKPOINT |
| Enrichment Score (ES) | 0.48520577 |
| Normalized Enrichment Score (NES) | 3.1930492 |
| Nominal p-value | 0.0 |
| FDR q-value | 0.0 |
| FWER p-Value | 0.0 |
Table: GSEA Results Summary

  

Fig 1: Enrichment plot: HALLMARK\_G2M\_CHECKPOINT      
 Profile of the Running ES Score & Positions of GeneSet Members on the Rank Ordered List

  

| PROBE | GENE SYMBOL | GENE\_TITLE | RANK IN GENE LIST | RANK METRIC SCORE | RUNNING ES | CORE ENRICHMENT || 1 | CDK1 |  |  | 3 | 1.006 | 0.0352 | Yes |
| 2 | MKI67 |  |  | 4 | 0.926 | 0.0705 | Yes |
| 3 | KIF11 |  |  | 6 | 0.870 | 0.1026 | Yes |
| 4 | UBE2C |  |  | 7 | 0.793 | 0.1329 | Yes |
| 5 | KIF20B |  |  | 10 | 0.749 | 0.1593 | Yes |
| 6 | BUB3 |  |  | 12 | 0.740 | 0.1864 | Yes |
| 7 | KIF5B |  |  | 16 | 0.687 | 0.2094 | Yes |
| 8 | KPNA2 |  |  | 20 | 0.667 | 0.2317 | Yes |
| 9 | CKS2 |  |  | 40 | 0.587 | 0.2335 | Yes |
| 10 | HMMR |  |  | 43 | 0.559 | 0.2527 | Yes |
| 11 | CKS1B |  |  | 50 | 0.530 | 0.2665 | Yes |
| 12 | DBF4 |  |  | 55 | 0.514 | 0.2818 | Yes |
| 13 | CENPE |  |  | 62 | 0.496 | 0.2942 | Yes |
| 14 | CCNA2 |  |  | 65 | 0.495 | 0.3109 | Yes |
| 15 | TPX2 |  |  | 66 | 0.488 | 0.3296 | Yes |
| 16 | MYBL2 |  |  | 69 | 0.486 | 0.3459 | Yes |
| 17 | AURKA |  |  | 78 | 0.474 | 0.3554 | Yes |
| 18 | CDC20 |  |  | 80 | 0.472 | 0.3723 | Yes |
| 19 | PLK1 |  |  | 89 | 0.464 | 0.3814 | Yes |
| 20 | CENPF |  |  | 94 | 0.461 | 0.3947 | Yes |
| 21 | TOP2A |  |  | 95 | 0.457 | 0.4121 | Yes |
| 22 | AURKB |  |  | 108 | 0.446 | 0.4162 | Yes |
| 23 | KIF23 |  |  | 116 | 0.436 | 0.4253 | Yes |
| 24 | JPT1 |  |  | 125 | 0.426 | 0.4329 | Yes |
| 25 | KNL1 |  |  | 133 | 0.418 | 0.4413 | Yes |
| 26 | CCNB2 |  |  | 147 | 0.411 | 0.4429 | Yes |
| 27 | MAD2L1 |  |  | 153 | 0.406 | 0.4530 | Yes |
| 28 | KIF2C |  |  | 165 | 0.397 | 0.4563 | Yes |
| 29 | MYC |  |  | 169 | 0.395 | 0.4682 | Yes |
| 30 | NDC80 |  |  | 173 | 0.393 | 0.4799 | Yes |
| 31 | BUB1 |  |  | 188 | 0.384 | 0.4795 | Yes |
| 32 | KIF22 |  |  | 197 | 0.377 | 0.4852 | Yes |
| 33 | SMC4 |  |  | 217 | 0.367 | 0.4787 | No |
| 34 | INCENP |  |  | 227 | 0.362 | 0.4828 | No |
| 35 | CENPA |  |  | 243 | 0.352 | 0.4800 | No |
| 36 | TTK |  |  | 262 | 0.340 | 0.4736 | No |
| 37 | ORC6 |  |  | 283 | 0.333 | 0.4647 | No |
| 38 | BRCA2 |  |  | 308 | 0.323 | 0.4511 | No |
| 39 | PRC1 |  |  | 350 | 0.310 | 0.4186 | No |
| 40 | CCNF |  |  | 366 | 0.305 | 0.4141 | No |
| 41 | CDC6 |  |  | 391 | 0.297 | 0.3995 | No |
| 42 | HMGA1 |  |  | 397 | 0.295 | 0.4053 | No |
| 43 | RACGAP1 |  |  | 399 | 0.293 | 0.4154 | No |
| 44 | NOLC1 |  |  | 425 | 0.288 | 0.3994 | No |
| 45 | NUSAP1 |  |  | 430 | 0.286 | 0.4060 | No |
| 46 | LMNB1 |  |  | 442 | 0.282 | 0.4049 | No |
| 47 | CDC45 |  |  | 446 | 0.282 | 0.4124 | No |
| 48 | BCL3 |  |  | 455 | 0.280 | 0.4145 | No |
| 49 | ODC1 |  |  | 460 | 0.279 | 0.4208 | No |
| 50 | EZH2 |  |  | 471 | 0.275 | 0.4205 | No |
| 51 | MT2A |  |  | 473 | 0.275 | 0.4299 | No |
| 52 | KIF4A |  |  | 504 | 0.269 | 0.4077 | No |
| 53 | UBE2S |  |  | 507 | 0.269 | 0.4159 | No |
| 54 | UCK2 |  |  | 508 | 0.269 | 0.4261 | No |
| 55 | KIF15 |  |  | 538 | 0.263 | 0.4048 | No |
| 56 | CDKN3 |  |  | 539 | 0.263 | 0.4149 | No |
| 57 | SRSF2 |  |  | 542 | 0.262 | 0.4227 | No |
| 58 | STIL |  |  | 545 | 0.261 | 0.4305 | No |
| 59 | FBXO5 |  |  | 557 | 0.258 | 0.4285 | No |
| 60 | DDX39A |  |  | 562 | 0.258 | 0.4340 | No |
| 61 | TMPO |  |  | 584 | 0.253 | 0.4210 | No |
| 62 | MEIS2 |  |  | 754 | -0.345 | 0.2516 | No |
Table: GSEA details [plain text format]

  

Fig 2: HALLMARK\_G2M\_CHECKPOINT: Random ES distribution      
 Gene set null distribution of ES for **HALLMARK\_G2M\_CHECKPOINT**

  
