# Supplementary material for: Modeling glioblastoma heterogeneity as a dynamic network of cell states
Source: Mol Syst Biol. 2021 Sep 16;17(9):e10105. doi: 10.15252/msb.202010105 (PMC8444284; doi:10.15252/msb.202010105)
Supplement: Supplementary file 6 — Source Data for Figure 5 [file MSB-17-e10105-s004.zip › Figure5A_sourcedata/GSEA_3017/hallmarks_stateA.GseaPreranked.1621934654007/HALLMARK_GLYCOLYSIS.html]

Details for gene set HALLMARK\_GLYCOLYSIS[GSEA]

|  || Dataset | state53017 |
| Phenotype | NoPhenotypeAvailable |
| Upregulated in class | na\_pos |
| GeneSet | HALLMARK\_GLYCOLYSIS |
| Enrichment Score (ES) | 0.2749477 |
| Normalized Enrichment Score (NES) | 1.3955998 |
| Nominal p-value | 0.11702128 |
| FDR q-value | 0.12546822 |
| FWER p-Value | 0.571 |
Table: GSEA Results Summary

  

Fig 1: Enrichment plot: HALLMARK\_GLYCOLYSIS      
 Profile of the Running ES Score & Positions of GeneSet Members on the Rank Ordered List

  

| PROBE | GENE SYMBOL | GENE\_TITLE | RANK IN GENE LIST | RANK METRIC SCORE | RUNNING ES | CORE ENRICHMENT || 1 | CDK1 |  |  | 3 | 1.006 | 0.0770 | Yes |
| 2 | MIF |  |  | 13 | 0.738 | 0.1263 | Yes |
| 3 | IGFBP3 |  |  | 26 | 0.641 | 0.1649 | Yes |
| 4 | PGAM1 |  |  | 35 | 0.608 | 0.2049 | Yes |
| 5 | HMMR |  |  | 43 | 0.559 | 0.2421 | Yes |
| 6 | AURKA |  |  | 78 | 0.474 | 0.2444 | Yes |
| 7 | STC1 |  |  | 92 | 0.463 | 0.2677 | Yes |
| 8 | DEPDC1 |  |  | 119 | 0.432 | 0.2749 | Yes |
| 9 | CENPA |  |  | 243 | 0.352 | 0.1747 | No |
| 10 | HOMER1 |  |  | 255 | 0.346 | 0.1908 | No |
| 11 | POLR3K |  |  | 351 | 0.309 | 0.1164 | No |
| 12 | P4HA1 |  |  | 411 | 0.290 | 0.0779 | No |
| 13 | NT5E |  |  | 445 | 0.282 | 0.0660 | No |
| 14 | KIF20A |  |  | 447 | 0.281 | 0.0873 | No |
| 15 | PFKP |  |  | 454 | 0.280 | 0.1034 | No |
| 16 | VEGFA |  |  | 546 | 0.261 | 0.0292 | No |
| 17 | SOX9 |  |  | 577 | 0.254 | 0.0182 | No |
| 18 | DPYSL4 |  |  | 581 | 0.253 | 0.0352 | No |
| 19 | SDC3 |  |  | 637 | -0.264 | -0.0011 | No |
| 20 | CHST2 |  |  | 652 | -0.271 | 0.0058 | No |
| 21 | GPC4 |  |  | 687 | -0.295 | -0.0061 | No |
| 22 | IRS2 |  |  | 781 | -0.368 | -0.0738 | No |
| 23 | CHPF |  |  | 793 | -0.375 | -0.0554 | No |
| 24 | GPC1 |  |  | 801 | -0.384 | -0.0321 | No |
| 25 | PAM |  |  | 832 | -0.422 | -0.0298 | No |
| 26 | PLOD2 |  |  | 838 | -0.431 | -0.0008 | No |
| 27 | VLDLR |  |  | 879 | -0.503 | -0.0024 | No |
| 28 | SDC2 |  |  | 908 | -0.568 | 0.0136 | No |
| 29 | CD44 |  |  | 957 | -0.851 | 0.0313 | No |
Table: GSEA details [plain text format]

  

Fig 2: HALLMARK\_GLYCOLYSIS: Random ES distribution      
 Gene set null distribution of ES for **HALLMARK\_GLYCOLYSIS**

  
