# Supplementary material for: Modeling glioblastoma heterogeneity as a dynamic network of cell states
Source: Mol Syst Biol. 2021 Sep 16;17(9):e10105. doi: 10.15252/msb.202010105 (PMC8444284; doi:10.15252/msb.202010105)
Supplement: Supplementary file 6 — Source Data for Figure 5 [file MSB-17-e10105-s004.zip › Figure5A_sourcedata/GSEA_3017/hallmarks_stateA.GseaPreranked.1621934654007/HALLMARK_HYPOXIA.html]

Details for gene set HALLMARK\_HYPOXIA[GSEA]

|  || Dataset | state53017 |
| Phenotype | NoPhenotypeAvailable |
| Upregulated in class | na\_neg |
| GeneSet | HALLMARK\_HYPOXIA |
| Enrichment Score (ES) | -0.32738045 |
| Normalized Enrichment Score (NES) | -1.5874249 |
| Nominal p-value | 0.035603717 |
| FDR q-value | 0.08044287 |
| FWER p-Value | 0.461 |
Table: GSEA Results Summary

  

Fig 1: Enrichment plot: HALLMARK\_HYPOXIA      
 Profile of the Running ES Score & Positions of GeneSet Members on the Rank Ordered List

  

| PROBE | GENE SYMBOL | GENE\_TITLE | RANK IN GENE LIST | RANK METRIC SCORE | RUNNING ES | CORE ENRICHMENT || 1 | MIF |  |  | 13 | 0.738 | 0.0343 | No |
| 2 | CA12 |  |  | 24 | 0.645 | 0.0657 | No |
| 3 | IGFBP3 |  |  | 26 | 0.641 | 0.1063 | No |
| 4 | STC1 |  |  | 92 | 0.463 | 0.0682 | No |
| 5 | S100A4 |  |  | 148 | 0.409 | 0.0371 | No |
| 6 | MAP3K1 |  |  | 167 | 0.396 | 0.0439 | No |
| 7 | NFIL3 |  |  | 204 | 0.371 | 0.0303 | No |
| 8 | BCAN |  |  | 276 | 0.334 | -0.0225 | No |
| 9 | P4HA1 |  |  | 411 | 0.290 | -0.1443 | No |
| 10 | PFKP |  |  | 454 | 0.280 | -0.1702 | No |
| 11 | MT2A |  |  | 473 | 0.275 | -0.1713 | No |
| 12 | VEGFA |  |  | 546 | 0.261 | -0.2299 | No |
| 13 | TPD52 |  |  | 566 | 0.257 | -0.2332 | No |
| 14 | DPYSL4 |  |  | 581 | 0.253 | -0.2314 | No |
| 15 | NR3C1 |  |  | 610 | -0.256 | -0.2441 | No |
| 16 | SDC3 |  |  | 637 | -0.264 | -0.2543 | No |
| 17 | CHST2 |  |  | 652 | -0.271 | -0.2514 | No |
| 18 | GPC4 |  |  | 687 | -0.295 | -0.2679 | No |
| 19 | AKAP12 |  |  | 690 | -0.296 | -0.2508 | No |
| 20 | NEDD4L |  |  | 764 | -0.355 | -0.3043 | Yes |
| 21 | IRS2 |  |  | 781 | -0.368 | -0.2972 | Yes |
| 22 | PNRC1 |  |  | 786 | -0.371 | -0.2773 | Yes |
| 23 | GPC1 |  |  | 801 | -0.384 | -0.2670 | Yes |
| 24 | PDGFB |  |  | 816 | -0.399 | -0.2558 | Yes |
| 25 | PAM |  |  | 832 | -0.422 | -0.2442 | Yes |
| 26 | ATF3 |  |  | 836 | -0.428 | -0.2195 | Yes |
| 27 | BNIP3L |  |  | 841 | -0.432 | -0.1956 | Yes |
| 28 | ANXA2 |  |  | 872 | -0.482 | -0.1958 | Yes |
| 29 | VLDLR |  |  | 879 | -0.503 | -0.1694 | Yes |
| 30 | JUN |  |  | 886 | -0.525 | -0.1416 | Yes |
| 31 | F3 |  |  | 888 | -0.526 | -0.1085 | Yes |
| 32 | SDC2 |  |  | 908 | -0.568 | -0.0916 | Yes |
| 33 | SERPINE1 |  |  | 951 | -0.774 | -0.0854 | Yes |
| 34 | CSRP2 |  |  | 960 | -0.877 | -0.0368 | Yes |
| 35 | CDKN1A |  |  | 969 | -0.987 | 0.0189 | Yes |
Table: GSEA details [plain text format]

  

Fig 2: HALLMARK\_HYPOXIA: Random ES distribution      
 Gene set null distribution of ES for **HALLMARK\_HYPOXIA**

  
