# Supplementary material for: Modeling glioblastoma heterogeneity as a dynamic network of cell states
Source: Mol Syst Biol. 2021 Sep 16;17(9):e10105. doi: 10.15252/msb.202010105 (PMC8444284; doi:10.15252/msb.202010105)
Supplement: Supplementary file 6 — Source Data for Figure 5 [file MSB-17-e10105-s004.zip › Figure5A_sourcedata/GSEA_3017/hallmarks_stateA.GseaPreranked.1621934654007/HALLMARK_IL2_STAT5_SIGNALING.html]

Details for gene set HALLMARK\_IL2\_STAT5\_SIGNALING[GSEA]

|  || Dataset | state53017 |
| Phenotype | NoPhenotypeAvailable |
| Upregulated in class | na\_neg |
| GeneSet | HALLMARK\_IL2\_STAT5\_SIGNALING |
| Enrichment Score (ES) | -0.2928404 |
| Normalized Enrichment Score (NES) | -1.1527234 |
| Nominal p-value | 0.29623288 |
| FDR q-value | 0.4114083 |
| FWER p-Value | 0.975 |
Table: GSEA Results Summary

  

Fig 1: Enrichment plot: HALLMARK\_IL2\_STAT5\_SIGNALING      
 Profile of the Running ES Score & Positions of GeneSet Members on the Rank Ordered List

  

| PROBE | GENE SYMBOL | GENE\_TITLE | RANK IN GENE LIST | RANK METRIC SCORE | RUNNING ES | CORE ENRICHMENT || 1 | GSTO1 |  |  | 15 | 0.718 | 0.0689 | No |
| 2 | MYC |  |  | 169 | 0.395 | -0.0427 | No |
| 3 | NFIL3 |  |  | 204 | 0.371 | -0.0342 | No |
| 4 | ALCAM |  |  | 252 | 0.348 | -0.0418 | No |
| 5 | SWAP70 |  |  | 323 | 0.317 | -0.0768 | No |
| 6 | CDC6 |  |  | 391 | 0.297 | -0.1111 | No |
| 7 | P4HA1 |  |  | 411 | 0.290 | -0.0967 | No |
| 8 | NT5E |  |  | 445 | 0.282 | -0.0976 | No |
| 9 | ODC1 |  |  | 460 | 0.279 | -0.0793 | No |
| 10 | UCK2 |  |  | 508 | 0.269 | -0.0963 | No |
| 11 | ITGA6 |  |  | 544 | 0.261 | -0.1017 | No |
| 12 | ITGAV |  |  | 599 | -0.250 | -0.1281 | No |
| 13 | RGS16 |  |  | 620 | -0.260 | -0.1182 | No |
| 14 | EMP1 |  |  | 724 | -0.322 | -0.1868 | No |
| 15 | HOPX |  |  | 821 | -0.403 | -0.2385 | Yes |
| 16 | SERPINB6 |  |  | 861 | -0.452 | -0.2257 | Yes |
| 17 | SPP1 |  |  | 927 | -0.630 | -0.2187 | Yes |
| 18 | APLP1 |  |  | 935 | -0.680 | -0.1460 | Yes |
| 19 | IFITM3 |  |  | 954 | -0.830 | -0.0670 | Yes |
| 20 | CD44 |  |  | 957 | -0.851 | 0.0310 | Yes |
Table: GSEA details [plain text format]

  

Fig 2: HALLMARK\_IL2\_STAT5\_SIGNALING: Random ES distribution      
 Gene set null distribution of ES for **HALLMARK\_IL2\_STAT5\_SIGNALING**

  
