# Supplementary material for: Modeling glioblastoma heterogeneity as a dynamic network of cell states
Source: Mol Syst Biol. 2021 Sep 16;17(9):e10105. doi: 10.15252/msb.202010105 (PMC8444284; doi:10.15252/msb.202010105)
Supplement: Supplementary file 6 — Source Data for Figure 5 [file MSB-17-e10105-s004.zip › Figure5A_sourcedata/GSEA_3017/hallmarks_stateA.GseaPreranked.1621934654007/HALLMARK_INFLAMMATORY_RESPONSE.html]

Details for gene set HALLMARK\_INFLAMMATORY\_RESPONSE[GSEA]

|  || Dataset | state53017 |
| Phenotype | NoPhenotypeAvailable |
| Upregulated in class | na\_neg |
| GeneSet | HALLMARK\_INFLAMMATORY\_RESPONSE |
| Enrichment Score (ES) | -0.44386017 |
| Normalized Enrichment Score (NES) | -1.7660167 |
| Nominal p-value | 0.013559322 |
| FDR q-value | 0.035304807 |
| FWER p-Value | 0.174 |
Table: GSEA Results Summary

  

Fig 1: Enrichment plot: HALLMARK\_INFLAMMATORY\_RESPONSE      
 Profile of the Running ES Score & Positions of GeneSet Members on the Rank Ordered List

  

| PROBE | GENE SYMBOL | GENE\_TITLE | RANK IN GENE LIST | RANK METRIC SCORE | RUNNING ES | CORE ENRICHMENT || 1 | HAS2 |  |  | 45 | 0.551 | 0.0070 | No |
| 2 | TNFAIP6 |  |  | 99 | 0.455 | -0.0035 | No |
| 3 | EMP3 |  |  | 101 | 0.452 | 0.0394 | No |
| 4 | MYC |  |  | 169 | 0.395 | 0.0085 | No |
| 5 | ABI1 |  |  | 459 | 0.279 | -0.2629 | No |
| 6 | CD82 |  |  | 597 | 0.250 | -0.3802 | No |
| 7 | BST2 |  |  | 614 | -0.259 | -0.3716 | No |
| 8 | RGS16 |  |  | 620 | -0.260 | -0.3516 | No |
| 9 | ABCA1 |  |  | 647 | -0.268 | -0.3524 | No |
| 10 | CHST2 |  |  | 652 | -0.271 | -0.3302 | No |
| 11 | SLC4A4 |  |  | 763 | -0.355 | -0.4094 | Yes |
| 12 | ITGB8 |  |  | 774 | -0.363 | -0.3844 | Yes |
| 13 | DCBLD2 |  |  | 791 | -0.375 | -0.3646 | Yes |
| 14 | SLC1A2 |  |  | 860 | -0.450 | -0.3911 | Yes |
| 15 | F3 |  |  | 888 | -0.526 | -0.3679 | Yes |
| 16 | PDPN |  |  | 890 | -0.529 | -0.3176 | Yes |
| 17 | KCNJ2 |  |  | 921 | -0.618 | -0.2886 | Yes |
| 18 | SERPINE1 |  |  | 951 | -0.774 | -0.2434 | Yes |
| 19 | CDKN1A |  |  | 969 | -0.987 | -0.1651 | Yes |
| 20 | CCL2 |  |  | 985 | -1.881 | 0.0021 | Yes |
Table: GSEA details [plain text format]

  

Fig 2: HALLMARK\_INFLAMMATORY\_RESPONSE: Random ES distribution      
 Gene set null distribution of ES for **HALLMARK\_INFLAMMATORY\_RESPONSE**

  
