# Supplementary material for: Modeling glioblastoma heterogeneity as a dynamic network of cell states
Source: Mol Syst Biol. 2021 Sep 16;17(9):e10105. doi: 10.15252/msb.202010105 (PMC8444284; doi:10.15252/msb.202010105)
Supplement: Supplementary file 6 — Source Data for Figure 5 [file MSB-17-e10105-s004.zip › Figure5A_sourcedata/GSEA_3017/hallmarks_stateA.GseaPreranked.1621934654007/HALLMARK_KRAS_SIGNALING_UP.html]

Details for gene set HALLMARK\_KRAS\_SIGNALING\_UP[GSEA]

|  || Dataset | state53017 |
| Phenotype | NoPhenotypeAvailable |
| Upregulated in class | na\_neg |
| GeneSet | HALLMARK\_KRAS\_SIGNALING\_UP |
| Enrichment Score (ES) | -0.31402898 |
| Normalized Enrichment Score (NES) | -1.2576221 |
| Nominal p-value | 0.19621342 |
| FDR q-value | 0.32224223 |
| FWER p-Value | 0.94 |
Table: GSEA Results Summary

  

Fig 1: Enrichment plot: HALLMARK\_KRAS\_SIGNALING\_UP      
 Profile of the Running ES Score & Positions of GeneSet Members on the Rank Ordered List

  

| PROBE | GENE SYMBOL | GENE\_TITLE | RANK IN GENE LIST | RANK METRIC SCORE | RUNNING ES | CORE ENRICHMENT || 1 | IGFBP3 |  |  | 26 | 0.641 | 0.0516 | No |
| 2 | MAP3K1 |  |  | 167 | 0.396 | -0.0444 | No |
| 3 | CCSER2 |  |  | 331 | 0.315 | -0.1741 | No |
| 4 | PEG3 |  |  | 335 | 0.314 | -0.1388 | No |
| 5 | PLAU |  |  | 347 | 0.310 | -0.1122 | No |
| 6 | AMMECR1 |  |  | 481 | 0.274 | -0.2160 | No |
| 7 | SOX9 |  |  | 577 | 0.254 | -0.2829 | Yes |
| 8 | PRDM1 |  |  | 601 | -0.252 | -0.2759 | Yes |
| 9 | RGS16 |  |  | 620 | -0.260 | -0.2627 | Yes |
| 10 | SPRY2 |  |  | 669 | -0.282 | -0.2778 | Yes |
| 11 | AKAP12 |  |  | 690 | -0.296 | -0.2623 | Yes |
| 12 | EMP1 |  |  | 724 | -0.322 | -0.2570 | Yes |
| 13 | ID2 |  |  | 727 | -0.323 | -0.2196 | Yes |
| 14 | TSPAN13 |  |  | 760 | -0.349 | -0.2099 | Yes |
| 15 | PSMB8 |  |  | 772 | -0.362 | -0.1770 | Yes |
| 16 | DCBLD2 |  |  | 791 | -0.375 | -0.1497 | Yes |
| 17 | PRRX1 |  |  | 829 | -0.413 | -0.1374 | Yes |
| 18 | SPP1 |  |  | 927 | -0.630 | -0.1604 | Yes |
| 19 | SPARCL1 |  |  | 984 | -1.810 | 0.0031 | Yes |
Table: GSEA details [plain text format]

  

Fig 2: HALLMARK\_KRAS\_SIGNALING\_UP: Random ES distribution      
 Gene set null distribution of ES for **HALLMARK\_KRAS\_SIGNALING\_UP**

  
