# Supplementary material for: Modeling glioblastoma heterogeneity as a dynamic network of cell states
Source: Mol Syst Biol. 2021 Sep 16;17(9):e10105. doi: 10.15252/msb.202010105 (PMC8444284; doi:10.15252/msb.202010105)
Supplement: Supplementary file 6 — Source Data for Figure 5 [file MSB-17-e10105-s004.zip › Figure5A_sourcedata/GSEA_3017/hallmarks_stateA.GseaPreranked.1621934654007/HALLMARK_MITOTIC_SPINDLE.html]

Details for gene set HALLMARK\_MITOTIC\_SPINDLE[GSEA]

|  || Dataset | state53017 |
| Phenotype | NoPhenotypeAvailable |
| Upregulated in class | na\_pos |
| GeneSet | HALLMARK\_MITOTIC\_SPINDLE |
| Enrichment Score (ES) | 0.3889335 |
| Normalized Enrichment Score (NES) | 2.4275417 |
| Nominal p-value | 0.0 |
| FDR q-value | 0.0 |
| FWER p-Value | 0.0 |
Table: GSEA Results Summary

  

Fig 1: Enrichment plot: HALLMARK\_MITOTIC\_SPINDLE      
 Profile of the Running ES Score & Positions of GeneSet Members on the Rank Ordered List

  

| PROBE | GENE SYMBOL | GENE\_TITLE | RANK IN GENE LIST | RANK METRIC SCORE | RUNNING ES | CORE ENRICHMENT || 1 | CDK1 |  |  | 3 | 1.006 | 0.0467 | Yes |
| 2 | KIF11 |  |  | 6 | 0.870 | 0.0878 | Yes |
| 3 | KIF20B |  |  | 10 | 0.749 | 0.1217 | Yes |
| 4 | KIF5B |  |  | 16 | 0.687 | 0.1505 | Yes |
| 5 | PIF1 |  |  | 30 | 0.627 | 0.1678 | Yes |
| 6 | SMC3 |  |  | 58 | 0.504 | 0.1641 | Yes |
| 7 | CENPE |  |  | 62 | 0.496 | 0.1855 | Yes |
| 8 | TPX2 |  |  | 66 | 0.488 | 0.2065 | Yes |
| 9 | DLGAP5 |  |  | 76 | 0.475 | 0.2205 | Yes |
| 10 | AURKA |  |  | 78 | 0.474 | 0.2429 | Yes |
| 11 | PLK1 |  |  | 89 | 0.464 | 0.2553 | Yes |
| 12 | CENPF |  |  | 94 | 0.461 | 0.2739 | Yes |
| 13 | TOP2A |  |  | 95 | 0.457 | 0.2966 | Yes |
| 14 | KIF23 |  |  | 116 | 0.436 | 0.2970 | Yes |
| 15 | CCNB2 |  |  | 147 | 0.411 | 0.2855 | Yes |
| 16 | ANLN |  |  | 152 | 0.407 | 0.3014 | Yes |
| 17 | KIF2C |  |  | 165 | 0.397 | 0.3083 | Yes |
| 18 | NDC80 |  |  | 173 | 0.393 | 0.3204 | Yes |
| 19 | BUB1 |  |  | 188 | 0.384 | 0.3246 | Yes |
| 20 | TUBGCP2 |  |  | 196 | 0.378 | 0.3359 | Yes |
| 21 | KIF22 |  |  | 197 | 0.377 | 0.3546 | Yes |
| 22 | EPB41L2 |  |  | 208 | 0.371 | 0.3623 | Yes |
| 23 | SMC4 |  |  | 217 | 0.367 | 0.3720 | Yes |
| 24 | INCENP |  |  | 227 | 0.362 | 0.3804 | Yes |
| 25 | TRIO |  |  | 246 | 0.351 | 0.3786 | Yes |
| 26 | TTK |  |  | 262 | 0.340 | 0.3796 | Yes |
| 27 | CENPJ |  |  | 270 | 0.339 | 0.3889 | Yes |
| 28 | BRCA2 |  |  | 308 | 0.323 | 0.3656 | No |
| 29 | FARP1 |  |  | 332 | 0.315 | 0.3568 | No |
| 30 | PRC1 |  |  | 350 | 0.310 | 0.3540 | No |
| 31 | ECT2 |  |  | 365 | 0.305 | 0.3543 | No |
| 32 | NET1 |  |  | 378 | 0.301 | 0.3564 | No |
| 33 | RACGAP1 |  |  | 399 | 0.293 | 0.3497 | No |
| 34 | NUSAP1 |  |  | 430 | 0.286 | 0.3320 | No |
| 35 | LMNB1 |  |  | 442 | 0.282 | 0.3343 | No |
| 36 | ABI1 |  |  | 459 | 0.279 | 0.3311 | No |
| 37 | KIF4A |  |  | 504 | 0.269 | 0.2976 | No |
| 38 | KIF15 |  |  | 538 | 0.263 | 0.2756 | No |
| 39 | FBXO5 |  |  | 557 | 0.258 | 0.2692 | No |
| 40 | DLG1 |  |  | 611 | -0.257 | 0.2256 | No |
| 41 | FLNA |  |  | 636 | -0.264 | 0.2132 | No |
| 42 | KIFAP3 |  |  | 656 | -0.272 | 0.2065 | No |
| 43 | SPTBN1 |  |  | 725 | -0.322 | 0.1501 | No |
| 44 | DST |  |  | 818 | -0.400 | 0.0721 | No |
| 45 | APC |  |  | 856 | -0.447 | 0.0549 | No |
| 46 | EZR |  |  | 865 | -0.464 | 0.0694 | No |
| 47 | ARHGAP29 |  |  | 899 | -0.548 | 0.0615 | No |
| 48 | NEDD9 |  |  | 923 | -0.625 | 0.0681 | No |
Table: GSEA details [plain text format]

  

Fig 2: HALLMARK\_MITOTIC\_SPINDLE: Random ES distribution      
 Gene set null distribution of ES for **HALLMARK\_MITOTIC\_SPINDLE**

  
