# Supplementary material for: Modeling glioblastoma heterogeneity as a dynamic network of cell states
Source: Mol Syst Biol. 2021 Sep 16;17(9):e10105. doi: 10.15252/msb.202010105 (PMC8444284; doi:10.15252/msb.202010105)
Supplement: Supplementary file 6 — Source Data for Figure 5 [file MSB-17-e10105-s004.zip › Figure5A_sourcedata/GSEA_3017/hallmarks_stateA.GseaPreranked.1621934654007/HALLMARK_MTORC1_SIGNALING.html]

Details for gene set HALLMARK\_MTORC1\_SIGNALING[GSEA]

|  || Dataset | state53017 |
| Phenotype | NoPhenotypeAvailable |
| Upregulated in class | na\_neg |
| GeneSet | HALLMARK\_MTORC1\_SIGNALING |
| Enrichment Score (ES) | -0.18379016 |
| Normalized Enrichment Score (NES) | -0.82704175 |
| Nominal p-value | 0.63917524 |
| FDR q-value | 0.72593707 |
| FWER p-Value | 1.0 |
Table: GSEA Results Summary

  

Fig 1: Enrichment plot: HALLMARK\_MTORC1\_SIGNALING      
 Profile of the Running ES Score & Positions of GeneSet Members on the Rank Ordered List

  

| PROBE | GENE SYMBOL | GENE\_TITLE | RANK IN GENE LIST | RANK METRIC SCORE | RUNNING ES | CORE ENRICHMENT || 1 | PPA1 |  |  | 19 | 0.668 | 0.0323 | Yes |
| 2 | AURKA |  |  | 78 | 0.474 | 0.0087 | Yes |
| 3 | PLK1 |  |  | 89 | 0.464 | 0.0345 | Yes |
| 4 | STC1 |  |  | 92 | 0.463 | 0.0686 | Yes |
| 5 | CTSC |  |  | 96 | 0.456 | 0.1011 | Yes |
| 6 | BUB1 |  |  | 188 | 0.384 | 0.0361 | Yes |
| 7 | ELOVL6 |  |  | 189 | 0.383 | 0.0659 | Yes |
| 8 | BCAT1 |  |  | 194 | 0.380 | 0.0914 | Yes |
| 9 | NFIL3 |  |  | 204 | 0.371 | 0.1110 | Yes |
| 10 | SDF2L1 |  |  | 257 | 0.345 | 0.0837 | Yes |
| 11 | PSMB5 |  |  | 280 | 0.334 | 0.0867 | Yes |
| 12 | MTHFD2 |  |  | 287 | 0.330 | 0.1062 | Yes |
| 13 | CACYBP |  |  | 288 | 0.330 | 0.1320 | Yes |
| 14 | HSPD1 |  |  | 291 | 0.328 | 0.1555 | Yes |
| 15 | SORD |  |  | 317 | 0.320 | 0.1543 | Yes |
| 16 | IDI1 |  |  | 334 | 0.314 | 0.1622 | Yes |
| 17 | CCNF |  |  | 366 | 0.305 | 0.1536 | No |
| 18 | RRM2 |  |  | 383 | 0.300 | 0.1603 | No |
| 19 | P4HA1 |  |  | 411 | 0.290 | 0.1547 | No |
| 20 | HSPE1 |  |  | 465 | 0.277 | 0.1210 | No |
| 21 | PSAT1 |  |  | 491 | 0.271 | 0.1161 | No |
| 22 | QDPR |  |  | 517 | 0.267 | 0.1108 | No |
| 23 | POLR3G |  |  | 561 | 0.258 | 0.0861 | No |
| 24 | DDX39A |  |  | 562 | 0.258 | 0.1062 | No |
| 25 | CYP51A1 |  |  | 759 | -0.349 | -0.0712 | No |
| 26 | SQSTM1 |  |  | 795 | -0.379 | -0.0782 | No |
| 27 | PLOD2 |  |  | 838 | -0.431 | -0.0884 | No |
| 28 | VLDLR |  |  | 879 | -0.503 | -0.0909 | No |
| 29 | CDKN1A |  |  | 969 | -0.987 | -0.1068 | No |
| 30 | IGFBP5 |  |  | 983 | -1.595 | 0.0042 | No |
Table: GSEA details [plain text format]

  

Fig 2: HALLMARK\_MTORC1\_SIGNALING: Random ES distribution      
 Gene set null distribution of ES for **HALLMARK\_MTORC1\_SIGNALING**

  
