# Supplementary material for: Modeling glioblastoma heterogeneity as a dynamic network of cell states
Source: Mol Syst Biol. 2021 Sep 16;17(9):e10105. doi: 10.15252/msb.202010105 (PMC8444284; doi:10.15252/msb.202010105)
Supplement: Supplementary file 6 — Source Data for Figure 5 [file MSB-17-e10105-s004.zip › Figure5A_sourcedata/GSEA_3017/hallmarks_stateA.GseaPreranked.1621934654007/HALLMARK_MYC_TARGETS_V1.html]

Details for gene set HALLMARK\_MYC\_TARGETS\_V1[GSEA]

|  || Dataset | state53017 |
| Phenotype | NoPhenotypeAvailable |
| Upregulated in class | na\_pos |
| GeneSet | HALLMARK\_MYC\_TARGETS\_V1 |
| Enrichment Score (ES) | 0.46499473 |
| Normalized Enrichment Score (NES) | 2.4497974 |
| Nominal p-value | 0.0 |
| FDR q-value | 0.0 |
| FWER p-Value | 0.0 |
Table: GSEA Results Summary

  

Fig 1: Enrichment plot: HALLMARK\_MYC\_TARGETS\_V1      
 Profile of the Running ES Score & Positions of GeneSet Members on the Rank Ordered List

  

| PROBE | GENE SYMBOL | GENE\_TITLE | RANK IN GENE LIST | RANK METRIC SCORE | RUNNING ES | CORE ENRICHMENT || 1 | BUB3 |  |  | 12 | 0.740 | 0.0529 | Yes |
| 2 | KPNA2 |  |  | 20 | 0.667 | 0.1046 | Yes |
| 3 | DDX21 |  |  | 53 | 0.525 | 0.1176 | Yes |
| 4 | CCNA2 |  |  | 65 | 0.495 | 0.1499 | Yes |
| 5 | CDC20 |  |  | 80 | 0.472 | 0.1770 | Yes |
| 6 | DUT |  |  | 87 | 0.466 | 0.2120 | Yes |
| 7 | PRDX3 |  |  | 100 | 0.453 | 0.2395 | Yes |
| 8 | MAD2L1 |  |  | 153 | 0.406 | 0.2211 | Yes |
| 9 | MYC |  |  | 169 | 0.395 | 0.2403 | Yes |
| 10 | MCM7 |  |  | 171 | 0.394 | 0.2742 | Yes |
| 11 | IMPDH2 |  |  | 211 | 0.370 | 0.2662 | Yes |
| 12 | C1QBP |  |  | 212 | 0.369 | 0.2988 | Yes |
| 13 | EIF4A1 |  |  | 229 | 0.362 | 0.3141 | Yes |
| 14 | HSPD1 |  |  | 291 | 0.328 | 0.2794 | Yes |
| 15 | NPM1 |  |  | 292 | 0.328 | 0.3084 | Yes |
| 16 | RPS6 |  |  | 306 | 0.324 | 0.3234 | Yes |
| 17 | RPS3 |  |  | 356 | 0.307 | 0.2993 | Yes |
| 18 | HSP90AB1 |  |  | 363 | 0.306 | 0.3201 | Yes |
| 19 | PA2G4 |  |  | 402 | 0.293 | 0.3063 | Yes |
| 20 | CCT5 |  |  | 409 | 0.290 | 0.3257 | Yes |
| 21 | NOLC1 |  |  | 425 | 0.288 | 0.3355 | Yes |
| 22 | CDC45 |  |  | 446 | 0.282 | 0.3395 | Yes |
| 23 | FBL |  |  | 451 | 0.281 | 0.3601 | Yes |
| 24 | ODC1 |  |  | 460 | 0.279 | 0.3764 | Yes |
| 25 | HSPE1 |  |  | 465 | 0.277 | 0.3968 | Yes |
| 26 | RPS5 |  |  | 476 | 0.274 | 0.4106 | Yes |
| 27 | LSM2 |  |  | 492 | 0.271 | 0.4189 | Yes |
| 28 | NOP16 |  |  | 499 | 0.270 | 0.4365 | Yes |
| 29 | NME1 |  |  | 509 | 0.269 | 0.4509 | Yes |
| 30 | RANBP1 |  |  | 536 | 0.263 | 0.4470 | Yes |
| 31 | SRSF2 |  |  | 542 | 0.262 | 0.4650 | Yes |
Table: GSEA details [plain text format]

  

Fig 2: HALLMARK\_MYC\_TARGETS\_V1: Random ES distribution      
 Gene set null distribution of ES for **HALLMARK\_MYC\_TARGETS\_V1**

  
