# Supplementary material for: Modeling glioblastoma heterogeneity as a dynamic network of cell states
Source: Mol Syst Biol. 2021 Sep 16;17(9):e10105. doi: 10.15252/msb.202010105 (PMC8444284; doi:10.15252/msb.202010105)
Supplement: Supplementary file 6 — Source Data for Figure 5 [file MSB-17-e10105-s004.zip › Figure5A_sourcedata/GSEA_3017/hallmarks_stateA.GseaPreranked.1621934654007/HALLMARK_MYOGENESIS.html]

Details for gene set HALLMARK\_MYOGENESIS[GSEA]

|  || Dataset | state53017 |
| Phenotype | NoPhenotypeAvailable |
| Upregulated in class | na\_neg |
| GeneSet | HALLMARK\_MYOGENESIS |
| Enrichment Score (ES) | -0.5189362 |
| Normalized Enrichment Score (NES) | -1.9907963 |
| Nominal p-value | 0.0063391444 |
| FDR q-value | 0.010965373 |
| FWER p-Value | 0.049 |
Table: GSEA Results Summary

  

Fig 1: Enrichment plot: HALLMARK\_MYOGENESIS      
 Profile of the Running ES Score & Positions of GeneSet Members on the Rank Ordered List

  

| PROBE | GENE SYMBOL | GENE\_TITLE | RANK IN GENE LIST | RANK METRIC SCORE | RUNNING ES | CORE ENRICHMENT || 1 | IGFBP3 |  |  | 26 | 0.641 | 0.0409 | No |
| 2 | ADAM12 |  |  | 90 | 0.464 | 0.0249 | No |
| 3 | ITGB1 |  |  | 256 | 0.346 | -0.1088 | No |
| 4 | ABLIM1 |  |  | 452 | 0.280 | -0.2804 | No |
| 5 | EFS |  |  | 487 | 0.272 | -0.2867 | No |
| 6 | NOTCH1 |  |  | 666 | -0.280 | -0.4409 | No |
| 7 | CKB |  |  | 676 | -0.287 | -0.4199 | No |
| 8 | ITGA7 |  |  | 773 | -0.362 | -0.4807 | Yes |
| 9 | IGFBP7 |  |  | 780 | -0.368 | -0.4480 | Yes |
| 10 | SPARC |  |  | 782 | -0.369 | -0.4101 | Yes |
| 11 | GNAO1 |  |  | 788 | -0.373 | -0.3758 | Yes |
| 12 | APP |  |  | 812 | -0.394 | -0.3580 | Yes |
| 13 | FHL1 |  |  | 814 | -0.397 | -0.3170 | Yes |
| 14 | ITGB5 |  |  | 903 | -0.558 | -0.3488 | Yes |
| 15 | FST |  |  | 911 | -0.576 | -0.2952 | Yes |
| 16 | CLU |  |  | 938 | -0.685 | -0.2496 | Yes |
| 17 | CNN3 |  |  | 961 | -0.883 | -0.1789 | Yes |
| 18 | CRYAB |  |  | 967 | -0.940 | -0.0847 | Yes |
| 19 | CDKN1A |  |  | 969 | -0.987 | 0.0186 | Yes |
Table: GSEA details [plain text format]

  

Fig 2: HALLMARK\_MYOGENESIS: Random ES distribution      
 Gene set null distribution of ES for **HALLMARK\_MYOGENESIS**

  
