# Supplementary material for: Modeling glioblastoma heterogeneity as a dynamic network of cell states
Source: Mol Syst Biol. 2021 Sep 16;17(9):e10105. doi: 10.15252/msb.202010105 (PMC8444284; doi:10.15252/msb.202010105)
Supplement: Supplementary file 6 — Source Data for Figure 5 [file MSB-17-e10105-s004.zip › Figure5A_sourcedata/GSEA_3017/hallmarks_stateA.GseaPreranked.1621934654007/HALLMARK_P53_PATHWAY.html]

Details for gene set HALLMARK\_P53\_PATHWAY[GSEA]

|  || Dataset | state53017 |
| Phenotype | NoPhenotypeAvailable |
| Upregulated in class | na\_neg |
| GeneSet | HALLMARK\_P53\_PATHWAY |
| Enrichment Score (ES) | -0.41636986 |
| Normalized Enrichment Score (NES) | -1.7392999 |
| Nominal p-value | 0.023026315 |
| FDR q-value | 0.036885582 |
| FWER p-Value | 0.207 |
Table: GSEA Results Summary

  

Fig 1: Enrichment plot: HALLMARK\_P53\_PATHWAY      
 Profile of the Running ES Score & Positions of GeneSet Members on the Rank Ordered List

  

| PROBE | GENE SYMBOL | GENE\_TITLE | RANK IN GENE LIST | RANK METRIC SCORE | RUNNING ES | CORE ENRICHMENT || 1 | TM4SF1 |  |  | 1 | 1.087 | 0.1083 | No |
| 2 | S100A4 |  |  | 148 | 0.409 | -0.0016 | No |
| 3 | ADA |  |  | 353 | 0.308 | -0.1818 | No |
| 4 | RPS12 |  |  | 479 | 0.274 | -0.2836 | No |
| 5 | PITPNC1 |  |  | 483 | 0.273 | -0.2592 | No |
| 6 | ZFP36L1 |  |  | 532 | 0.264 | -0.2824 | No |
| 7 | CD82 |  |  | 597 | 0.250 | -0.3235 | No |
| 8 | ABAT |  |  | 617 | -0.259 | -0.3171 | No |
| 9 | RGS16 |  |  | 620 | -0.260 | -0.2930 | No |
| 10 | NOTCH1 |  |  | 666 | -0.280 | -0.3115 | No |
| 11 | RPS27L |  |  | 688 | -0.295 | -0.3035 | No |
| 12 | PHLDA3 |  |  | 798 | -0.382 | -0.3779 | Yes |
| 13 | APP |  |  | 812 | -0.394 | -0.3518 | Yes |
| 14 | ATF3 |  |  | 836 | -0.428 | -0.3325 | Yes |
| 15 | MDM2 |  |  | 847 | -0.442 | -0.2983 | Yes |
| 16 | UPP1 |  |  | 867 | -0.467 | -0.2710 | Yes |
| 17 | JUN |  |  | 886 | -0.525 | -0.2368 | Yes |
| 18 | PLK2 |  |  | 901 | -0.551 | -0.1959 | Yes |
| 19 | SAT1 |  |  | 909 | -0.573 | -0.1455 | Yes |
| 20 | PDGFA |  |  | 913 | -0.581 | -0.0901 | Yes |
| 21 | ZMAT3 |  |  | 929 | -0.650 | -0.0403 | Yes |
| 22 | CDKN1A |  |  | 969 | -0.987 | 0.0186 | Yes |
Table: GSEA details [plain text format]

  

Fig 2: HALLMARK\_P53\_PATHWAY: Random ES distribution      
 Gene set null distribution of ES for **HALLMARK\_P53\_PATHWAY**

  
