# Supplementary material for: Modeling glioblastoma heterogeneity as a dynamic network of cell states
Source: Mol Syst Biol. 2021 Sep 16;17(9):e10105. doi: 10.15252/msb.202010105 (PMC8444284; doi:10.15252/msb.202010105)
Supplement: Supplementary file 6 — Source Data for Figure 5 [file MSB-17-e10105-s004.zip › Figure5A_sourcedata/GSEA_3017/hallmarks_stateA.GseaPreranked.1621934654007/HALLMARK_TNFA_SIGNALING_VIA_NFKB.html]

Details for gene set HALLMARK\_TNFA\_SIGNALING\_VIA\_NFKB[GSEA]

|  || Dataset | state53017 |
| Phenotype | NoPhenotypeAvailable |
| Upregulated in class | na\_neg |
| GeneSet | HALLMARK\_TNFA\_SIGNALING\_VIA\_NFKB |
| Enrichment Score (ES) | -0.5081107 |
| Normalized Enrichment Score (NES) | -2.2592337 |
| Nominal p-value | 0.0016313214 |
| FDR q-value | 2.6923078E-4 |
| FWER p-Value | 0.001 |
Table: GSEA Results Summary

  

Fig 1: Enrichment plot: HALLMARK\_TNFA\_SIGNALING\_VIA\_NFKB      
 Profile of the Running ES Score & Positions of GeneSet Members on the Rank Ordered List

  

| PROBE | GENE SYMBOL | GENE\_TITLE | RANK IN GENE LIST | RANK METRIC SCORE | RUNNING ES | CORE ENRICHMENT || 1 | TNFAIP6 |  |  | 99 | 0.455 | -0.0749 | No |
| 2 | TGIF1 |  |  | 107 | 0.447 | -0.0542 | No |
| 3 | TNC |  |  | 159 | 0.402 | -0.0823 | No |
| 4 | MYC |  |  | 169 | 0.395 | -0.0669 | No |
| 5 | NFIL3 |  |  | 204 | 0.371 | -0.0792 | No |
| 6 | B4GALT5 |  |  | 214 | 0.369 | -0.0654 | No |
| 7 | PLAU |  |  | 347 | 0.310 | -0.1839 | No |
| 8 | BCL3 |  |  | 455 | 0.280 | -0.2782 | No |
| 9 | VEGFA |  |  | 546 | 0.261 | -0.3559 | No |
| 10 | ABCA1 |  |  | 647 | -0.268 | -0.4436 | No |
| 11 | SOCS3 |  |  | 651 | -0.270 | -0.4297 | No |
| 12 | ID2 |  |  | 727 | -0.323 | -0.4879 | Yes |
| 13 | HES1 |  |  | 736 | -0.331 | -0.4754 | Yes |
| 14 | JAG1 |  |  | 745 | -0.339 | -0.4625 | Yes |
| 15 | IRS2 |  |  | 781 | -0.368 | -0.4760 | Yes |
| 16 | PNRC1 |  |  | 786 | -0.371 | -0.4570 | Yes |
| 17 | TUBB2A |  |  | 792 | -0.375 | -0.4387 | Yes |
| 18 | SQSTM1 |  |  | 795 | -0.379 | -0.4170 | Yes |
| 19 | ATF3 |  |  | 836 | -0.428 | -0.4320 | Yes |
| 20 | PMEPA1 |  |  | 877 | -0.497 | -0.4426 | Yes |
| 21 | JUN |  |  | 886 | -0.525 | -0.4181 | Yes |
| 22 | F3 |  |  | 888 | -0.526 | -0.3861 | Yes |
| 23 | PLK2 |  |  | 901 | -0.551 | -0.3641 | Yes |
| 24 | SAT1 |  |  | 909 | -0.573 | -0.3355 | Yes |
| 25 | PTX3 |  |  | 919 | -0.603 | -0.3071 | Yes |
| 26 | CEBPD |  |  | 939 | -0.690 | -0.2836 | Yes |
| 27 | RCAN1 |  |  | 946 | -0.747 | -0.2431 | Yes |
| 28 | SERPINE1 |  |  | 951 | -0.774 | -0.1987 | Yes |
| 29 | CD44 |  |  | 957 | -0.851 | -0.1506 | Yes |
| 30 | CDKN1A |  |  | 969 | -0.987 | -0.1002 | Yes |
| 31 | CCL2 |  |  | 985 | -1.881 | 0.0021 | Yes |
Table: GSEA details [plain text format]

  

Fig 2: HALLMARK\_TNFA\_SIGNALING\_VIA\_NFKB: Random ES distribution      
 Gene set null distribution of ES for **HALLMARK\_TNFA\_SIGNALING\_VIA\_NFKB**

  
