# Supplementary material for: Modeling glioblastoma heterogeneity as a dynamic network of cell states
Source: Mol Syst Biol. 2021 Sep 16;17(9):e10105. doi: 10.15252/msb.202010105 (PMC8444284; doi:10.15252/msb.202010105)
Supplement: Supplementary file 6 — Source Data for Figure 5 [file MSB-17-e10105-s004.zip › Figure5A_sourcedata/GSEA_3017/hallmarks_stateA.GseaPreranked.1621934654007/HALLMARK_UV_RESPONSE_DN.html]

Details for gene set HALLMARK\_UV\_RESPONSE\_DN[GSEA]

|  || Dataset | state53017 |
| Phenotype | NoPhenotypeAvailable |
| Upregulated in class | na\_neg |
| GeneSet | HALLMARK\_UV\_RESPONSE\_DN |
| Enrichment Score (ES) | -0.521325 |
| Normalized Enrichment Score (NES) | -2.3374352 |
| Nominal p-value | 0.0 |
| FDR q-value | 0.0 |
| FWER p-Value | 0.0 |
Table: GSEA Results Summary

  

Fig 1: Enrichment plot: HALLMARK\_UV\_RESPONSE\_DN      
 Profile of the Running ES Score & Positions of GeneSet Members on the Rank Ordered List

  

| PROBE | GENE SYMBOL | GENE\_TITLE | RANK IN GENE LIST | RANK METRIC SCORE | RUNNING ES | CORE ENRICHMENT || 1 | HAS2 |  |  | 45 | 0.551 | -0.0090 | No |
| 2 | MMP16 |  |  | 74 | 0.477 | -0.0055 | No |
| 3 | MYC |  |  | 169 | 0.395 | -0.0763 | No |
| 4 | MGMT |  |  | 301 | 0.325 | -0.1904 | No |
| 5 | RBPMS |  |  | 372 | 0.302 | -0.2426 | No |
| 6 | FZD2 |  |  | 551 | 0.259 | -0.4102 | No |
| 7 | PTEN |  |  | 572 | 0.255 | -0.4135 | No |
| 8 | ZMIZ1 |  |  | 573 | 0.255 | -0.3960 | No |
| 9 | NR3C1 |  |  | 610 | -0.256 | -0.4159 | No |
| 10 | DLG1 |  |  | 611 | -0.257 | -0.3982 | No |
| 11 | SIPA1L1 |  |  | 645 | -0.267 | -0.4143 | No |
| 12 | IRS1 |  |  | 657 | -0.273 | -0.4070 | No |
| 13 | PRKAR2B |  |  | 726 | -0.323 | -0.4557 | No |
| 14 | BDNF |  |  | 790 | -0.374 | -0.4957 | Yes |
| 15 | LTBP1 |  |  | 815 | -0.397 | -0.4934 | Yes |
| 16 | GJA1 |  |  | 823 | -0.405 | -0.4728 | Yes |
| 17 | DDAH1 |  |  | 828 | -0.413 | -0.4487 | Yes |
| 18 | MAP1B |  |  | 851 | -0.444 | -0.4411 | Yes |
| 19 | ANXA2 |  |  | 872 | -0.482 | -0.4289 | Yes |
| 20 | VLDLR |  |  | 879 | -0.503 | -0.4006 | Yes |
| 21 | F3 |  |  | 888 | -0.526 | -0.3729 | Yes |
| 22 | SDC2 |  |  | 908 | -0.568 | -0.3537 | Yes |
| 23 | ID1 |  |  | 931 | -0.651 | -0.3319 | Yes |
| 24 | SERPINE1 |  |  | 951 | -0.774 | -0.2986 | Yes |
| 25 | COL5A2 |  |  | 958 | -0.856 | -0.2461 | Yes |
| 26 | RND3 |  |  | 959 | -0.864 | -0.1867 | Yes |
| 27 | PMP22 |  |  | 982 | -1.519 | -0.1053 | Yes |
| 28 | IGFBP5 |  |  | 983 | -1.595 | 0.0042 | Yes |
Table: GSEA details [plain text format]

  

Fig 2: HALLMARK\_UV\_RESPONSE\_DN: Random ES distribution      
 Gene set null distribution of ES for **HALLMARK\_UV\_RESPONSE\_DN**

  
