# Supplementary material for: Modeling glioblastoma heterogeneity as a dynamic network of cell states
Source: Mol Syst Biol. 2021 Sep 16;17(9):e10105. doi: 10.15252/msb.202010105 (PMC8444284; doi:10.15252/msb.202010105)
Supplement: Supplementary file 6 — Source Data for Figure 5 [file MSB-17-e10105-s004.zip › Figure5A_sourcedata/GSEA_3017/hallmarks_stateB.GseaPreranked.1621934634368/gsea_report_for_na_neg_1621934634368.html]

Report for na\_neg 1621934634368 [GSEA]

| GS  follow link to MSigDB | GS DETAILS | SIZE | ES | NES | NOM p-val | FDR q-val | FWER p-val | RANK AT MAX | LEADING EDGE || 1 | HALLMARK\_ESTROGEN\_RESPONSE\_LATE | Details ... | 25 | -0.16 | -0.97 | 0.470 | 0.933 | 0.630 | 2 | tags=4%, list=0%, signal=4% |
| 2 | HALLMARK\_HYPOXIA | Details ... | 18 | -0.18 | -0.92 | 0.517 | 0.541 | 0.678 | 63 | tags=28%, list=8%, signal=30% |
Table: Gene sets enriched in phenotype **na**[plain text format]****

  
