# Supplementary material for: Modeling glioblastoma heterogeneity as a dynamic network of cell states
Source: Mol Syst Biol. 2021 Sep 16;17(9):e10105. doi: 10.15252/msb.202010105 (PMC8444284; doi:10.15252/msb.202010105)
Supplement: Supplementary file 6 — Source Data for Figure 5 [file MSB-17-e10105-s004.zip › Figure5A_sourcedata/GSEA_3017/hallmarks_stateB.GseaPreranked.1621934634368/gsea_report_for_na_pos_1621934634368.html]

Report for na\_pos 1621934634368 [GSEA]

| GS  follow link to MSigDB | GS DETAILS | SIZE | ES | NES | NOM p-val | FDR q-val | FWER p-val | RANK AT MAX | LEADING EDGE || 1 | HALLMARK\_G2M\_CHECKPOINT | Details ... | 78 | 0.57 | 3.26 | 0.000 | 0.000 | 0.000 | 194 | tags=67%, list=26%, signal=80% |
| 2 | HALLMARK\_E2F\_TARGETS | Details ... | 105 | 0.49 | 2.93 | 0.000 | 0.000 | 0.000 | 286 | tags=72%, list=38%, signal=100% |
| 3 | HALLMARK\_MITOTIC\_SPINDLE | Details ... | 51 | 0.52 | 2.63 | 0.000 | 0.000 | 0.000 | 194 | tags=61%, list=26%, signal=76% |
| 4 | HALLMARK\_SPERMATOGENESIS | Details ... | 15 | 0.49 | 1.76 | 0.011 | 0.033 | 0.124 | 250 | tags=73%, list=33%, signal=107% |
| 5 | HALLMARK\_GLYCOLYSIS | Details ... | 16 | 0.39 | 1.43 | 0.089 | 0.208 | 0.665 | 224 | tags=56%, list=29%, signal=78% |
| 6 | HALLMARK\_MYC\_TARGETS\_V1 | Details ... | 21 | 0.34 | 1.40 | 0.123 | 0.198 | 0.722 | 199 | tags=48%, list=26%, signal=63% |
| 7 | HALLMARK\_APOPTOSIS | Details ... | 17 | 0.35 | 1.33 | 0.166 | 0.246 | 0.852 | 171 | tags=41%, list=23%, signal=52% |
| 8 | HALLMARK\_EPITHELIAL\_MESENCHYMAL\_TRANSITION | Details ... | 29 | 0.26 | 1.14 | 0.300 | 0.429 | 0.979 | 226 | tags=41%, list=30%, signal=57% |
| 9 | HALLMARK\_MTORC1\_SIGNALING | Details ... | 25 | 0.27 | 1.13 | 0.325 | 0.402 | 0.983 | 113 | tags=28%, list=15%, signal=32% |
| 10 | HALLMARK\_DNA\_REPAIR | Details ... | 20 | 0.25 | 0.99 | 0.465 | 0.527 | 0.998 | 331 | tags=60%, list=44%, signal=103% |
| 11 | HALLMARK\_UV\_RESPONSE\_DN | Details ... | 16 | 0.26 | 0.95 | 0.503 | 0.523 | 1.000 | 107 | tags=25%, list=14%, signal=28% |
Table: Gene sets enriched in phenotype **na**[plain text format]****

  
