# Supplementary material for: Modeling glioblastoma heterogeneity as a dynamic network of cell states
Source: Mol Syst Biol. 2021 Sep 16;17(9):e10105. doi: 10.15252/msb.202010105 (PMC8444284; doi:10.15252/msb.202010105)
Supplement: Supplementary file 6 — Source Data for Figure 5 [file MSB-17-e10105-s004.zip › Figure5A_sourcedata/GSEA_3017/hallmarks_stateB.GseaPreranked.1621934634368/HALLMARK_APOPTOSIS.html]

Details for gene set HALLMARK\_APOPTOSIS[GSEA]

|  || Dataset | state43017 |
| Phenotype | NoPhenotypeAvailable |
| Upregulated in class | na\_pos |
| GeneSet | HALLMARK\_APOPTOSIS |
| Enrichment Score (ES) | 0.34846833 |
| Normalized Enrichment Score (NES) | 1.3262141 |
| Nominal p-value | 0.16625616 |
| FDR q-value | 0.2458785 |
| FWER p-Value | 0.852 |
Table: GSEA Results Summary

  

Fig 1: Enrichment plot: HALLMARK\_APOPTOSIS      
 Profile of the Running ES Score & Positions of GeneSet Members on the Rank Ordered List

  

| PROBE | GENE SYMBOL | GENE\_TITLE | RANK IN GENE LIST | RANK METRIC SCORE | RUNNING ES | CORE ENRICHMENT || 1 | TOP2A |  |  | 3 | 1.079 | 0.1401 | Yes |
| 2 | HMGB2 |  |  | 14 | 0.786 | 0.2316 | Yes |
| 3 | MMP2 |  |  | 80 | 0.565 | 0.2196 | Yes |
| 4 | TNFRSF12A |  |  | 134 | 0.484 | 0.2129 | Yes |
| 5 | CD44 |  |  | 146 | 0.464 | 0.2601 | Yes |
| 6 | ANXA1 |  |  | 160 | 0.453 | 0.3031 | Yes |
| 7 | BRCA1 |  |  | 171 | 0.440 | 0.3485 | Yes |
| 8 | EMP1 |  |  | 235 | 0.390 | 0.3157 | No |
| 9 | CDC25B |  |  | 286 | 0.356 | 0.2960 | No |
| 10 | BTG3 |  |  | 337 | 0.331 | 0.2729 | No |
| 11 | TGFB2 |  |  | 388 | 0.312 | 0.2472 | No |
| 12 | CDKN1B |  |  | 427 | 0.298 | 0.2359 | No |
| 13 | IFITM3 |  |  | 512 | 0.279 | 0.1600 | No |
| 14 | CDK2 |  |  | 565 | 0.270 | 0.1261 | No |
| 15 | DPYD |  |  | 634 | 0.258 | 0.0690 | No |
| 16 | TSPO |  |  | 723 | -0.345 | -0.0034 | No |
| 17 | HSPB1 |  |  | 730 | -0.378 | 0.0390 | No |
Table: GSEA details [plain text format]

  

Fig 2: HALLMARK\_APOPTOSIS: Random ES distribution      
 Gene set null distribution of ES for **HALLMARK\_APOPTOSIS**

  
