# Supplementary material for: Modeling glioblastoma heterogeneity as a dynamic network of cell states
Source: Mol Syst Biol. 2021 Sep 16;17(9):e10105. doi: 10.15252/msb.202010105 (PMC8444284; doi:10.15252/msb.202010105)
Supplement: Supplementary file 6 — Source Data for Figure 5 [file MSB-17-e10105-s004.zip › Figure5A_sourcedata/GSEA_3017/hallmarks_stateB.GseaPreranked.1621934634368/HALLMARK_DNA_REPAIR.html]

Details for gene set HALLMARK\_DNA\_REPAIR[GSEA]

|  || Dataset | state43017 |
| Phenotype | NoPhenotypeAvailable |
| Upregulated in class | na\_pos |
| GeneSet | HALLMARK\_DNA\_REPAIR |
| Enrichment Score (ES) | 0.25295308 |
| Normalized Enrichment Score (NES) | 0.98886853 |
| Nominal p-value | 0.4647202 |
| FDR q-value | 0.52748406 |
| FWER p-Value | 0.998 |
Table: GSEA Results Summary

  

Fig 1: Enrichment plot: HALLMARK\_DNA\_REPAIR      
 Profile of the Running ES Score & Positions of GeneSet Members on the Rank Ordered List

  

| PROBE | GENE SYMBOL | GENE\_TITLE | RANK IN GENE LIST | RANK METRIC SCORE | RUNNING ES | CORE ENRICHMENT || 1 | TYMS |  |  | 63 | 0.601 | -0.0007 | Yes |
| 2 | DUT |  |  | 124 | 0.494 | -0.0125 | Yes |
| 3 | FEN1 |  |  | 180 | 0.435 | -0.0258 | Yes |
| 4 | ZWINT |  |  | 212 | 0.407 | -0.0105 | Yes |
| 5 | LIG1 |  |  | 214 | 0.407 | 0.0452 | Yes |
| 6 | PCNA |  |  | 242 | 0.386 | 0.0629 | Yes |
| 7 | POLA1 |  |  | 247 | 0.383 | 0.1113 | Yes |
| 8 | POLD3 |  |  | 252 | 0.377 | 0.1588 | Yes |
| 9 | RFC5 |  |  | 260 | 0.370 | 0.2013 | Yes |
| 10 | RFC4 |  |  | 295 | 0.351 | 0.2047 | Yes |
| 11 | RFC3 |  |  | 322 | 0.338 | 0.2170 | Yes |
| 12 | RFC2 |  |  | 331 | 0.333 | 0.2530 | Yes |
| 13 | POLA2 |  |  | 380 | 0.314 | 0.2322 | No |
| 14 | MPC2 |  |  | 412 | 0.303 | 0.2329 | No |
| 15 | POLH |  |  | 492 | 0.282 | 0.1657 | No |
| 16 | SAC3D1 |  |  | 529 | 0.276 | 0.1558 | No |
| 17 | POLR2A |  |  | 545 | 0.273 | 0.1738 | No |
| 18 | ALYREF |  |  | 546 | 0.273 | 0.2121 | No |
| 19 | RPA3 |  |  | 584 | 0.267 | 0.1997 | No |
| 20 | RAD51 |  |  | 670 | 0.252 | 0.1203 | No |
Table: GSEA details [plain text format]

  

Fig 2: HALLMARK\_DNA\_REPAIR: Random ES distribution      
 Gene set null distribution of ES for **HALLMARK\_DNA\_REPAIR**

  
