# Supplementary material for: Modeling glioblastoma heterogeneity as a dynamic network of cell states
Source: Mol Syst Biol. 2021 Sep 16;17(9):e10105. doi: 10.15252/msb.202010105 (PMC8444284; doi:10.15252/msb.202010105)
Supplement: Supplementary file 6 — Source Data for Figure 5 [file MSB-17-e10105-s004.zip › Figure5A_sourcedata/GSEA_3017/hallmarks_stateB.GseaPreranked.1621934634368/HALLMARK_E2F_TARGETS.html]

Details for gene set HALLMARK\_E2F\_TARGETS[GSEA]

|  || Dataset | state43017 |
| Phenotype | NoPhenotypeAvailable |
| Upregulated in class | na\_pos |
| GeneSet | HALLMARK\_E2F\_TARGETS |
| Enrichment Score (ES) | 0.49304935 |
| Normalized Enrichment Score (NES) | 2.92544 |
| Nominal p-value | 0.0 |
| FDR q-value | 0.0 |
| FWER p-Value | 0.0 |
Table: GSEA Results Summary

  

Fig 1: Enrichment plot: HALLMARK\_E2F\_TARGETS      
 Profile of the Running ES Score & Positions of GeneSet Members on the Rank Ordered List

  

| PROBE | GENE SYMBOL | GENE\_TITLE | RANK IN GENE LIST | RANK METRIC SCORE | RUNNING ES | CORE ENRICHMENT || 1 | TOP2A |  |  | 3 | 1.079 | 0.0180 | Yes |
| 2 | RRM2 |  |  | 7 | 0.875 | 0.0317 | Yes |
| 3 | HMGB2 |  |  | 14 | 0.786 | 0.0389 | Yes |
| 4 | SPC25 |  |  | 17 | 0.763 | 0.0518 | Yes |
| 5 | TACC3 |  |  | 21 | 0.741 | 0.0628 | Yes |
| 6 | ATAD2 |  |  | 28 | 0.685 | 0.0679 | Yes |
| 7 | KPNA2 |  |  | 33 | 0.676 | 0.0759 | Yes |
| 8 | KIF4A |  |  | 37 | 0.662 | 0.0852 | Yes |
| 9 | RAD51AP1 |  |  | 40 | 0.657 | 0.0959 | Yes |
| 10 | SMC4 |  |  | 41 | 0.655 | 0.1095 | Yes |
| 11 | MELK |  |  | 42 | 0.655 | 0.1232 | Yes |
| 12 | CENPE |  |  | 43 | 0.654 | 0.1369 | Yes |
| 13 | DLGAP5 |  |  | 46 | 0.647 | 0.1474 | Yes |
| 14 | AURKA |  |  | 55 | 0.617 | 0.1481 | Yes |
| 15 | MYBL2 |  |  | 58 | 0.612 | 0.1578 | Yes |
| 16 | BUB1B |  |  | 64 | 0.596 | 0.1626 | Yes |
| 17 | KIF2C |  |  | 66 | 0.591 | 0.1734 | Yes |
| 18 | CDK1 |  |  | 69 | 0.585 | 0.1826 | Yes |
| 19 | CKS1B |  |  | 72 | 0.575 | 0.1916 | Yes |
| 20 | RACGAP1 |  |  | 75 | 0.572 | 0.2005 | Yes |
| 21 | MAD2L1 |  |  | 77 | 0.569 | 0.2108 | Yes |
| 22 | PLK1 |  |  | 78 | 0.567 | 0.2227 | Yes |
| 23 | CDCA8 |  |  | 82 | 0.562 | 0.2298 | Yes |
| 24 | CKS2 |  |  | 83 | 0.558 | 0.2415 | Yes |
| 25 | UBE2S |  |  | 87 | 0.555 | 0.2485 | Yes |
| 26 | AURKB |  |  | 89 | 0.548 | 0.2584 | Yes |
| 27 | SPC24 |  |  | 91 | 0.544 | 0.2683 | Yes |
| 28 | CENPM |  |  | 92 | 0.544 | 0.2797 | Yes |
| 29 | DEPDC1 |  |  | 97 | 0.536 | 0.2847 | Yes |
| 30 | MXD3 |  |  | 104 | 0.519 | 0.2864 | Yes |
| 31 | CDKN3 |  |  | 108 | 0.513 | 0.2926 | Yes |
| 32 | UBE2T |  |  | 110 | 0.512 | 0.3017 | Yes |
| 33 | CDCA3 |  |  | 111 | 0.511 | 0.3124 | Yes |
| 34 | DIAPH3 |  |  | 112 | 0.510 | 0.3231 | Yes |
| 35 | RAD21 |  |  | 115 | 0.506 | 0.3306 | Yes |
| 36 | BIRC5 |  |  | 116 | 0.504 | 0.3411 | Yes |
| 37 | ASF1B |  |  | 120 | 0.501 | 0.3470 | Yes |
| 38 | PSMC3IP |  |  | 122 | 0.495 | 0.3558 | Yes |
| 39 | KIF18B |  |  | 123 | 0.494 | 0.3662 | Yes |
| 40 | DUT |  |  | 124 | 0.494 | 0.3765 | Yes |
| 41 | E2F8 |  |  | 125 | 0.493 | 0.3868 | Yes |
| 42 | TMPO |  |  | 127 | 0.492 | 0.3955 | Yes |
| 43 | NCAPD2 |  |  | 136 | 0.483 | 0.3934 | Yes |
| 44 | ORC6 |  |  | 150 | 0.461 | 0.3832 | Yes |
| 45 | KIF22 |  |  | 152 | 0.459 | 0.3912 | Yes |
| 46 | SPAG5 |  |  | 156 | 0.455 | 0.3962 | Yes |
| 47 | CDKN2C |  |  | 164 | 0.445 | 0.3948 | Yes |
| 48 | RPA1 |  |  | 165 | 0.445 | 0.4041 | Yes |
| 49 | TK1 |  |  | 167 | 0.444 | 0.4118 | Yes |
| 50 | BRCA1 |  |  | 171 | 0.440 | 0.4165 | Yes |
| 51 | BRCA2 |  |  | 174 | 0.436 | 0.4225 | Yes |
| 52 | MKI67 |  |  | 179 | 0.436 | 0.4255 | Yes |
| 53 | CDC20 |  |  | 184 | 0.431 | 0.4284 | Yes |
| 54 | USP1 |  |  | 185 | 0.430 | 0.4374 | Yes |
| 55 | BARD1 |  |  | 189 | 0.427 | 0.4418 | Yes |
| 56 | HMMR |  |  | 191 | 0.426 | 0.4491 | Yes |
| 57 | MCM4 |  |  | 199 | 0.418 | 0.4472 | Yes |
| 58 | CCP110 |  |  | 204 | 0.411 | 0.4497 | Yes |
| 59 | TCF19 |  |  | 207 | 0.410 | 0.4552 | Yes |
| 60 | LIG1 |  |  | 214 | 0.407 | 0.4545 | Yes |
| 61 | TIMELESS |  |  | 228 | 0.395 | 0.4429 | Yes |
| 62 | EZH2 |  |  | 236 | 0.388 | 0.4403 | Yes |
| 63 | TRIP13 |  |  | 241 | 0.387 | 0.4423 | Yes |
| 64 | PCNA |  |  | 242 | 0.386 | 0.4504 | Yes |
| 65 | CCNB2 |  |  | 250 | 0.379 | 0.4476 | Yes |
| 66 | POLD3 |  |  | 252 | 0.377 | 0.4540 | Yes |
| 67 | PLK4 |  |  | 255 | 0.374 | 0.4587 | Yes |
| 68 | TUBB |  |  | 263 | 0.368 | 0.4557 | Yes |
| 69 | GINS4 |  |  | 265 | 0.367 | 0.4619 | Yes |
| 70 | TUBG1 |  |  | 268 | 0.365 | 0.4665 | Yes |
| 71 | SMC1A |  |  | 274 | 0.361 | 0.4664 | Yes |
| 72 | MMS22L |  |  | 277 | 0.359 | 0.4708 | Yes |
| 73 | ANP32E |  |  | 280 | 0.358 | 0.4752 | Yes |
| 74 | GINS1 |  |  | 283 | 0.357 | 0.4797 | Yes |
| 75 | PSIP1 |  |  | 284 | 0.357 | 0.4871 | Yes |
| 76 | CDC25B |  |  | 286 | 0.356 | 0.4930 | Yes |
| 77 | PRKDC |  |  | 306 | 0.344 | 0.4712 | No |
| 78 | DEK |  |  | 316 | 0.341 | 0.4646 | No |
| 79 | CSE1L |  |  | 321 | 0.338 | 0.4656 | No |
| 80 | RFC3 |  |  | 322 | 0.338 | 0.4726 | No |
| 81 | CIT |  |  | 326 | 0.336 | 0.4751 | No |
| 82 | RFC2 |  |  | 331 | 0.333 | 0.4759 | No |
| 83 | MRE11 |  |  | 332 | 0.333 | 0.4829 | No |
| 84 | UBR7 |  |  | 340 | 0.330 | 0.4791 | No |
| 85 | LMNB1 |  |  | 363 | 0.321 | 0.4522 | No |
| 86 | XPO1 |  |  | 365 | 0.321 | 0.4574 | No |
| 87 | RBBP7 |  |  | 367 | 0.321 | 0.4626 | No |
| 88 | POLA2 |  |  | 380 | 0.314 | 0.4508 | No |
| 89 | DCK |  |  | 383 | 0.313 | 0.4543 | No |
| 90 | PRIM2 |  |  | 391 | 0.311 | 0.4501 | No |
| 91 | PTTG1 |  |  | 413 | 0.302 | 0.4243 | No |
| 92 | DSCC1 |  |  | 422 | 0.299 | 0.4184 | No |
| 93 | CCNE1 |  |  | 424 | 0.299 | 0.4231 | No |
| 94 | CDKN1B |  |  | 427 | 0.298 | 0.4263 | No |
| 95 | NUP107 |  |  | 430 | 0.298 | 0.4295 | No |
| 96 | CBX5 |  |  | 457 | 0.291 | 0.3958 | No |
| 97 | EED |  |  | 459 | 0.290 | 0.4004 | No |
| 98 | CHEK1 |  |  | 477 | 0.286 | 0.3804 | No |
| 99 | RNASEH2A |  |  | 478 | 0.285 | 0.3864 | No |
| 100 | DNMT1 |  |  | 523 | 0.277 | 0.3250 | No |
| 101 | DONSON |  |  | 559 | 0.271 | 0.2772 | No |
| 102 | NUP205 |  |  | 571 | 0.269 | 0.2660 | No |
| 103 | RPA3 |  |  | 584 | 0.267 | 0.2533 | No |
| 104 | RAD1 |  |  | 607 | 0.262 | 0.2252 | No |
| 105 | EXOSC8 |  |  | 647 | 0.256 | 0.1710 | No |
Table: GSEA details [plain text format]

  

Fig 2: HALLMARK\_E2F\_TARGETS: Random ES distribution      
 Gene set null distribution of ES for **HALLMARK\_E2F\_TARGETS**

  
