# Supplementary material for: Modeling glioblastoma heterogeneity as a dynamic network of cell states
Source: Mol Syst Biol. 2021 Sep 16;17(9):e10105. doi: 10.15252/msb.202010105 (PMC8444284; doi:10.15252/msb.202010105)
Supplement: Supplementary file 6 — Source Data for Figure 5 [file MSB-17-e10105-s004.zip › Figure5A_sourcedata/GSEA_3017/hallmarks_stateB.GseaPreranked.1621934634368/HALLMARK_EPITHELIAL_MESENCHYMAL_TRANSITION.html]

Details for gene set HALLMARK\_EPITHELIAL\_MESENCHYMAL\_TRANSITION[GSEA]

|  || Dataset | state43017 |
| Phenotype | NoPhenotypeAvailable |
| Upregulated in class | na\_pos |
| GeneSet | HALLMARK\_EPITHELIAL\_MESENCHYMAL\_TRANSITION |
| Enrichment Score (ES) | 0.26000845 |
| Normalized Enrichment Score (NES) | 1.1437147 |
| Nominal p-value | 0.30046403 |
| FDR q-value | 0.42864308 |
| FWER p-Value | 0.979 |
Table: GSEA Results Summary

  

Fig 1: Enrichment plot: HALLMARK\_EPITHELIAL\_MESENCHYMAL\_TRANSITION      
 Profile of the Running ES Score & Positions of GeneSet Members on the Rank Ordered List

  

| PROBE | GENE SYMBOL | GENE\_TITLE | RANK IN GENE LIST | RANK METRIC SCORE | RUNNING ES | CORE ENRICHMENT || 1 | SERPINE1 |  |  | 8 | 0.873 | 0.0685 | Yes |
| 2 | SPP1 |  |  | 38 | 0.661 | 0.0890 | Yes |
| 3 | PMP22 |  |  | 70 | 0.576 | 0.0990 | Yes |
| 4 | MMP2 |  |  | 80 | 0.565 | 0.1381 | Yes |
| 5 | TNFRSF12A |  |  | 134 | 0.484 | 0.1096 | Yes |
| 6 | CD44 |  |  | 146 | 0.464 | 0.1368 | Yes |
| 7 | ELN |  |  | 182 | 0.433 | 0.1283 | Yes |
| 8 | PMEPA1 |  |  | 198 | 0.418 | 0.1458 | Yes |
| 9 | CADM1 |  |  | 201 | 0.414 | 0.1807 | Yes |
| 10 | NTM |  |  | 209 | 0.409 | 0.2084 | Yes |
| 11 | TPM1 |  |  | 225 | 0.397 | 0.2240 | Yes |
| 12 | ITGB5 |  |  | 226 | 0.396 | 0.2600 | Yes |
| 13 | TFPI2 |  |  | 301 | 0.346 | 0.1903 | No |
| 14 | PRRX1 |  |  | 318 | 0.339 | 0.1993 | No |
| 15 | SPARC |  |  | 350 | 0.325 | 0.1865 | No |
| 16 | FSTL1 |  |  | 362 | 0.321 | 0.2007 | No |
| 17 | LAMC1 |  |  | 438 | 0.295 | 0.1249 | No |
| 18 | MEST |  |  | 441 | 0.294 | 0.1489 | No |
| 19 | PLOD2 |  |  | 474 | 0.286 | 0.1312 | No |
| 20 | APLP1 |  |  | 480 | 0.285 | 0.1503 | No |
| 21 | EFEMP2 |  |  | 507 | 0.280 | 0.1402 | No |
| 22 | COL5A2 |  |  | 516 | 0.278 | 0.1545 | No |
| 23 | CALU |  |  | 552 | 0.272 | 0.1314 | No |
| 24 | CDH2 |  |  | 558 | 0.271 | 0.1493 | No |
| 25 | COL5A3 |  |  | 609 | 0.262 | 0.1047 | No |
| 26 | GPC1 |  |  | 651 | 0.256 | 0.0718 | No |
| 27 | FLNA |  |  | 676 | 0.251 | 0.0619 | No |
| 28 | VIM |  |  | 692 | -0.267 | 0.0656 | No |
| 29 | ITGB1 |  |  | 693 | -0.271 | 0.0903 | No |
Table: GSEA details [plain text format]

  

Fig 2: HALLMARK\_EPITHELIAL\_MESENCHYMAL\_TRANSITION: Random ES distribution      
 Gene set null distribution of ES for **HALLMARK\_EPITHELIAL\_MESENCHYMAL\_TRANSITION**

  
