# Supplementary material for: Modeling glioblastoma heterogeneity as a dynamic network of cell states
Source: Mol Syst Biol. 2021 Sep 16;17(9):e10105. doi: 10.15252/msb.202010105 (PMC8444284; doi:10.15252/msb.202010105)
Supplement: Supplementary file 6 — Source Data for Figure 5 [file MSB-17-e10105-s004.zip › Figure5A_sourcedata/GSEA_3017/hallmarks_stateB.GseaPreranked.1621934634368/HALLMARK_ESTROGEN_RESPONSE_LATE.html]

Details for gene set HALLMARK\_ESTROGEN\_RESPONSE\_LATE[GSEA]

|  || Dataset | state43017 |
| Phenotype | NoPhenotypeAvailable |
| Upregulated in class | na\_neg |
| GeneSet | HALLMARK\_ESTROGEN\_RESPONSE\_LATE |
| Enrichment Score (ES) | -0.15966314 |
| Normalized Enrichment Score (NES) | -0.96879476 |
| Nominal p-value | 0.4695122 |
| FDR q-value | 0.93338186 |
| FWER p-Value | 0.63 |
Table: GSEA Results Summary

  

Fig 1: Enrichment plot: HALLMARK\_ESTROGEN\_RESPONSE\_LATE      
 Profile of the Running ES Score & Positions of GeneSet Members on the Rank Ordered List

  

| PROBE | GENE SYMBOL | GENE\_TITLE | RANK IN GENE LIST | RANK METRIC SCORE | RUNNING ES | CORE ENRICHMENT || 1 | TOP2A |  |  | 3 | 1.079 | 0.1009 | Yes |
| 2 | CD44 |  |  | 146 | 0.464 | -0.0471 | No |
| 3 | RBBP8 |  |  | 176 | 0.436 | -0.0442 | No |
| 4 | CDC20 |  |  | 184 | 0.431 | -0.0117 | No |
| 5 | STIL |  |  | 231 | 0.393 | -0.0361 | No |
| 6 | CDC6 |  |  | 239 | 0.388 | -0.0079 | No |
| 7 | PLK4 |  |  | 255 | 0.374 | 0.0081 | No |
| 8 | TFPI2 |  |  | 301 | 0.346 | -0.0194 | No |
| 9 | OLFM1 |  |  | 325 | 0.337 | -0.0179 | No |
| 10 | PRSS23 |  |  | 329 | 0.334 | 0.0105 | No |
| 11 | EMP2 |  |  | 333 | 0.333 | 0.0388 | No |
| 12 | BTG3 |  |  | 337 | 0.331 | 0.0668 | No |
| 13 | KIF20A |  |  | 392 | 0.311 | 0.0237 | No |
| 14 | MOCS2 |  |  | 431 | 0.296 | 0.0007 | No |
| 15 | MEST |  |  | 441 | 0.294 | 0.0172 | No |
| 16 | CHPT1 |  |  | 445 | 0.294 | 0.0417 | No |
| 17 | HSPA4L |  |  | 461 | 0.290 | 0.0494 | No |
| 18 | RNASEH2A |  |  | 478 | 0.285 | 0.0554 | No |
| 19 | MYOF |  |  | 502 | 0.280 | 0.0514 | No |
| 20 | TSPAN13 |  |  | 528 | 0.276 | 0.0443 | No |
| 21 | ALDH3A2 |  |  | 593 | 0.265 | -0.0170 | No |
| 22 | IDH2 |  |  | 598 | 0.263 | 0.0032 | No |
| 23 | PRKAR2B |  |  | 611 | 0.262 | 0.0123 | No |
| 24 | CA12 |  |  | 698 | -0.274 | -0.0780 | No |
| 25 | FABP5 |  |  | 759 | -1.641 | -0.0000 | No |
Table: GSEA details [plain text format]

  

Fig 2: HALLMARK\_ESTROGEN\_RESPONSE\_LATE: Random ES distribution      
 Gene set null distribution of ES for **HALLMARK\_ESTROGEN\_RESPONSE\_LATE**

  
