# Supplementary material for: Modeling glioblastoma heterogeneity as a dynamic network of cell states
Source: Mol Syst Biol. 2021 Sep 16;17(9):e10105. doi: 10.15252/msb.202010105 (PMC8444284; doi:10.15252/msb.202010105)
Supplement: Supplementary file 6 — Source Data for Figure 5 [file MSB-17-e10105-s004.zip › Figure5A_sourcedata/GSEA_3017/hallmarks_stateB.GseaPreranked.1621934634368/HALLMARK_G2M_CHECKPOINT.html]

Details for gene set HALLMARK\_G2M\_CHECKPOINT[GSEA]

|  || Dataset | state43017 |
| Phenotype | NoPhenotypeAvailable |
| Upregulated in class | na\_pos |
| GeneSet | HALLMARK\_G2M\_CHECKPOINT |
| Enrichment Score (ES) | 0.57088006 |
| Normalized Enrichment Score (NES) | 3.261261 |
| Nominal p-value | 0.0 |
| FDR q-value | 0.0 |
| FWER p-Value | 0.0 |
Table: GSEA Results Summary

  

Fig 1: Enrichment plot: HALLMARK\_G2M\_CHECKPOINT      
 Profile of the Running ES Score & Positions of GeneSet Members on the Rank Ordered List

  

| PROBE | GENE SYMBOL | GENE\_TITLE | RANK IN GENE LIST | RANK METRIC SCORE | RUNNING ES | CORE ENRICHMENT || 1 | TOP2A |  |  | 3 | 1.079 | 0.0232 | Yes |
| 2 | UBE2C |  |  | 11 | 0.802 | 0.0335 | Yes |
| 3 | TPX2 |  |  | 12 | 0.801 | 0.0540 | Yes |
| 4 | NUSAP1 |  |  | 13 | 0.801 | 0.0745 | Yes |
| 5 | FBXO5 |  |  | 15 | 0.785 | 0.0931 | Yes |
| 6 | PRC1 |  |  | 18 | 0.756 | 0.1095 | Yes |
| 7 | TACC3 |  |  | 21 | 0.741 | 0.1256 | Yes |
| 8 | KIF23 |  |  | 24 | 0.721 | 0.1411 | Yes |
| 9 | NDC80 |  |  | 27 | 0.695 | 0.1559 | Yes |
| 10 | KIF15 |  |  | 29 | 0.684 | 0.1720 | Yes |
| 11 | KPNA2 |  |  | 33 | 0.676 | 0.1849 | Yes |
| 12 | CENPF |  |  | 34 | 0.670 | 0.2020 | Yes |
| 13 | KNL1 |  |  | 35 | 0.666 | 0.2191 | Yes |
| 14 | KIF4A |  |  | 37 | 0.662 | 0.2345 | Yes |
| 15 | SMC4 |  |  | 41 | 0.655 | 0.2469 | Yes |
| 16 | CENPE |  |  | 43 | 0.654 | 0.2622 | Yes |
| 17 | CENPA |  |  | 51 | 0.636 | 0.2682 | Yes |
| 18 | CCNA2 |  |  | 54 | 0.626 | 0.2813 | Yes |
| 19 | AURKA |  |  | 55 | 0.617 | 0.2971 | Yes |
| 20 | MYBL2 |  |  | 58 | 0.612 | 0.3098 | Yes |
| 21 | KIF2C |  |  | 66 | 0.591 | 0.3147 | Yes |
| 22 | SMC2 |  |  | 68 | 0.586 | 0.3282 | Yes |
| 23 | CDK1 |  |  | 69 | 0.585 | 0.3432 | Yes |
| 24 | CKS1B |  |  | 72 | 0.575 | 0.3549 | Yes |
| 25 | RACGAP1 |  |  | 75 | 0.572 | 0.3666 | Yes |
| 26 | MAD2L1 |  |  | 77 | 0.569 | 0.3797 | Yes |
| 27 | PLK1 |  |  | 78 | 0.567 | 0.3942 | Yes |
| 28 | DBF4 |  |  | 79 | 0.567 | 0.4087 | Yes |
| 29 | CKS2 |  |  | 83 | 0.558 | 0.4186 | Yes |
| 30 | UBE2S |  |  | 87 | 0.555 | 0.4284 | Yes |
| 31 | AURKB |  |  | 89 | 0.548 | 0.4410 | Yes |
| 32 | PBK |  |  | 95 | 0.540 | 0.4475 | Yes |
| 33 | CCNF |  |  | 106 | 0.513 | 0.4459 | Yes |
| 34 | CDKN3 |  |  | 108 | 0.513 | 0.4576 | Yes |
| 35 | BUB1 |  |  | 113 | 0.508 | 0.4647 | Yes |
| 36 | RAD21 |  |  | 115 | 0.506 | 0.4762 | Yes |
| 37 | BIRC5 |  |  | 116 | 0.504 | 0.4891 | Yes |
| 38 | DTYMK |  |  | 118 | 0.503 | 0.5005 | Yes |
| 39 | TMPO |  |  | 127 | 0.492 | 0.5014 | Yes |
| 40 | TTK |  |  | 137 | 0.482 | 0.5005 | Yes |
| 41 | INCENP |  |  | 140 | 0.476 | 0.5098 | Yes |
| 42 | ORC6 |  |  | 150 | 0.461 | 0.5084 | Yes |
| 43 | KIF22 |  |  | 152 | 0.459 | 0.5187 | Yes |
| 44 | CDC45 |  |  | 154 | 0.456 | 0.5289 | Yes |
| 45 | POLQ |  |  | 162 | 0.450 | 0.5301 | Yes |
| 46 | CDKN2C |  |  | 164 | 0.445 | 0.5400 | Yes |
| 47 | BRCA2 |  |  | 174 | 0.436 | 0.5380 | Yes |
| 48 | MKI67 |  |  | 179 | 0.436 | 0.5433 | Yes |
| 49 | CDC20 |  |  | 184 | 0.431 | 0.5485 | Yes |
| 50 | BARD1 |  |  | 189 | 0.427 | 0.5535 | Yes |
| 51 | HMMR |  |  | 191 | 0.426 | 0.5630 | Yes |
| 52 | NEK2 |  |  | 194 | 0.423 | 0.5709 | Yes |
| 53 | STIL |  |  | 231 | 0.393 | 0.5281 | No |
| 54 | EZH2 |  |  | 236 | 0.388 | 0.5322 | No |
| 55 | CDC6 |  |  | 239 | 0.388 | 0.5392 | No |
| 56 | HMGN2 |  |  | 243 | 0.386 | 0.5447 | No |
| 57 | CCNB2 |  |  | 250 | 0.379 | 0.5456 | No |
| 58 | PLK4 |  |  | 255 | 0.374 | 0.5493 | No |
| 59 | SMC1A |  |  | 274 | 0.361 | 0.5321 | No |
| 60 | CHAF1A |  |  | 276 | 0.359 | 0.5399 | No |
| 61 | CDC25B |  |  | 286 | 0.356 | 0.5358 | No |
| 62 | NSD2 |  |  | 303 | 0.346 | 0.5212 | No |
| 63 | LMNB1 |  |  | 363 | 0.321 | 0.4429 | No |
| 64 | XPO1 |  |  | 365 | 0.321 | 0.4496 | No |
| 65 | KMT5A |  |  | 373 | 0.318 | 0.4475 | No |
| 66 | POLA2 |  |  | 380 | 0.314 | 0.4467 | No |
| 67 | PRIM2 |  |  | 391 | 0.311 | 0.4400 | No |
| 68 | MEIS2 |  |  | 405 | 0.307 | 0.4288 | No |
| 69 | PTTG1 |  |  | 413 | 0.302 | 0.4263 | No |
| 70 | CDKN1B |  |  | 427 | 0.298 | 0.4149 | No |
| 71 | TFDP1 |  |  | 444 | 0.294 | 0.3989 | No |
| 72 | NUP50 |  |  | 470 | 0.287 | 0.3696 | No |
| 73 | CHEK1 |  |  | 477 | 0.286 | 0.3681 | No |
| 74 | CDC27 |  |  | 520 | 0.278 | 0.3136 | No |
| 75 | SAP30 |  |  | 547 | 0.273 | 0.2825 | No |
| 76 | MAP3K20 |  |  | 582 | 0.268 | 0.2395 | No |
| 77 | KPNB1 |  |  | 600 | 0.263 | 0.2213 | No |
| 78 | SQLE |  |  | 735 | -0.405 | 0.0352 | No |
Table: GSEA details [plain text format]

  

Fig 2: HALLMARK\_G2M\_CHECKPOINT: Random ES distribution      
 Gene set null distribution of ES for **HALLMARK\_G2M\_CHECKPOINT**

  
