# Supplementary material for: Modeling glioblastoma heterogeneity as a dynamic network of cell states
Source: Mol Syst Biol. 2021 Sep 16;17(9):e10105. doi: 10.15252/msb.202010105 (PMC8444284; doi:10.15252/msb.202010105)
Supplement: Supplementary file 6 — Source Data for Figure 5 [file MSB-17-e10105-s004.zip › Figure5A_sourcedata/GSEA_3017/hallmarks_stateB.GseaPreranked.1621934634368/HALLMARK_GLYCOLYSIS.html]

Details for gene set HALLMARK\_GLYCOLYSIS[GSEA]

|  || Dataset | state43017 |
| Phenotype | NoPhenotypeAvailable |
| Upregulated in class | na\_pos |
| GeneSet | HALLMARK\_GLYCOLYSIS |
| Enrichment Score (ES) | 0.38691828 |
| Normalized Enrichment Score (NES) | 1.4322698 |
| Nominal p-value | 0.089121886 |
| FDR q-value | 0.20761603 |
| FWER p-Value | 0.665 |
Table: GSEA Results Summary

  

Fig 1: Enrichment plot: HALLMARK\_GLYCOLYSIS      
 Profile of the Running ES Score & Positions of GeneSet Members on the Rank Ordered List

  

| PROBE | GENE SYMBOL | GENE\_TITLE | RANK IN GENE LIST | RANK METRIC SCORE | RUNNING ES | CORE ENRICHMENT || 1 | CENPA |  |  | 51 | 0.636 | 0.0255 | Yes |
| 2 | AURKA |  |  | 55 | 0.617 | 0.1127 | Yes |
| 3 | CDK1 |  |  | 69 | 0.585 | 0.1819 | Yes |
| 4 | DEPDC1 |  |  | 97 | 0.536 | 0.2248 | Yes |
| 5 | SDC2 |  |  | 107 | 0.513 | 0.2886 | Yes |
| 6 | CD44 |  |  | 146 | 0.464 | 0.3062 | Yes |
| 7 | HMMR |  |  | 191 | 0.426 | 0.3101 | Yes |
| 8 | GPC4 |  |  | 217 | 0.404 | 0.3362 | Yes |
| 9 | CHST2 |  |  | 224 | 0.397 | 0.3869 | Yes |
| 10 | KIF20A |  |  | 392 | 0.311 | 0.2085 | No |
| 11 | PLOD2 |  |  | 474 | 0.286 | 0.1420 | No |
| 12 | SAP30 |  |  | 547 | 0.273 | 0.0855 | No |
| 13 | GMPPB |  |  | 622 | 0.259 | 0.0244 | No |
| 14 | GPC1 |  |  | 651 | 0.256 | 0.0245 | No |
| 15 | STC1 |  |  | 703 | -0.279 | -0.0028 | No |
| 16 | MIF |  |  | 751 | -0.519 | 0.0108 | No |
Table: GSEA details [plain text format]

  

Fig 2: HALLMARK\_GLYCOLYSIS: Random ES distribution      
 Gene set null distribution of ES for **HALLMARK\_GLYCOLYSIS**

  
