# Supplementary material for: Modeling glioblastoma heterogeneity as a dynamic network of cell states
Source: Mol Syst Biol. 2021 Sep 16;17(9):e10105. doi: 10.15252/msb.202010105 (PMC8444284; doi:10.15252/msb.202010105)
Supplement: Supplementary file 6 — Source Data for Figure 5 [file MSB-17-e10105-s004.zip › Figure5A_sourcedata/GSEA_3017/hallmarks_stateB.GseaPreranked.1621934634368/HALLMARK_HYPOXIA.html]

Details for gene set HALLMARK\_HYPOXIA[GSEA]

|  || Dataset | state43017 |
| Phenotype | NoPhenotypeAvailable |
| Upregulated in class | na\_neg |
| GeneSet | HALLMARK\_HYPOXIA |
| Enrichment Score (ES) | -0.18074803 |
| Normalized Enrichment Score (NES) | -0.9180282 |
| Nominal p-value | 0.51741296 |
| FDR q-value | 0.54135394 |
| FWER p-Value | 0.678 |
Table: GSEA Results Summary

  

Fig 1: Enrichment plot: HALLMARK\_HYPOXIA      
 Profile of the Running ES Score & Positions of GeneSet Members on the Rank Ordered List

  

| PROBE | GENE SYMBOL | GENE\_TITLE | RANK IN GENE LIST | RANK METRIC SCORE | RUNNING ES | CORE ENRICHMENT || 1 | SERPINE1 |  |  | 8 | 0.873 | 0.1113 | Yes |
| 2 | CSRP2 |  |  | 96 | 0.539 | 0.0694 | Yes |
| 3 | SDC2 |  |  | 107 | 0.513 | 0.1277 | Yes |
| 4 | ANXA2 |  |  | 211 | 0.408 | 0.0459 | Yes |
| 5 | GPC4 |  |  | 217 | 0.404 | 0.0956 | Yes |
| 6 | CHST2 |  |  | 224 | 0.397 | 0.1431 | Yes |
| 7 | MYH9 |  |  | 273 | 0.362 | 0.1290 | Yes |
| 8 | PDGFB |  |  | 278 | 0.359 | 0.1738 | Yes |
| 9 | AKAP12 |  |  | 320 | 0.339 | 0.1660 | No |
| 10 | CDKN1B |  |  | 427 | 0.298 | 0.0648 | No |
| 11 | IDS |  |  | 465 | 0.289 | 0.0554 | No |
| 12 | SAP30 |  |  | 547 | 0.273 | -0.0157 | No |
| 13 | GPC1 |  |  | 651 | 0.256 | -0.1188 | No |
| 14 | CA12 |  |  | 698 | -0.274 | -0.1424 | No |
| 15 | STC1 |  |  | 703 | -0.279 | -0.1088 | No |
| 16 | PNRC1 |  |  | 726 | -0.348 | -0.0899 | No |
| 17 | BTG1 |  |  | 741 | -0.423 | -0.0496 | No |
| 18 | MIF |  |  | 751 | -0.519 | 0.0108 | No |
Table: GSEA details [plain text format]

  

Fig 2: HALLMARK\_HYPOXIA: Random ES distribution      
 Gene set null distribution of ES for **HALLMARK\_HYPOXIA**

  
