# Supplementary material for: Modeling glioblastoma heterogeneity as a dynamic network of cell states
Source: Mol Syst Biol. 2021 Sep 16;17(9):e10105. doi: 10.15252/msb.202010105 (PMC8444284; doi:10.15252/msb.202010105)
Supplement: Supplementary file 6 — Source Data for Figure 5 [file MSB-17-e10105-s004.zip › Figure5A_sourcedata/GSEA_3017/hallmarks_stateB.GseaPreranked.1621934634368/HALLMARK_MITOTIC_SPINDLE.html]

Details for gene set HALLMARK\_MITOTIC\_SPINDLE[GSEA]

|  || Dataset | state43017 |
| Phenotype | NoPhenotypeAvailable |
| Upregulated in class | na\_pos |
| GeneSet | HALLMARK\_MITOTIC\_SPINDLE |
| Enrichment Score (ES) | 0.5186123 |
| Normalized Enrichment Score (NES) | 2.6313732 |
| Nominal p-value | 0.0 |
| FDR q-value | 0.0 |
| FWER p-Value | 0.0 |
Table: GSEA Results Summary

  

Fig 1: Enrichment plot: HALLMARK\_MITOTIC\_SPINDLE      
 Profile of the Running ES Score & Positions of GeneSet Members on the Rank Ordered List

  

| PROBE | GENE SYMBOL | GENE\_TITLE | RANK IN GENE LIST | RANK METRIC SCORE | RUNNING ES | CORE ENRICHMENT || 1 | TOP2A |  |  | 3 | 1.079 | 0.0386 | Yes |
| 2 | TPX2 |  |  | 12 | 0.801 | 0.0591 | Yes |
| 3 | NUSAP1 |  |  | 13 | 0.801 | 0.0909 | Yes |
| 4 | FBXO5 |  |  | 15 | 0.785 | 0.1207 | Yes |
| 5 | PRC1 |  |  | 18 | 0.756 | 0.1479 | Yes |
| 6 | KIF23 |  |  | 24 | 0.721 | 0.1694 | Yes |
| 7 | NDC80 |  |  | 27 | 0.695 | 0.1942 | Yes |
| 8 | KIF15 |  |  | 29 | 0.684 | 0.2199 | Yes |
| 9 | CENPF |  |  | 34 | 0.670 | 0.2409 | Yes |
| 10 | KIF4A |  |  | 37 | 0.662 | 0.2644 | Yes |
| 11 | ANLN |  |  | 39 | 0.659 | 0.2891 | Yes |
| 12 | SMC4 |  |  | 41 | 0.655 | 0.3137 | Yes |
| 13 | CENPE |  |  | 43 | 0.654 | 0.3383 | Yes |
| 14 | DLGAP5 |  |  | 46 | 0.647 | 0.3611 | Yes |
| 15 | AURKA |  |  | 55 | 0.617 | 0.3744 | Yes |
| 16 | KIF2C |  |  | 66 | 0.591 | 0.3837 | Yes |
| 17 | CDK1 |  |  | 69 | 0.585 | 0.4041 | Yes |
| 18 | RACGAP1 |  |  | 75 | 0.572 | 0.4197 | Yes |
| 19 | PLK1 |  |  | 78 | 0.567 | 0.4394 | Yes |
| 20 | ECT2 |  |  | 84 | 0.558 | 0.4545 | Yes |
| 21 | PIF1 |  |  | 90 | 0.545 | 0.4691 | Yes |
| 22 | BUB1 |  |  | 113 | 0.508 | 0.4583 | Yes |
| 23 | BIRC5 |  |  | 116 | 0.504 | 0.4755 | Yes |
| 24 | TTK |  |  | 137 | 0.482 | 0.4664 | Yes |
| 25 | INCENP |  |  | 140 | 0.476 | 0.4825 | Yes |
| 26 | KIF22 |  |  | 152 | 0.459 | 0.4852 | Yes |
| 27 | CDK5RAP2 |  |  | 173 | 0.438 | 0.4743 | Yes |
| 28 | BRCA2 |  |  | 174 | 0.436 | 0.4916 | Yes |
| 29 | ARHGAP29 |  |  | 181 | 0.434 | 0.5004 | Yes |
| 30 | CKAP5 |  |  | 192 | 0.426 | 0.5032 | Yes |
| 31 | NEK2 |  |  | 194 | 0.423 | 0.5186 | Yes |
| 32 | PCM1 |  |  | 232 | 0.393 | 0.4820 | No |
| 33 | CCNB2 |  |  | 250 | 0.379 | 0.4731 | No |
| 34 | CENPJ |  |  | 261 | 0.370 | 0.4736 | No |
| 35 | MYH9 |  |  | 273 | 0.362 | 0.4725 | No |
| 36 | SMC1A |  |  | 274 | 0.361 | 0.4868 | No |
| 37 | PCNT |  |  | 275 | 0.360 | 0.5011 | No |
| 38 | DLG1 |  |  | 347 | 0.327 | 0.4140 | No |
| 39 | LMNB1 |  |  | 363 | 0.321 | 0.4055 | No |
| 40 | MID1 |  |  | 375 | 0.317 | 0.4026 | No |
| 41 | ACTN4 |  |  | 376 | 0.316 | 0.4152 | No |
| 42 | CEP192 |  |  | 395 | 0.310 | 0.4021 | No |
| 43 | EZR |  |  | 403 | 0.307 | 0.4044 | No |
| 44 | SASS6 |  |  | 432 | 0.296 | 0.3766 | No |
| 45 | KIFAP3 |  |  | 518 | 0.278 | 0.2678 | No |
| 46 | CDC27 |  |  | 520 | 0.278 | 0.2774 | No |
| 47 | SAC3D1 |  |  | 529 | 0.276 | 0.2770 | No |
| 48 | CNTRL |  |  | 575 | 0.269 | 0.2242 | No |
| 49 | RANBP9 |  |  | 576 | 0.269 | 0.2349 | No |
| 50 | CEP57 |  |  | 605 | 0.262 | 0.2058 | No |
| 51 | FLNA |  |  | 676 | 0.251 | 0.1171 | No |
Table: GSEA details [plain text format]

  

Fig 2: HALLMARK\_MITOTIC\_SPINDLE: Random ES distribution      
 Gene set null distribution of ES for **HALLMARK\_MITOTIC\_SPINDLE**

  
