# Supplementary material for: Modeling glioblastoma heterogeneity as a dynamic network of cell states
Source: Mol Syst Biol. 2021 Sep 16;17(9):e10105. doi: 10.15252/msb.202010105 (PMC8444284; doi:10.15252/msb.202010105)
Supplement: Supplementary file 6 — Source Data for Figure 5 [file MSB-17-e10105-s004.zip › Figure5A_sourcedata/GSEA_3017/hallmarks_stateB.GseaPreranked.1621934634368/HALLMARK_MTORC1_SIGNALING.html]

Details for gene set HALLMARK\_MTORC1\_SIGNALING[GSEA]

|  || Dataset | state43017 |
| Phenotype | NoPhenotypeAvailable |
| Upregulated in class | na\_pos |
| GeneSet | HALLMARK\_MTORC1\_SIGNALING |
| Enrichment Score (ES) | 0.269185 |
| Normalized Enrichment Score (NES) | 1.1267734 |
| Nominal p-value | 0.32464454 |
| FDR q-value | 0.40227747 |
| FWER p-Value | 0.983 |
Table: GSEA Results Summary

  

Fig 1: Enrichment plot: HALLMARK\_MTORC1\_SIGNALING      
 Profile of the Running ES Score & Positions of GeneSet Members on the Rank Ordered List

  

| PROBE | GENE SYMBOL | GENE\_TITLE | RANK IN GENE LIST | RANK METRIC SCORE | RUNNING ES | CORE ENRICHMENT || 1 | RRM2 |  |  | 7 | 0.875 | 0.0747 | Yes |
| 2 | DHFR |  |  | 49 | 0.638 | 0.0804 | Yes |
| 3 | AURKA |  |  | 55 | 0.617 | 0.1330 | Yes |
| 4 | IGFBP5 |  |  | 67 | 0.590 | 0.1748 | Yes |
| 5 | PLK1 |  |  | 78 | 0.567 | 0.2157 | Yes |
| 6 | CCNF |  |  | 106 | 0.513 | 0.2284 | Yes |
| 7 | BUB1 |  |  | 113 | 0.508 | 0.2692 | Yes |
| 8 | RPA1 |  |  | 165 | 0.445 | 0.2426 | No |
| 9 | MCM4 |  |  | 199 | 0.418 | 0.2380 | No |
| 10 | TUBG1 |  |  | 268 | 0.365 | 0.1806 | No |
| 11 | PHGDH |  |  | 344 | 0.328 | 0.1102 | No |
| 12 | PLOD2 |  |  | 474 | 0.286 | -0.0378 | No |
| 13 | NUP205 |  |  | 571 | 0.269 | -0.1425 | No |
| 14 | IFRD1 |  |  | 594 | 0.264 | -0.1470 | No |
| 15 | RPN1 |  |  | 662 | 0.254 | -0.2138 | No |
| 16 | UCHL5 |  |  | 671 | 0.252 | -0.2004 | No |
| 17 | HMGCR |  |  | 683 | -0.257 | -0.1906 | No |
| 18 | SCD |  |  | 688 | -0.263 | -0.1708 | No |
| 19 | STC1 |  |  | 703 | -0.279 | -0.1630 | No |
| 20 | PRDX1 |  |  | 719 | -0.326 | -0.1520 | No |
| 21 | IDI1 |  |  | 734 | -0.397 | -0.1329 | No |
| 22 | SQLE |  |  | 735 | -0.405 | -0.0939 | No |
| 23 | HMGCS1 |  |  | 739 | -0.418 | -0.0577 | No |
| 24 | PPA1 |  |  | 742 | -0.424 | -0.0196 | No |
| 25 | MLLT11 |  |  | 744 | -0.430 | 0.0204 | No |
Table: GSEA details [plain text format]

  

Fig 2: HALLMARK\_MTORC1\_SIGNALING: Random ES distribution      
 Gene set null distribution of ES for **HALLMARK\_MTORC1\_SIGNALING**

  
