# Supplementary material for: Modeling glioblastoma heterogeneity as a dynamic network of cell states
Source: Mol Syst Biol. 2021 Sep 16;17(9):e10105. doi: 10.15252/msb.202010105 (PMC8444284; doi:10.15252/msb.202010105)
Supplement: Supplementary file 6 — Source Data for Figure 5 [file MSB-17-e10105-s004.zip › Figure5A_sourcedata/GSEA_3017/hallmarks_stateB.GseaPreranked.1621934634368/HALLMARK_MYC_TARGETS_V1.html]

Details for gene set HALLMARK\_MYC\_TARGETS\_V1[GSEA]

|  || Dataset | state43017 |
| Phenotype | NoPhenotypeAvailable |
| Upregulated in class | na\_pos |
| GeneSet | HALLMARK\_MYC\_TARGETS\_V1 |
| Enrichment Score (ES) | 0.3439196 |
| Normalized Enrichment Score (NES) | 1.403078 |
| Nominal p-value | 0.12254902 |
| FDR q-value | 0.19789568 |
| FWER p-Value | 0.722 |
Table: GSEA Results Summary

  

Fig 1: Enrichment plot: HALLMARK\_MYC\_TARGETS\_V1      
 Profile of the Running ES Score & Positions of GeneSet Members on the Rank Ordered List

  

| PROBE | GENE SYMBOL | GENE\_TITLE | RANK IN GENE LIST | RANK METRIC SCORE | RUNNING ES | CORE ENRICHMENT || 1 | KPNA2 |  |  | 33 | 0.676 | 0.0332 | Yes |
| 2 | CCNA2 |  |  | 54 | 0.626 | 0.0782 | Yes |
| 3 | TYMS |  |  | 63 | 0.601 | 0.1366 | Yes |
| 4 | MAD2L1 |  |  | 77 | 0.569 | 0.1845 | Yes |
| 5 | RRM1 |  |  | 103 | 0.520 | 0.2105 | Yes |
| 6 | DUT |  |  | 124 | 0.494 | 0.2402 | Yes |
| 7 | CDC45 |  |  | 154 | 0.456 | 0.2535 | Yes |
| 8 | CDC20 |  |  | 184 | 0.431 | 0.2639 | Yes |
| 9 | USP1 |  |  | 185 | 0.430 | 0.3134 | Yes |
| 10 | MCM4 |  |  | 199 | 0.418 | 0.3439 | Yes |
| 11 | PCNA |  |  | 242 | 0.386 | 0.3315 | No |
| 12 | RFC4 |  |  | 295 | 0.351 | 0.3016 | No |
| 13 | DEK |  |  | 316 | 0.341 | 0.3138 | No |
| 14 | XPO1 |  |  | 365 | 0.321 | 0.2858 | No |
| 15 | TFDP1 |  |  | 444 | 0.294 | 0.2140 | No |
| 16 | CDK2 |  |  | 565 | 0.270 | 0.0827 | No |
| 17 | IFRD1 |  |  | 594 | 0.264 | 0.0752 | No |
| 18 | KPNB1 |  |  | 600 | 0.263 | 0.0987 | No |
| 19 | UBA2 |  |  | 626 | 0.259 | 0.0947 | No |
| 20 | HSP90AB1 |  |  | 701 | -0.277 | 0.0265 | No |
| 21 | DDX21 |  |  | 745 | -0.440 | 0.0189 | No |
Table: GSEA details [plain text format]

  

Fig 2: HALLMARK\_MYC\_TARGETS\_V1: Random ES distribution      
 Gene set null distribution of ES for **HALLMARK\_MYC\_TARGETS\_V1**

  
