# Supplementary material for: Modeling glioblastoma heterogeneity as a dynamic network of cell states
Source: Mol Syst Biol. 2021 Sep 16;17(9):e10105. doi: 10.15252/msb.202010105 (PMC8444284; doi:10.15252/msb.202010105)
Supplement: Supplementary file 6 — Source Data for Figure 5 [file MSB-17-e10105-s004.zip › Figure5A_sourcedata/GSEA_3017/hallmarks_stateB.GseaPreranked.1621934634368/HALLMARK_SPERMATOGENESIS.html]

Details for gene set HALLMARK\_SPERMATOGENESIS[GSEA]

|  || Dataset | state43017 |
| Phenotype | NoPhenotypeAvailable |
| Upregulated in class | na\_pos |
| GeneSet | HALLMARK\_SPERMATOGENESIS |
| Enrichment Score (ES) | 0.49186653 |
| Normalized Enrichment Score (NES) | 1.7641675 |
| Nominal p-value | 0.011435832 |
| FDR q-value | 0.033112988 |
| FWER p-Value | 0.124 |
Table: GSEA Results Summary

  

Fig 1: Enrichment plot: HALLMARK\_SPERMATOGENESIS      
 Profile of the Running ES Score & Positions of GeneSet Members on the Rank Ordered List

  

| PROBE | GENE SYMBOL | GENE\_TITLE | RANK IN GENE LIST | RANK METRIC SCORE | RUNNING ES | CORE ENRICHMENT || 1 | AURKA |  |  | 55 | 0.617 | 0.0160 | Yes |
| 2 | KIF2C |  |  | 66 | 0.591 | 0.0884 | Yes |
| 3 | CDK1 |  |  | 69 | 0.585 | 0.1709 | Yes |
| 4 | DBF4 |  |  | 79 | 0.567 | 0.2412 | Yes |
| 5 | NCAPH |  |  | 94 | 0.543 | 0.3014 | Yes |
| 6 | CDKN3 |  |  | 108 | 0.513 | 0.3585 | Yes |
| 7 | BUB1 |  |  | 113 | 0.508 | 0.4271 | Yes |
| 8 | TTK |  |  | 137 | 0.482 | 0.4663 | Yes |
| 9 | NEK2 |  |  | 194 | 0.423 | 0.4527 | Yes |
| 10 | EZH2 |  |  | 236 | 0.388 | 0.4542 | Yes |
| 11 | CCNB2 |  |  | 250 | 0.379 | 0.4919 | Yes |
| 12 | RFC4 |  |  | 295 | 0.351 | 0.4839 | No |
| 13 | TOPBP1 |  |  | 369 | 0.319 | 0.4324 | No |
| 14 | PARP2 |  |  | 372 | 0.319 | 0.4760 | No |
| 15 | HSPA4L |  |  | 461 | 0.290 | 0.4000 | No |
Table: GSEA details [plain text format]

  

Fig 2: HALLMARK\_SPERMATOGENESIS: Random ES distribution      
 Gene set null distribution of ES for **HALLMARK\_SPERMATOGENESIS**

  
