# Supplementary material for: Modeling glioblastoma heterogeneity as a dynamic network of cell states
Source: Mol Syst Biol. 2021 Sep 16;17(9):e10105. doi: 10.15252/msb.202010105 (PMC8444284; doi:10.15252/msb.202010105)
Supplement: Supplementary file 6 — Source Data for Figure 5 [file MSB-17-e10105-s004.zip › Figure5A_sourcedata/GSEA_3017/hallmarks_stateB.GseaPreranked.1621934634368/HALLMARK_UV_RESPONSE_DN.html]

Details for gene set HALLMARK\_UV\_RESPONSE\_DN[GSEA]

|  || Dataset | state43017 |
| Phenotype | NoPhenotypeAvailable |
| Upregulated in class | na\_pos |
| GeneSet | HALLMARK\_UV\_RESPONSE\_DN |
| Enrichment Score (ES) | 0.26309577 |
| Normalized Enrichment Score (NES) | 0.9515486 |
| Nominal p-value | 0.5030979 |
| FDR q-value | 0.5233297 |
| FWER p-Value | 1.0 |
Table: GSEA Results Summary

  

Fig 1: Enrichment plot: HALLMARK\_UV\_RESPONSE\_DN      
 Profile of the Running ES Score & Positions of GeneSet Members on the Rank Ordered List

  

| PROBE | GENE SYMBOL | GENE\_TITLE | RANK IN GENE LIST | RANK METRIC SCORE | RUNNING ES | CORE ENRICHMENT || 1 | SERPINE1 |  |  | 8 | 0.873 | 0.1271 | Yes |
| 2 | IGFBP5 |  |  | 67 | 0.590 | 0.1422 | Yes |
| 3 | PMP22 |  |  | 70 | 0.576 | 0.2305 | Yes |
| 4 | SDC2 |  |  | 107 | 0.513 | 0.2631 | Yes |
| 5 | ANXA2 |  |  | 211 | 0.408 | 0.1890 | No |
| 6 | DDAH1 |  |  | 300 | 0.346 | 0.1254 | No |
| 7 | DLG1 |  |  | 347 | 0.327 | 0.1152 | No |
| 8 | ID1 |  |  | 390 | 0.311 | 0.1079 | No |
| 9 | CDKN1B |  |  | 427 | 0.298 | 0.1066 | No |
| 10 | LAMC1 |  |  | 438 | 0.295 | 0.1396 | No |
| 11 | DYRK1A |  |  | 450 | 0.293 | 0.1711 | No |
| 12 | SYNE1 |  |  | 458 | 0.291 | 0.2076 | No |
| 13 | COL5A2 |  |  | 516 | 0.278 | 0.1749 | No |
| 14 | PRKAR2B |  |  | 611 | 0.262 | 0.0898 | No |
| 15 | MMP16 |  |  | 707 | -0.285 | 0.0071 | No |
| 16 | HAS2 |  |  | 733 | -0.389 | 0.0349 | No |
Table: GSEA details [plain text format]

  

Fig 2: HALLMARK\_UV\_RESPONSE\_DN: Random ES distribution      
 Gene set null distribution of ES for **HALLMARK\_UV\_RESPONSE\_DN**

  
