# Supplementary material for: Modeling glioblastoma heterogeneity as a dynamic network of cell states
Source: Mol Syst Biol. 2021 Sep 16;17(9):e10105. doi: 10.15252/msb.202010105 (PMC8444284; doi:10.15252/msb.202010105)
Supplement: Supplementary file 6 — Source Data for Figure 5 [file MSB-17-e10105-s004.zip › Figure5A_sourcedata/GSEA_3017/hallmarks_stateC.GseaPreranked.1621934520804/gsea_report_for_na_neg_1621934520804.html]

Report for na\_neg 1621934520804 [GSEA]

| GS  follow link to MSigDB | GS DETAILS | SIZE | ES | NES | NOM p-val | FDR q-val | FWER p-val | RANK AT MAX | LEADING EDGE || 1 | HALLMARK\_TNFA\_SIGNALING\_VIA\_NFKB | Details ... | 35 | -0.48 | -2.45 | 0.000 | 0.001 | 0.001 | 304 | tags=94%, list=51%, signal=181% |
| 2 | HALLMARK\_EPITHELIAL\_MESENCHYMAL\_TRANSITION | Details ... | 38 | -0.46 | -2.45 | 0.002 | 0.001 | 0.001 | 246 | tags=79%, list=41%, signal=126% |
| 3 | HALLMARK\_IL2\_STAT5\_SIGNALING | Details ... | 24 | -0.52 | -2.38 | 0.000 | 0.002 | 0.005 | 177 | tags=71%, list=30%, signal=97% |
| 4 | HALLMARK\_CHOLESTEROL\_HOMEOSTASIS | Details ... | 18 | -0.55 | -2.28 | 0.002 | 0.003 | 0.008 | 180 | tags=72%, list=30%, signal=100% |
| 5 | HALLMARK\_INFLAMMATORY\_RESPONSE | Details ... | 19 | -0.51 | -2.14 | 0.000 | 0.004 | 0.017 | 306 | tags=100%, list=51%, signal=199% |
| 6 | HALLMARK\_HYPOXIA | Details ... | 28 | -0.42 | -2.02 | 0.002 | 0.007 | 0.029 | 221 | tags=71%, list=37%, signal=108% |
| 7 | HALLMARK\_UV\_RESPONSE\_DN | Details ... | 17 | -0.50 | -1.98 | 0.000 | 0.008 | 0.040 | 186 | tags=76%, list=31%, signal=108% |
| 8 | HALLMARK\_COMPLEMENT | Details ... | 15 | -0.51 | -1.96 | 0.003 | 0.008 | 0.046 | 275 | tags=93%, list=46%, signal=169% |
| 9 | HALLMARK\_ESTROGEN\_RESPONSE\_LATE | Details ... | 21 | -0.38 | -1.68 | 0.027 | 0.051 | 0.279 | 88 | tags=33%, list=15%, signal=38% |
| 10 | HALLMARK\_APOPTOSIS | Details ... | 20 | -0.38 | -1.61 | 0.036 | 0.068 | 0.385 | 65 | tags=35%, list=11%, signal=38% |
| 11 | HALLMARK\_KRAS\_SIGNALING\_UP | Details ... | 15 | -0.41 | -1.57 | 0.047 | 0.077 | 0.452 | 80 | tags=40%, list=13%, signal=45% |
| 12 | HALLMARK\_MYOGENESIS | Details ... | 17 | -0.39 | -1.57 | 0.054 | 0.073 | 0.464 | 126 | tags=47%, list=21%, signal=58% |
| 13 | HALLMARK\_GLYCOLYSIS | Details ... | 16 | -0.40 | -1.55 | 0.059 | 0.075 | 0.505 | 162 | tags=56%, list=27%, signal=75% |
| 14 | HALLMARK\_MTORC1\_SIGNALING | Details ... | 25 | -0.34 | -1.54 | 0.080 | 0.071 | 0.512 | 215 | tags=64%, list=36%, signal=96% |
| 15 | HALLMARK\_ESTROGEN\_RESPONSE\_EARLY | Details ... | 23 | -0.30 | -1.33 | 0.149 | 0.168 | 0.843 | 311 | tags=74%, list=52%, signal=148% |
| 16 | HALLMARK\_ANDROGEN\_RESPONSE | Details ... | 16 | -0.33 | -1.26 | 0.208 | 0.211 | 0.924 | 180 | tags=56%, list=30%, signal=78% |
| 17 | HALLMARK\_P53\_PATHWAY | Details ... | 16 | -0.29 | -1.12 | 0.324 | 0.323 | 0.984 | 221 | tags=56%, list=37%, signal=87% |
Table: Gene sets enriched in phenotype **na**[plain text format]****

  
