# Supplementary material for: Modeling glioblastoma heterogeneity as a dynamic network of cell states
Source: Mol Syst Biol. 2021 Sep 16;17(9):e10105. doi: 10.15252/msb.202010105 (PMC8444284; doi:10.15252/msb.202010105)
Supplement: Supplementary file 6 — Source Data for Figure 5 [file MSB-17-e10105-s004.zip › Figure5A_sourcedata/GSEA_3017/hallmarks_stateC.GseaPreranked.1621934520804/gsea_report_for_na_pos_1621934520804.html]

Report for na\_pos 1621934520804 [GSEA]

| GS  follow link to MSigDB | GS DETAILS | SIZE | ES | NES | NOM p-val | FDR q-val | FWER p-val | RANK AT MAX | LEADING EDGE || 1 | HALLMARK\_E2F\_TARGETS | Details ... | 40 | 0.70 | 4.46 | 0.000 | 0.000 | 0.000 | 191 | tags=98%, list=32%, signal=134% |
| 2 | HALLMARK\_G2M\_CHECKPOINT | Details ... | 28 | 0.58 | 3.24 | 0.000 | 0.000 | 0.000 | 194 | tags=89%, list=32%, signal=126% |
Table: Gene sets enriched in phenotype **na**[plain text format]****

  
